# Desymmetrization of Bisallylic Amides: A Catalytic Enantioselective Diastereoselective Chlorocyclization Strategy

**DOI:** 10.1021/acs.orglett.5c01865

**Published:** 2025-07-01

**Authors:** Yi Yi, Ankush Chakraborty, Xinliang Ding, Arvind Jaganathan, Neil Heberer, Babak Borhan

**Affiliations:** Department of Chemistry, 3078 Michigan State University, East Lansing, Michigan 48824, United States

## Abstract

(DHQD)_2_PHAL-catalyzed diastereo­selective and enantio­selective chloro­cyclization reactions of bisallylic amides were achieved with *N*-chloro­phthal­imide (NCP) as the chlorinating reagent. A series of chlorinated oxazines bearing three contiguous chiral centers were obtained in high yields (up to 99%), high diastereo­selectivity and excellent enantio­selectivity (up to 99% *ee*).

## Graphical abstract

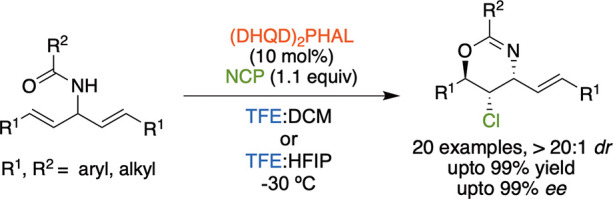

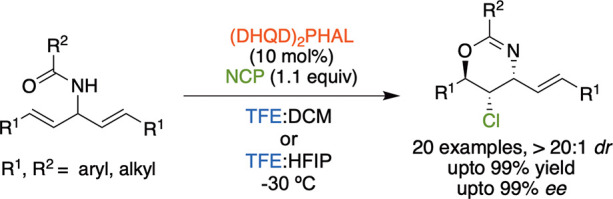

Enantioselective desymmetrization of symmetric dienes offers a unique opportunity for the construction of stereocenters prevalent in naturally occurring biologically active molecules, as it enables the generation of multiple stereogenic centers in a single step.^1^ The unreacted alkene can be further functionalized to generate complex targets with up to five contiguous stereocenters. Along this line, enantio­selective desymmetrization of bis-allylic amides enables the conversion of relatively simple achiral starting materials to high-value enantio-enriched oxazines. The 1,3-oxazine framework is often encountered in biologically active molecules with a range of pharmaco­phoric activities, such as antimicrobial, analgesic, antitumor, anti­spasmodic, to name a few.^2^ These motifs have been utilized as fundamental scaffolds for the construction of chiral ligands.^3^

Although there have been several reports on asymmetric desymmetrization of dienes,^1,4^ organo­catalyzed halo-desymmetrization strategies to synthesize these motifs are rare. Hennecke and co-workers reported (DHQD)_2_PHAL-catalyzed bromo­lactonization of symmetric dialkynes (Figure 1a).^5^ Yeung’s group also developed a protocol for the desymmetrization of olefinic 1,3-dicarbonyl compounds via bromo­cyclization using quinine-derived thiocarbamate catalysts.^6^ Previously, our group developed a highly stereo­selective strategy to form oxazine derivatives via chloro­cyclization of unsaturated amides catalyzed by (DHQD)_2_PHAL (Figure 1b).^7^ In 2017, Hamashima and co-workers reported the BINAP monoxide-catalyzed desymmetrization of bisallylic amides through bromo­cyclization and further investigated its mechanistic details and extended their strategy to parallel kinetic resolution (Figure 1c).^8^ We also had previously described the development of a new methodology for the kinetic resolution of unsaturated amides, where it was surmised that (DHQD)_2_PHAL accelerates the reaction rate and imparts high facial selectivity for a single enantiomer during halocyclization.^7b^ Therefore, we envisioned an extension of this concept wherein the development of a highly enantio- and diastereo­selective organo­catalytic desymmetrization protocol on a substituted olefin would generate oxazines along with the concomitant formation of three contiguous stereocenters (Figure 1d).

**Figure 1:**
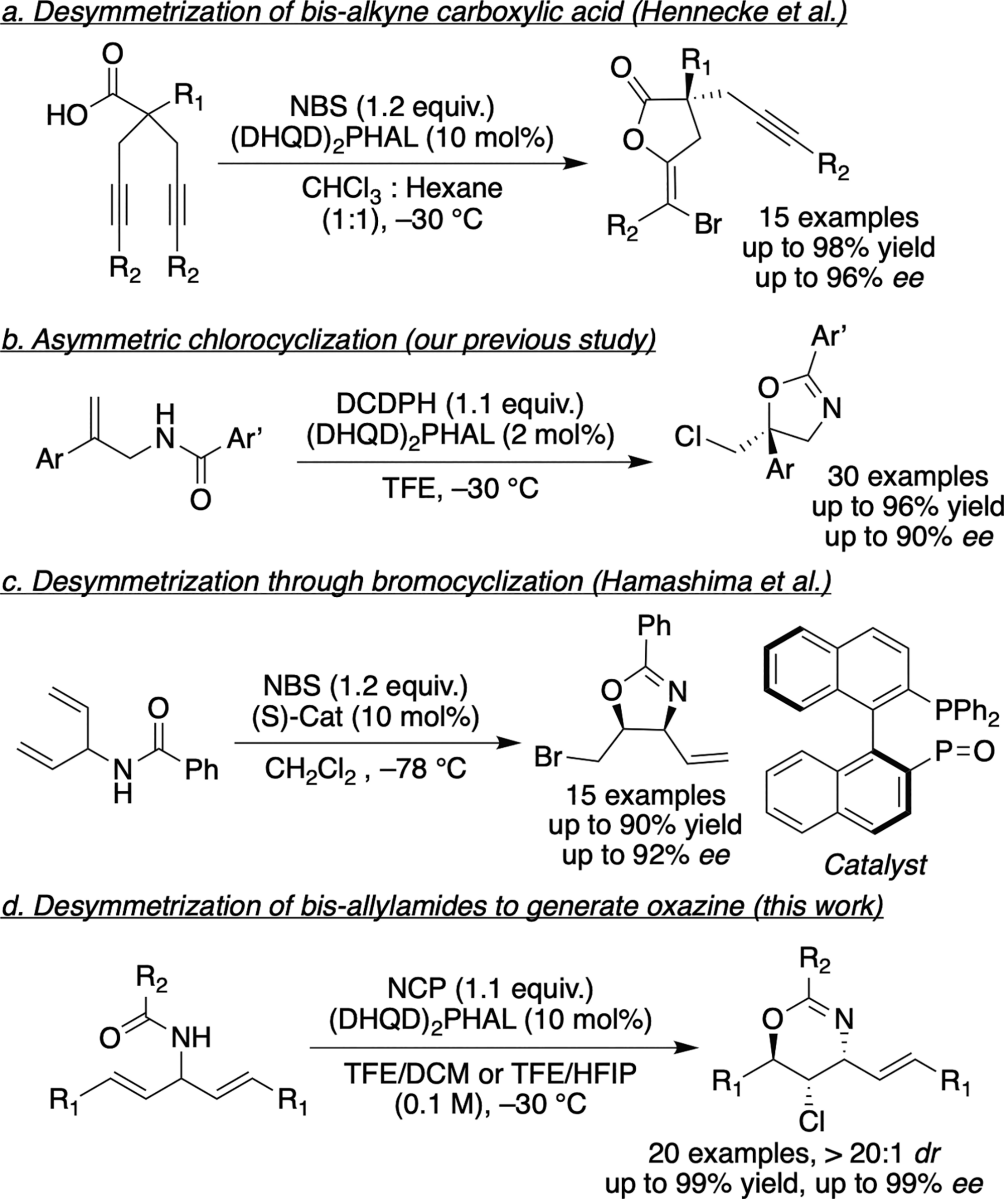
(a) Desymmetrization to bromolactones. (b) Asymmetric chlorination to oxazines. (c) Oxazines through desymmetrization. (d) Desymmetrization of bis-allyl amides to oxazines.

Initial optimizations were performed on the substrate bisphenyl­allylic amide **1a** (Table 1). Reaction of **1a** at room temperature in trifluoro­ethanol (TFE) with *N*-chloro­phthal­imide (NCP) as the electro­philic chlorine source and catalytic (DHQD)_2_PHAL led to the isolation of product **2a** in 61% yield with a high *er* (96:4), albeit with only 76% conversion. Incomplete conversion and a considerable amount of TFE incorporated product **3a** (10%) initiated screening studies for further optimization. Use of hexafluoro­isopropanol (HFIP) as solvent accelerated the reaction and led to higher yields without the incorporation of the alcoholic solvent; however, the enantio­selectivity and diastereo­selectivity eroded (Table 1, entry 2). Combinations of TFE and HFIP as the solvent system were pursued, ultimately reaching a 7:3 ratio as optimal (entry 3). Lowering the temperature increased the *dr* and *er* of the product, but the yield suffered, even at longer reaction times (entries 4 and 5). This may be due to the reduced solubility of the substrate in HFIP at lower temperatures. Increasing the loading of the halogenating agent was pursued to increase the yield of **2a**. Larger equivalents led to the formation of the undesired product **3a**, but employing 1.1 equiv gave better yield, *dr* and *er* of the oxazine (entries 6–8).

**Table 1:**
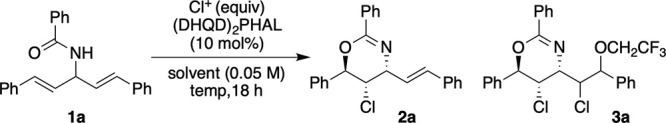
Optimization Studies for the Desymmetrization of Bis-allyl Amide **1a**

				yield^b^ (%)			
entry^a^	Cl^+^ (equiv)	temp (°C)	solvent (ratio)	**2a**	**3a**	*dr* **2a** ^c^	*er* **2a** ^d^
1	NCP (1.0)	rt	TFE	61 (76)	10	6.7:1	96:4
2^e^	NCP (1.0)	rt	HFIP	95 (100)		4.5:1	95:5
3	NCP (1.0)	rt	TFE:HFIP (7:3)	91 (100)		4.5:1	95:5
4^f^	NCP (1.0)	–10	TFE:HFIP (7:3)	69 (70)		6.7:1	96:4
5^f^	NCP (1.0)	–30	TFE:HFIP (7:3)	55(70)		10:1	98:2
6	NCP (1.1)	–30	TFE:HFIP (7:3)	65 (80)		10:1	99:1
7	NCP (1.3)	–30	TFE:HFIP (7:3)	29 (67)	31		96:4
8	NCP (2.0)	–30	TFE:HFIP (7:3)	32(100)	34		
9	NCS (1.1)	–30	TFE:HFIP (7:3)	52(100)	10		
10	DCDMH (1.1)	–30	TFE:HFIP (1:1)	15 (100)			
11^g^	NCP (1.1)	–30	TFE:HFIP (7:3)	59 (70)		>20:1	99:1
12^h^	NCP (1.1)	–30	TFE:HFIP (7:3)	73 (88)		16:1	99:1
**13** ^i^	**NCP (1.1)**	–**30**	**TFE:HFIP**(7:3)	90 (100)	**0**	>20:1	99:1
14	NCP (1.1)	–30	TFE:ACN (7:3)	68 (85)		>20:1	92:8
**15**	**NCP (1.1)**	**–30**	**TFE:DCM**(7:3)	91 (100)	**0**	>20:1	99:1
16	NCP (1.1)	–30	DCM	0 (0)	0		
17^f^ ^,^ ^j^	NCP (1.1)	–30	TFE:HFIP (7:3)	0 (0)	0		

a Reactions were performed on a 0.25 mmol scale.

b Total isolated yields are reported; numbers in parentheses are conversions determined by ^1^H NMR analysis of crude using MTBE as internal standard.

c *dr* was determined by ^1^H NMR.

d *ee* was determined by chiral HPLC.

e Reaction was complete in 2 h.

f Reaction time was 24 h.

g Zn­(OTf)_2_ was used as an additive.

h AgOTf was used as an additive.

i Yt­(OTf)_3_ was used as an additive.

j No catalyst.

Other chlorinating agents such as NCS and DCDMH were utilized to further increase the yield and *dr* of the reaction, but these efforts were not fruitful (entries 9 and 10).^9^ It was hypothesized that additives, primarily Lewis acids, might affect the reaction rates as coordination with the chlorinating agent could result in a faster chlorenium transfer.^10^ Zn­(OTf)_2_ and AgOTf afforded good to excellent *dr* and *er* values for **2a**, albeit with reduced yields. Use of 10 mol% of Y­(OTf)_3_, afforded **2a** in 90% yield with excellent *dr* and *er* (entries 11–13, see SI for the complete list of additives). Although a number of additives including Lewis acids were examined, the ideal combination of yield, *dr* and *er* were attained when a 7:3 mixture of TFE:DCM was used as the solvent system (entries 14, 15, see SI for other solvent system combinations). Interestingly, no product was observed with DCM as the sole solvent after 24 h (Table 1, entry 16, recovered **1a**), highlighting the crucial role of TFE in facilitating the catalysis.^7b^ The background reaction is also not of concern, as no discernible reaction was observed in the absence of the catalyst at −30 °C, even after 24 h (entry 17). We later found that a 7:3 solvent mixture of TFE:HFIP system gave higher *er* and *dr* for some substrates. As such, both solvent systems were examined for substrate scope studies (see SI). Generally, cleaner reactions were observed in TFE:DCM solvent systems, presumably due to better solubility of the substrate at −30 °C.

With the optimized reaction conditions in hand, we examined the generality of the chlorocyclization protocol with various symmetric bisallylic amides (Figure 2). Substrates containing substitutions on the phenyl ring were evaluated in terms of both electronics as well as sterics. Most of the halogenated substrates (**1b**–**1f**) gave excellent *dr* and *er* values. Nonetheless, 2,6-dichlorophenyl-substituted diene (**1g**) was less efficient when subjected to the same conditions, delivering the corresponding chloro-oxazines in reduced enantio­induction. Substrates containing electron-withdrawing substituents such as **1h** delivered the corresponding product **2h** in great yield and with excellent diastereo­selectivity in both solvent systems. With the pseudo­enantiomeric catalyst (DHQ)_2_PHAL, the enantiomeric product was also obtained in high yield with excellent *er* and *dr* (**1i**, **1j**). Substrates bearing electron-donating groups (EDGs) such as methyl (**1k**), methoxy (**1l**) and *tert*-butyl (**1m**) in the *para*-position delivered their corresponding products in only moderate yields with poor diastereo­selectivities but good enantio­selectivities. This was not surprising as our previous results have also shown that the presence of an EDG stabilizes the positive charge at the benzylic position, resulting in reduced enantio­selection.^10a,11^ *Ortho*-substitution (**1j**) gave a high yield and *er* but low *dr* in TFE:DCM (6:1 *dr*, see SI), but appreciably higher *dr* (20:1) in TFE:HFIP (7:3). The reaction proceeded slowly with thiophene-substituted substrate **1n**, yielding the chloro-oxazine product with moderate *dr* and *er* in 26% yield and 18% recovered starting material. Simple alkyl-substituted *trans*-alkenes were well tolerated, giving high yields, *dr* and *er* (see **1o** and **1p**); however, a significant decrease in enantio­selectivity was observed with the bulky *tert*-butyl-substituted alkene **1q**, possibly due to an increased steric interaction during cyclization. Other *N*-acyl amides were also tested to better understand the scope of this methodology. While *p-*bromobenzoyl **1t** and *p-*methoxy benzoyl **1s** substrates delivered the final chloro-oxazine product in good yields, high *dr* and excellent *er* values, isobutyramide substrate **1r** generated the oxazine **2r** as a nearly racemic product under standard conditions. The alkyl­amide in substrate **1r** lacks the ability to form π–π stacking interactions with the catalyst, which might suggest a key role between the arylamide substituent and the catalyst for achieving high enantio­selectivity during the halocyclization.^7b,11^

**Figure 2:**
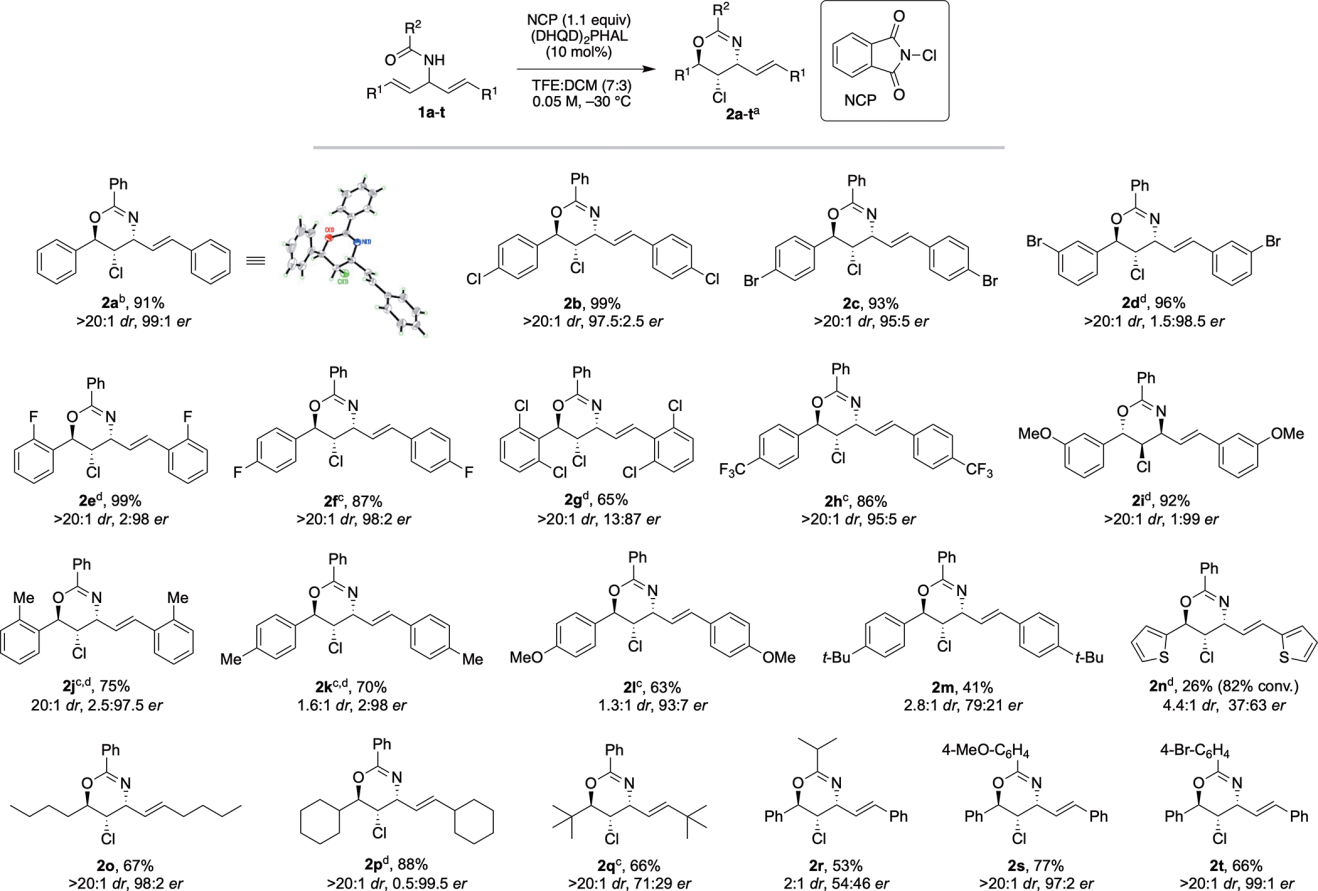
Substrate scope for the chlorocyclization of bisallylic amides^a^ to 1,3-oxazines. ^a^Reactions were performed on 0.024–0.4 mmol scale; total isolated yields are reported; *ee* is determined by chiral HPLC analysis. ^b^Absolute configuration was determined by X-ray crystallographic analysis; all other products are assigned by analogy. ^c^TFE/HFIP (v/v, 7:3, 0.05 M) as solvent. ^d^(DHQ)_2_PHAL was used as catalyst.

The oxazine product with a pendant alkene moiety presents the opportunity for further functionalization of the alkenyl moiety, potentially granting access to systems with 5-contiguous stereocenters. The synthetic utility of the products obtained from desymmetrization of the bisallylic substrates was demonstrated with **2a**, when it was subjected to a number of different transformations (Figure 3). Upjohn dihydroxylation of **2a** afforded compound **4** in 86% yield with excellent diastereo­selection (>20:1) containing five contiguous stereocenters. Epoxidation of **2a** with *m*CPBA generated compound **5** in 67% yield with a high diastereo­selectivity (16:1). The absolute stereochemistries of diol **4** and epoxide **5** were determined by X-ray crystallography (see SI). The structurally important 1,3-amino alcohols that comprise the core of many biologically active natural products could be obtained upon hydrolysis in high yields without the erosion of original stereochemistry (see **2a** to **6**). Gram-scale reaction of **1a** under the optimized conditions proceeded similarly, furnishing product **2a** in 82% yield, with >20:1 *dr* and comparable *er* (98:02).

**Figure 3:**
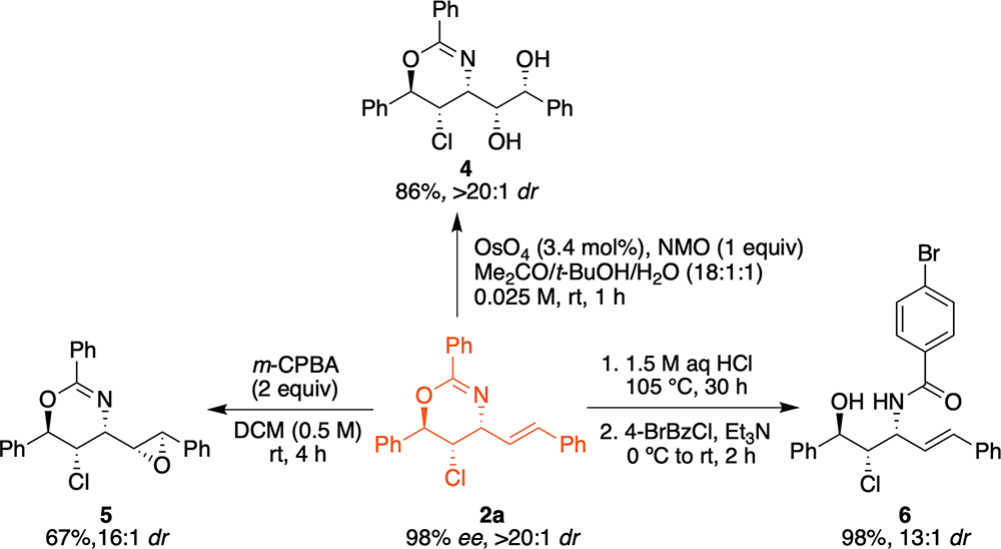
Diastereoselective reactions to further functionalize **2a**.

In conclusion, an efficient desymmetrization of bisallylic amides mediated by (DHQD)_2_PHAL-catalyzed chlorocyclization has been developed. This method works well on a variety of aromatic and alkyl-substituted dienes, allowing access to a highly functionalized chiral oxazine scaffold. The synthetic utility of the reaction is highlighted by the transformation of the products into highly functionalized molecules.

## Supplementary Material



## Data Availability

The data underlying this study are available in the published article and its Supporting Information.

## Notes

Experimental procedures, NMRs, X-ray structure factors, and HPLC traces (PDF)

## †

Revolution Medicines, Redwood City, California 94063, United States
