# Supplementary material for: Desymmetrization of Bisallylic Amides: A Catalytic Enantioselective Diastereoselective Chlorocyclization Strategy
Source: Org Lett. 2025 Jul 1;27(27):7297–301. doi: 10.1021/acs.orglett.5c01865 (PMC12261326; doi:10.1021/acs.orglett.5c01865)
Supplement: Supplementary file 1 [file ol5c01865_si_001.pdf]

Supporting Information for

Desymmetrization of Bisallylic Amides: A Catalytic Enantioselective Diastereoselective Chlorocyclization Strategy

Yi Yi,‡ Ankush Chakraborty,‡ Xinliang Ding, Arvind Jaganathan, Neil Heberer and Babak Borhan*

Department of Chemistry, Michigan State University, East Lansing, MI 48824, United States
Correspondence to: babak@chemistry.msu.edu

Table of Contents

1. General Information	S3
2. Synthesis of Bisallylic Amide Substrates	S5
3. Optimization of the Reaction Conditions for Desymmetrization	S24
4. Enantioselective Desymmetrization of Bisallylic Amides	S29
5. General Method for Growing Crystals	S49
6. General Method for Gram-Scale Reaction	S49
7. Comparison of Two Solvent Systems	S51
8. Derivatization of Oxazine Product	S52
9. X-Ray Crystal Structures	S56
10. Reference	S80
11. HPLC Data	S82
12. NMR Spectral Data	S109

1. General Information

General Procedures. All reactions were performed in oven-dried or flame-dried round-bottom flasks. The flasks were fitted with rubber septa and reactions were conducted under a positive pressure of nitrogen. Gas-tight syringes with stainless steel needles or cannula were used to transfer air- and moisture-sensitive liquids. Where necessary (so noted), solutions were deoxygenated by sparging with nitrogen for a minimum of 10 min. Flash column chromatography was performed as described by Still *et al.*[1] using granular silica gel (SiliCycle, silica gel 60, 32-63 μm). Analytical thin layer chromatography (TLC) was performed using glass plates pre-coated with 0.20 mm 230–400 mesh silica gel impregnated with a fluorescent indicator (254 nm). Visualization was by short wave (254 nm) and long wave (365 nm) ultraviolet light, or by staining with phosphomolybdic acid in ethanol. TLC plates were visualized by exposure to short wave ultraviolet light (254 nm) and an aqueous solution of basic $KMnO_4$ followed by heating on a hot plate (~250 ºC). Organic solutions were concentrated at 29–30 ºC on rotary evaporators capable of achieving a minimum pressure of ~2 torr.

Materials. All reagents were purchased from commercial sources and were used without purification. Unless otherwise specified, all solvents were strictly dried before use: dichloromethane was distilled over calcium hydride under nitrogen; tetrahydrofuran, diethyl ether were distilled from sodium and benzophenone; toluene was distilled over sodium under a nitrogen atmosphere. Hexane and ethyl acetate were ACS grade and used as purchased. $(DHQD)_2PHAL$ and *N*-chlorophthalimide were purchased from Sigma Aldrich. Trifluoroethanol and hexafluoroisopropanol were purchased from Combi-Blocks.

Instrumentation. Melting points (mp) were recorded on a Thomas Hoover capillary melting point apparatus and are uncorrected. Proton nuclear magnetic resonance (^{1}H NMR) spectra were recorded with a Varian Unity Plus 500 MHz spectrometer using $CDCl_3$ as solvent (unless otherwise noted). The residual peak of $CDCl_3$ or TMS was used as the internal standard for both ^{1}H NMR (δ = 7.24 ppm for $CDCl_3$ or δ = 0 ppm for TMS) and ^{13}C NMR (δ = 77.0 ppm). Chemical shifts are reported in parts per million on the δ scale and are referenced from the residual protium in the NMR solvent ($CDCl_3$: δ 7.26 ($CHCl_3$), CD_3OD: δ 3.31 (CD_3OH), DMSO-d_6: δ 2.50 (DMSO-d_5)). Data are reported as

follows: chemical shift [multiplicity (s = singlet, d = doublet, t = triplet, sp = septet, m = multiplet), coupling constant(s) in Hertz, integration, assignment]. Carbon-13 nuclear magnetic resonance (^{13}C NMR) spectra were recorded with a Varian 500 INOVA spectrometer using $CDCl_3$ as solvent (unless otherwise noted). Chemical shifts are reported in parts per million on the δ scale and are referenced from the carbon resonances of the solvent ($CDCl_3$: δ 77.00, CD_3OD: δ 49.00, DMSO-d_6: δ 39.51). Infrared spectra were recorded on NaCl disc (for liquids) on a Nicolet IR/42 spectrometer or on a JASCO FT/IR-6600. Samples were prepared as KBr pellets. High Resolution Mass Spectrometry was performed in the Department of Mass Spectrometry & Metabolomics Core Facility at Michigan State University Mass Facility. HPLC analyses were performed using Agilent 1100 or 1260 HPLC system with Diacel Chiralpak IA and Chiralpak OD-H and AD-H columns. HPLC grade hexanes (mixture of isomers) and 2-propanol were used for HPLC analyses. Optical rotations were obtained at a wavelength of 589 nm (sodium D line) using a 1.0 decimeter cell with a total volume of 1.0 mL. Specific rotations are reported in degrees per decimeter at 20 °C and the concentrations are given in gram per 100 mL in ethyl acetate unless otherwise noted.

2. Synthesis of Bisallylic Amide Substrates

General procedure A:

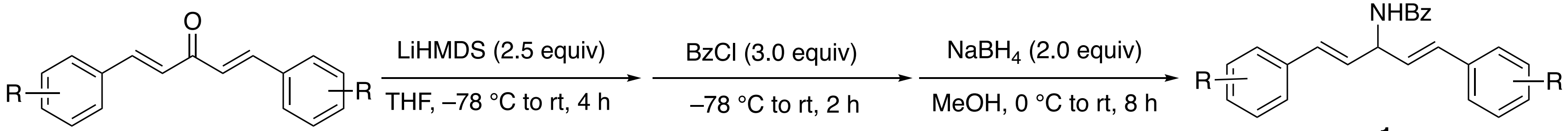

S1 **1**

i. Dienone **S1** (synthesized by modifications of the known procedure)[2, 3] (10.0 mmol, 1.00 equiv.) was dissolved in dry THF (10 mL) yielding a 1M solution in a 100 mL round-bottom flask. The solution was then cooled to –78 ºC in a dry ice bath. After 10 min, lithium bis(trimethylsilyl)amide solution (25.0 mL, 1 M in tetrahydrofuran, 25.0 mmol, 2.5 equiv.) was added dropwise to this solution. The reaction mixture was allowed to warm to room temperature where it was stirred for 4 h. The solution was cooled to –78 ºC in a dry ice bath. After 10 min, benzoyl chloride (3.50 mL, 30.0 mmol, 3.0 equiv.) was added to the solution in one portion. The reaction mixture was warmed to room temperature and stirred for 2 h. The reaction mixture was then quenched with saturated aqueous ammonium chloride solution (5 mL). The aqueous layer was extracted with ethyl acetate (3 × 10 mL). The combined organic layers were dried over anhydrous sodium sulfate, filtered and concentrated under reduced pressure to provide crude benzoylimine, which was used directly in the next step without further purification.

ii. The benzoylimine intermediate was dissolved in methanol (10 mL) in a 100 mL round-bottom flask. The solution was cooled to 0 ºC in an ice-water bath. After 5 min, sodium borohydride (757 mg, 20.0 mmol, 2.0 equiv.) was added in one portion to the solution. The reaction mixture was allowed to warm to room temperature and stirred for 8 h. The reaction mixture was quenched by the addition of water (5 mL). The aqueous layer was extracted with ethyl acetate (3 × 10 mL). The combined organic layers were dried over anhydrous sodium sulfate,

filtered and concentrated under reduced pressure to provide the crude product. The crude product was purified by flash column chromatography on silica gel (eluent: ethyl acetate/ hexanes) to furnish the pure bisallylic amide product **1**.

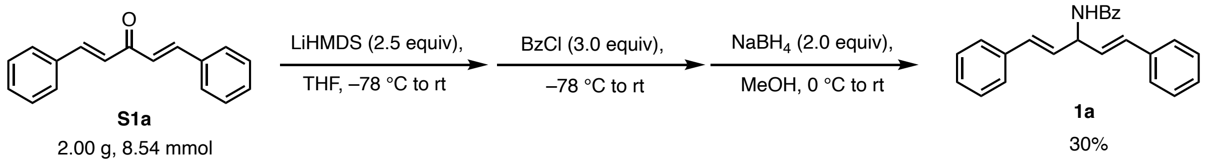

<u>*N*-((1*E*,4*E*)-1,5-diphenylpenta-1,4-dien-3-yl)benzamide **1a**:</u>

Bisallylic amide **1a** was prepared from dienone **S1a** (2.00 g, 8.54 mmol, 1.00 equiv.) using General Procedure A. The crude product was purified by flash column chromatography on silica gel (eluent: 20% ethyl acetate in hexanes) to give **1a** (0.87 g, mp: 203-205 ºC, 30%) as a white snowflake solid.

TLC (30% ethyl acetate in hexanes), R*f*: 0.54 (UV).

^{1}H NMR (500 MHz, $CDCl_3$): δ 7.86 (d, *J* = 7.8 Hz, 2H), 7.57 – 7.49 (m, 1H), 7.46 (d, J = 7.5 Hz, 2H), 7.40 (d, *J* = 7.0 Hz, 4H), 7.33 (t, *J* = 7.5 Hz, 4H), 7.26 (td, *J* = 7.3, 1.3 Hz, 2H), 6.67 (d, *J* = 16.0 Hz, 2H), 6.45 (d, *J* = 8.4 Hz, 1H), 6.36 (dd, *J* = 16.0, 6.1 Hz, 2H), 5.70 – 5.56 (m, 1H). ^{13}C NMR (126 MHz, $CDCl_3$): δ 166.5, 136.4, 134.4, 131.8, 131.7, 128.7, 128.6, 128.0, 127.9, 127.0, 126.5, 52.8. HRMS (ESI) (m/z): calculated for $(M+H)^+$: $C_{24}H_{21}ClNO$ 374.1312; found: 374.1346. These spectral data match those previously reported for this compound.[4]

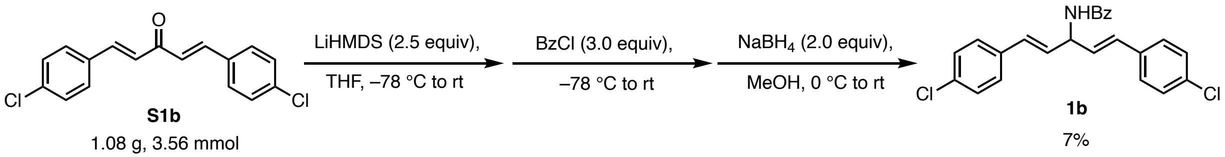

<u>*N*-((1*E*,4*E*)-1,5-bis(4-chlorophenyl)penta-1,4-dien-3-yl)benzamide **1b**:</u>

Bisallylic amide **1b** was prepared from dienone **S1b** (1.08 g, 3.56 mmol, 1.00 equiv.) by General Procedure A. The crude product was purified by flash column chromatography on silica gel (eluent: 20% ethyl acetate in hexanes) to give **1b** (0.10 g, mp: 156-158 ºC, 7%) as a white solid. TLC (15% ethyl acetate in hexanes), R*f*: 0.33 (UV).

^{1}H NMR (500 MHz, $CDCl_3$): δ 7.83 (d, *J* = 7.0 Hz, 1H), 7.54 – 7.49 (m, 1H), 7.47 – 7.40 (m, 2H), 7.32 – 7.24 (m, 9H), 6.59 (dd, *J* = 15.9, 1.5 Hz, 2H), 6.38 (d, *J* = 8.3 Hz, 1H), 6.28 (dd, *J* = 15.9, 6.1 Hz, 2H), 5.58 (dddd, *J* = 8.0, 6.2, 4.6, 1.5 Hz, 1H). ^{13}C NMR (126 MHz, $CDCl_3$): δ 166.5, 134.8, 134.1, 133.6, 131.8, 130.8, 128.8, 128.7, 128.4, 127.7, 127.0, 52.8. HRMS (ESI) (m/z): calculated for $(M+H)^+$: $C_{24}H_{20}Cl_2NO$ 408.0922; found: 408.0918.

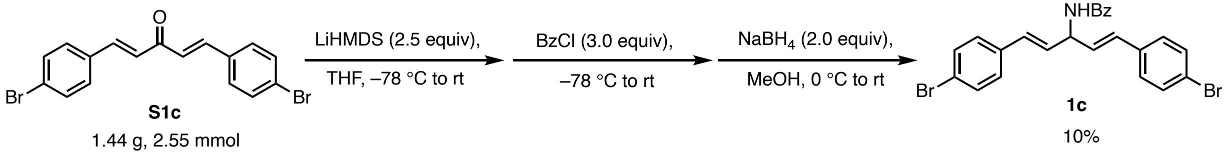

N-((1*E*,4*E*)-1,5-bis(4-bromophenyl)penta-1,4-dien-3-yl)benzamide **1c**:

Bisallylic amide **1c** was prepared from dienone **S1c** (1.44 g, 2.55 mmol, 1.00 equiv.) by General Procedure A. The crude product was purified by flash column chromatography on silica gel (eluent: 20% ethyl acetate in hexanes) to give **1c** (0.35 g, mp 180-186 ºC, 10%) as a white solid. TLC (15% ethyl acetate in hexanes), R*f*: 0.13 (UV).

^{1}H NMR (500 MHz, $CDCl_3$): δ 7.85 – 7.77 (m, 2H), 7.56 – 7.47 (m, 1H), 7.47 – 7.36 (m, 6H), 7.27 – 7.18 (m, 4H), 6.58 (d, *J* = 16.0 Hz, 2H), 6.30 (dd, *J*= 15.8, 6.3 Hz, 3H), 5.57 (q, *J* = 6.8 Hz, 1H). ^{13}C NMR (126 MHz, $CDCl_3$): δ 166.5, 135.2, 134.1, 131.8, 131.7, 130.9, 128.7, 128.5, 128.1, 127.0, 121.8, 52.8. HRMS (ESI) (m/z): calculated for $(M-H)^-$: $C_{24}H_{18}NOBr_2$ 493.9755; found: 493.9752. These spectral data match those previously reported for this compound.[4]

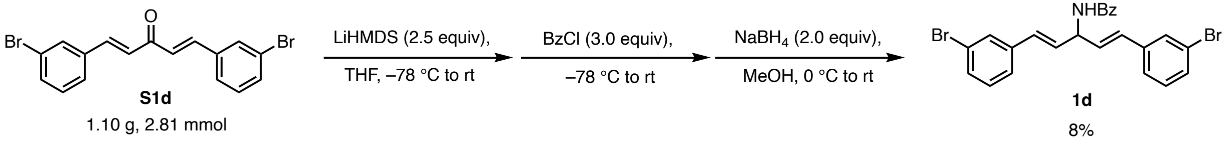

N-((1*E*,4*E*)-1,5-bis(3-bromophenyl)penta-1,4-dien-3-yl)benzamide **1d**:

Bisallylic amide **1d** was prepared from dienone **S1d** (1.10 g, 2.81 mmol, 1.00 equiv.) by General Procedure A. The crude product was purified by flash column chromatography on silica gel (eluent: 20% ethyl acetate in hexanes) to give **1d** (0.11 g, mp 120-125 ºC, 8%) as a white solid. TLC (15% ethyl acetate in hexanes), R*f*: 0.20 (UV).

^{1}H NMR (500 MHz, $CDCl_3$): δ 7.83 (d, J = 7.5 Hz, 2H), 7.57 – 7.48 (m, 3H), 7.44 (t, J = 7.6 Hz, 2H), 7.36 (dd, J = 7.8, 1.9 Hz, 2H), 7.28 (d, J = 7.8 Hz, 2H), 7.17 (t, J = 7.8 Hz, 2H), 6.57 (d, J = 15.9 Hz, 2H), 6.37 (d, J = 8.3 Hz, 1H), 6.31 (dd, J = 15.9, 6.1 Hz, 2H), 5.60 (q, J = 6.8 Hz, 1H). ^{13}C NMR (126 MHz, $CDCl_3$): δ 166.5, 138.4, 134.1, 131.8, 130.8, 130.6, 130.1, 129.3, 129.3, 128.7, 127.0, 125.3, 122.8, 52.7. HRMS (ESI) (m/z): calculated for $(M-H)^-$: $C_{24}H_{18}NOBr_2$ 493.9755; found: 493.9740.

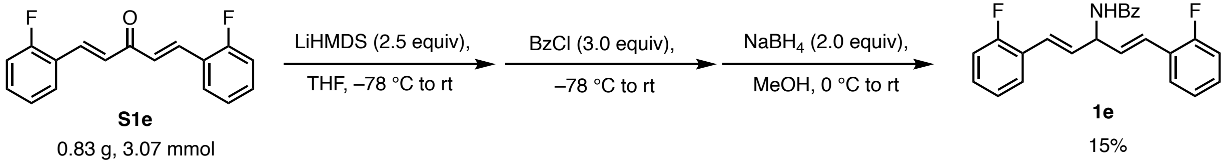

N-((1*E*,4*E*)-1,5-bis(2-fluorophenyl)penta-1,4-dien-3-yl)benzamide **1e**:

Bisallylic amide **1e** was prepared from dienone **S1e** (0.83 g, 3.1 mmol, 1.0 equiv) using General Procedure A. The crude product was purified by flash column chromatography on silica gel (eluent: 20% ethyl acetate in hexanes) to give **1e** (0.17 g, mp 155-158 ºC, 15%) as a white solid.

TLC (30% ethyl acetate in hexanes), R*f*: 0.59 (UV).

^{1}H NMR (500 MHz, $CDCl_3$): δ 7.87 – 7.78 (m, 2H), 7.54 – 7.48 (m, 1H), 7.45 (ddd, *J* = 8.9, 7.1, 1.6 Hz, 4H), 7.24 – 7.16 (m, 2H), 7.14 – 7.04 (m, 3H), 6.80 (dd, *J* = 16.1, 1.4 Hz, 2H), 6.45 (dd, *J* = 16.1, 6.0 Hz, 2H), 6.33 (d, *J* = 8.3 Hz, 1H), 5.68 – 5.59 (m, 1H). ^{13}C NMR (126 MHz, $CDCl_3$): δ 166.6, δ 160.3 (d, *J* = 249.8 Hz), 134.3, 131.7, 130.6 (d, *J* = 5.3 Hz), 129.2 (d, *J* = 8.3 Hz), 128.7, 127.8 (d, *J* = 3.6 Hz), 127.1, 124.5 (d, *J* = 3.2 Hz), 124.3, 124.2 (d, *J* = 3.4 Hz), 115.8 (d, *J* = 22.1 Hz), 53.4. HRMS (ESI) (m/z): calculated for $(M+Na)^+$: $C_{24}H_{19}F_2NONa$ 398.1332; found: 398.1336.

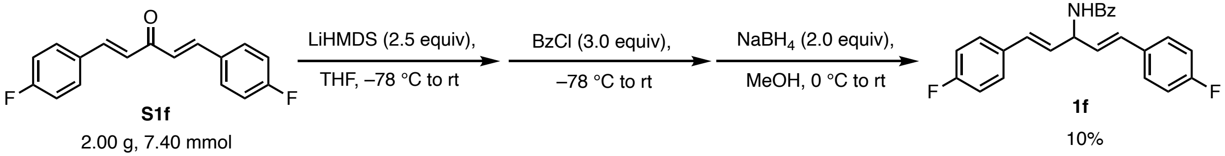

<u>*N*-((1*E*,4*E*)-1,5-bis(4-fluorophenyl)penta-1,4-dien-3-yl)benzamide **1f**:</u>

Bisallylic amide **1f** was prepared from dienone **S1f** (2.00 g, 7.40 mmol, 1.00 equiv.) using General Procedure A. The crude product was purified by flash column chromatography on silica gel (eluent: 20% ethyl acetate in hexanes) to give **1f** (0.28 g, mp 148-152 ºC, 10%) as a white solid.

TLC (15% ethyl acetate in hexanes), R*f*: 0.12 (UV).

^{1}H NMR (500 MHz, $CDCl_3$): δ 7.83 – 7.81 (m, 2H), 7.56 – 7.41 (m, 3H), 7.36 (ddd, *J* = 8.5, 5.1, 2.2 Hz, 4H), 7.00 (td, *J* = 8.7, 2.2 Hz, 4H), 6.62 (d, *J* = 15.9 Hz, 2H), 6.28 (s, 1H), 6.24 (ddd, *J* = 15.9, 6.2, 2.2 Hz, 2H), 5.58 (d, *J* = 7.4 Hz, 1H). ^{13}C NMR (126 MHz, $CDCl_3$): δ 166.4, 162.5 (d, *J* = 247.4 Hz), 134.2, 132.5 (d, *J* = 3.4 Hz), 131.8, 130.8, 128.7, 128.1 (d, *J* = 8.1 Hz), 127.7 (d, *J* = 2.2 Hz), 127.0, 115.6 (d, *J* = 21.6 Hz), 52.8. HRMS (ESI) (m/z): calculated for $(M+H)^+$: $C_{24}H_{20}NOF_2$ 376.1513; found: 376.1507.

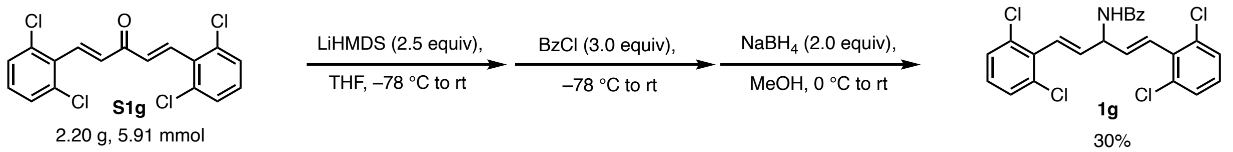

N-((1E,4E)-1,5-bis(2,6-dichlorophenyl)penta-1,4-dien-3-yl)benzamide **1g**:

Bisallylic amide **1g** was prepared from dienone **S1g** (2.20 g, 5.91 mmol, 1.00 equiv.) using General Procedure A. The crude product was purified by flash column chromatography on silica gel (eluent: 20% ethyl acetate in hexanes) to give **1g** (0.84 g, mp 145-150 ºC, 30%) as a white solid.

TLC (30% ethyl acetate in hexanes), R*f*: 0.54 (UV).

^{1}H NMR (500 MHz, $CDCl_3$): δ 7.83 (dd, *J* = 7.5, 1.6 Hz, 2H), 7.56 – 7.39 (m, 3H), 7.30 (d, *J* = 8.1 Hz, 4H), 7.09 (t, *J* = 8.1 Hz, 2H), 6.76 (dd, *J* = 16.4, 1.5 Hz, 2H), 6.43 (dd, *J* = 16.3, 5.6 Hz, 2H), 6.32 (d, *J* = 8.5 Hz, 1H), 5.82 – 5.71 (m, 1H). ^{13}C NMR (126 MHz, $CDCl_3$): δ 166.7, 136.0, 134.4, 134.0, 131.7, 128.7, 128.4, 128.4, 127.0, 126.0, 125.3, 52.9. HRMS (ESI) (m/z): calculated for $(M\text{-}H)^-$: $C_{24}H_{16}NOCl_4$ 473.9986; found: 473.9980.

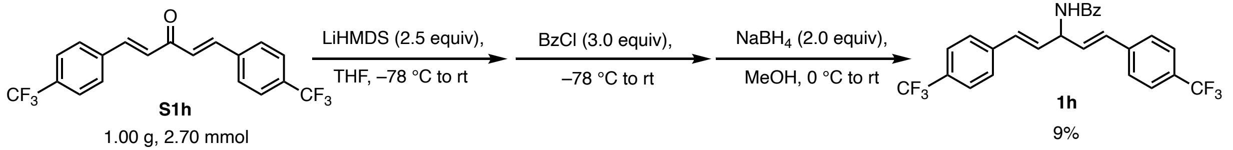

N-((1E,4E)-1,5-bis(4-(trifluoromethyl)phenyl)penta-1,4-dien-3-yl)benzamide **1h**:

Bisallylic amide **1h** was prepared from dienone **S1h** (1.00 g, 2.70 mmol, 1.00 equiv.) by General Procedure A. The crude product was purified by flash column chromatography on silica gel (eluent: 20% ethyl acetate in hexanes) to give **1h** (0.11 g, mp 156-162 ºC, 9%) as a white solid.

TLC (15% ethyl acetate in hexanes), R*f*: 0.14 (UV).

^{1}H NMR (500 MHz, $CDCl_3$): δ 7.84 (d, *J* = 7.0 Hz, 2H), 7.57 – 7.50 (m, 5H), 7.49 – 7.42 (m, 6H), 6.71 – 6.67 (m, 2H), 6.43 (dd, *J* = 16.0, 6.1 Hz, 2H), 6.38 (d, *J* = 8.3 Hz, 1H), 5.68 – 5.63 (m, 1H). ^{13}C NMR (126 MHz, $CDCl_3$): δ 166.6, 139.7, 134.0, 131.9, 130.9, 130.2, 130.0, 129.7, 128.8, 127.0, 126.7, 125.7, 125.6, 125.6, 125.6, 52.7. HRMS (ESI) (m/z): calculated for $(M+H)^+$: $C_{26}H_{20}NOF_6$ 476.1449; found: 476.1451.

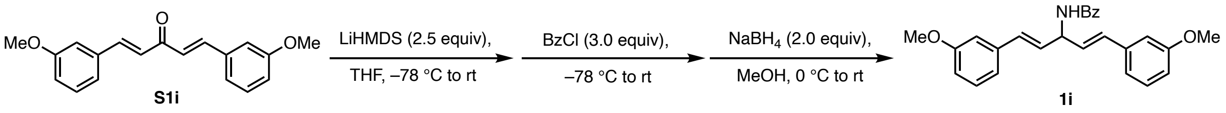

S1i
1.89 g, 6.42 mmol

1i
23%

<u>*N*-((1*E*,4*E*)-1,5-bis(3-methoxyphenyl)penta-1,4-dien-3-yl)benzamide **1i**:</u>

Bisallylic amide **1i** was prepared from dienone **S1i** (1.89 g, 6.42 mmol, 1.00 equiv.) by General Procedure A. The crude product was purified by flash column chromatography on silica gel (eluent: 20% ethyl acetate in hexanes) to give **1i** (0.59 g, mp 110-115 ºC, 23%) as a white solid. TLC (15% ethyl acetate in hexanes), R*f*: 0.17 (UV).

^{1}H NMR (500 MHz, $CDCl_3$): δ 7.85 – 7.79 (m, 2H), 7.53 – 7.48 (m, 1H), 7.47 – 7.41 (m, 2H), 7.24 – 7.18 (m, 2H), 6.98 (dt, *J* = 7.8, 1.2 Hz, 2H), 6.92 (dd, *J* = 2.6, 1.6 Hz, 2H), 6.80 (ddd, *J* = 8.2, 2.6, 0.9 Hz, 2H), 6.63 (dd, *J* = 15.9, 1.4 Hz, 2H), 6.33 (dd, *J* = 16.0, 6.1 Hz, 3H), 5.65 – 5.56 (m, 1H), 3.79 (s, 6H). ^{13}C NMR (126 MHz, $CDCl_3$): δ 166.4, 159.8, 137.8, 134.3, 131.8, 131.7, 129.6, 128.7, 128.3, 127.0, 119.2, 113.7, 111.7, 110.0, 55.3, 52.7. HRMS (ESI) (m/z): calculated for $(M+Na)^+$: $C_{26}H_{25}NO_3Na$ 422.1732; found: 422.1734. These spectral data match those previously reported for this compound.[4]

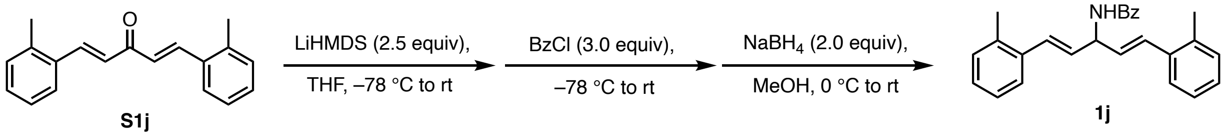

S1j
1.30 g, 5.00 mmol

1j
19%

<u>*N*-((1*E*,4*E*)-1,5-di-*o*-tolylpenta-1,4-dien-3-yl)benzamide **1j**:</u>

Bisallylic amide **1j** was prepared from dienone **S1j** (1.30 g, 5.00 mmol, 1.00 equiv.) by General Procedure A. The crude product was purified by flash column chromatography on silica gel (eluent: 20% ethyl acetate in hexanes) to give **1j** (0.35 g, mp 145-150 ºC, 19%) as a white solid. TLC (15% ethyl acetate in hexanes), R*f*: 0.22 (UV).

^{1}H NMR (500 MHz, $CDCl_3$): δ 7.82 (d, *J* = 6.9 Hz, 2H), 7.52 – 7.49 (m, 1H), 7.46 – 7.43 (m, 4H), 7.15 (ddd, *J* = 8.6, 5.2, 3.7 Hz, 6H), 6.89 (d, *J* = 1.5 Hz, 2H), 6.31 (d, *J* = 8.3 Hz, 1H), 6.21 (dd, *J* = 15.8, 6.1 Hz, 2H), 5.67 – 5.62 (m, 1H), 2.33 (s, 6H). ^{13}C NMR (126 MHz, $CDCl_3$): δ 166.5, 135.6, 134.5, 131.6, 130.3, 129.8, 129.5, 128.7, 127.8, 127.0, 126.1, 125.7, 53.4, 19.9. HRMS (ESI) (m/z): calculated for $(M+H)^+$: $C_{26}H_{26}NO$ 368.2016; found: 368.2014.

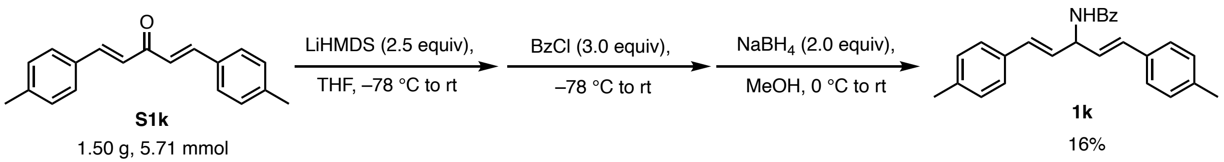

<u>*N*-((1*E*,4*E*)-1,5-di-*p*-tolylpenta-1,4-dien-3-yl)benzamide **1k**:</u>

Bisallylic amide **1k** was prepared from dienone **S1k** (1.50 g, 5.71 mmol, 1.00 equiv.) by General Procedure A. The crude product was purified by flash column chromatography on silica gel (eluent: 20% ethyl acetate in hexanes) to give **1k** (0.34 g, mp 165-175 ºC, 16%) as a white solid. TLC (15% ethyl acetate in hexanes), R*f*: 0.14 (UV).

^{1}H NMR (500 MHz, $CDCl_3$): δ 7.83 (dt, *J* = 7.2, 1.4 Hz, 2H), 7.54 – 7.40 (m, 3H), 7.28 (d, *J* = 7.8 Hz, 4H), 7.11 (d, *J* = 7.8 Hz, 4H), 6.61 (d, *J* = 15.8 Hz, 2H), 6.36 – 6.24 (m, 3H), 5.59 (q, *J* = 7.5, 6.8 Hz, 1H), 2.32 (s, 6H). ^{13}C NMR (126 MHz, $CDCl_3$): δ 166.4, 137.7, 134.5, 133.7, 131.6, 129.3, 128.6, 127.1, 127.0, 126.5, 126.4, 52.8, 21.2. HRMS (ESI) (m/z): calculated for $(M+H)^+$: $C_{26}H_{26}NO$ 368.2016; found: 368.2010.

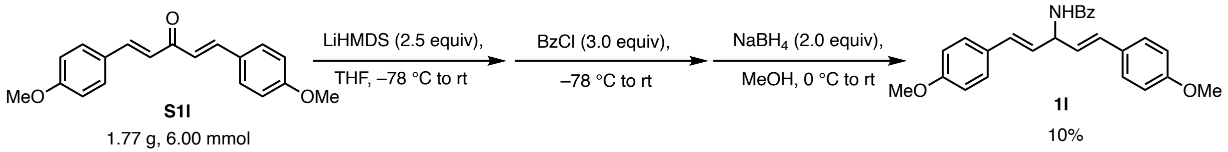

<u>*N*-((1*E*,4*E*)-1,5-bis(4-methoxyphenyl)penta-1,4-dien-3-yl)benzamide **1l**:</u>

Bisallylic amide **1l** was prepared from dienone **S1l** (1.77 g, 6.00 mmol, 1.00 equiv.) by General Procedure A. The crude product was purified by flash column chromatography on silica gel (eluent: 30% ethyl acetate in hexanes) to give **1l** (0.24 g, mp 157-161 ºC, 10%) as a white solid. TLC (30% ethyl acetate in hexanes), R*f*: 0.25 (UV).

^{1}H NMR (500 MHz, $CDCl_3$): δ 7.85 – 7.78 (m, 2H), 7.54 – 7.39 (m, 3H), 7.35 – 7.25 (m, 4H), 6.91 – 6.77 (m, 4H), 6.59 (dd, *J* = 15.9, 1.4 Hz, 2H), 6.29 (d, *J* = 8.3 Hz, 1H), 6.19 (dd, J = 15.9, 6.1 Hz, 2H), 5.61 – 5.49 (m, 1H), 3.79 (s, 6H). ^{13}C NMR (126 MHz, $CDCl_3$): δ 166.4, 159.4, 134.5, 131.6, 131.1, 129.2, 128.6, 127.7, 127.0, 126.0, 114.0, 55.3, 52.9. HRMS (ESI) (m/z): calculated for $(M+H)^+$: $C_{26}H_{26}NO_3$ 400.1913; found: 400.1908.

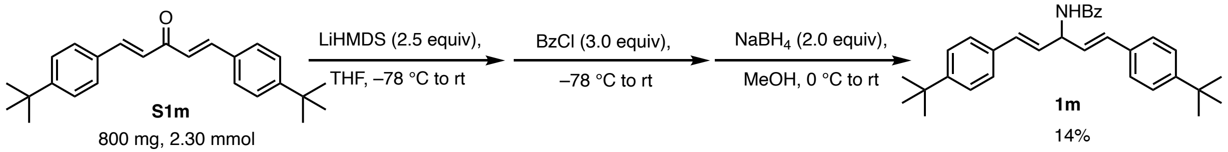

<u>*N*-((1*E*,4*E*)-1,5-bis(4-(*tert*-butyl)phenyl)penta-1,4-dien-3-yl)benzamide **1m**:</u>

Bisallylic amide **1m** was prepared from dienone **S1m** (800 mg, 2.30 mmol, 1.00 equiv.) using General Procedure A. The crude product was purified by flash column chromatography on silica gel (eluent: 15% ethyl acetate in hexanes) to give **1m** (0.15 g, 14%) as a colorless oil. TLC (15% ethyl acetate in hexanes), R*f*: 0.28 (UV).

^{1}H NMR (500 MHz, $CDCl_3$): δ 7.84 – 7.78 (m, 2H), 7.54 – 7.37 (m, 3H), 7.33 (s, 8H), 6.63 (dd, J = 15.9, 1.5 Hz, 2H), 6.34 – 6.24 (m, 3H), 5.63 – 5.57 (m, 1H), 1.29 (s, 18H). ^{13}C NMR (126 MHz, $CDCl_3$): δ 166.4, 151.0, 134.5, 133.6, 131.6, 131.5, 128.6, 127.3, 126.9, 126.2, 125.5, 52.7, 34.6, 31.3. HRMS (ESI) (m/z): calculated for $(M+Na)^+$: $C_{32}H_{37}NONa$ 474.2773; found: 474.2778.

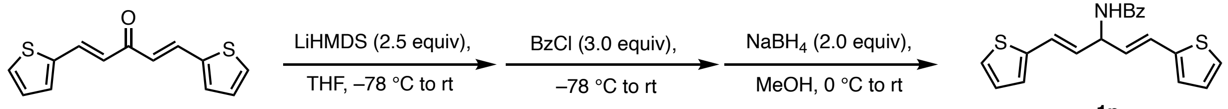

S1n
2.2 g, 8.93 mmol

1n
10%

N-((1*E*,4*E*)-1,5-di(thiophen-2-yl)penta-1,4-dien-3-yl)benzamide **1n**:

Bisallylic amide **1n** was prepared from dienone **S1n** (2.20 g, 8.93 mmol, 1.00 equiv.) using General Procedure A. The crude product was purified by flash column chromatography on silica gel (eluent: 20% ethyl acetate in hexanes) to give **1n** (0.31 g, mp 160-163 ºC, 10%) as a yellow solid.

TLC (30% ethyl acetate in hexanes), R*f*: 0.35 (UV).

^{1}H NMR (500 MHz, $CDCl_3$): δ 7.89 – 7.73 (m, 2H), 7.58 – 7.33 (m, 3H), 7.16 (dt, J =4.8, 0.9 Hz, 2H), 7.02 – 6.85 (m, 4H), 6.86 – 6.67 (m, 2H), 6.27 (d, J = 8.3 Hz, 1H), 6.13 (dd, J = 15.8, 6.1 Hz, 2H), 5.67 – 5.44 (m, 1H). ^{13}C NMR (126 MHz, $CDCl_3$): δ 166.4, 141.4, 134.2, 131.7, 128.7, 127.5, 127.2, 127.0, 126.4, 125.3, 124.6, 52.5. HRMS (ESI) (m/z): calculated for $(M-H)^-$: $C_{20}H_{16}NOS_2$ 350.0673; found: 350.0670. These spectral data match those previously reported for this compound.[4]

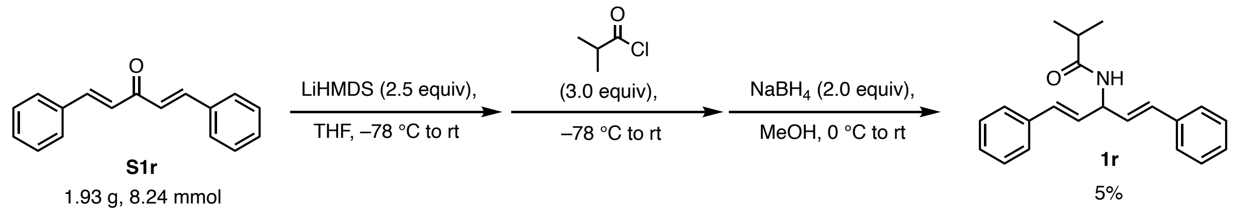

<u>*N*-((1*E*,4*E*)-1,5-diphenylpenta-1,4-dien-3-yl)isobutyramide **1r**:</u>

Bisallylic amide **1r** was prepared from dienone **S1r** (1.93 g, 8.24 mmol, 1.00 equiv.) and isobutyryl chloride (2.63 g, 24.7 mmol, 3.00 equiv.) using General Procedure A. The crude product was purified by flash column chromatography on silica gel (eluent: 20% ethyl acetate in hexanes) to give **1r** (0.12 g, mp 172-176 ºC, 5%) as a white solid.

TLC (30% ethyl acetate in hexanes), R*f*: 0.43 (UV).

^{1}H NMR (500 MHz, $CDCl_3$): δ 7.40 – 7.35 (m, 4H), 7.31 (dd, *J* = 8.5, 6.8 Hz, 4H), 7.27 – 7.21 (m, 2H), 6.57 (dd, *J* = 16.0, 1.4 Hz, 2H), 6.25 (dd, *J* = 16.0, 6.0 Hz, 2H), 5.69 (d, *J* = 8.5 Hz, 1H), 5.51 – 5.35 (m, 1H), 2.43 (hept, *J* = 6.9 Hz, 1H), 1.21 (d, *J* = 6.9 Hz, 6H). ^{13}C NMR (126 MHz, $CDCl_3$): δ 175.9, 136.5, 131.4, 131.4, 128.6, 128.3, 127.8, 126.5, 51.9, 35.9, 19.7. HRMS (ESI) (m/z): calculated for $(M-H)^-$: $C_{21}H_{22}NO$ 304.1701; found: 304.1695.

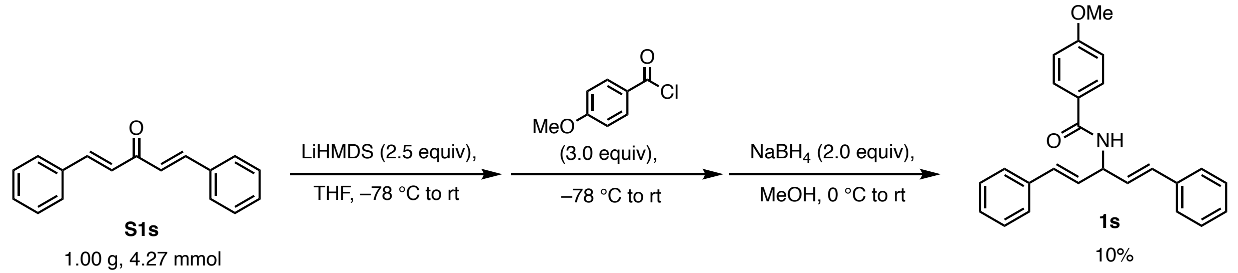

<u>*N*-((1*E*,4*E*)-1,5-diphenylpenta-1,4-dien-3-yl)-4-methoxybenzamide **1s**:</u>

Bisallylic amide **1s** was prepared from dienone **S1s** (1.00 g, 4.27 mmol, 1.00 equiv.) and 4-methoxybenzoyl chloride (2.19 g, 12.8 mmol, 3.00 equiv.) using General Procedure A. The crude product was purified by flash column chromatography on silica gel (eluent: 20% ethyl acetate in hexanes) to give **1s** (0.15 g, mp 188-193 °C, 10%) as a white solid.

TLC (30% ethyl acetate in hexanes), R*f*: 0.39 (UV).

^{1}H NMR (500 MHz, $CDCl_3$): δ 7.80 (d, *J* = 8.8 Hz, 2H), 7.41 – 7.35 (m, 4H), 7.34 – 7.26 (m, 4H), 7.27 – 7.21 (m, 2H), 6.92 (d, *J* = 8.8 Hz, 2H), 6.64 (dd, *J* = 16.0, 1.4 Hz, 2H), 6.38 – 6.26 (m, 3H), 5.65 – 5.56 (m, 1H), 3.83 (s, 3H). ^{13}C NMR (126 MHz, $CDCl_3$): δ 165.9, 162.3, 136.4, 131.7, 128.8, 128.6, 128.2, 127.8, 126.6, 126.5, 113.8, 55.4, 52.7. HRMS (ESI) (m/z): calculated for $(M+H)^+$: $C_{25}H_{24}NO_2$ 370.1809; found: 370.1812.

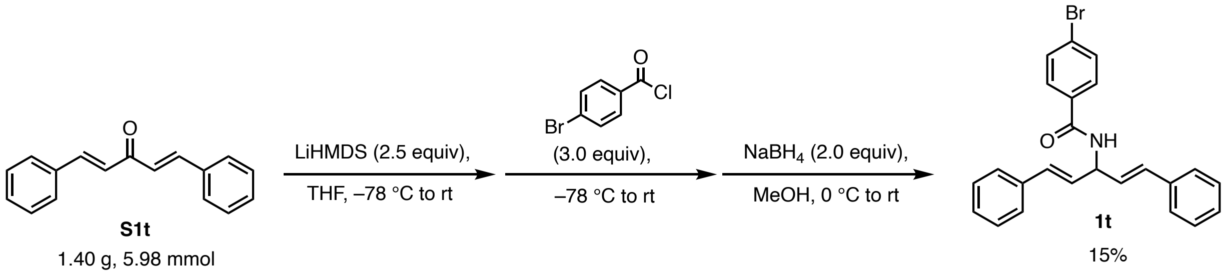

4-bromo-*N*-((1*E*,4*E*)-1,5-diphenylpenta-1,4-dien-3-yl)benzamide **1t**:

Bisallylic amide **1t** was prepared from dienone **S1t** (1.40 g, 5.98 mmol, 1.00 equiv.) and 4-bromobenzoyl chloride (3.94 g, 17.9 mmol, 3.00 equiv.) by General Procedure A. The crude product was purified by flash column chromatography on silica gel (eluent: 10% ethyl acetate in hexanes) to give **1t** (0.37 g, mp 215-220 ºC, 15%) as a white solid.

TLC (30% ethyl acetate in hexanes), R*f*: 0.65 (UV).

^{1}H NMR (500 MHz, $CDCl_3$): δ 7.69 (d, *J* = 8.6 Hz, 2H), 7.58 (d, *J* = 8.6 Hz, 2H), 7.42 – 7.35 (m, 4H), 7.35 – 7.27 (m, 4H), 7.25 (d, *J* = 7.6 Hz, 2H), 6.65 (dd, *J* = 16.0, 1.4 Hz, 2H), 6.32 (dd, *J* = 15.9, 6.1 Hz, 2H), 6.27 (d, *J* = 8.2 Hz, 1H), 5.63 – 5.54 (m, 1H). ^{13}C NMR (126 MHz, $CDCl_3$):

δ 165.5, 136.3, 133.2, 132.1, 131.9, 128.6, 128.6, 127.9, 127.7, 126.5, 126.4, 52.9. HRMS (ESI) (m/z): calculated for $(M+H)^+$: $C_{24}H_{21}BrNO$ 418.0807; found: 418.0791.

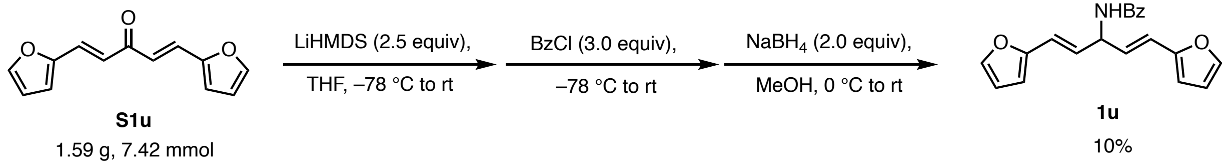

N-((1*E*,4*E*)-1,5-di(furan-2-yl)penta-1,4-dien-3-yl)benzamide **1u**:

Bisallylic amide **1u** was prepared from dienone **S1u** (1.59 g, 7.42 mmol, 1.00 equiv.) by General Procedure A. The crude product was purified by flash column chromatography on silica gel (eluent: 20% ethyl acetate in hexanes) to give **1u** (0.84 g, mp 135-142 ºC, 10%) as a yellow solid.

TLC (30% ethyl acetate in hexanes), R*f*: 0.45 (UV).

^{1}H NMR (500 MHz, $CDCl_3$): δ 7.81 (dd, *J* = 7.7, 1.6 Hz, 2H), 7.55 – 7.45 (m, 1H), 7.47 – 7.27 (m, 5H), 6.55 – 6.43 (m, 4H), 6.36 (d, *J* = 8.3 Hz, 1H), 6.02 (dd, *J* = 15.8, 6.0 Hz, 2H), 5.49 (dt, *J* = 7.8, 5.9 Hz, 1H). ^{13}C NMR (126 MHz, $CDCl_3$): δ 166.4, 143.7, 140.9, 134.3, 131.7, 128.6, 127.6, 127.0, 123.4, 121.6, 107.4, 107.4, 52.7. HRMS (ESI) (m/z): calculated for $(M-H)^-$: $C_{20}H_{16}NO_3$ 318.1130; found: 318.1118.

General procedure B:

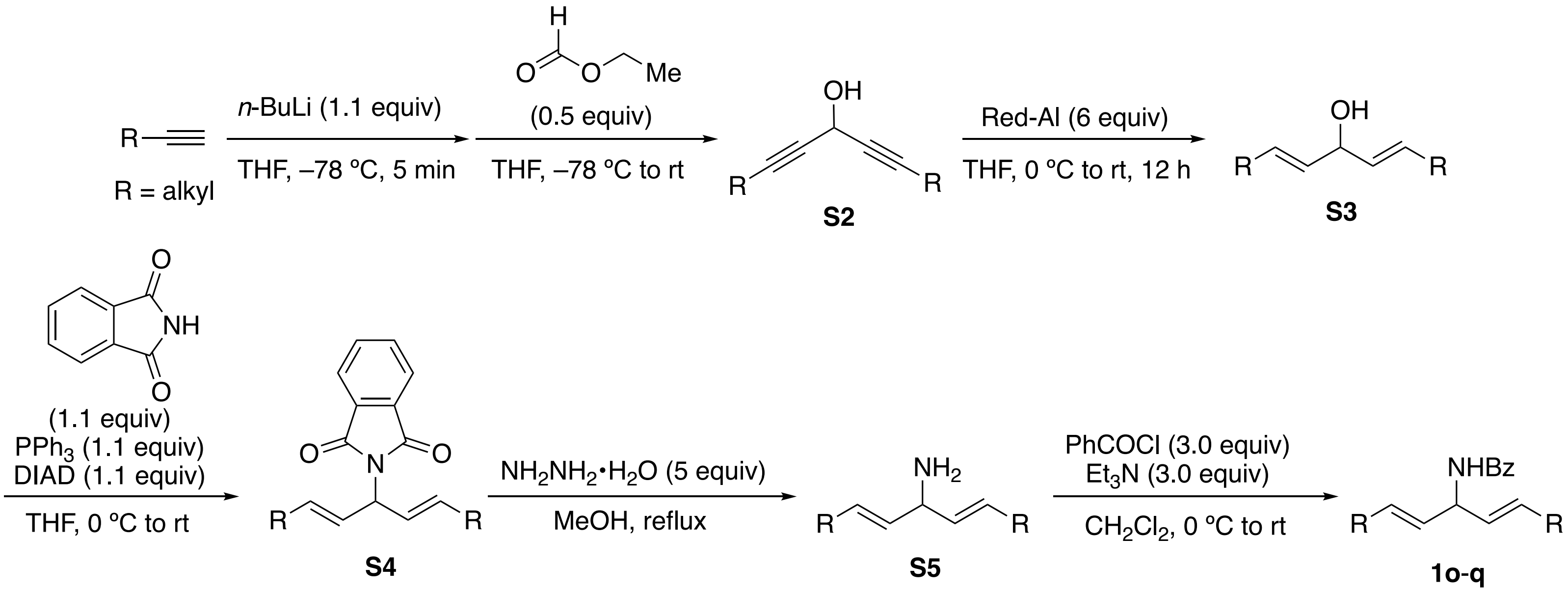

i. Alkylacetylene (13.9 mmol, 1 equiv.) was dissolved in anhydrous tetrahydrofuran (20 mL) in a 100 mL round bottom flask. The homogeneous solution was cooled to –78 °C using a dry ice bath. After 10 min, *n*-butyllithium solution (2.5 M in hexanes, 1.1 equiv.) was added dropwise to the reaction mixture. After stirring for 5 min, ethyl formate (0.50 equiv.) was added to the round bottom in one portion. The reaction mixture was stirred at the same temperature for another 30 min and then warmed up to room temperature. The completion of the reaction was monitored by the disappearance of the starting material. The reaction mixture was quenched by adding saturated aqueous ammonium chloride solution (20 mL), the layers were separated, and the organic layer was extracted with dichloromethane (3 × 10 mL). The combined organic layers were combined and dried over anhydrous sodium sulfate, filtered and concentrated under reduced pressure to

provide crude product. The crude reaction mixture was purified by flash column chromatography on silica gel to provide propargylic alcohol **S2**. [This experimental procedure has been modified from a known procedure].[5]

ii. Propargyl alcohol **S2** (6.9 mmol, 1 equiv.) was dissolved in anhydrous tetrahydrofuran (50 mL) in a 250 mL round bottom. The solution was cooled to 0 ºC in an ice-water bath. After stirring for 5 min at this temperature, Red-Al (70% in toluene, 6.0 equiv.) was added to the solution dropwise. The reaction mixture was allowed to warm to room temperature and stirred for 12 h. The reaction mixture was quenched by adding saturated aqueous Rochelle salt solution (10 mL) and stirred for 1 h until two clear layers were seen. The aqueous layer was extracted with ethyl acetate (3 × 20 mL). The combined organic layers were washed with brine (20 mL) and dried over anhydrous sodium sulfate, filtered and concentrated under reduced pressure to provide the crude bis allylic alcohol. The crude alcohol was purified by flash column chromatography on silica gel (eluent: ethyl acetate/ hexanes) to provide bisallylic alcohol **S3**. [This experimental procedure has been modified from a known procedure].[6]

iii. Bisallylic alcohol **S3** (4.67 mmol, 1 equiv.) was dissolved in anhydrous tetrahydrofuran (25 mL) and the reaction mixture was subsequently cooled to 0 °C in an ice-bath. After stirring at this temperature for 5 min, triphenylphosphine (1.1 equiv.), phthalimide (1.1 equiv.) and diisopropyl azodicarboxylate (1.1 equiv.) were added sequentially. The reaction mixture was allowed to warm to room temperature over a period of 30 min where it was stirred for an additional hour. The reaction mixture was quenched by adding water (20 mL) and the aqueous layer was extracted with ethyl acetate (3 × 10 mL). The combined organic layers were washed with brine (20 mL), dried over anhydrous sodium sulfate, filtered and concentrated under reduced pressure to provide crude allylic imide. The crude imide was purified by flash column chromatography on silica gel (eluent: 3% ethyl acetate in hexanes) to provide bisallylic phthalimide **S4**.

iv. Bisallylic phthalimide **S4** (2.52 mmol, 1 equiv.) was dissolved in methanol (20 mL) and to this was added hydrazine hydrate (5.0 equiv.). The reaction mixture was refluxed for 12 h. It was then brought to room temperature where TLC showed complete consumption of the stating imide.

After reaction was complete, the reaction mixture was concentrated under reduced pressure. The residue was dissolved in dichloromethane (20 mL) and washed with water (20 mL). The aqueous layer was extracted with dichloromethane (3 × 10 mL). The combined organic layers were dried over anhydrous sodium sulfate, filtered and concentrated under reduced pressure to provide the crude bisallylic amine which was used for the next step without further purification.

v. The crude bisallylic amine was transferred to a 50 mL round bottom flask where it was dissolved in anhydrous dichloromethane (10 mL). The solution was cooled to 0 ºC in an ice-water bath. After stirring the solution at 0° C for 5 min, triethylamine (10.0 equiv.) and benzoyl chloride (10.0 equiv.) were added sequentially. The ice-water bath was removed, the reaction mixture was allowed to warm to room temperature and stirred until the reaction mixture showed complete disappearance of the amine. The reaction mixture was then poured into water (5 mL) and the layers were separated. The aqueous layer was extracted with dichloromethane (3 × 10 mL). The combined organic layers were dried over anhydrous sodium sulfate, filtered and concentrated under reduced pressure. The crude product was purified by flash column chromatography on silica gel [eluent: ethyl acetate/ hexanes (1:5)] to provide bisallylic amides **1**.

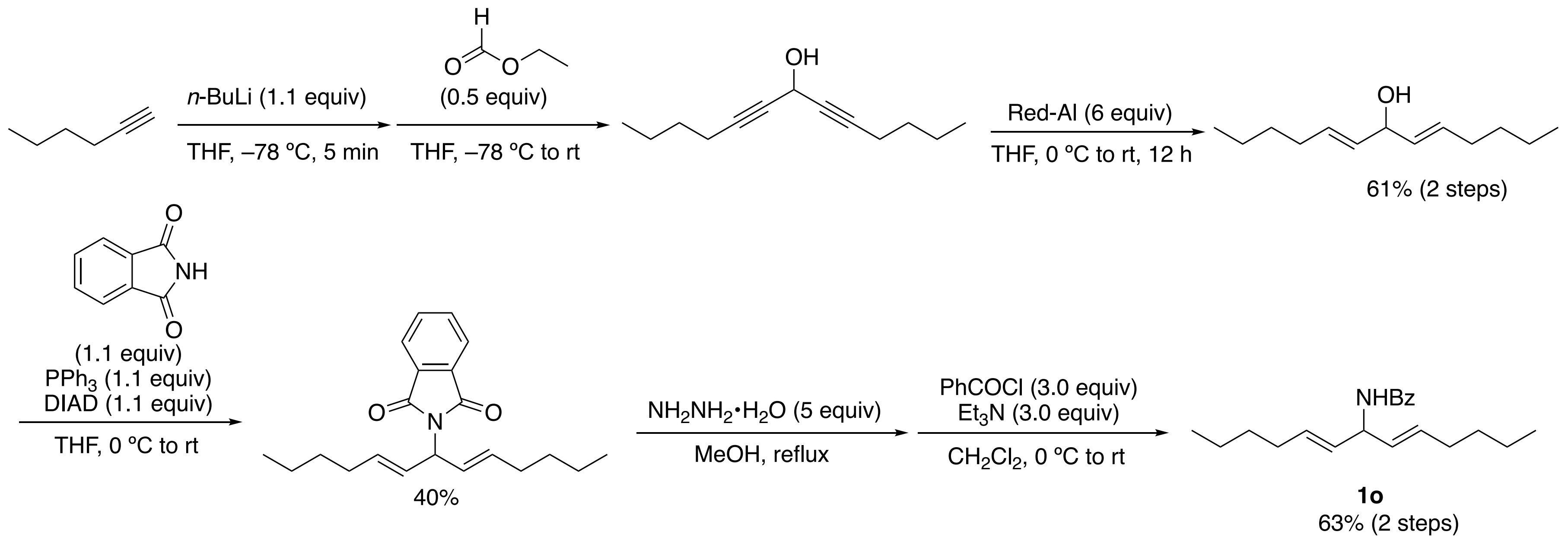

N-((5*E*,8*E*)-trideca-5,8-dien-7-yl)benzamide **1o**:

Bisallylic amide **1o** was prepared from 1-hexyne (13.9 mmol) using General Procedure B as a colorless oil with an overall yield of 15.4% over 4 steps (220.1 mg, 2.14 mmol). [Note: This product is volatile so care must be taken during concentration].

TLC (15% ethyl acetate in hexanes), R*f*: 0.58 (UV).

^{1}H NMR (500 MHz, $CDCl_3$): δ 7.83 – 7.74 (m, 2H), 7.53 – 7.41 (m, 3H), 6.08 (d, *J* = 8.2 Hz, 1H), 5.98 (ddt, *J* = 16.5, 5.8, 1.5 Hz, 1H), 5.68 (ddt, *J* = 15.4, 8.5, 1.3 Hz, 1H), 5.51 (ddt, *J* = 15.4, 5.8, 1.4 Hz, 1H), 5.43 (ddt, *J* = 15.4, 6.4, 1.5 Hz, 1H), 5.22 – 5.13 (m, 1H), 2.06 (td, *J* = 6.9, 4.7 Hz, 4H), 1.66 – 1.53 (m, 2H), 1.44 – 1.20 (m, 6H), 0.90 (t, *J* = 6.7 Hz, 6H). ^{13}C NMR (126 MHz, $CDCl_3$): δ 166.5, 166.2, 134.9, 134.8, 132.4, 131.9,

131.3, 131.2, 129.9, 128.9, 128.5, 128.5, 126.9, 126.9, 52.5, 51.4, 35.5, 32.0, 31.9, 31.7, 31.3, 31.3, 29.1, 25.8, 22.6, 22.2, 14.0, 13.9. HRMS (ESI) (m/z): calculated for $(M+H)^+$: $C_{20}H_{30}NO$ 300.2329; found: 300.2346. The spectral data match those previously reported for this compound.[4]

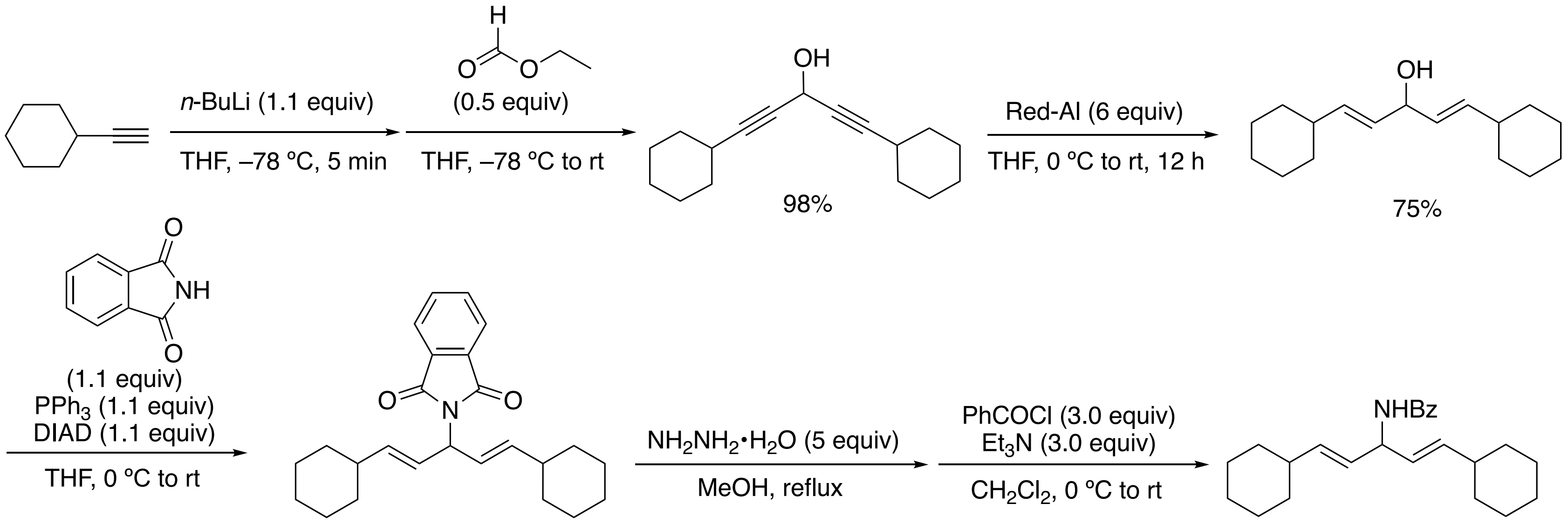

1p
45% (2 steps)

<u>*N*-((1*E*,4*E*)-1,5-dicyclohexylpenta-1,4-dien-3-yl)benzamide **1p**:</u>

Bisallylic amide **1p** was prepared from cyclohexylacetylene (13.9 mmol) using General Procedure B as a colorless oil with an overall 18% over 4 steps (622.2 mg, 2.48 mmol).

TLC (15% ethyl acetate in hexanes), R*f*: 0.58 (UV).

^{1}H NMR (500 MHz, $CDCl_3$): δ 7.76 (dd, *J* = 9.7, 7.8 Hz, 2H), 7.52 – 7.38 (m, 4H), 6.03 (d, *J* = 8.5 Hz, 1H), 5.94 (d, *J* = 8.6 Hz, 0.27H), 5.58 (dd, *J* = 15.5, 6.5 Hz, 2.27H), 5.42 (dd, *J* = 15.6, 5.7 Hz, 2H), 5.35 (dd, *J* = 15.6, 6.2 Hz, 0.27H), 5.15 (q, *J* = 6.5 Hz, 1H), 4.61 – 4.50 (m, 0.27H), 1.94 (ddd, *J* = 14.5, 11.0, 6.5 Hz, 2H), 1.75 – 1.50 (m, 15H), 1.35 – 0.93 (m, 13H). ^{13}C NMR (126 MHz, $CDCl_3$): δ 166.2, 138.0, 137.7, 134.9, 131.4,

131.2, 128.5, 127.5, 126.9, 126.9, 126.6, 52.3, 51.6, 40.4, 37.6, 33.4, 33.3, 32.9, 32.9, 32.8, 26.6, 26.4, 26.1, 26.0. HRMS (ESI) (m/z): calculated for $(M+H)^+$: $C_{24}H_{34}NO$ 352.2640; found: 352.2661.

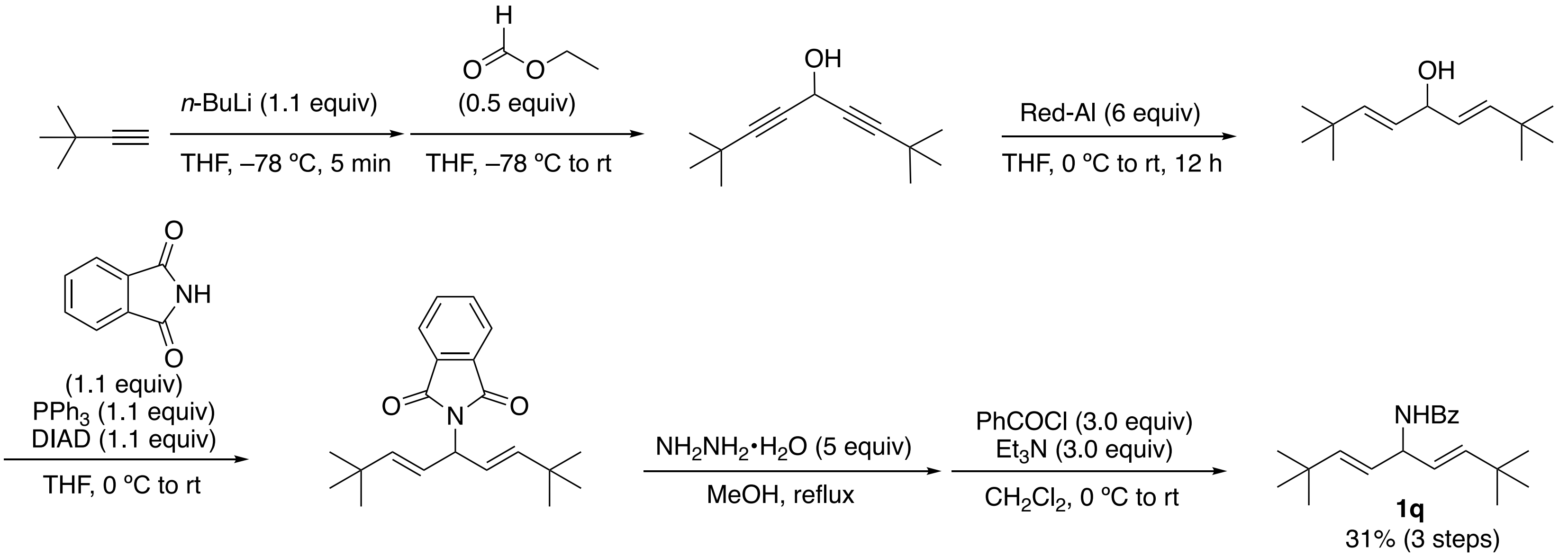

<u>*N*-((3*E*,6*E*)-2,2,8,8-tetramethylnona-3,6-dien-5-yl)benzamide **1q**:</u>

Bisallylic amide **1q** was prepared from 3,3-dimethylbut-1-yne using General Procedure B as a clear oil with a 10% overall yield over 4 steps (209.6 mg, 0.7 mmol). [Note: This product is volatile so care must be taken during concentration].

TLC (20% ethyl acetate in hexanes), R*f*: 0.70(UV).

^{1}H NMR (500 MHz, $CDCl_3$): δ 7.83 – 7.75 (m, 2H), 7.54 – 7.40 (m, 3H), 6.17 – 6.10 (m, 1H), 5.66 (dd, *J* = 15.7, 1.6 2H), 5.39 (dd, *J* = 15.7, 5.8 Hz, 2H), 5.19 (dtt, *J* = 10.0, 5.8, 1.4 Hz, 1H), 1.01 (s, 18H). ^{13}C NMR (126 MHz, $CDCl_3$): δ 166.5, 143.0, 131.3, 128.5, 126.9, 126.9, 124.1, 51.8, 32.9, 29.6. HRMS (ESI) (m/z): calculated for $(M+H)^+$: $C_{20}H_{30}NO$ 300.2327; found: 300.2342.

3. Optimization of the Reaction Conditions for Desymmetrization

3.1 Solvent

Table S1. The study of TFE-HFIP co-solvent system

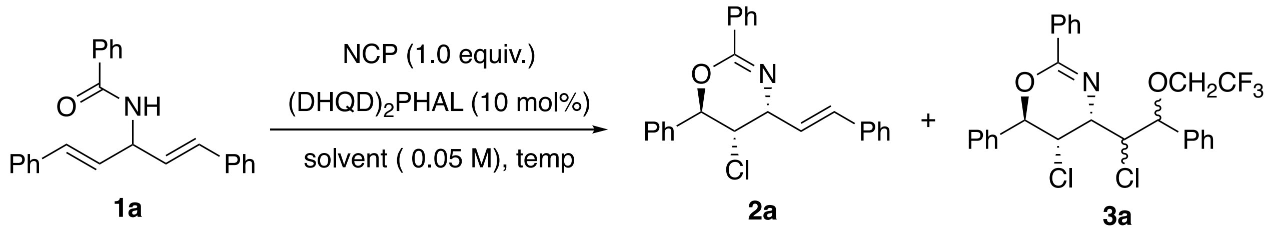

entry	temp (°C)	solvent (ratio)	time (h)	% yield (**2a**)[a]	% yield (**3a**)[a]	*dr* (**2a**)[b]	% *ee* (**2a**)[c]
1	rt	TFE	18	61 (76)	10	> 20 : 1	93
2	rt	HFIP	2	95 (100)	—	4.5 : 1	91
3	rt	TFE-HFIP (7 : 3)	12	91 (100)	—	4.5 : 1	91
4	−10	TFE-HFIP (7 : 3)	18	69 (71)	—	6.7 : 1	92
5	−30	TFE-HFIP (7 : 3)	18	55 (70)	—	10 : 1	96
6	−10	TFE-HFIP (1 : 1)	12	75 (90)	—	4 : 1	92
7	−10	TFE-HFIP (3 : 7)	12	86 (100)	—	4 : 1	97
8	−10	TFE-HFIP (6 : 4)	18	65 (80)	—	6 : 1	92
9	–30	TFE-HFIP (3 : 7)	18	62 (80)	—	6 : 1	92

[a] isolated yield, conversions are reported in the parentheses; [b] *dr* was determined form ^{1}H NMR; [c] *ee* was determined by chiral HPLC analysis.

Table S2. The study of other solvent systems

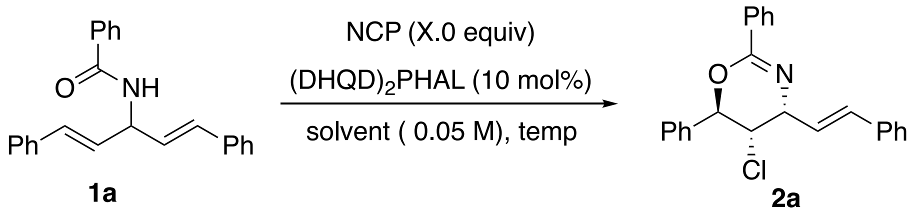

entry	NCP (equiv)	temp (°C)	solvent (ratio)	time (h)	% yield (**2a**)[a]	*dr* (**2a**)[b]	% *ee* (**2a**)[c]
1	NCP (1.0)	rt	TFE	18	61 (76)	> 20 : 1	93
2	NCP (1.0)	rt	HFIP	2	95 (100)	4.5 : 1	91
3	NCS (1.1)	rt	MeCN	28	32 (41)	3.8 : 1	47
4	NCP (1.1)	–30	DCM	24	60 (80)	8 : 1	82
5	NCP (1.1)	–30	TFE - HFIP (7 : 3)	18	65 (80)	10 : 1	98
6	NCP (1.1)	–30	TFE - MeCN (7 : 3)	22	68 (85)	> 20 : 1	84
7	NCP (1.1)	–30	TFE - DMF (7 : 3)	48	59 (76)	10 : 1	51
8	NCP (1.1)	–30	TFE - DCM (7 : 3)	18	91 (100)	> 20 : 1	98

[a] isolated yield, conversions are reported in the parentheses; [b] *dr* was determined form ^{1}H NMR; [c] *ee* was determined by chiral HPLC analysis.

3.2 Chlorenium Source

Table S3. The study of electrophilic chlorine source

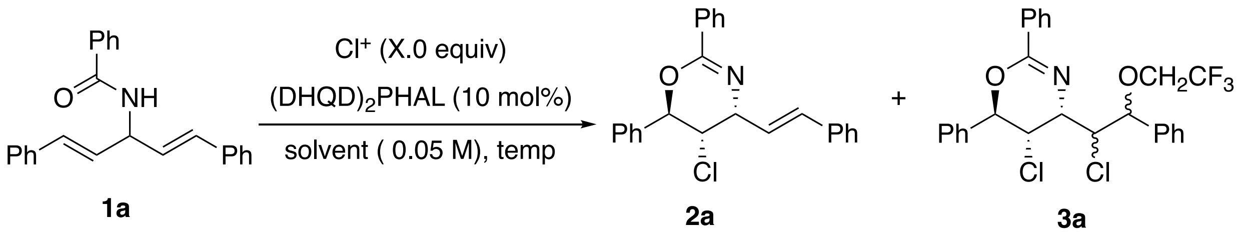

entry	NCP (equiv)	temp (°C)	solvent (ratio)	time (h)	% yield (**2a**)[a]	% yield (**3a**)[a]	*dr* (**2a**)[b]	% *ee* (**2a**)[c]
1	NCP (2.0)	–10	TFE-HFIP (7 : 3)	4	0	44	–	–
2	NCP (2.0)	–30	TFE-HFIP (7 : 3)	18	32 (100)	34	–	–
3	NCP (1.0)	–30	TFE-HFIP (7 : 3)	18	55 (70)	—	10 : 1	96
4	NCP (1.3)	–30	TFE-HFIP (7 : 3)	18	29 (67)	31	—	92
5	DCDMH (1.1)	–30	TFE-HFIP (1 : 1)	12	15 (100)	—	–	–
6	NCP (1.1)	–30	TFE-HFIP (7 : 3)	18	65 (80)	—	10:1	98
7	NCS (1.1)	–30	TFE-HFIP (7 : 3)	24	52 (100)	10	–	—

[a] isolated yield, conversions are reported in the parentheses; [b] *dr* was determined form ^{1}H NMR; [c] *ee* was determined by chiral HPLC analysis.

3.3 Additive

Table S4. The study of additive

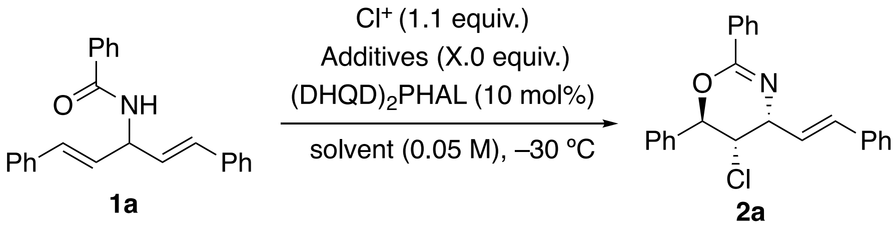

entry	NCP (equiv)	Additives (X.0 equiv.)	solvent (ratio)	time (h)	% yield (**2a**)[a]	*dr* (**2a**)[b]	% *ee* (**2a**)[c]
1	NCS (1.1)	$NaHCO_3$ (1.0)	TFE-HFIP (1 : 1)	12	91 (100)	5.5 : 1	93
2	NCS (1.1)	PPh_3 (1.0)	TFE-HFIP (1 : 1)	12	26 (38)	5.2 : 1	95
3	NCS (1.1)	BzOH (1.0)	TFE-HFIP (1 : 1)	18	48 (53)	7.9 : 1	94
4[d]	NCS (1.1)	$Yt(OTf)_3$ (0.1)	TFE-HFIP (7 : 3)	18	80 (95)	10 : 1	98
5	NCP (1.1)	$Yt(OTf)_3$ (0.1)	TFE-HFIP (7 : 3)	18	90 (100)	> 20 : 1	98
6	NCP (1.1)	AgOTf (0.1)	TFE-HFIP (7 : 3)	24	73 (88)	16 : 1	98
7	NCP (1.1)	$Zn(OTf)_2$ (0.1)	TFE-HFIP (7 : 3)	24	59 (70)	> 20 : 1	98
8	NCP (1.1)	4 Å MS	TFE-HFIP (7 : 3)	24	80 (82)	> 20 : 1	90

[a] isolated yield, conversions are reported in the parentheses; [b] *dr* was determined form ^{1}H NMR; [c] *ee* was determined by chiral HPLC analysis. [d] 7% of **3a** was observed.

3.4 Catalyst

Table S5. The study of catalyst

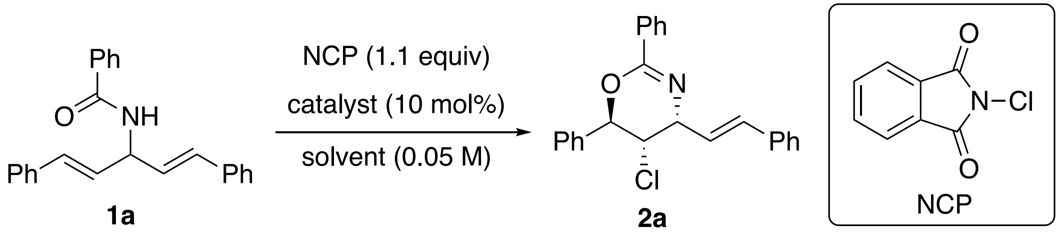

entry	NCP (equiv)	Temp (°C)	solvent (ratio)	time (h)	% yield (**2a**)[a]	*dr* (**2a**)[b]	% *ee* (**2a**)[c]
1	**A**	−30	TFE-HFIP (1 : 1)	17	59 (60)	1 : 4	2
2	**B**	−30	toluene	48	0 (0)	–	–
3[d]	$(DHQD)_2PHAL$	−30	TFE-HFIP (1 : 1)	12	80 (100)	7 : 1	94

[a] isolated yield, conversions are reported in the parentheses; [b] *dr* was determined form ^{1}H NMR; [c] *ee* was determined by chiral HPLC analysis. [d] reaction concentration is 0.025 M.

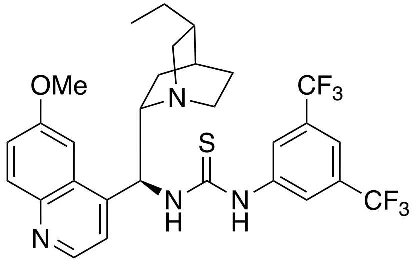

A

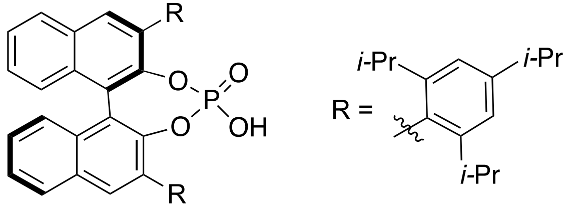

B- *(R)*-TRIP-PO_3H

4. Enantioselective Desymmetrization of Bisallylic Amides

General procedure C: Bisallylic amide **1** (0.10 mmol, 1.0 equiv), $(DHQD)_2PHAL$ (8 mg, 0.01 mmol, 0.1 equiv), 2,2,2-trifluoroethanol (0.7 mL) and dichloromethane (0.3 mL) were sequentially added to a 20 mL scintillation vial, and the reaction mixture was cooled to –30 °C in a cold bath with a recirculating chiller. After 5 min, when the reaction mixture became homogeneous, *N*-chlorophthalimide (20 mg, 0.11 mmol, 1.1 equiv) was added to the solution in one portion. The progress of the reaction was monitored through TLC, whereupon complete consumption of the starting material indicated the completion of the reaction. The reaction was quenched by adding sodium sulfite (10% aq. solution, 2 mL) and slowly warmed up to room temperature where it was stirred for an additional 10 min. The organic layer was separated, and the aqueous layer extracted with dichloromethane (3 X 5 mL). The combined organics were dried over Na_2SO_4 and concentrated under reduced pressure. Crude ^{1}H-NMR spectra was recorded to determine the diastereomeric ratio of the corresponding substrate. The crude product was purified via column chromatography (20 X 250 mm, 90:10 hexane: ethyl acetate as eluent) to generate **2** as the pure desired product. [Note: Although a few compounds nmr might show the leftover of a trace amount of solvents, but calculations of the yield were done after a thorough (overnight high vacuum) removal of the solvents].

General procedure D: Bisallylic amide **1** (0.10 mmol, 1.0 equiv), $(DHQD)_2PHAL$ (8 mg, 0.01 mmol, 0.1 equiv), 2,2,2-trifluoroethanol (0.7 mL) and hexafluoroisopropanol (0.3 mL) were sequentially added to a 20 mL scintillation vial, and the reaction mixture was cooled to –30 °C in a cold bath with a recirculating chiller. After 5 min, when the reaction mixture became homogeneous, *N*-chlorophthalimide (20 mg, 0.11 mmol, 1.1 equiv) was added to the solution in one portion. The progress of the reaction was monitored through TLC, whereupon complete consumption of the starting material indicated the completion of the reaction. The reaction was quenched by adding sodium sulfite (10% aq. solution, 2 mL) and slowly warmed up to room temperature where it was stirred for an additional 10 min. The organic layer was separated, and the aqueous layer extracted with dichloromethane (3 X 5 mL). The combined organics were dried over Na_2SO_4 and concentrated under reduced pressure. Crude ^{1}H-NMR spectra was recorded to determine the diastereomeric ratio of the corresponding substrate. The crude product was purified via column chromatography (20 X 250 mm, 90:10 hexane: ethyl acetate as eluent) to generate **2** as the pure desired product.

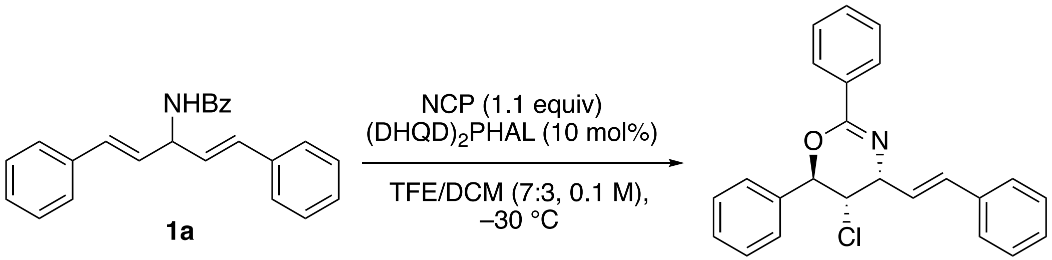

2a

91%, dr > 20:1, 98% ee

(4*R*,5*S*,6*R*)-5-chloro-2,6-diphenyl-4-((*E*)-styryl)-5,6-dihydro-4*H*-1,3-oxazine **2a**:

Oxazine **2a** was prepared from bisallylic-amide **1a** (136 mg, 0.40 mmol, 1.00 equiv.) by the General Procedure C. The diastereomeric ratio of the crude mixture was determined to be >20:1 by ^{1}H-NMR. The crude product was purified by flash column chromatography on silica gel (eluent: 10% ethyl acetate in hexanes) to give **2a** (135 mg, mp: 110 – 112 °C, 91%) as a yellow solid.

TLC (15% ethyl acetate in hexanes), R*f*: 0.54 (UV).

^{1}H NMR (500 MHz, $CDCl_3$): δ 8.14 – 8.07 (m, 2H), 7.51 – 7.19 (m, 13H), 6.69 (d, *J* = 15.7 Hz, 1H), 6.43 (dd, *J* = 15.8, 5.6 Hz, 1H), 5.47 (d, *J* = 6.1 Hz, 1H), 4.51 (t, *J* = 4.9 Hz, 1H), 4.43 – 4.36 (m, 1H). ^{13}C NMR (126 MHz, $CDCl_3$): δ 154.4, 137.7, 136.7, 133.4, 132.6, 131.1, 128.9, 128.9, 128.5, 128.2, 127.7, 127.6, 127.1, 126.7, 126.2, 79.2, 58.3, 54.8. IR (cm $^{-1}$): 3091, 1638, 1607, 1566, 1349, 1296, 1058, 855. HRMS (ESI) (m/z): calculated for $(M+H)^+$: $C_{24}H_{21}ClNO$ 374.1312; found: 374.1346. $[\alpha]_D^{20}$: +29.5° (C 1.0, CH_2Cl_2, *ee* = 98%). HPLC: Daicel Chiralpak IA, 5% IPA-Hex, 1 mL/min; 254 nm, $RT_{1(minor)}$ = 9.4 min, $RT_{2(major)}$ = 10.6 min.

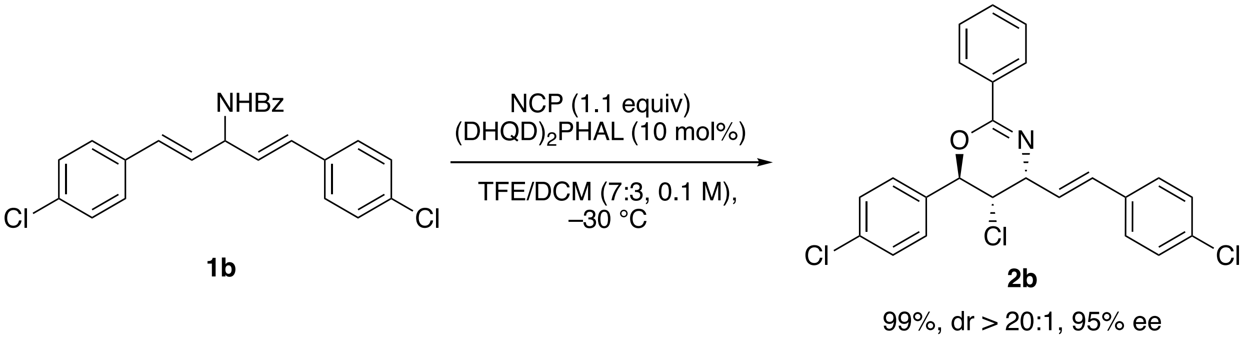

(4*R*,5*S*,6*R*)-5-chloro-6-(4-chlorophenyl)-4-((*E*)-4-chlorostyryl)-2-phenyl-5,6-dihydro-4*H*-1,3-oxazine **2b**:

Oxazine **2b** was prepared from bisallylic-amide **1b** (20.0 mg, 0.050 mmol, 1.00 equiv.) using General Procedure C. The diastereomeric ratio of the crude mixture was determined to be >20:1 by ^{1}H-NMR. The crude product was purified by flash column chromatography on silica gel (eluent: 5% ethyl acetate in hexanes) to give **2b** (21.9 mg, 99%) as an oily liquid.

TLC (15% ethyl acetate in hexanes), R*f*: 0.67 (UV).

^{1}H NMR (500 MHz, $CDCl_3$): δ 8.09 – 8.03 (m, 2H), 7.53 – 7.45 (m, 1H), 7.45 – 7.25 (m, 10H), 6.62 (dd, *J* = 15.8, 1.5 Hz, 1H), 6.42 (dd, *J* = 15.8, 5.5 Hz, 1H), 5.38 (d, *J* = 6.7 Hz, 1H), 4.51 (td, *J* = 4.3, 2.2 Hz, 1H), 4.33 (dd, *J* = 6.7, 4.3 Hz, 1H). ^{13}C NMR (126 MHz, $CDCl_3$): δ 154.4, 136.0, 135.1, 134.9, 133.4, 132.5, 132.2, 131.3, 129.1, 128.7, 128.3, 127.9, 127.8, 127.6, 127.4, 78.3, 57.9, 55.1. IR (cm $^{-1}$): 3050, 1658, 1516, 1309, 1058, 815, 696, 616. HRMS (ESI) (m/z): calculated for $(M+H)^+$: $C_{24}H_{19}Cl_3NO$ 444.0690; found: 444.0692. $[\alpha]_D^{20}$: +23.2º (C 1.0, CH_2Cl_2, *ee* = 95%). HPLC: Daicel Chiralpak IA, 5% IPA-Hex, 1 mL/min; 254 nm, $RT_{1(minor)}$ = 9.6 min, $RT_{2(major)}$ = 12.5 min.

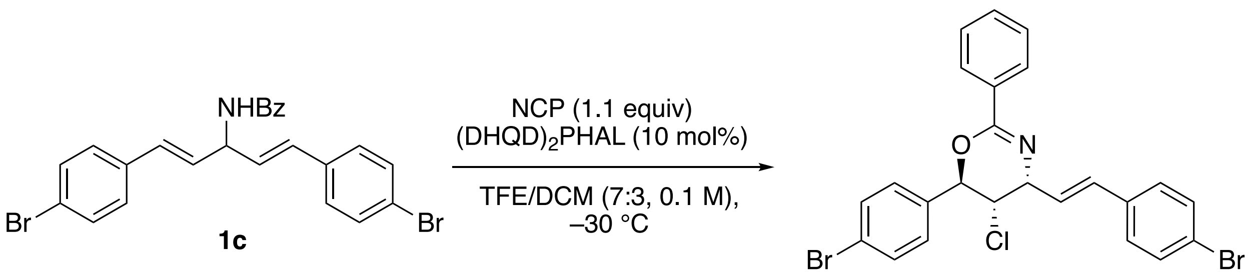

1c → **2c**

93%, dr > 20:1, 90% ee

(4*R*,5*S*,6*R*)-6-(4-bromophenyl)-4-((*E*)-4-bromostyryl)-5-chloro-2-phenyl-5,6-dihydro-4*H*-1,3-oxazine **2c**:

Oxazine **2c** was prepared from bisallylic-amide **1c** (12.0 mg, 0.024 mmol, 1.00 equiv.) using General Procedure C. The diastereomeric ratio of the crude mixture was determined to be >20:1 by ^{1}H-NMR. The crude product was purified by flash column chromatography on silica gel (eluent: 10% ethyl acetate in hexanes) to give **2c** (11.8 mg, 93%) as a yellow oil.

TLC (15% ethyl acetate in hexanes), R*f* : 0.49 (UV).

^{1}H NMR (500 MHz, $CDCl_3$): δ 8.06 (dt, *J* = 8.5, 1.3 Hz, 2H), 7.56 – 7.51 (m, 2H), 7.52 – 7.46 (m, 1H), 7.46 – 7.37 (m, 4H), 7.25 (ddd, *J* = 21.2, 8.3, 1.1 Hz, 4H), 6.61 (d, *J* = 15.8 Hz, 1H), 6.45 – 6.31 (m, 1H), 5.36 (d, *J* = 6.6 Hz, 1H), 4.50 (dt, *J* = 5.5, 2.8 Hz, 1H), 4.33 (ddd, *J* = 6.6, 4.3, 1.1 Hz, 1H). ^{13}C NMR (126 MHz, $CDCl_3$): δ 154.4, 136.5, 135.6, 132.6, 132.2, 132.1, 131.67, 131., 128.31, 128.2, 128.1, 127.5, 127.5, 123.1, 121.6, 78.3, 57.8, 55.1. IR (cm $^{-1}$): 3107, 1630, 1516, 1330, 1058, 915, 646. HRMS (ESI) (m/z): calculated for $(M+H)^+$: $C_{24}H_{19}ClNOBr_2$ 529.9522; found: 529.9521. $[\alpha]_D^{20}$: +20.0° (C 1.0, CH_2Cl_2, *ee* = 90%). HPLC: Daicel Chiralpak IA, 5% IPA- Hex, 1 mL/min; 254 nm, $RT_{1(minor)}$ = 10.5 min, $RT_{2(major)}$ = 13.9 min.

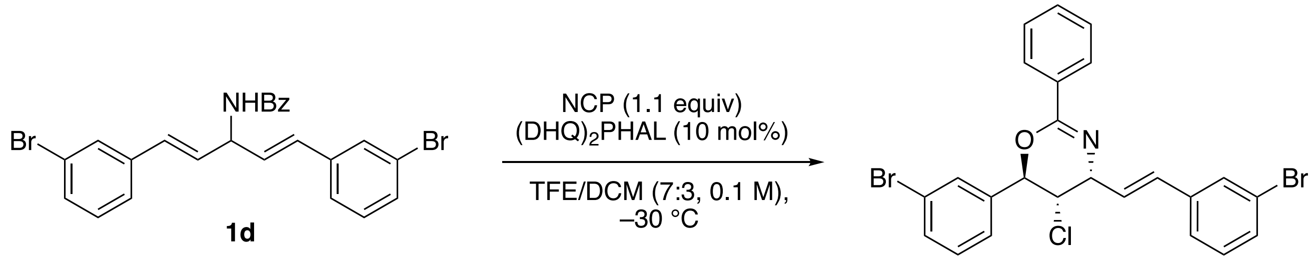

(4*R*,5*S*,6*R*)-6-(3-bromophenyl)-4-((*E*)-3-bromostyryl)-5-chloro-2-phenyl-5,6-dihydro-4*H*-1,3-oxazine **2d**:

Oxazine **2d** was prepared from bisallylic-amide **1d** (12.0 mg, 0.024 mmol, 1.00 equiv.) using General Procedure C using [(DHQ)2PHAL] instead of [(DHQD)2PHAL] as the catalyst. The diastereomeric ratio of the crude mixture was determined to be >20:1 by ^{1}H-NMR. The crude product was purified by flash column chromatography on silica gel (eluent: 10% ethyl acetate in hexanes) to give **2d** (12.2 mg, 96%) as a colorless oil.

TLC (15% ethyl acetate in hexanes), R*f*: 0.53 (UV).

^{1}H NMR (500 MHz, $CDCl_3$): δ 8.10 – 8.04 (m, 2H), 7.57 (d, *J* = 1.8 Hz, 1H), 7.55 – 7.47 (m, 3H), 7.43 (dd, *J* = 8.2, 6.9 Hz, 2H), 7.37 – 7.26 (m, 4H), 7.17 (t, *J* = 7.8 Hz, 1H), 6.62 (dd, *J* = 15.8, 1.5 Hz, 1H), 6.46 – 6.40 (m, 1H), 5.39 – 5.24 (m, 1H), 4.55 – 4.51 (m, 1H), 4.35 (dd, *J* = 6.6, 4.3 Hz, 1H). ^{13}C NMR (126 MHz, $CDCl_3$): δ 154.4, 139.7, 138.7, 132.4, 132.2, 132.2, 131.3, 130.6, 130.4, 130.1, 129.5, 128.3, 128.3, 127.6, 125.3, 125.1, 122.9, 122.7, 78.2, 57.7, 54.9. IR (cm $^{-1}$): 3100, 1650, 1516, 1330, 1058, 915, 646. HRMS (ESI) (m/z): calculated for $(M+H)^+$: $C_{24}H_{19}ClNOBr_2$ 529.9522; found: 529.9539. $[\alpha]_D^{20}$: +8.1° (C 1.0, CH_2Cl_2, *ee* = 97%). HPLC: Daicel Chiralpak AD-H, 5% IPA- Hex, 1 mL/min; 254 nm, $RT_{1(major)}$ = 9.2 min, $RT_{2(minor)}$ = 10.6 min.

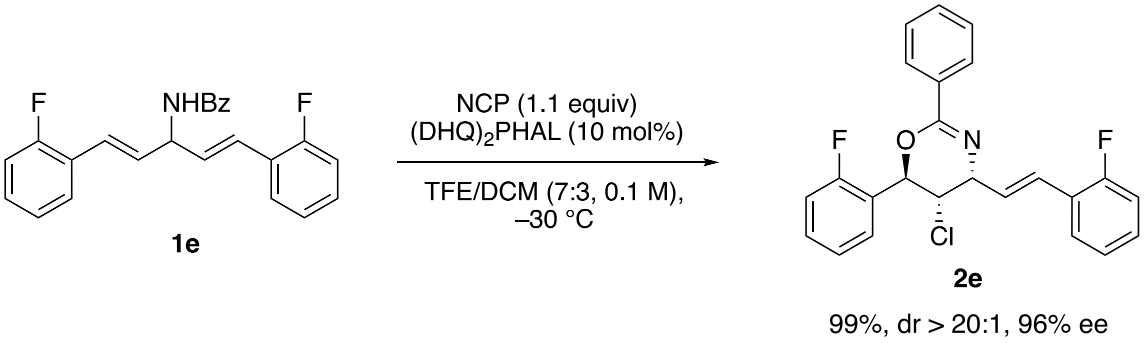

(4*R*,5*S*,6*R*)-5-chloro-6-(2-fluorophenyl)-4-((*E*)-2-fluorostyryl)-2-phenyl-5,6-dihydro-4*H*-1,3-oxazine **2e**:

Oxazine **2e** was prepared from bisallylic amide **1e** (36.0 mg, 0.10 mmol, 1.00 equiv.) using General Procedure C using [$(DHQ)_2$PHAL] instead of [$(DHQD)_2$PHAL] as the catalyst. The diastereomeric ratio of the crude mixture was determined to be >20:1 by ^{1}H-NMR. The crude product was purified by flash column chromatography on silica gel (eluent: 10% ethyl acetate in hexanes) to give **2e** (40.1 mg, mp: 109 - 111 °C, 99%) as a yellow solid.

TLC (15% ethyl acetate in hexanes), R*f* : 0.57 (UV).

^{1}H NMR (500 MHz, $CDCl_3$): δ 8.14 – 8.08 (m, 2H), 7.50 (tq, *J* = 6.6, 1.7 Hz, 2H), 7.47 – 7.35 (m, 3H), 7.31 (td, *J* = 7.6, 1.7 Hz, 1H), 7.23 – 7.05 (m, 4H), 7.02 (ddd, *J* = 10.7, 8.2, 1.2 Hz, 1H), 6.89 (d, *J* = 15.9 Hz, 1H), 6.48 (dd, *J* = 15.9, 5.4 Hz, 1H), 5.80 (d, *J* = 5.0 Hz, 1H), 4.55 (t, *J* = 4.4 Hz, 1H), 4.50 – 4.43 (m, 1H). ^{13}C NMR (126 MHz, $CDCl_3$): δ 161.3, 160.6, 159.3, 158.6, 154.2, 132.4, 131.2, 130.8, 129.8, 127.8, 125.9, 125.1, 124.8, 124.1, 116.0, 74.8, 74.82, 56.4, 56.3, 54.6. IR (cm $^{-1}$): 3100, 1610, 1510, 1430, 1100, 686. HRMS (ESI) (m/z): calculated for $(M+H)^+$: $C_{24}H_{19}ClNOF_2$ 410.1123; found: 410.1150. $[\alpha]_D^{20}$: +39.7° (C 1.0, CH_2Cl_2). HPLC: Daicel Chiralpak IA, 5% IPA-Hex, 1 mL/min; 254 nm, $RT_{1(major)}$ = 6.5 min, $RT_{2(minor)}$ = 9.0 min.

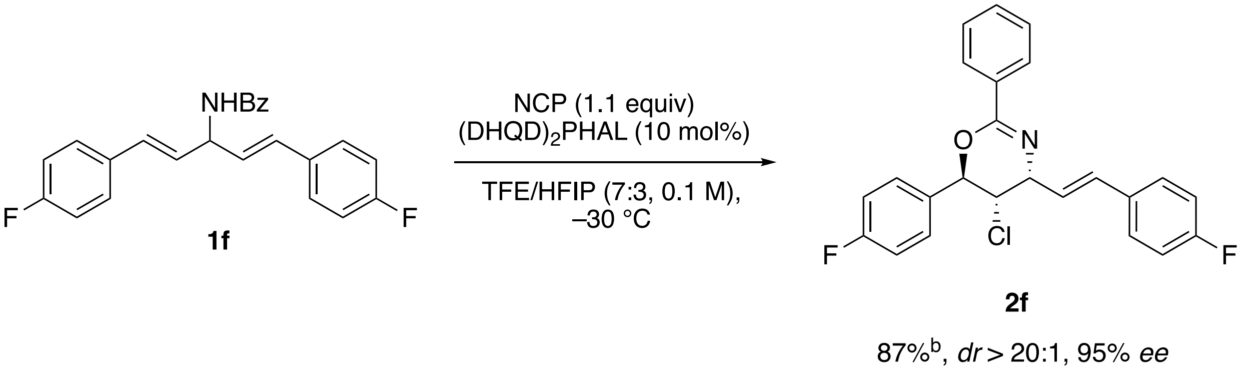

(4*R*,5*S*,6*R*)-5-chloro-6-(4-fluorophenyl)-4-((*E*)-4-fluorostyryl)-2-phenyl-5,6-dihydro-4*H*-1,3-oxazine **2f**:

Oxazine **2f** was prepared from bisallylic-amide **1f** (19.0 mg, 0.051 mmol, 1.00 equiv.) using General Procedure D. The diastereomeric ratio of the crude mixture was determined to be >20:1 by ^{1}H-NMR. The crude product was purified by flash column chromatography on silica gel (eluent: 10% ethyl acetate in hexanes) to give **2f** (18.2 mg, mp: 110 - 112 °C, 87%) as a yellow solid.

TLC (15% ethyl acetate in hexanes), R*f*: 0.48 (UV).

^{1}H NMR (500 MHz, $CDCl_3$): δ 8.09 – 8.04 (m, 2H), 7.52 – 7.46 (m, 1H), 7.46 – 7.28 (m, 6H), 7.10 (m, 2H), 7.00 (m, 2H), 6.63 (d, *J* = 15.7 Hz, 1H), 6.37 (dd, *J* = 15.8, 5.5 Hz, 1H), 5.38 (d, *J* = 6.8 Hz, 1H), 4.55 – 4.49 (m, 1H), 4.34 (dd, *J* = 6.8, 4.4 Hz, 1H). ^{13}C NMR (126 MHz, $CDCl_3$): δ 163.9, 163.4, 161.9, 161.4, 154.5, 133.4, 132.8, 132.6, 131.3, 128.3, 127.5, 126.5, 115.9, 78., 58.16, 55.3. IR (cm $^{-1}$): 3101, 1645, 1510, 1430, 1100, 686. HRMS (ESI) (m/z): calculated for $(M+H)^+$: $C_{24}H_{19}ClNOF_2$ 410.1123; found: 410.1129. $[\alpha]_D^{20}$: +25.5° (C 1.0, $CDCl_3$, *ee* = 95%). HPLC: Daicel Chiralpak IA, 5% IPA-Hex, 1 mL/min; 254 nm, $RT_{1(minor)}$ = 9.0 min, $RT_{2(major)}$ = 13.3 min.

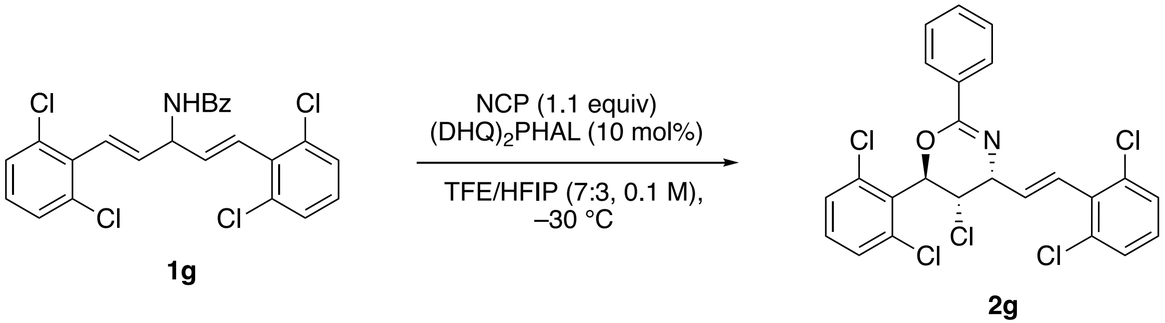

65%[b], *dr* > 20:1, 74% *ee*

(4*R*,5*S*,6*R*)-5-chloro-6-(2,6-dichlorophenyl)-4-((*E*)-2,6-dichlorostyryl)-2-phenyl-5,6-dihydro-4*H*-1,3-oxazine **2g**:

Oxazine **2g** was prepared from bisallylic-amide **1g** (0.051 mmol, 1.00 equiv.) using General Procedure D using [(DHQ)$_2$PHAL] instead of [(DHQD)$_2$PHAL] as the catalyst. The diastereomeric ratio of the crude mixture was determined to be >20:1 by ^{1}H-NMR. The crude product was purified by flash column chromatography on silica gel (eluent: 10% ethyl acetate in hexanes) to give **2g** (18.2 mg, 65%) as a colorless oil.

TLC (15% ethyl acetate in hexanes), R*f*: 0.48 (UV).

^{1}H NMR (500 MHz, $CDCl_3$): δ 8.12 – 7.95 (m, 2H), 7.52 – 7.44 (m, 1H), 7.43 – 7.36 (m, 4H), 7.32 (d, *J* = 8.1 Hz, 2H), 7.29 (dd, *J* = 8.6, 7.5 Hz, 1H), 7.11 (t, *J* = 8.0 Hz, 1H), 6.70 (dd, *J* = 16.2, 4.5 Hz, 1H), 6.59 (dd, *J* = 16.3, 1.7 Hz, 1H), 6.32 (d, *J* = 11.0 Hz, 1H), 5.25 (dd, *J* = 11.0, 5.2 Hz, 1H), 4.89 (td, *J* = 4.9, 1.7 Hz, 1H). ^{13}C NMR (126 MHz, $CDCl_3$): δ 173.4, 155.9, 134.7, 134.6, 134.0, 132.3, 131.3, 131.0, 130.7, 129.0, 128.8, 128.5, 128.5, 128.3, 127.9, 74.2, 57.8, 53.1. IR (cm $^{-1}$): 3050, 1658, 1516, 1309, 1058, 815, 696, 616. HRMS (ESI) (m/z): calculated for $(M+H)^+$: $C_{24}H_{19}Cl5NO$ 511.9645; found: 511.9655. $[\alpha]_D^{20}$: +15.5° (C 1.0, $CDCl_3$). HPLC: Daicel Chiralpak IA, 5% IPA-Hex, 1 mL/min; 254 nm, $RT_{1(major)}$ = 11.0 min, $RT_{2(minor)}$ = 38.6 min.

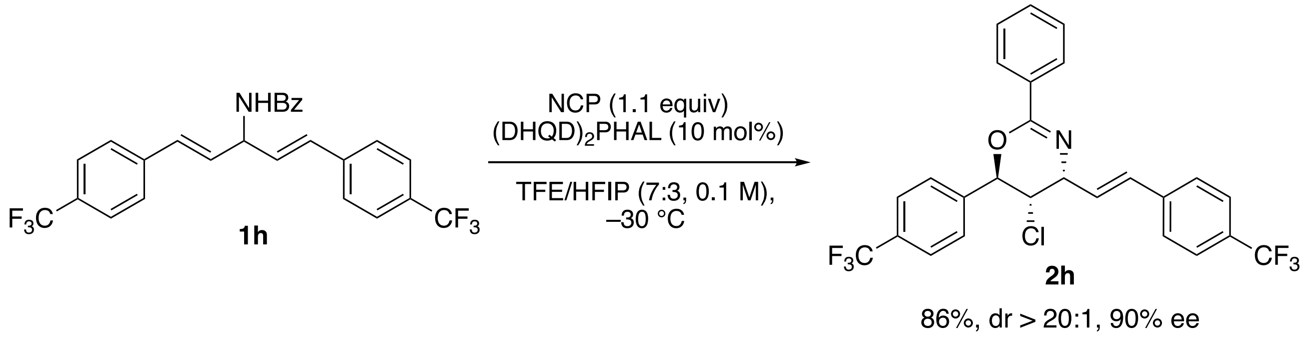

(4*R*,5*S*,6*R*)-5-chloro-2-phenyl-6-(4-(trifluoromethyl)phenyl)-4-((*E*)-4-(trifluoromethyl)styryl)-5,6-dihydro-4*H*-1,3-oxazine **2h**:

Oxazine **2h** was prepared from bisallylic amide **1h** (19.0 mg, 0.040 mmol, 1.00 equiv.) by General Procedure D. The diastereomeric ratio of the crude mixture was determined to be >20:1 by ^{1}H-NMR. The crude product was purified by flash column chromatography on silica gel (eluent: 10% ethyl acetate in hexanes) to give **2h** (17.5 mg, 86%) as a yellowish green oil.

TLC (15% ethyl acetate in hexanes), R*f* : 0.43 (UV).

^{1}H NMR (500 MHz, $CDCl_3$): δ 8.09 (dd, *J* = 8.2, 3.5 Hz, 2H), 7.69 (dd, *J* = 8.4, 3.4 Hz, 2H), 7.50 (dddd, *J* = 29.0, 24.7, 8.4, 4.5 Hz, 9H), 6.73 (dd, *J* = 15.9, 3.5 Hz, 1H), 6.54 (dt, *J* = 15.9, 4.6 Hz, 1H), 5.47 (dd, *J* = 6.7, 3.6 Hz, 1H), 4.54 (t, *J* = 4.6 Hz, 1H), 4.39 (dt, *J* = 7.8, 3.8 Hz, 1H). ^{13}C NMR (126 MHz, $CDCl_3$): δ 154.4, 141.2, 140.0, 132.6, 132.1, 131.5, 131.2, 129.7, 129.5, 129.3, 128.4, 127.6, 126.9, 126.8, 125.9(q), 125.5(q), 125.2, 78.3, 57.5, 55.0. IR (cm $^{-1}$): 3070, 1660, 1516, 1440, 1309, 1058, 815. HRMS (ESI) (m/z): calculated for $(M+H)^+$: $C_{26}H_{19}ClF_6NO$ 510.1059; found: 510.1074. $[\alpha]_D^{20}$: +13.8° (C 1.0, CH_2Cl_2). HPLC: Daicel Chiralpak IA, 5% IPA-Hex, 1 mL/min; 254 nm, $RT_{1(minor)}$ = 6.5 min, $RT_{2(major)}$ = 9.5 min.

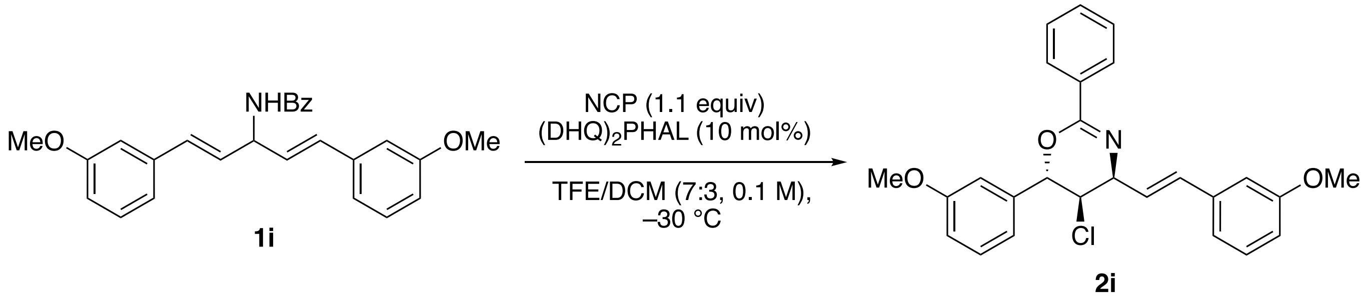

92%, dr = 7:1, 97% ee

(4*S*,5*R*,6*S*)-5-chloro-6-(3-methoxyphenyl)-4-((*E*)-3-methoxystyryl)-2-phenyl-5,6-dihydro-4*H*-1,3-oxazine **2i**:

Oxazine **2i** was prepared from bisallylic amide **1i** (19.0 mg, 0.048 mmol, 1.00 equiv.) by General Procedure C using [(DHQ)$_2$PHAL] instead of [(DHQD)$_2$PHAL] as the catalyst. The diastereomeric ratio of the crude mixture was determined to be 7:1 by ^{1}H-NMR. The crude product was purified by flash column chromatography on silica gel (eluent: 10% ethyl acetate in hexanes) to give **2i** (19.1 mg, mp: 72 – 76 °C, 92%) as a white solid.

TLC (20% ethyl acetate in hexanes), R*f* : 0.46 (UV).

^{1}H NMR (500 MHz, $CDCl_3$): δ 8.10 (dt, *J* = 7.1, 1.4 Hz, 2H), 7.51 – 7.45 (m, 1H), 7.42 (dd, *J* = 8.3, 6.8 Hz, 2H), 7.32 (t, *J* = 7.9 Hz, 1H), 7.27 – 7.18 (m, 1H), 7.02 (dt, *J* = 7.7, 1.2 Hz, 1H), 6.99 – 6.85 (m, 4H), 6.79 (ddd, *J* = 8.3, 2.6, 0.9 Hz, 1H), 6.66 (dd, *J* = 15.8, 1.5 Hz, 1H), 6.42 (dd, *J* = 15.8, 5.5 Hz, 1H), 5.44 (d, *J* = 5.8 Hz, 1H), 4.50 (ddd, *J* = 5.6, 4.0, 1.5 Hz, 1H), 4.40 (dd, *J* = 5.8, 4.1 Hz, 1H), 3.79 (d, *J* = 5.6 Hz, 6H). ^{13}C NMR (126 MHz, $CDCl_3$): δ 159.9, 159.7, 154.4, 139.3, 138.2, 133.2, 132.6, 131.1, 130.0, 129.5, 128.2, 127.6, 127.5, 119.3, 118.3, 114.1, 113.4, 112.0, 111.9, 79.1, 58.2, 55.3, 55.3, 54.7. IR (cm $^{-1}$): 3050, 1658, 1516, 1309, 1100, 1058, 796. HRMS (ESI) (m/z): calculated for $(M+H)^+$: $C_{26}H_{25}ClNO_3$ 434.1523; found: 434.1518. $[\alpha]_D^{20}$: –18.1° (C 1.0, CH_2Cl_2). HPLC: Daicel Chiralpak AD-H, 3% IPA- Hex, 1 mL/min; 254 nm, major diastereomer: $RT_{1(major)}$ = 24.5 min, $RT_{2(minor)}$ = 27.2 min.

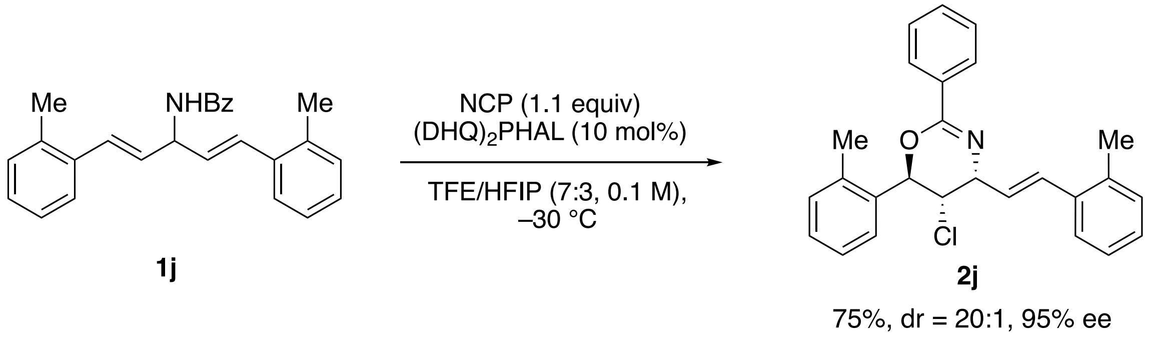

(4*R*,5*S*,6*R*)-5-chloro-4-((*E*)-2-methylstyryl)-2-phenyl-6-(*o*-tolyl)-5,6-dihydro-4*H*-1,3-oxazine **2j**:

Oxazine **2j** was prepared from bisallylic amide **1j** (18.4 mg, 0.050 mmol, 1.00 equiv.) by General Procedure D using [$(DHQ)_2$PHAL] instead of [$(DHQD)_2$PHAL] as the catalyst. The diastereomeric ratio of the crude mixture was determined to be 20:1 by ^{1}H-NMR.The crude product was purified by flash column chromatography on silica gel (eluent: 10% ethyl acetate in hexanes) to give **2j** (15.1 mg, mp: 107 – 110 °C, 75%) as a yellow solid.

TLC (15% ethyl acetate in hexanes), R*f*: 0.53 (UV).

^{1}H NMR (500 MHz, $CDCl_3$): δ 8.13 – 8.03 (m, 2H), 7.52 – 7.37 (m, 4H), 7.34 – 7.20 (m, 4H), 7.19 – 7.10 (m, 3H), 6.95 (dd, *J* = 15.5, 1.5 Hz, 1H), 6.26 (dd, *J* = 15.6, 5.6 Hz, 1H), 5.75 (d, *J* = 5.4 Hz, 1H), 4.56 (s, 1H), 4.39 (dd, *J* = 5.4, 3.9 Hz, 1H), 2.42 (s, 3H), 2.34 (s, 3H). ^{13}C NMR (126 MHz, $CDCl_3$): δ 154.7, 136.2, 135.9, 135.6, 135.2, 132.7, 131.5, 131.1, 130.9, 130.2, 128.8, 128.8, 128.2, 127.6, 127.6, 126.7, 126.1, 126.1, 125.8, 76.9, 57.2, 54.7, 19.9, 19.2. IR (cm^{-1}): 3050, 1658, 1516, 1309, 1100, 1058, 796. HRMS (ESI) (m/z): calculated for $(M+H)^+$: $C_{26}H_{25}ClNO$ 404.1783; found: 402.1781. $[\alpha]_D^{20}$: +33.9° (C 1.0, CH_2Cl_2). HPLC: Daicel Chiralpak IA, 5% IPA-Hex, 1 mL/min; 254 nm, $RT_{1(major)}$ = 5.4 min, $RT_{2(minor)}$ = 7.9 min.

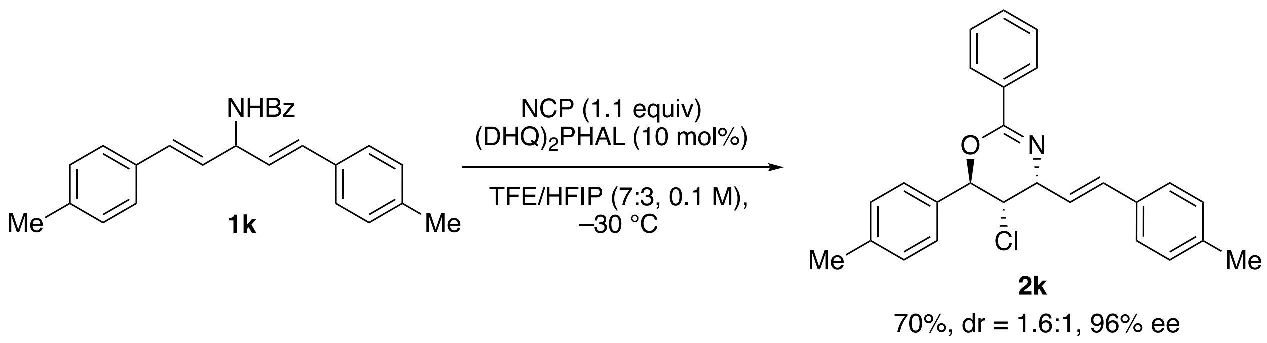

(4*R*,5*S*,6*R*)-5-chloro-4-((*E*)-4-methylstyryl)-2-phenyl-6-(*p*-tolyl)-5,6-dihydro-4*H*-1,3-oxazine **2k**:

Oxazine **2k** was prepared from bisallylic amide **1k** (18.4 mg, 0.050 mmol, 1.00 equiv.) by General Procedure D using [(DHQ)$_2$PHAL] instead of [(DHQD)$_2$PHAL] as the catalyst. The diastereomeric ratio of the crude mixture was determined to be 1.6:1 by ^{1}H-NMR.The crude product was purified by flash column chromatography on silica gel (eluent: 10% ethyl acetate in hexanes) to give **2k** (14.1 mg, mp: 134 – 136 °C, 70%) as a greenish yellow solid.

TLC (30% ethyl acetate in hexanes), R*f* : 0.79 (UV).

^{1}H NMR (500 MHz, $CDCl_3$): δ 8.14 – 8.02 (m, 2H), 7.53 – 7.37 (m, 2H), 7.31 (d, *J* = 8.1 Hz, 2H), 7.28 – 7.17 (m, 6H), 7.11 (d, *J* = 7.8 Hz, 2H), 6.63 (dd, *J* = 15.8, 1.4 Hz, 1H), 6.38 (dd, *J* = 15.8, 5.6 Hz, 1H), 5.40 (d, *J* = 6.3 Hz, 1H), 4.50 (ddd, *J* = 5.7, 4.1, 1.5 Hz, 1H), 4.37 (dd, *J* = 6.3, 4.1 Hz, 1H), 2.36 (s, 3H), 2.32 (s, 3H). ^{13}C NMR (126 MHz, $CDCl_3$): δ 154.5, 138.8, 137.5, 134.7, 133.9, 133.2, 132.6, 131.1, 129.5, 129.2, 128.2, 127.6, 126.6, 126.2, 126.0, 79.0, 58.4, 55.1, 21.2, 21.2. IR (cm $^{-1}$): 3045, 1670, 1516, 1309, 1100, 1018, 896. HRMS (ESI) (m/z): calculated for $(M+H)^+$: $C_{26}H_{25}ClNO$ 402.1625; found: 402.1648. $[\alpha]_D^{20}$: +8.6° (C 0.4, CH_2Cl_2). HPLC: Daicel Chiralpak OD-H, 5% IPA-Hex, 1 mL/min; 254 nm, $RT_{1(major)}$ = 5.3 min, $RT_{2(minor)}$ = 7.6 min.

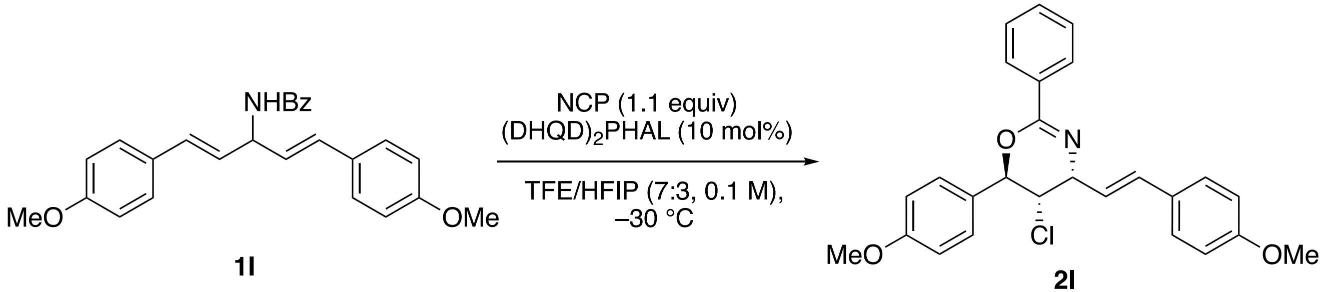

1l

2l

63%, dr = 1.3:1, 86% ee

(4*R*,5*S*,6*R*)-5-chloro-6-(4-methoxyphenyl)-4-((*E*)-4-methoxystyryl)-2-phenyl-5,6-dihydro-4*H*-1,3-oxazine **2l**:

Oxazine **2l** was prepared from bisallylic amide **1l** (17.0 mg, 0.042 mmol, 1.00 equiv.) by General Procedure D. The diastereomeric ratio of the crude mixture was determined to be 1.3:1 by ^{1}H-NMR. The crude product was purified by flash column chromatography on silica gel (eluent: 10% ethyl acetate in hexanes) to give **2l** (11.5 mg, 63%) as a colorless oil.

TLC (30% ethyl acetate in hexanes), R*f* : 0.67 (UV).

^{1}H NMR (500 MHz, $CDCl_3$): δ 8.12 – 8.04 (m, 2H), 7.55 – 7.39 (m, 3H), 7.39 – 7.33 (m, 2H), 7.32 – 7.27 (m, 2H), 6.96 – 6.90 (m, 2H), 6.89 – 6.82 (m, 2H), 6.66 – 6.57 (m, 1H), 6.33 (dd, *J* = 15.8, 5.7 Hz, 1H), 5.39 (d, *J* = 6.7 Hz, 1H), 4.54 (ddd, *J* = 5.7, 4.2, 1.5 Hz, 1H), 4.37 (dd, *J* = 6.7, 4.2 Hz, 1H), 3.83 (s, 3H), 3.81 (s, 3H). ^{13}C NMR (126 MHz, $CDCl_3$): δ 159.9, 159.3, 154.6, 132.9, 132.6, 131.0, 129.7, 129.6, 128.2, 127.9, 127.7, 127.6, 124.8, 114.2, 113.9, 78.6, 58.6, 55.4, 55.3, 55.3. IR (cm $^{-1}$): 3050, 1650, 1516, 1309, 1100, 1058, 756. HRMS (ESI) (m/z): calculated for $(M+H)^+$: $C_{26}H_{25}NO_3Cl$ 434.1523; found: 434.1509. HPLC: Daicel Chiralpak IA, 5% IPA-Hex, 1 mL/min; 254 nm, $RT_{1(minor)}$ = 18.9 min, $RT_{2(major)}$ = 24.1 min.

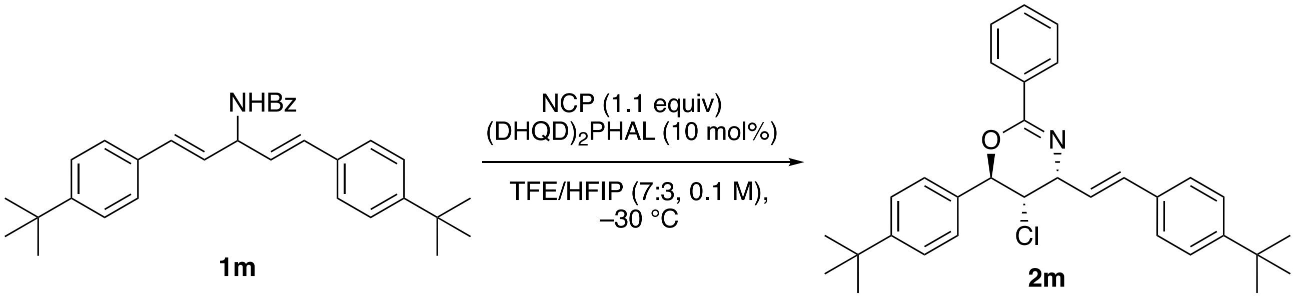

1m

2m
41%, dr = 2.8:1, 58% ee

(4*R*,5*S*,6*R*)-6-(4-(*tert*-butyl)phenyl)-4-((*E*)-4-(*tert*-butyl)styryl)-5-chloro-2-phenyl-5,6-dihydro-4*H*-1,3-oxazine **2m**:

Oxazine **2m** was prepared from bisallylic amide **1m** (22.6 mg, 0.050 mmol, 1.00 equiv.) by General Procedure C. The diastereomeric ratio of the crude mixture was determined to be 2.8:1 by ^{1}H-NMR. The crude product was purified by flash column chromatography on silica gel (eluent: 10% ethyl acetate in hexanes) to give **2m** (10.0 mg, mp: 152 – 155 °C, 41%) as a yellow solid.

TLC (30% ethyl acetate in hexanes), R*f*: 0.89 (UV).

^{1}H NMR (500 MHz, $CDCl_3$): δ 8.13 – 8.04 (m, 2H), 7.51 – 7.30 (m, 9H), 7.26 (d, *J* = 8.3 Hz, 2H), 6.66 (d, *J* = 15.9 Hz, 1H), 6.39 (dd, *J* = 15.8, 5.5 Hz, 1H), 5.43 (d, *J* = 6.0 Hz, 1H), 4.53 – 4.49 (m, 1H), 4.38 (dd, *J* = 6.0, 4.1 Hz, 1H), 1.30 (d, *J* = 8.2 Hz, 18H). ^{13}C NMR (126 MHz, $CDCl_3$): δ 166.6, 161.3, 159.3, 134.3, 131.7, 130.6, 130.6, 129.2, 129.1, 128.6, 127.8, 127.8, 127.1, 124.5, 124.5, 124.3, 124.2, 124.1, 115.9, 115.7, 53.4. IR (cm $^{-1}$): 3005, 1618, 1516, 1309, 796. HRMS (ESI) (m/z): calculated for $(M+H)^+$: $C_{32}H_{37}ClNO$ 488.2722; found: 488.2721. HPLC: Daicel Chiralpak IA, 1% IPA-Hex, 1 mL/min; 254 nm, $RT_{1(minor)}$ = 8.3 min, $RT_{2(major)}$ = 9.1 min.

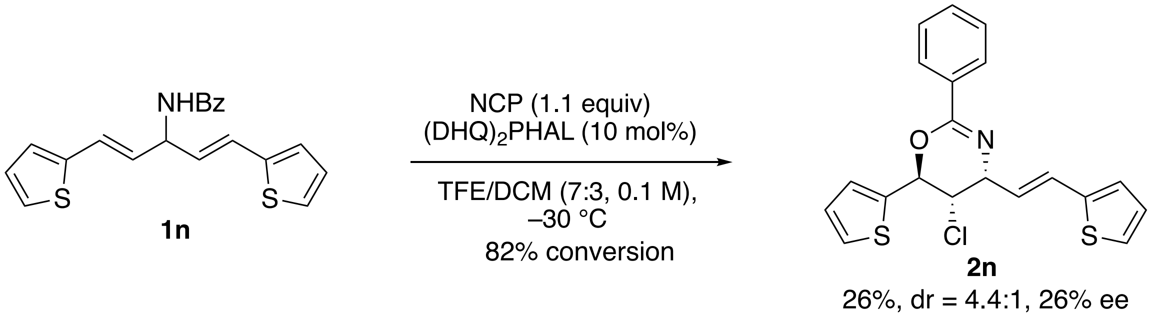

(4*R*,5*S*,6*R*)-5-chloro-2-phenyl-6-(thiophen-2-yl)-4-((*E*)-2-(thiophen-2-yl)vinyl)-5,6-dihydro-4*H*-1,3-oxazine **2n**:

Oxazine **2n** was prepared from bisallylic amide **1n** (21.0 mg, 0.060 mmol, 1.00 equiv.) by the General Procedure C using [(DHQ)$_2$PHAL] instead of [(DHQD)$_2$PHAL] as the catalyst. The diastereomeric ratio of the crude mixture was determined to be 4.4:1 by ^{1}H-NMR. The crude product was purified by flash column chromatography on silica gel (eluent: 10% ethyl acetate in hexanes) to give **2n** (6.1 mg, 26%) as a pale-yellow oil.

TLC (30% ethyl acetate in hexanes), R*f*: 0.77 (UV).

^{1}H NMR (500 MHz, $CDCl_3$): δ 8.08 – 8.02 (m, 2H), 7.51 –7.38 (m, 3H), 7.36 (dd, *J* = 5.0, 1.2 Hz, 1H), 7.16 (dt, *J* = 5.2, 0.9 Hz, 1H), 7.12 (dt, J = 3.6, 1.1 Hz, 1H), 7.03 (dd, *J* = 5.0, 3.6 Hz, 1H), 7.00 – 6.92 (m, 2H), 6.86 – 6.78 (m, 1H), 6.29 (dd, *J* = 15.6, 5.3 Hz, 1H), 5.65 (dd, *J* = 6.6, 0.9 Hz, 1H), 4.61 (ddd, *J* = 5.6, 4.3, 1.7 Hz, 1H), 4.40 (dd, *J* = 6.6, 4.3 Hz, 1H). ^{13}C NMR (126 MHz, $CDCl_3$): δ 154.1, 141.8, 140.5, 132.4, 131.2, 128.2, 127.6, 127.4, 127.1, 126.8, 126.2, 126.2, 126.1, 126.1, 124.5, 75.4, 58.1, 55.2. IR (cm $^{-1}$): 3150, 1618, 1130, 1008, 706. HRMS (ESI) (m/z): $C_{20}H_{17}NOS_2Cl$ 386.0440; found:386.0435. HPLC: Daicel Chiralpak AD-H, 5% IPA-Hex, 1 mL/min; 254 nm, $RT_{1(major)}$ = 12.6 min, $RT_{2(minor)}$ = 14.2 min.

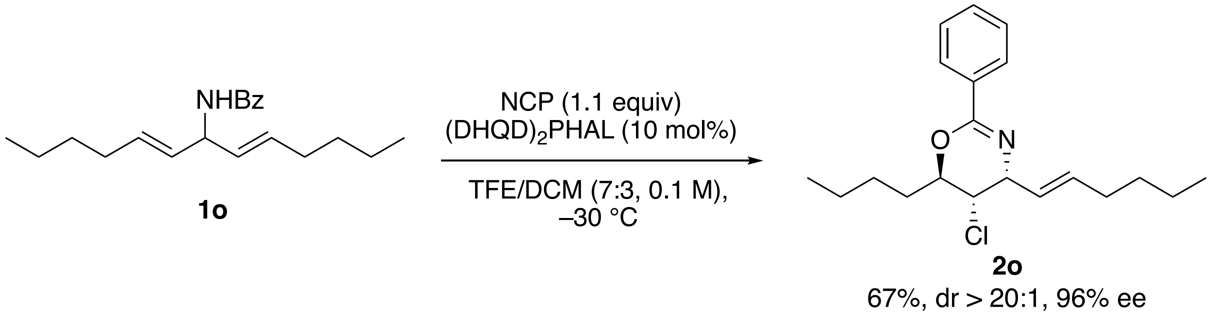

(4*R*,5*S*,6*R*)-6-butyl-5-chloro-4-((*E*)-hex-1-en-1-yl)-2-phenyl-5,6-dihydro-4*H*-1,3-oxazine **2o**:

Oxazine **2o** was prepared from bisallylic amide **1o** (30.0 mg, 0.10 mmol, 1.00 equiv.) by General Procedure C. The diastereomeric ratio of the crude mixture was determined to be >20:1 by ^{1}H-NMR. The crude product was purified by flash column chromatography on silica gel (eluent: 10% ethyl acetate in hexanes) to give **2o** (22.3 mg, 67%) as an oily liquid.

TLC (15% ethyl acetate in hexanes), R*f*: 0.64 (UV).

^{1}H NMR (500 MHz, $CDCl_3$): δ 7.99 – 7.90 (m, 2H), 7.51 – 7.30 (m, 3H), 5.80 – 5.55 (m, 2H), 4.38 – 4.35 (m, 1H), 4.28 (td, *J* = 8.3, 3.2 Hz, 1H), 4.04 (dd, *J* = 7.9, 4.6 Hz, 1H), 2.13 – 2.05 (m, 2H), 1.96 – 1.86 (m, 1H), 1.76 – 1.56 (m, 2H), 1.51 – 1.22 (m, 7H), 0.94 (t, *J* = 7.2 Hz, 3H), 0.87 (t, *J* = 7.2 Hz, 3H). ^{13}C NMR (126 MHz, $CDCl_3$): δ 154.2, 135.4, 133.0, 130.7, 128.0, 127.4, 126.7, 76.1, 57.3, 56.5, 32.6, 32.2, 31.2, 26.8, 22.5, 22.2, 14.0, 13.9. IR (cm $^{-1}$): 2910, 1642, 1506, 1370, 856. HRMS (ESI) (m/z): calculated for $(M+H)^+$: $C_{20}H_{29}ClNO$ 334.1939; found: 334.1942. $[\alpha]_D^{20}$: +83.1° (C 1.0, CH_2Cl_2, *ee* = 96%). HPLC: Daicel Chiralpak IA, 1% IPA-Hex, 1 mL/min; 254 nm, $RT_{1(minor)}$ = 3.9 min, $RT_{2(major)}$ = 4.7 min.

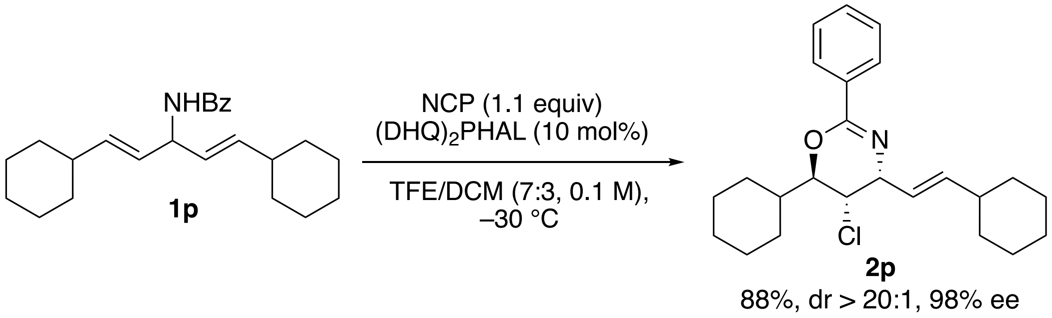

(4*R*,5*S*,6*R*)-5-chloro-6-cyclohexyl-4-((*E*)-2-cyclohexylvinyl)-2-phenyl-5,6-dihydro-4*H*-1,3-oxazine **2p**:

Oxazine **2p** was prepared from bisallylic amide **1p** (28.0 mg, 0.080 mmol, 1.00 equiv.) by General Procedure C using [(DHQ)$_2$PHAL] instead of [(DHQD)$_2$PHAL] as the catalyst. The diastereomeric ratio of the crude mixture was determined to be >20:1 by ^{1}H-NMR. The crude product was purified by flash column chromatography on silica gel (eluent: 10% ethyl acetate in hexanes) to give **2p** (27.2 mg, 88%) as a colorless oil.

TLC (30% ethyl acetate in hexanes), R*f*: 0.89 (UV).

^{1}H NMR (500 MHz, CDCl$_3$): δ 8.03 – 7.85 (m, 2H), 7.51 – 7.30 (m, 3H), 5.61 (qd, *J* = 15.5, 5.7 Hz, 2H), 4.35 (t, *J* = 5.0 Hz, 1H), 4.22 (dd, *J* = 7.9, 4.6 Hz, 1H), 4.08 (dd, *J* = 7.9, 4.4 Hz, 1H), 2.02 (dq, *J* = 5.0, 2.7, 2.0 Hz, 1H), 1.89 – 1.49 (m, 11H), 1.44 (qd, *J* = 12.5, 3.5 Hz, 1H), 1.36 – 1.04 (m, 9H). ^{13}C NMR (126 MHz, CDCl$_3$): δ 154.4, 141.0, 133.0, 130.7, 128.1, 127.4, 124.5, 79.7, 77.2, 56.5, 54.9, 40.6, 38.9, 32.9, 32.8, 29.4, 26.3, 26.3, 26.2, 26.2, 26.0, 25.8. IR (cm $^{-1}$): 2930, 1640, 1516, 1310, 816. HRMS (ESI) (m/z): calculated for $(M+H)^+$: $C_{24}H_{33}ClNO$ 386.2251; found: 386.2263. $[\alpha]_D^{20}$: +91° (C 1.0, CH_2Cl_2). HPLC: Daicel Chiralpak IA, 1% IPA-Hex, 1 mL/min; 254 nm, $RT_{1(major)}$ = 5.1 min, $RT_{2(minor)}$ = 11.7 min.

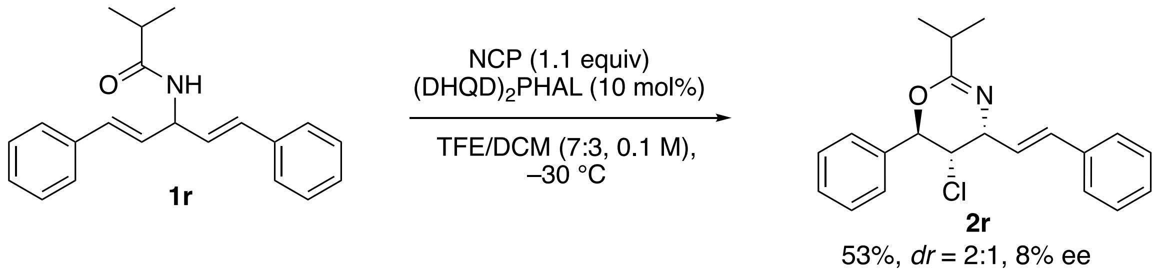

(4*R*,5*S*,6*R*)-5-chloro-2-isopropyl-6-phenyl-4-((*E*)-styryl)-5,6-dihydro-4*H*-1,3-oxazine **2r**:

Oxazine **2r** was prepared from bisallylic amide **1r** (30.5 mg, 0.10 mmol, 1.00 equiv.) by General Procedure C. The diastereomeric ratio of the crude mixture was determined to be 2:1 by ^{1}H-NMR. The crude product was purified by flash column chromatography on silica gel (eluent: 10% ethyl acetate in hexanes) to give **2r** (18 mg, 53%) as a colorless oil.

TLC (30% ethyl acetate in hexanes), R*f*: 0.74 (UV).

^{1}H NMR (500 MHz, $CDCl_3$): δ 7.39 (m, 5H), 7.33 – 7.26 (m, 4H), 7.23 – 7.20 (m, 1H), 6.60 – 6.53 (m, 1H), 6.39 – 6.30 (m, 1H), 5.23 (d, *J* = 5.8 Hz, 1H), 4.26 (m, 2H), 2.67 (p, *J* = 6.9 Hz, 1H), 1.29 (dd, *J* = 6.9, 2.9 Hz, 6H). ^{13}C NMR (126 MHz, $CDCl_3$): δ 163.6, 138.0, 136.8, 133.1, 128.8, 128.8, 128.5, 127.6, 127.3, 126.6, 126.2, 78.9, 58.5, 54.0, 34.5, 20.1, 20.0. IR (cm $^{-1}$): 3110, 1632, 1556, 1370, 836. HRMS (ESI) (m/z): calculated for $C_{21}H_{21}ClNO$ 338.1312; found: 338.1298. HPLC: Daicel Chiralpak IA, 15% IPA-Hex, 1 mL/min; 254 nm, $RT_{1(minor)}$ = 5.3 min, $RT_{2(major)}$ = 7.5 min.

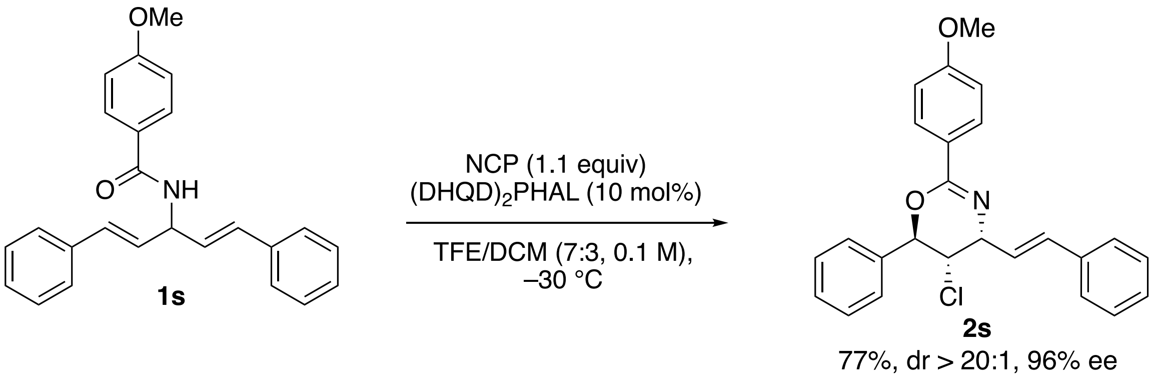

(4*R*,5*S*,6*R*)-5-chloro-2-(4-methoxyphenyl)-6-phenyl-4-((*E*)-styryl)-5,6-dihydro-4*H*-1,3-oxazine **2s**:

Oxazine **2o** was prepared from bisallylic amide **1s** (37.0 mg, 0.10 mmol, 1.00 equiv.) by General Procedure C. The diastereomeric ratio of the crude mixture was determined to be >20:1 by ^{1}H-NMR. The crude product was purified by flash column chromatography on silica gel (eluent: 10% ethyl acetate in hexanes) to give **2s** (31.1 mg, mp 148-152 °C, 77%) as a off-white solid.

TLC (30% ethyl acetate in hexanes), R*f*: 0.77 (UV).

^{1}H NMR (500 MHz, $CDCl_3$): δ 8.08 – 8.02 (m, 2H), 7.45 – 7.28 (m, 10H), 7.26 – 7.20 (m, 1H), 6.95 – 6.89 (m, 2H), 6.68 (dd, *J* = 15.8, 1.5 Hz, 1H), 6.43 (dd, *J* = 15.8, 5.6 Hz, 1H), 5.44 (d, *J* = 6.1 Hz, 1H), 4.48 (ddd, *J* = 5.6, 4.1, 1.6 Hz, 1H), 4.39 (dd, *J* = 6.2, 4.1 Hz, 1H), 3.85 (s, 3H). ^{13}C NMR (126 MHz, $CDCl_3$): δ 162.0, 154.2, 137.8, 136.8, 133.3, 129.3, 128.9, 128.8, 128.5, 127.6, 127.3, 126.7, 126.3, 125.0, 113.5, 79.0, 58.4, 55.4, 54.8. IR (cm $^{-1}$): 3070, 1670, 1560, 1516, 1309, 1226, 1100, 1048, 681. HRMS (ESI) (m/z): calculated for $(M+H)^+$: $C_{25}H_{23}ClNO_2$ 404.1417; found: 404.1457. $[\alpha]_D^{20}$: +2.8° (C 1.0, CH_2Cl_2, *ee* = 96%). HPLC: Daicel Chiralpak IA, 15% IPA-Hex, 1 mL/min; 254 nm, $RT_{1(minor)}$ = 16.3 min, $RT_{2(major)}$ = 19.7 min.

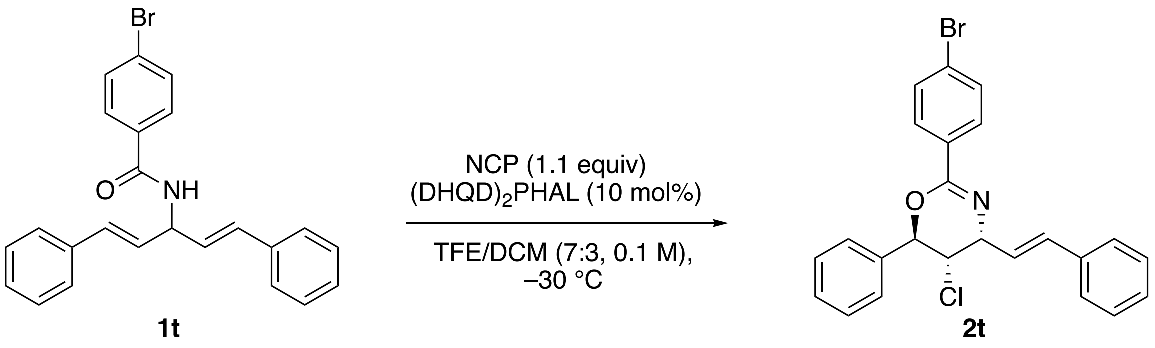

66%, dr > 20:1, 98% ee

(4*R*,5*S*,6*R*)-2-(4-bromophenyl)-5-chloro-6-phenyl-4-((*E*)-styryl)-5,6-dihydro-4*H*-1,3-oxazine **2t**:

Oxazine **2t** was prepared from bisallylic amide **1t** (21.0 mg, 0.050 mmol, 1.00 equiv.) by General Procedure C. The diastereomeric ratio of the crude mixture was determined to be >20:1 by ^{1}H-NMR. The crude product was purified by flash column chromatography on silica gel (eluent: 10% ethyl acetate in hexanes) to give **2t** (15.0 mg, mp 128-134 ºC, 66%) as a white solid.

TLC (30% ethyl acetate in hexanes), R*f*: 0.74 (UV).

^{1}H NMR (500 MHz, $CDCl_3$): δ 7.96 (d, *J* = 8.6 Hz, 2H), 7.55 (d, *J* = 8.6 Hz, 2H), 7.45 – 7.37 (m, 5H), 7.34 – 7.26 (m, 4H), 7.27 – 7.19 (m, 1H), 6.66 (dd, *J* = 15.8, 1.4 Hz, 1H), 6.40 (dd, *J* = 15.8, 5.6 Hz, 1H), 5.46 (d, *J* = 5.8 Hz, 1H), 4.47 (ddd, *J* = 5.6, 4.0, 1.5 Hz, 1H), 4.39 (dd, *J* = 5.9, 4.0 Hz, 1H). ^{13}C NMR (126 MHz, $CDCl_3$): δ 153.6, 137.6, 136.6, 133.4, 131.5, 131.5, 129.2, 129.0, 128.9, 128.6, 127.8, 126.9, 126.7, 126.1, 125.9, 79.4, 58.1, 54.8. IR (cm $^{-1}$): 3110, 1640, 1316, 1230, 945, 676. HRMS (ESI) (m/z): calculated for $(M+H)^+$: $C_{24}H_{20}ClBrNO$ 452.0417; found: 452.0415. $[\alpha]_D^{20}$: +4.8° (C 1.0, CH_2Cl_2, *ee* = 98%). HPLC: Daicel Chiralpak IA, 5% IPA-Hex, 1 mL/min; 254 nm, $RT_{1(minor)}$ = 12.9 min, $RT_{2(major)}$ = 17.3 min.

5. General Method for Growing Crystals:

Illustrated for compound **2a** & **4**: In a 4 mL vial was added compound **2a/4** (15 - 20 mg) followed by the addition of CH_2Cl_2 (0.5 mL). Once the compound was fully dissolved in CH_2Cl_2, hexane (~3 mL) was slowly added (the layer between CH_2Cl_2 and hexane should not be disturbed). The 4 mL vial was placed inside another 20 mL vial, caped loosely. The crystal growth was noticed after 24 hours, and they were collected after 48 hours via filtration through a Hirsch funnel.

Crystal Growth technique for compound **5**: In a 4 mL vial was added compound **5** (10 mg) followed by the addition of Me_2CO (0.5 mL). Once the compound was fully dissolved in Me_2CO; the 4 mL vial was placed inside another 20 mL vial, where upon Et_2O (~10 mL) was slowly added outside the 4ml vial and caped loosely. The crystal growth was noticed after 24 hours, and they were collected after 48 hours via filtration through a Hirsch funnel.

6. General Method for Gram-Scale Reaction:

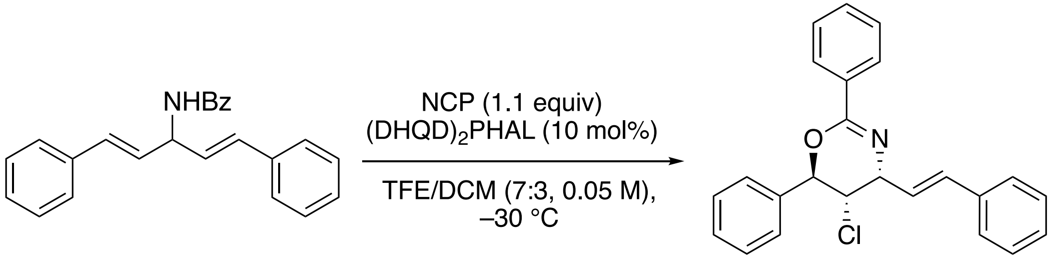

1a (1.0 g, 2.95 mmol)

2a

82%, dr > 20:1, 96% ee

Compound **2a** was prepared from bisallylic amide **1a** (1.0 g, 2.95 mmol, 1.0 equiv) following General Procedure C, where higher yield was obtained following a slight dilution to 0.05M from the original reaction condition. The diastereomeric ratio of the crude mixture was determined to be >20:1 by ^{1}H-NMR. The crude product was purified by column chromatography on silica gel (eluent: 10% ethyl acetate in hexanes) to give **2a** (903.2 mg, 82%) as a yellow solid with slight erosion in *ee* (96%).

7. Comparison of Two Solvent Systems

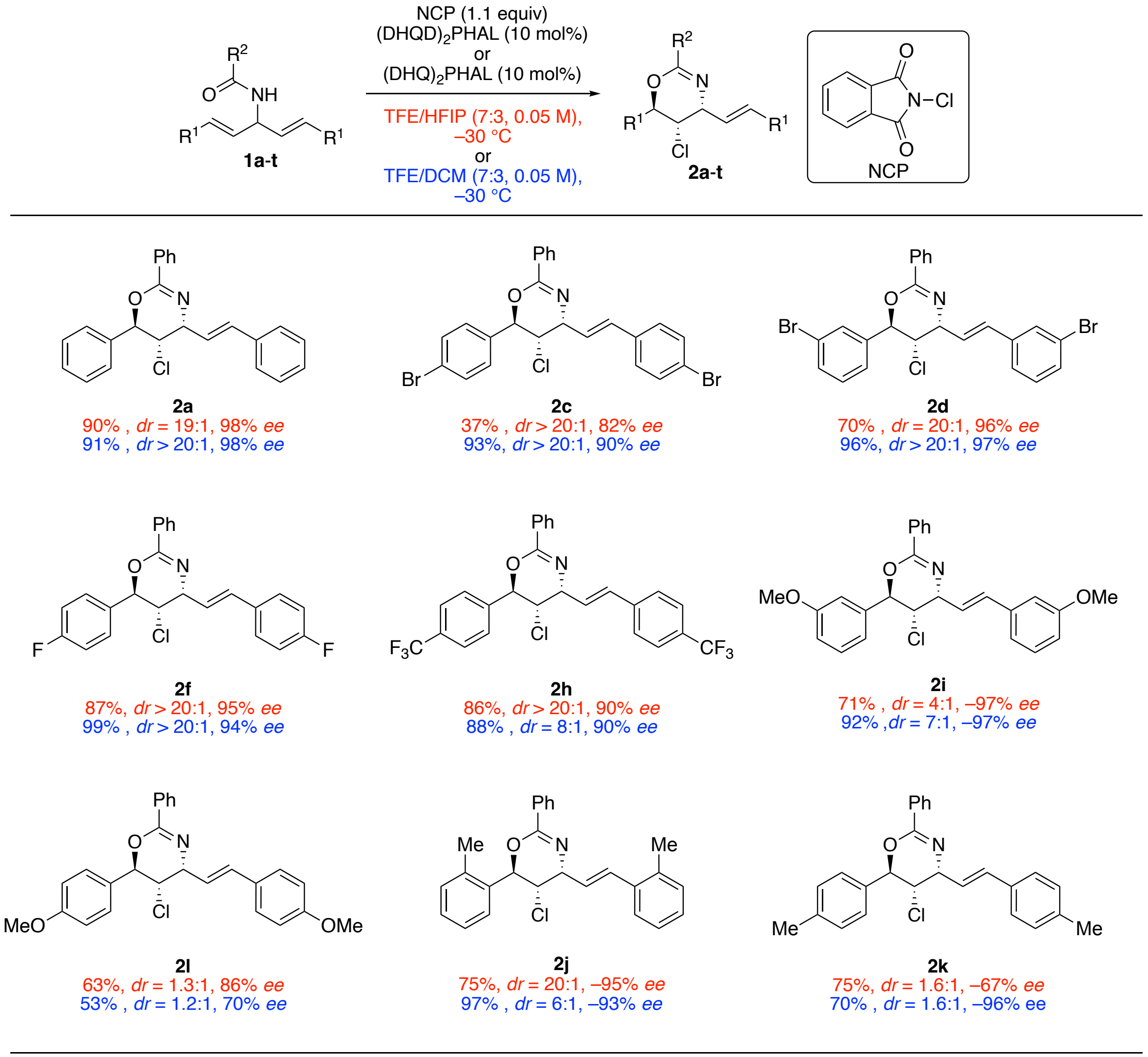

Scheme S1 Comparison of Two Solvent Systems

8. Derivatization of Oxazine Product

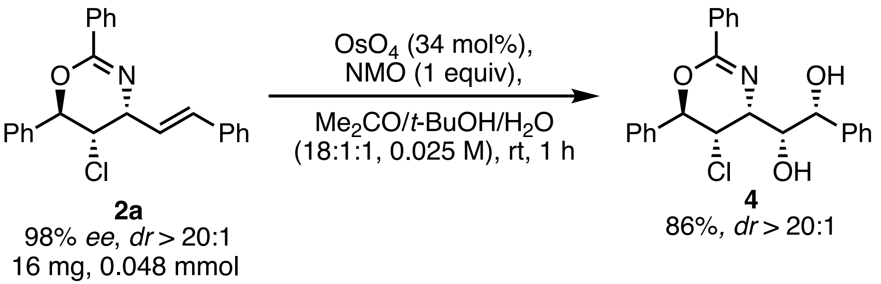

(1*R*,2*R*)-1-((4*R*,5*S*,6*R*)-5-chloro-2,6-diphenyl-5,6-dihydro-4*H*-1,3-oxazin-4-yl)-2-phenylethane-1,2-diol **4**:

Oxazine substrate **2a** (16 mg, 0.048 mmol, 1.0 equiv) was added to a 20 mL scintillation vial followed by the sequential addition of acetone (1.8 mL), *tert*-butyl alcohol (0.1 mL) and water (0.1 mL). Osmium tetroxide (4.2 mg, 0.0165 mmol, 0.34 equiv) and *N*-methylmorpholine-*N*-oxide (6.2 mg, 0.053mmol, 1.1 equiv) were subsequently added. The reaction mixture was stirred at room temperature until full consumption of the oxazine. The reaction mixture was then concentrated under reduced pressure to provide the crude product **4** which was purified by flash column chromatography on silica gel (eluent: 10% ethyl acetate in hexanes) to generate the oxazine-diol product (16.8 mg, mp 142 - 150 °C, 86%) as a brown solid. The absolute stereochemistry was determined by X-ray crystallography.

TLC (30% ethyl acetate in hexanes), R*f*: 0.51 (UV).

^{1}H NMR (500 MHz, $CDCl_3$): δ 8.16 – 8.11 (m, 2H), 7.58 – 7.51 (m, 1H), 7.48 (dd, *J* = 8.3, 6.8 Hz, 2H), 7.43 (d, *J* = 7.6 Hz, 2H), 7.39 – 7.31 (m, 5H), 7.30 – 7.25 (m, 1H), 7.24 – 7.20 (m, 2H), 5.78 (s, 1H), 5.44 (s, 1H), 4.74 – 4.66 (m, 1H), 4.03 (ddd, *J* = 9.0, 6.7, 2.0 Hz, 1H), 3.73 (s, 1H), 3.64 (dd, *J* = 9.2, 3.0 Hz, 1H), 2.03 – 1.92 (br, 1H). ^{13}C NMR (126 MHz, $CDCl_3$): δ 154.1, 141.6, 138.3, 132.3, 131.3, 129.1, 128.7, 128.4, 128.3, 127.5, 126.3, 124.8, 81.4, 74.6, 72.7, 56.2, 51.8. HRMS (ESI) (m/z): calculated for $(M+H)^+$: $C_{24}H_{23}ClNO_3$ 408.1366; found: 408.1391.

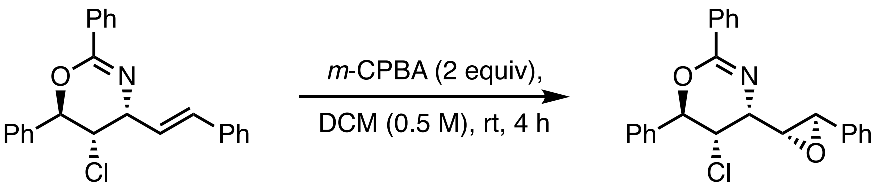

(4*R*,5*S*,6*R*)-5-chloro-2,6-diphenyl-4-((2*R*,3*R*)-3-phenyloxiran-2-yl)-5,6-dihydro-4*H*-1,3-oxazine **5**:

2a (38 mg, 0.10 mmol, 1.0 equiv) was dissolved in dichloromethane (2 mL) in a 20 mL scintillation vial. *meta*-chloroperoxybenzoic acid (44 mg, 77% wt, 0.20 mmol, 2.0 equiv) was then added to the solution. The reaction mixture was stirred at room temperature until the complete consumption of oxazine (ca. 4 h) was observed through TLC. The reaction was then quenched by the addition of water (1 mL). The mixture was extracted with dichloromethane (3 × 5 mL) and the organic layers were combined, dried over anhydrous sodium sulfate, filtered and concentrated under reduced pressure to provide crude product. The crude product was purified by flash column chromatography on silica gel (eluent: 10% ethyl acetate in hexanes) to provide **5** (26 mg, 67%, mp: 98 - 102 °C) as a colorless waxy solid. The absolute stereochemistry was determined by X-ray crystallography.

TLC (30% ethyl acetate in hexanes), R*f*: 0.78 (UV).

^{1}H NMR (500 MHz, $CDCl_3$): δ 8.17 – 8.07 (m, 2H), 7.55 – 7.31 (m, 11H), 7.30 – 7.25 (m, 2H), 5.75 (d, *J* = 2.1 Hz, 1H), 4.61 (dd, *J* = 3.2, 2.2 Hz, 1H), 3.99 (d, *J* = 1.9 Hz, 1H), 3.58 (dd, *J* = 5.5, 3.1 Hz, 1H), 3.29 (dd, *J* = 5.5, 2.0 Hz, 1H). ^{13}C NMR (126 MHz, $CDCl_3$): δ 154.3, 138.1, 136.9, 132.4, 131.3, 129.2, 128.9, 128.4, 128.2, 128.2, 127.6, 125.8, 124.9, 80.5, 62.7, 57.9, 55.7, 51.9. HRMS (ESI) (m/z): calculated for $(M+H)^+$: $C_{24}H_{21}ClNO_2$ 390.1261; found: 390.1273.

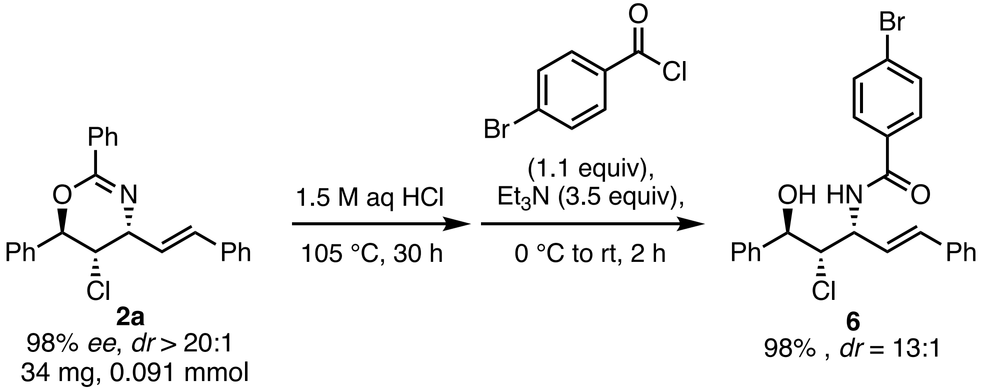

4-bromo-*N*-((3*R*,4*S*,5*R*,*E*)-4-chloro-5-hydroxy-1,5-diphenylpent-1-en-3-yl)benzamide **6** (modified from a known procedure [7, 8]):

Enantioenriched chloro-oxazine substrate **2a** (34 mg, 0.091 mmol, 1.0 equiv.) was added to a 5 mL seal tube. An aqueous solution of hydrochloric acid (1.5 M, 1.0 mL, 16 equiv.) was added into the tube in one portion. The tube was sealed and heated in an oil bath preheated to 105 ºC for 30 h. The reaction mixture was then concentrated under reduced pressure to provide crude hydrochloride salt of the amine. This was used directly for next step without further purification.

The crude amine hydrochloride salt was transferred a 20 mL scintillation vial using anhydrous tetrahydrofuran (1 mL). The solution was cooled to 0 ºC in an ice-water bath. After 5 min, triethylamine (32 mg, 0.32 mmol, 3.5 equiv.) and *p*-bromobenzoyl chloride (22 mg, 0.10 mmol, 1.1 equiv.) were added sequentially. The reaction mixture was then slowly warmed to room temperature where it was stirred for 2 h. The reaction mixture was poured into water and the organic layer was extracted with dichloromethane (3 × 5 mL). The combined organic layers were dried over anhydrous sodium sulphate, filtered, and concentrated under reduced pressure to provide the crude product. The crude product was purified by flash column chromatography on silica gel (eluent: 10% ethyl acetate in hexanes) to provide **6** (46 mg, 98%) as a colorless oil.

TLC (30% ethyl acetate in hexanes), R*f*: 0.65 (UV).

^{1}H NMR (500 MHz, $CDCl_3$): δ 8.15 – 8.09 (m, 2H), 7.54 – 7.48 (m, 1H), 7.48 – 7.40 (m, 6H), 7.37 (dd, *J* = 8.0, 1.8 Hz, 2H), 7.33 (t, *J* = 7.6 Hz, 2H), 7.28 – 7.24 (m, 1H), 6.71 (dd, *J* = 15.9, 1.5 Hz, 1H), 6.46 (dd, *J* = 15.8, 5.5 Hz, 1H), 5.49 (d, *J* = 6.0 Hz, 1H), 4.53 (ddd, *J* = 5.6, 4.1, 1.5 Hz, 1H), 4.42 (dd, *J* = 6.1, 4.1 Hz, 1H). ^{13}C NMR (126 MHz, $CDCl_3$) δ 154.6, 137.9, 136.9, 133.5, 132.7, 131.3, 129.1, 129.1, 128.7, 128.4, 127.8, 127.7, 127.2, 126.8, 126.4, 79.3, 58.4, 54.9. HRMS (ESI) (m/z): calculated for $(M\text{–}H)^{-}$: $C_{24}H_{20}NO_2ClBr$ 468.0364; found: 468.0366.

9. X-Ray Crystal Structures

9.1 X-ray crystal structure for 2a

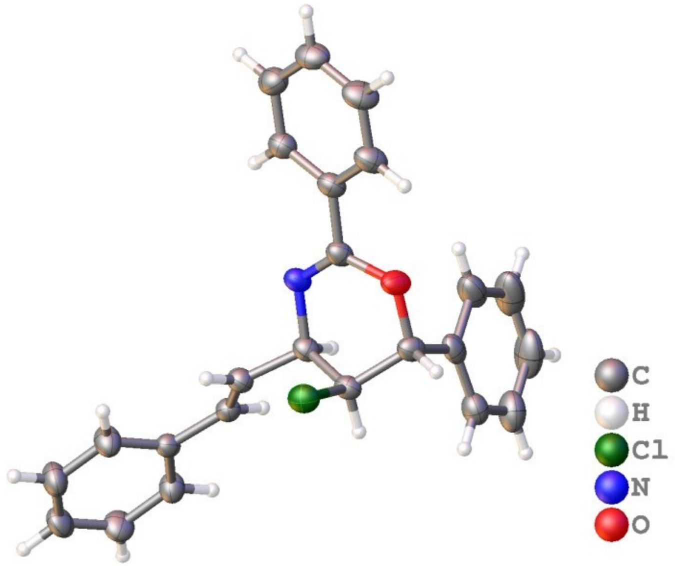

Experimental. Single colourless chunk-shaped crystals of **2a** used as received. A suitable crystal with dimensions 0.30 × 0.22 × 0.12 mm^3 was selected and mounted on a nylon loop with paratone oil on a Bruker APEX-II CCD diffractometer. The crystal was kept at a steady T = 173(2) K during data collection. The structure was solved with the XT (Sheldrick, 2015) solution program using dual methods and by using Olex2 1.5 as the graphical interface. [9-12] The model was refined with XL (Sheldrick, 2008) using full matrix least squares minimisation on $\boldsymbol{F^2}$.

Crystal Data. $C_{24}H_{20}ClNO$, M_r = 373.86, orthorhombic, P212121 (No. 19), a = 9.3060(8) Å, b =9.6574(9) Å, c = 21.8911(19) Å, $\alpha = \beta = \gamma = 90^\circ$, V = 1967.4(3) $Å^3$, T = 173(2) K, Z = 4, Z'= 1, μ(MoKα) = 0.207, 14580 reflections measured, 3615 unique (R_{int} = 0.0335) which were used in all calculations. The final $wR2$ was 0.0784 (all data) and $R1$ was 0.0332 (I≥2 σ(I)).

Structure Quality Indicators

Reflections:

d min (MoKα) 2Θ=50.8°	I/σ(I)	Rint m=4.05	Full 50.5°
0.83	33.4	3.35%	100

Refinement:

Shift	Max Peak	Min Peak	GooF	Hooft
0.000	0.2	-0.2	1.059	.02(3)

**Compound**	**2a**
Formula	$C_{24}H_{20}ClNO$
CCDC	2337924
$D_{calc.}$/ g cm^{-3}	1.262
μ/mm^{-1}	0.207
Formula Weight	373.86
Colour	colourless
Shape	chunk-shaped
Size/mm^3	0.30×0.22×0.12
T/K	173(2)
Crystal System	orthorhombic
Flack Parameter	0.02(3)
Hooft Parameter	0.04(3)
Space Group	$P2_12_12_1$
a/Å	9.3060(8)
b/Å	9.6574(9)
c/Å	21.8911(19)
α/°	90
β/°	90
γ/°	90
V/Å^3	1967.4(3)
Z	4
Z'	1
Wavelength/Å	0.71073
Radiation type	MoK$_α$
$Θ_{min}$/°	1.861
$Θ_{max}$/°	25.393
Measured Refl's.	14580
Indep't Refl's	3615
Refl's I≥2 σ(I)	3255
R_{int}	0.0335
Parameters	244
Restraints	0
Largest Peak	0.161
Deepest Hole	-0.165
GooF	1.059
wR_2 (all data)	0.0784
wR_2	0.0749
R_1 (all data)	0.0384
R_1	0.0332

The Model has Chirality at C1 (Chiral SPGR) R Verify; The Model has Chirality at C2 (Chiral SPGR) S Verify; The Model has Chirality at C3 (Chiral SPGR) R Verify.

A colourless chunk-shaped crystal with dimensions 0.30×0.22×0.12 mm^3 was mounted on a nylon loop with paratone oil. Data were collected using a Bruker APEX-II CCD diffractometer equipped withan Oxford Cryosystems 800 low-temperature device, operating at T = 173(2) K.

MSU Data were measured using ϕ and ω scans using MoKα radiation. The total number of runs and images was based on the strategy calculation from the program COSMO (BRUKER, V1.61, 2009). The achieved resolution was Θ = 25.393.

Cell parameters were retrieved using the SAINT v8.34A (Bruker, 2013) software and refined using SAINT v8.34A (Bruker, 2013) on 7960 reflections, 55 % of the observed reflections. Data reduction was performed using the SAINT v8.34A (Bruker, 2013) software which corrects for Lorentz polarization. The final completeness is 100.00 out to 25.393 in Θ SADABS-2014/5 (Bruker, 2014/5) was used for absorption correction. *wR2*(int) was 0.0549 before and 0.0509 after correction. The Ratio of minimum to maximum transmission is 0.9063. The λ/2 correction factor is 0.00150.

The structure was solved in the space group *P*212121 (# 19) by using dual methods using the XT (Sheldrick, 2015) structure solution program. The structure was refined by Least Squares using version 2014/6 of XL incorporated in Olex2. [9, 10] All non-hydrogen atoms were refined anisotropically. Hydrogen atom positions were calculated geometrically and refined using the riding model.

CCDC 2337924 contains the supplementary crystallographic data for 2a. The data can be obtained free of charge from The Cambridge Crystallographic Data Centre via www.ccdc.cam.ac.uk/structures.

_refine_special_details: The structure was refined by Least Squares using version 2014/6 of XL incorporated in Olex2. [9, 10] All non-hydrogen atoms were refined anisotropically. Hydrogen atom positions were calculated geometrically and refined using theriding model.

_exptl_absorpt_process_details: SADABS-2014/5 (Bruker,2014/5) was used for absorption correction. *wR2*(int) was 0.0549 before and 0.0509 after correction. The Ratio of minimum to maximum transmission is 0.9063. The λ/2 correction factor is 0.00150.

There is a single formula unit in the asymmetric unit, which is represented by the reported sum formula. In other words: Z is 4 and Z' is 1. The

moiety formula is $C_{24}H_{20}ClNO$.

The Flack parameter was refined to 0.02(3). Determination of absolute structure using Bayesian statistics on Bijvoet differences using the Olex2 results in 0.04(3). The chiral atoms in this structure are: C1(R), C2(S), C3(R). Note: The Flack parameter is used to determine chirality of the

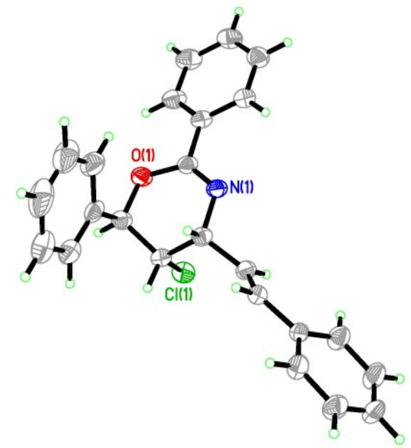

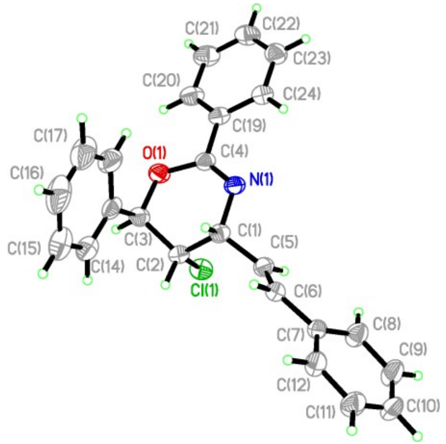

crystal studied, the value should be near 0, a value of 1 means that the stereochemistry is wrong and the model should be inverted. A value of 0.5 means that the crystal consists of a racemic mixture of the two enantiomers.

The Model has Chirality at C1 (Chiral SPGR) R Verify; The Model has Chirality at C2 (Chiral SPGR)S Verify; The Model has Chirality at C3 (Chiral SPGR) R Verify.

Figure 3 Packing diagram of **2a**

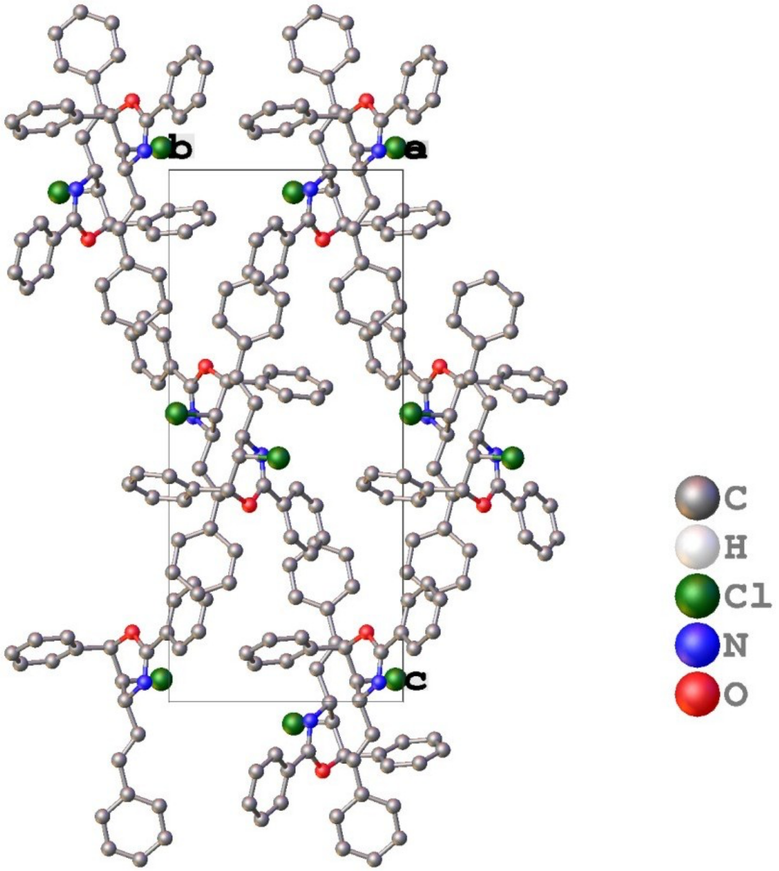

Data Plots: Diffraction Data

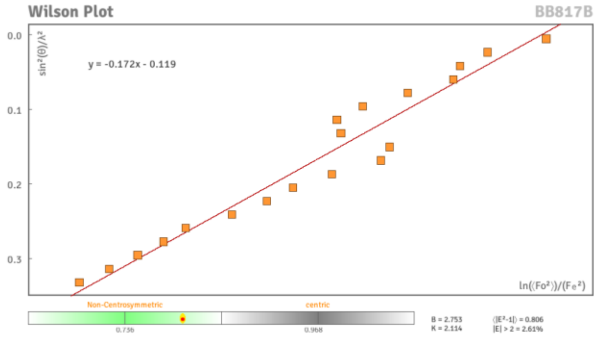

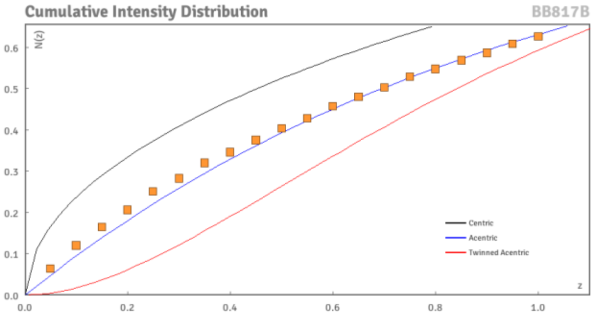

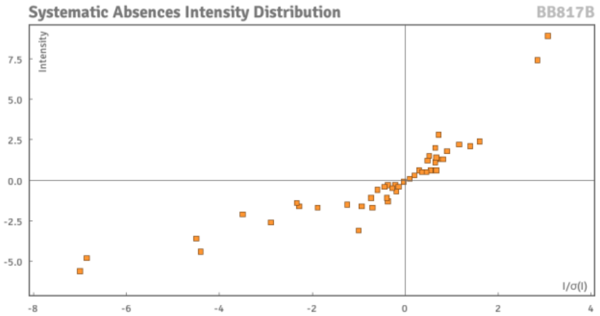

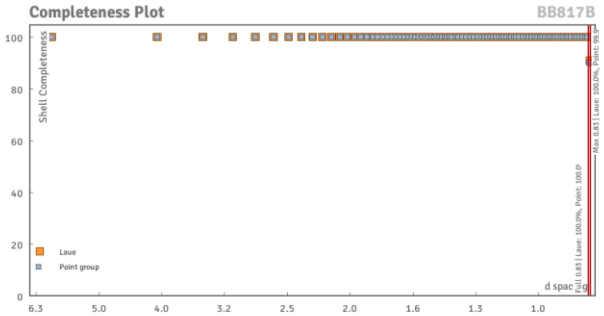

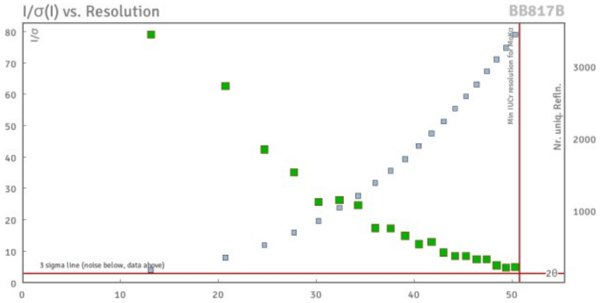

Data Plots: Refinement and Data

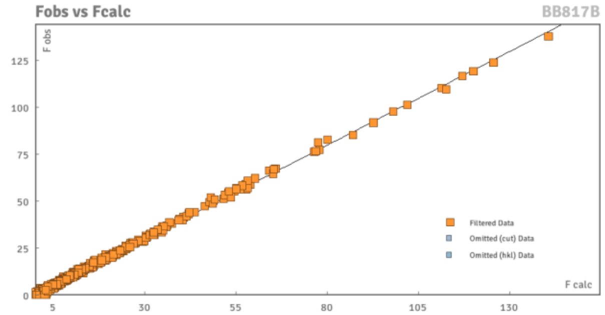

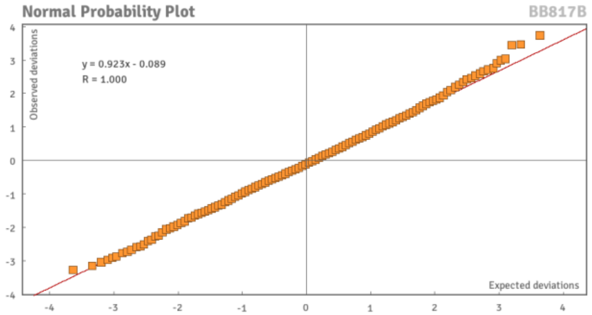

Reflection Statistics

Total reflections (after filtering)	14633	Unique reflections	3615
Completeness	0.999	Mean I/σ	21.38
hklmax collected	(11, 11, 26)	hklmin collected	(-11, -11, -26)
hklmax used	(11, 11, 26)	hklmin used	(-11, 0, 0)
Lim dmax collected	100.0	Lim dmin collected	0.36
dmax used	10.95	dmin used	0.83
Friedel pairs	5578	Friedel pairs merged	0
Inconsistent equivalents	0	Rint	0.0335
Rsigma	0.0299	Intensity transformed	0
Omitted reflections	0	Omitted by user (OMIT hkl)	0
Multiplicity	(10859, 1842, 30)	Maximum multiplicity	7
Removed systematic	53	Filtered off (Shel/OMIT)	0

Table 1: Fractional Atomic Coordinates (×10^4) and Equivalent Isotropic Displacement Parameters (Å^2×10^3) for **2a**. *Ueq* is defined as 1/3 of the trace of the orthogonalised *Uij*.

Atom	x	y	z	*Ueq*
Cl1	8331.8(7)	9671.3(7)	4581.2(3)	37.46(18)
O1	6057.4(19)	8436(2)	3695.7(8)	37.4(5)
N1	4968(2)	8961(2)	4640.0(10)	30.6(5)
C1	6029(3)	8141(3)	4975.4(11)	29.6(6)
C2	7425(3)	8021(2)	4621.2(11)	29.1(5)
C3	7078(3)	7515(3)	3979.6(11)	32.5(6)
C4	5057(3)	9051(3)	4065.5(12)	30.7(6)
C5	6203(3)	8684(3)	5614.9(11)	30.2(6)
C6	6068(3)	7873(3)	6100.0(11)	28.9(6)
C7	6286(2)	8234(3)	6747.3(11)	27.9(5)
C8	6358(3)	9584(3)	6965.1(12)	40.4(7)
C9	6660(4)	9835(3)	7570.2(12)	46.0(7)
C10	6882(3)	8766(3)	7971.9(12)	41.7(7)
C11	6779(3)	7431(3)	7766.8(12)	42.9(7)
C12	6471(3)	7167(3)	7161.8(11)	36.7(6)
C13	6543(3)	6037(3)	3998.4(11)	35.0(6)
C14	7432(3)	5023(3)	4237.7(12)	41.0(7)
C15	6962(4)	3665(3)	4289.1(14)	54.0(9)
C16	5609(4)	3314(4)	4091.0(18)	67.8(11)
C17	4733(4)	4302(4)	3839.9(19)	70.6(12)
C18	5185(3)	5663(4)	3793.6(15)	51.5(8)
C19	4039(3)	9915(3)	3708.8(11)	32.2(6)
C20	4268(3)	10220(3)	3096.5(12)	41.3(7)
C21	3298(3)	11056(3)	2784.5(13)	45.7(7)
C22	2100(3)	11569(3)	3075.4(14)	42.8(7)
C23	1874(3)	11272(3)	3683.7(13)	40.5(7)
C24	2837(3)	10456(3)	4001.1(12)	34.6(6)

Table 2: Anisotropic Displacement Parameters (×10^4) for **2a**. The anisotropic displacement factor exponent takes the form: $-2\pi^2[h^2a^{*2} \times U11 + ... + 2hka^* \times b^* \times U12]$

**Atom**	***U11***	***U22***	***U33***	***U23***	***U13***	***U12***
Cl1	33.6(3)	35.5(3)	43.3(4)	4.2(3)	-0.8(3)	-5.7(3)
O1	33.0(10)	55.8(13)	23.4(9)	3.8(9)	0.1(8)	8.4(9)
N1	26.1(11)	39.0(12)	26.9(12)	0.6(10)	-2.4(9)	-0.9(9)
C1	29.5(13)	33.2(13)	26.0(13)	2.4(11)	-1.6(11)	-2.2(11)
C2	28.4(12)	30.0(13)	29.0(13)	2.6(12)	-3.6(11)	-3.0(10)
C3	26.2(13)	43.5(16)	27.9(13)	2.5(12)	2.0(10)	0.9(12)
C4	25.3(13)	40.0(15)	26.8(14)	0.0(12)	-0.5(10)	-3.6(11)
C5	30.1(14)	32.6(14)	27.9(13)	0.0(11)	-2.4(10)	-0.2(11)
C6	26.9(12)	30.3(14)	29.6(13)	-1.7(11)	-1.6(11)	2.1(10)
C7	25.6(13)	33.6(13)	24.6(12)	-0.1(11)	0.4(10)	1.7(11)
C8	56.7(19)	32.6(14)	31.8(14)	0.4(12)	-3.1(12)	2.8(14)
C9	63(2)	39.3(16)	35.6(15)	-9.2(13)	-2.9(14)	0.7(16)
C10	46.6(17)	53.7(18)	24.9(14)	-5.1(13)	-2.4(12)	0.3(15)
C11	56.0(18)	44.2(17)	28.4(14)	7.2(13)	-0.9(14)	4.6(16)
C12	45.6(17)	33.2(15)	31.5(14)	-2.1(11)	2.5(13)	1.4(13)

Atom	*U11*	*U22*	*U33*	*U23*	*U13*	*U12*
C13	33.9(14)	47.8(16)	23.3(12)	-8.9(12)	4.5(11)	-6.0(13)
C14	44.4(16)	41.7(17)	37.0(15)	-7.2(12)	-0.1(13)	-4.6(13)
C15	70(2)	41.8(18)	49.6(19)	-12.4(15)	8.6(16)	-7.5(17)
C16	71(3)	52(2)	81(3)	-31(2)	22(2)	-25(2)
C17	45(2)	83(3)	85(3)	-45(2)	12.1(19)	-25(2)
C18	36.2(15)	70(2)	47.8(19)	-20.5(16)	-0.1(13)	-7.3(16)
C19	29.4(13)	36.9(15)	30.4(13)	1.8(11)	-5.0(11)	-4.0(11)
C20	35.0(15)	55.8(18)	33.1(15)	5.3(14)	-3.6(12)	-4.4(14)
C21	46.4(16)	54.6(18)	36.3(15)	15.6(14)	-5.8(14)	-3.5(16)
C22	44.1(17)	35.4(16)	48.9(17)	10.2(13)	-11.2(14)	0.2(13)
C23	38.6(16)	35.5(15)	47.3(17)	-1.9(13)	-5.4(13)	2.5(13)
C24	33.7(14)	36.3(15)	33.8(14)	0.7(12)	-3.9(11)	-1.9(12)

Table 3: Bond Lengths in Å for **2a**.

Atom	Atom	Length/Å	Atom	Atom	Length/Å
Cl1	C2	1.806(2)	C9	C10	1.372(4)
O1	C3	1.442(3)	C10	C11	1.368(4)
O1	C4	1.369(3)	C11	C12	1.379(4)
N1	C1	1.464(3)	C13	C14	1.385(4)
N1	C4	1.263(3)	C13	C18	1.389(4)
C1	C2	1.517(3)	C14	C15	1.387(4)
C1	C5	1.504(3)	C15	C16	1.375(5)
C2	C3	1.521(3)	C16	C17	1.370(6)
C3	C13	1.512(4)	C17	C18	1.385(5)
C4	C19	1.485(4)	C19	C20	1.389(4)
C5	C6	1.325(3)	C19	C24	1.390(4)
C6	C7	1.473(3)	C20	C21	1.391(4)
C7	C8	1.390(4)	C21	C22	1.376(4)
C7	C12	1.384(3)	C22	C23	1.378(4)
C8	C9	1.376(4)	C23	C24	1.381(4)

Table 4: Bond Angles in ° for **2a**.

Atom	Atom	Atom	Angle/°	Atom	Atom	Atom	Angle/°
C4	O1	C3	117.42(18)	C9	C8	C7	120.3(3)
C4	N1	C1	119.5(2)	C10	C9	C8	121.0(3)
N1	C1	C2	111.27(19)	C11	C10	C9	119.2(3)
N1	C1	C5	110.5(2)	C10	C11	C12	120.3(3)
C5	C1	C2	114.2(2)	C11	C12	C7	121.2(3)
C1	C2	Cl1	110.95(17)	C14	C13	C3	118.8(2)
C1	C2	C3	108.34(19)	C14	C13	C18	118.8(3)
C3	C2	Cl1	109.73(17)	C18	C13	C3	122.4(3)
O1	C3	C2	109.9(2)	C13	C14	C15	120.7(3)
O1	C3	C13	112.1(2)	C16	C15	C14	119.8(4)
C13	C3	C2	110.3(2)	C17	C16	C15	120.0(3)
O1	C4	C19	111.5(2)	C16	C17	C18	120.7(3)
N1	C4	O1	127.1(2)	C17	C18	C13	120.0(3)
N1	C4	C19	121.3(2)	C20	C19	C4	121.9(2)
C6	C5	C1	122.0(2)	C20	C19	C24	119.2(2)
C5	C6	C7	128.2(2)	C24	C19	C4	118.8(2)
C8	C7	C6	123.9(2)	C19	C20	C21	119.8(3)
C12	C7	C6	118.1(2)	C22	C21	C20	120.5(3)
C12	C7	C8	117.9(2)	C21	C22	C23	119.7(3)
C22	C23	C24	120.4(3)	C23	C24	C19	120.3(3)

Table 5: Hydrogen Fractional Atomic Coordinates (×10^4) and Equivalent Isotropic Displacement Parameters (Å^2×10^3) for **2a**. *Ueq* is defined as 1/3 of the trace of the orthogonalised *Uij*.

Atom	x	y	z	*Ueq*
H1	5629	7183	5012	35
H2	8065	7333	4827	35
H3	7984	7536	3734	39
H5	6417	9636	5673	36
H6	5796	6943	6020	35
H8	6197	10338	6695	48
H9	6716	10763	7712	55
H10	7104	8950	8388	50
H11	6921	6684	8042	51
H12	6384	6235	7027	44
H14	8374	5260	4368	49
H15	7574	2980	4461	65
H16	5281	2386	4128	81
H17	3806	4050	3696	85
H18	4566	6342	3622	62
H20	5086	9858	2891	50
H21	3463	11274	2367	55
H22	1431	12126	2858	51
H23	1051	11630	3886	49
H24	2678	10263	4421	42

9.2 X-ray crystal structure for 4

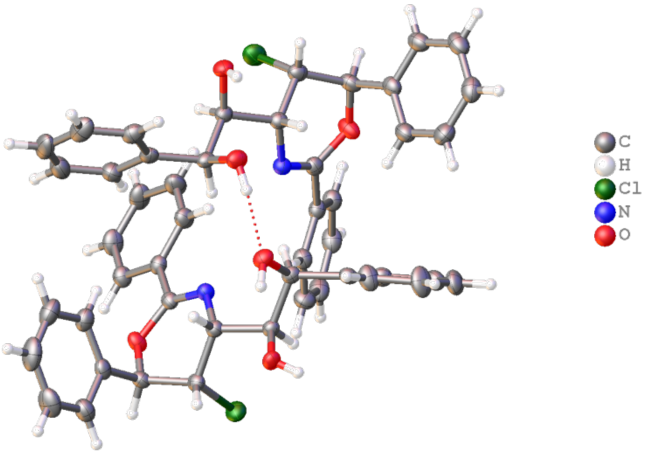

Experimental. Single colourless needle-shaped crystals of **4** used as received. A suitable crystal with dimensions 0.34 × 0.08 × 0.06 mm^3 was selected and mounted on a nylon loop with paratone oil on a Bruker APEX-II CCD diffractometer. The crystal was kept at a steady T = 173 K during data collection. The structure was solved with the ShelXT solution program using dual methods and by using Olex2 1.5 as the graphical interface. The model was refined with XL using full matrix least squares minimisation on $\boldsymbol{F}^2$.[9,10]

Crystal Data. $C_{24}H_{22}ClNO_3$, M_r = 407.87, monoclinic, $P2_1$ (No. 4), a = 10.39310(10) Å, b = 9.62820(10) Å, c = 20.5187(2) Å, β = 98.2450(10)°, $\alpha = \gamma$ = 90°, V = 2032.02(4) Å^3, T = 173(2) K, Z = 4, Z' = 2, μ(CuK_α) = 1.869, 27382 reflections measured, 7691 unique (R_{int} = 0.0470) which were used in all calculations. The final wR_2 was 0.0732 (all data) and R_1 was 0.0306 (I≥2 σ(I)).

Crystal data and structure refinement

**Reflections:**	d min (CuKα) 2Θ=144.3° 0.81	I/σ(I) 21.6	Rint m=3.56 4.70%	Full 135.4° 99.3	
**Refinement:**	Shift 0.001	Max Peak 0.2	Min Peak -0.2	GooF 1.027	Hooft .018(6)

Compound	**4**	Parameters	539
Formula	$C_{24}H_{22}ClNO_3$	Restraints	1
**CCDC**	**2337932**		
$D_{calc.}$/ g cm^{-3}	1.333	Largest Peak	0.159
μ/mm^{-1}	1.869	Deepest Hole	-0.186
Formula Weight	407.87	GooF	1.027
Colour	colourless	wR_2 (all data)	0.0732
Shape	needle	wR_2	0.0710
Size/mm^3	0.34×0.08×0.06	R_1 (all data)	0.0346
T/K	173(2)	R_1	0.0306
Crystal System	monoclinic		
Flack Parameter	0.018(6)		
Hooft Parameter	0.010(7)		
Space Group	$P2_1$		
a/Å	10.39310(10)		
b/Å	9.62820(10)		
c/Å	20.5187(2)		
α/°	90		
β/°	98.2450(10)		
γ/°	90		
V/Å^3	2032.02(4)		
Z	4		
Z'	2		
Wavelength/Å	1.541838		
Radiation type	CuK$_\alpha$		
Θ_{min}/°	4.298		
Θ_{max}/°	72.150		

Measured Refl.	27382
Independent Refl.	7691
Reflections Used	7125
R_{int}	0.0470

The following are 50% thermal ellipsoidal drawings of the molecule in the asymmetric cell with various amount of labeling:

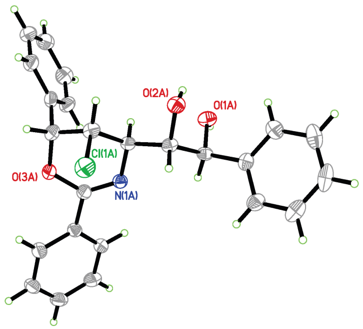

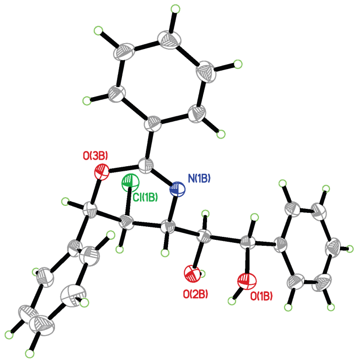

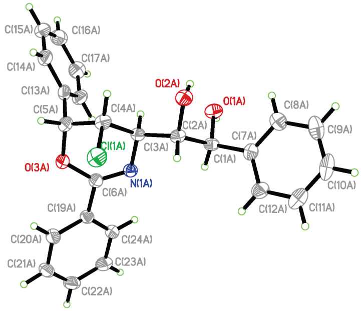

The Model has Chirality at C1A (Chiral SPGR) S Verify; The Model has Chirality at C2A (Chiral SPGR) S Verify; The Model has Chirality at C3A (Chiral SPGR) R Verify; The Model has Chirality at C4A (Chiral SPGR) S Verify; The Model has Chirality at C5A (Chiral SPGR) R Verify.

The Model has Chirality at C1B (Chiral SPGR) S Verify; The Model has Chirality at C2B (Chiral SPGR) S Verify; The Model has Chirality at C3B (Chiral SPGR) R Verify; The Model has Chirality at C4B (Chiral SPGR) S Verify; The Model has Chirality at C5B (Chiral SPGR) R Verify:

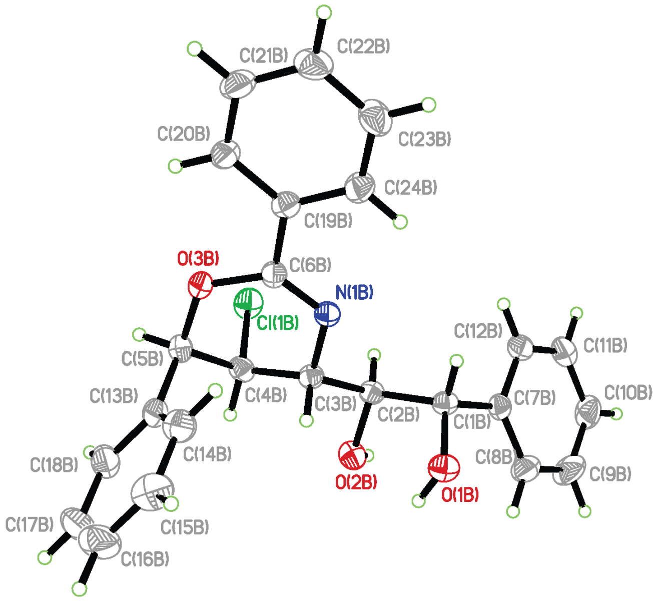

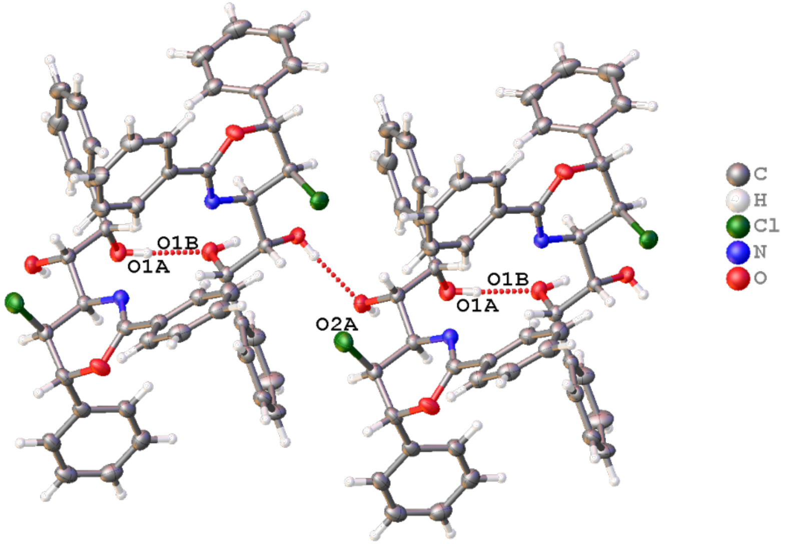

The following hydrogen bonding interactions with a maximum D-D distance of 3.1 Å and a minimum angle of 110° are present in **4**: O1A–O1B: 2.668 Å, O2A–O1A: 2.673 Å, O1B–O2B: 2.685 Å, O2B–O2A_*1: 2.718 Å*:

Packing diagram of **4**:

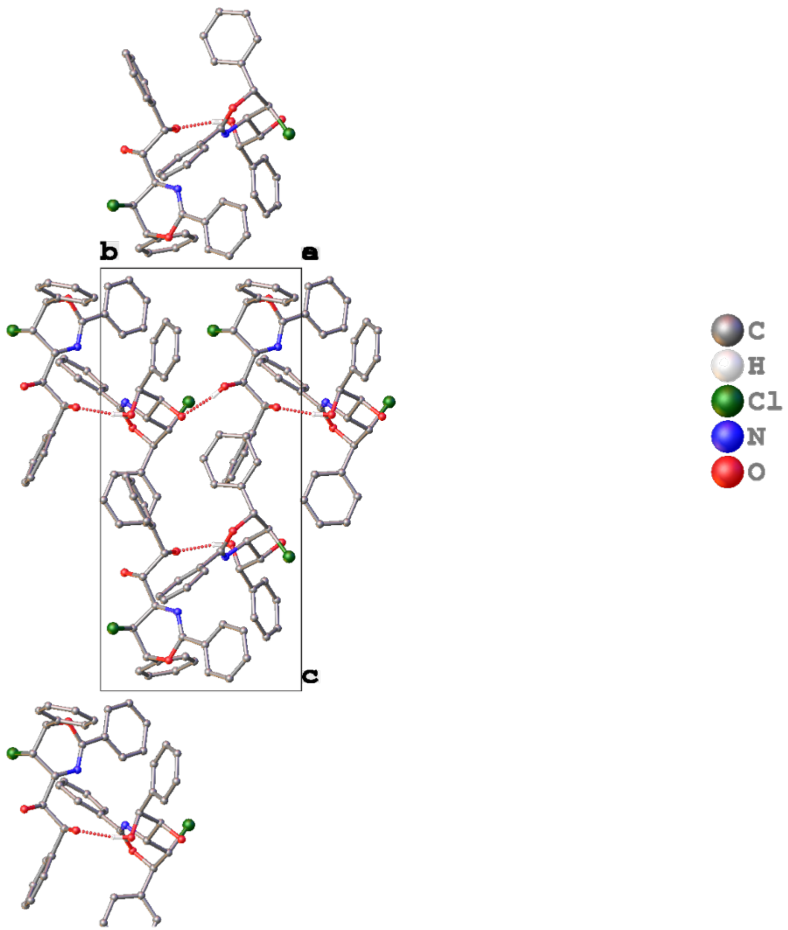

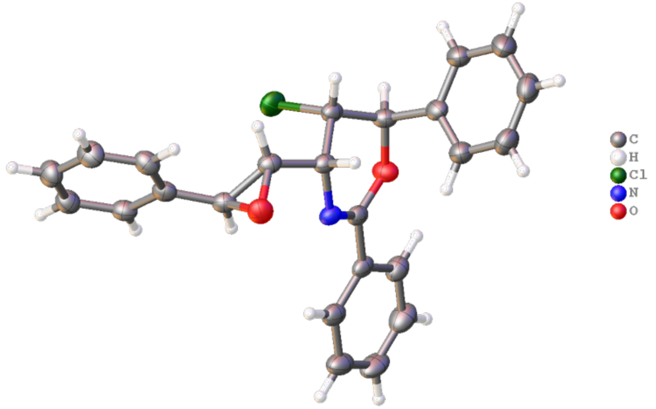

9.3 X-ray crystal structure for 5

Experimental. Single colourless needle-shaped crystals of **5** used as received. A suitable crystal with dimensions 0.22 × 0.10 × 0.08 mm^3 was selected and mounted on a nylon loop with paratone oil on a Bruker APEX-II CCD diffractometer. The crystal was kept at a steady T = 173 K during data collection. The structure was solved with the XT (Sheldrick, 2015) solution program using dual methods and by using Olex2 1.5 as the graphical interface. The model was refined with XL using full matrix least squares minimisation on F^2. [9, 10]

Crystal Data. $C_{24}H_{20}ClNO_2$, M_r = 389.86, orthorhombic, $P2_12_12_1$ (No. 19), a = 10.0607(3) Å, b = 10.4054(3) Å, c = 19.2999(5) Å, $\alpha = \beta = \gamma = 90^\circ$, V = 2020.42(10) Å^3, T = 173(2) K, Z = 4, Z' = 1, μ(CuK$_\alpha$) = 1.820, 12613 reflections measured, 3804 unique (R_{int} = 0.0423) which were used in all calculations. The final wR_2 was 0.0793 (all data) and R_1 was 0.0323 (I≥2 σ(I)).

Crystal data and structure refinement:

Compound	**5**		
Formula	$C_{24}H_{20}ClNO_2$	R_{int}	0.0423
CCDC	2337937		
$D_{calc.}$/ g cm^{-3}	1.282	Parameters	253
μ/mm^{-1}	1.820	Restraints	0
Formula Weight	389.86	Largest Peak	0.154
Colour	colourless	Deepest Hole	-0.193
Shape	needle	GooF	1.029
Size/mm^3	0.22×0.10×0.08	wR_2 (all data)	0.0793
T/K	173(2)	wR_2	0.0761
Crystal System	orthorhombic	R_1 (all data)	0.0385
Flack Parameter	0.041(10)	R_1	0.0323
Hooft Parameter	0.031(10)		
Space Group	$P2_12_12_1$		
a/Å	10.0607(3)		
b/Å	10.4054(3)		
c/Å	19.2999(5)		
α/°	90		
β/°	90		
γ/°	90		
V/Å^3	2020.42(10)		
Z	4		
Z'	1		
Wavelength/Å	1.541838		
Radiation type	CuK$_\alpha$		
Θ_{min}/°	4.582		
Θ_{max}/°	69.898		

Measured Refl.	12613
Independent Refl.	3804
Reflections Used	3403

The following are 50% thermal ellipsoidal drawings of the molecule in the asymmetric cell with various amount of labeling:

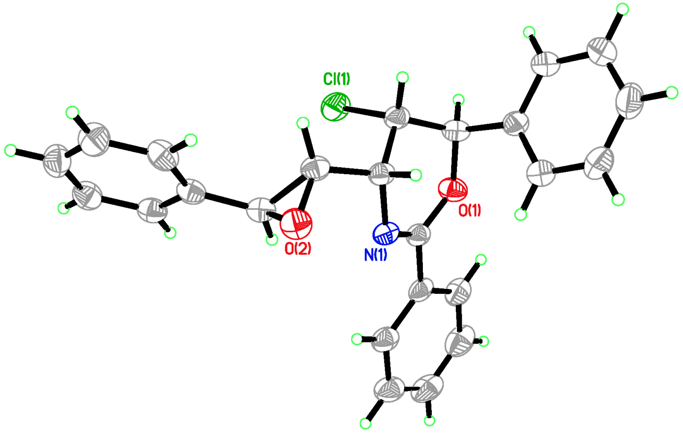

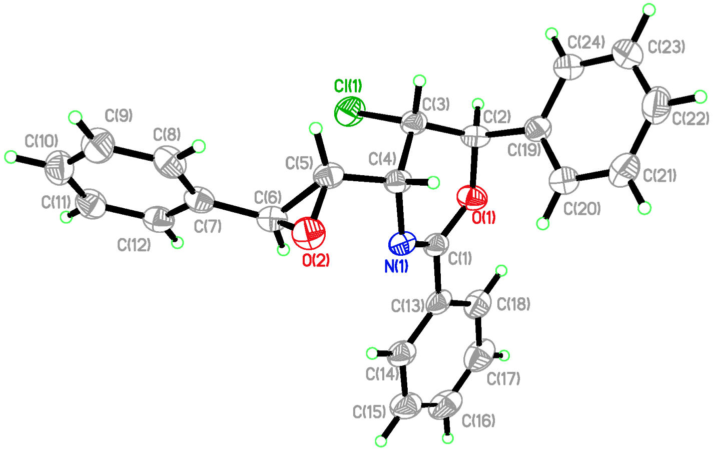

The Model has Chirality at C2 (Chiral SPGR) R Verify; The Model has Chirality at C3 (Chiral SPGR) S Verify; The Model has Chirality at C4 (Chiral SPGR) R Verify; The Model has Chirality at C5 (Chiral SPGR) S Verify; The Model has Chirality at C6 (Chiral SPGR) S Verify:

Packing diagram of **5**:

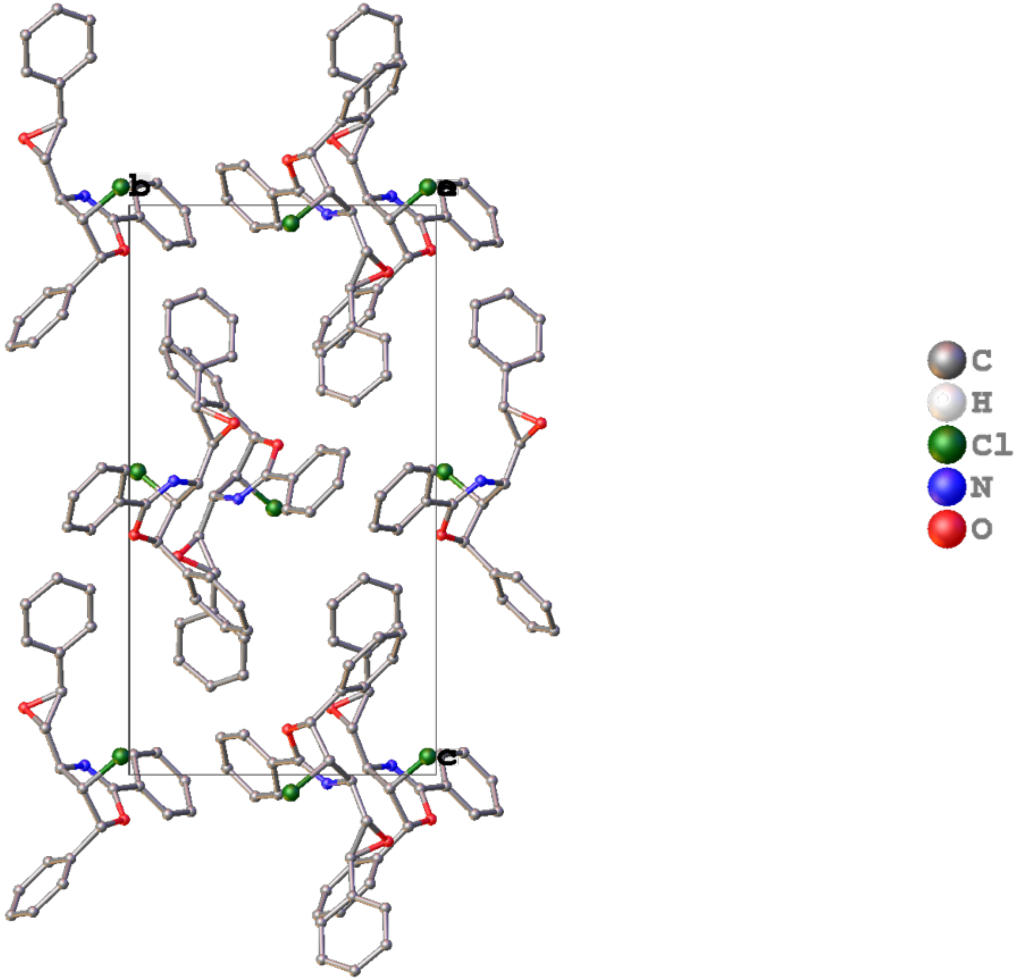

10. Reference

(1) Still, W. C.; Kahn, M.; Mitra, A. Rapid chromatographic technique for preparative separations with moderate resolution. *The Journal of Organic Chemistry* **1978**, *43* (14), 2923-2925. DOI: 10.1021/jo00408a041.

(2) Bhaskar R, S. R., Subba R. Tetrahydrothiapyran-4,ones - Source for annelated 1,2,3- selena/thiadiazoles and their reactivity. *Indian Journal of Chemistry - Section B Organic and Medicinal Chemistry* **1999**, *38* (12), 1342 - 1348.

(3) Polaquini, C. R.; Marques, B. C.; Ayusso, G. M.; Morão, L. G.; Sardi, J. C. O.; Campos, D. L.; Silva, I. C.; Cavalca, L. B.; Scheffers, D.-J.; Rosalen, P. L.; et al. Antibacterial activity of a new monocarbonyl analog of curcumin MAC 4 is associated with divisome disruption. *Bioorganic Chemistry* **2021**, *109*, 104668. DOI: https://doi.org/10.1016/j.bioorg.2021.104668.

(4) Nagao, Y.; Hisanaga, T.; Egami, H.; Kawato, Y.; Hamashima, Y. Desymmetrization of Bisallylic Amides through Catalytic Enantioselective Bromocyclization with BINAP Monoxide. *Chemistry – A European Journal* **2017**, *23* (66), 16758-16762. DOI: https://doi.org/10.1002/chem.201704847.

(5) Farran, D.; Slawin, A. M. Z.; Kirsch, P.; O'Hagan, D. Diastereoselective Synthesis of 2,3,4,5,6-Pentafluoroheptanes. *The Journal of Organic Chemistry* **2009**, *74* (18), 7168-7171. DOI: 10.1021/jo901360e.

(6) Ashtekar, K. D.; Ding, X.; Toma, E.; Sheng, W.; Gholami, H.; Rahn, C.; Reed, P.; Borhan, B. Mechanistically Inspired Route toward Hexahydro-2H-chromenes via Consecutive [4 + 2] Cycloadditions. *Organic Letters* **2016**, *18* (16), 3976-3979. DOI: 10.1021/acs.orglett.6b01742.

(7) Jaganathan, A.; Garzan, A.; Whitehead, D. C.; Staples, R. J.; Borhan, B. A Catalytic Asymmetric Chlorocyclization of Unsaturated Amides. *Angewandte Chemie International Edition* **2011**, *50* (11), 2593-2596. DOI: https://doi.org/10.1002/anie.201006910.

(8) Jaganathan, A.; Staples, R. J.; Borhan, B. Kinetic Resolution of Unsaturated Amides in a Chlorocyclization Reaction: Concomitant Enantiomer Differentiation and Face Selective Alkene Chlorination by a Single Catalyst. *Journal of the American Chemical Society* **2013**, *135* (39), 14806-14813. DOI: 10.1021/ja407241d.

(9) Oleg V. Dolomanov, L. J. B., Richard J. Gildea, Judith A. K. Howarda, Horst Puschmanna. OLEX2: a complete structure solution, refinement and analysis program. *J. Appl. Cryst.* **2009**, *42*, 339 - 341. DOI: https://doi.org/10.1107/S0021889808042726.

(10) Sheldrick, G. M. A short history of SHELX. *Acta Crystallogr A* **2008**, *64* (Pt 1), 112-122. DOI: 10.1107/s0108767307043930 From NLM.

(11) Software for the CCD Detector Systems for Determining Data Collection Parameters. *COSMO-V1.61* **2000**.

(12) Software for the Integration of CCD Detector System Bruker Analytical X-ray Systems, Bruker axs, Madison, WI **2013**.

11. HPLC SPECTRA:

Compound 2a: (4*R*,5*S*,6*R*)-5-chloro-2,6-diphenyl-4-((*E*)-styryl)-5,6-dihydro-4*H*-1,3-oxazine

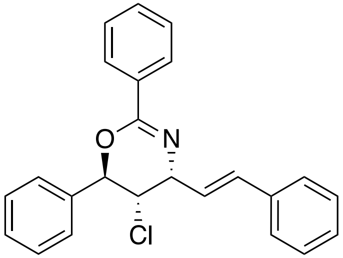

2a

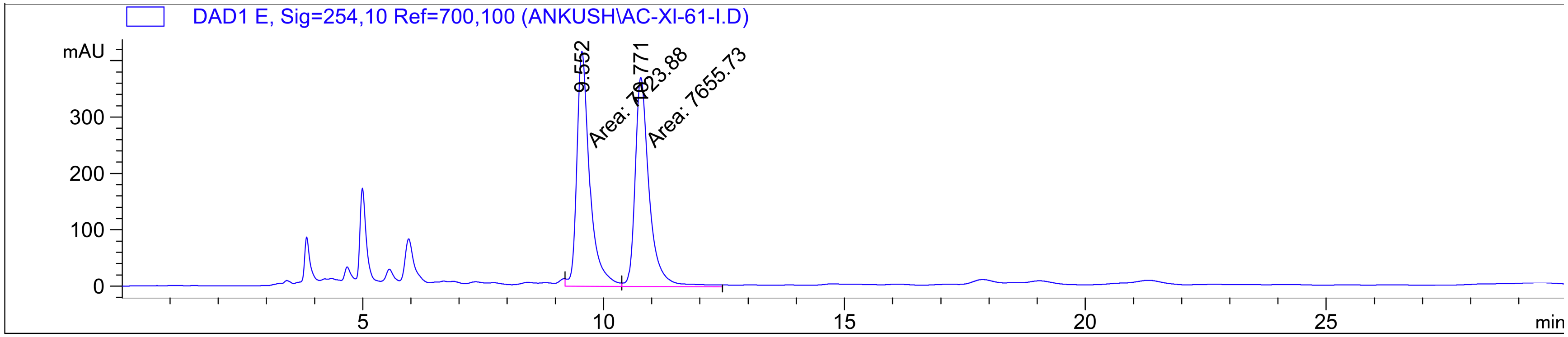

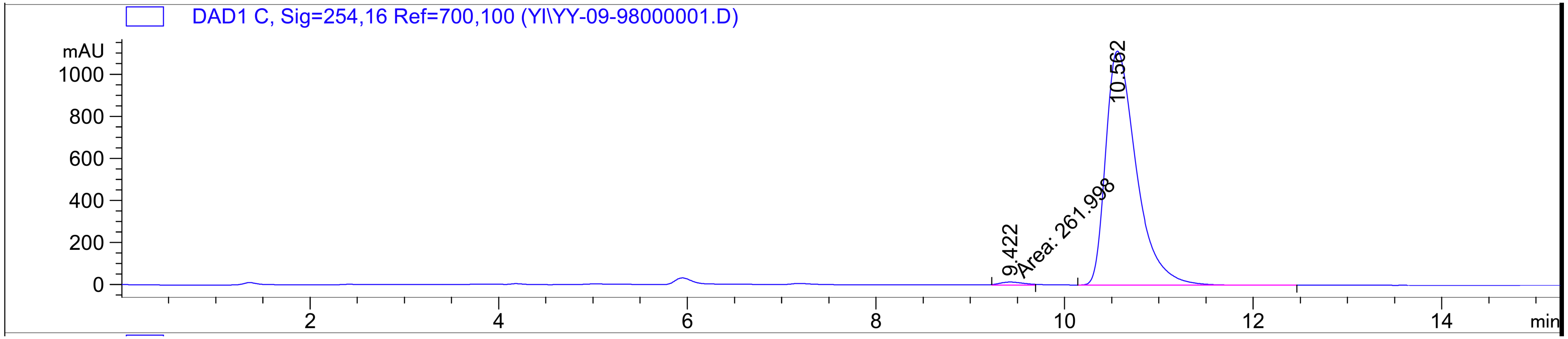


```
Signal 3: DAD1 C, Sig=254,16 Ref=700,100

Peak RetTime Type  Width      Area       Height      Area
  #    [min]        [min]    [mAU*s]      [mAU]        %
----|-------|----|-------|----------|----------|--------|
   1    9.422 MM    0.2967  261.99762   14.71719   1.0319
   2   10.562 VB    0.3477 2.51285e4  1111.56909  98.9681

Totals :                   2.53905e4  1126.28628
```

Compound 2b: (4*R*,5*S*,6*R*)-5-chloro-6-(4-chlorophenyl)-4-((*E*)-4-chlorostyryl)-2-phenyl-5,6-dihydro-4*H*-1,3-oxazine

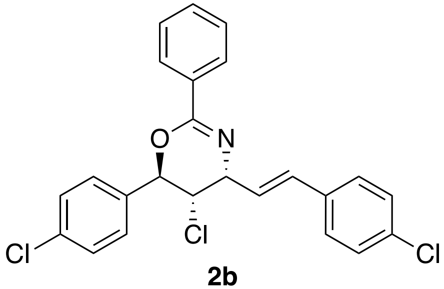

2b

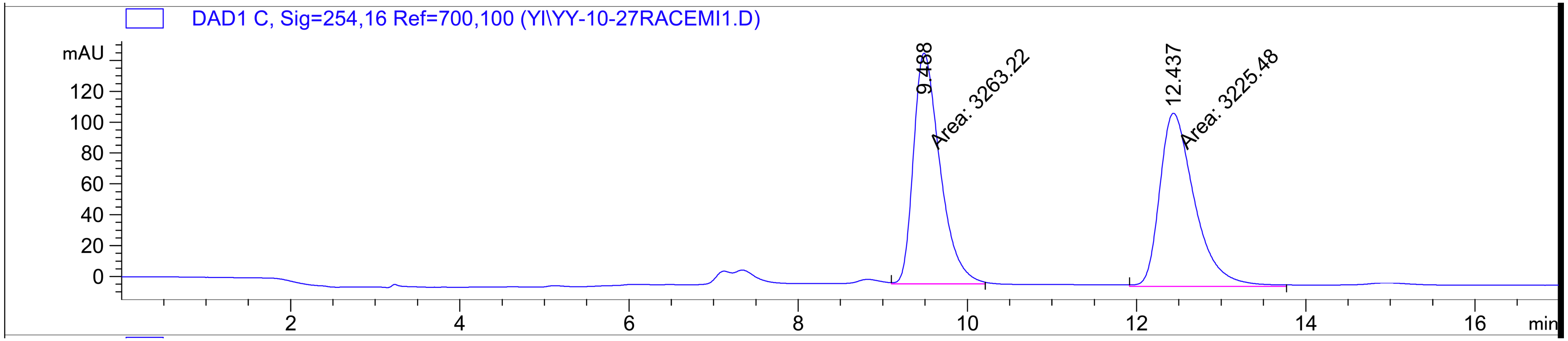

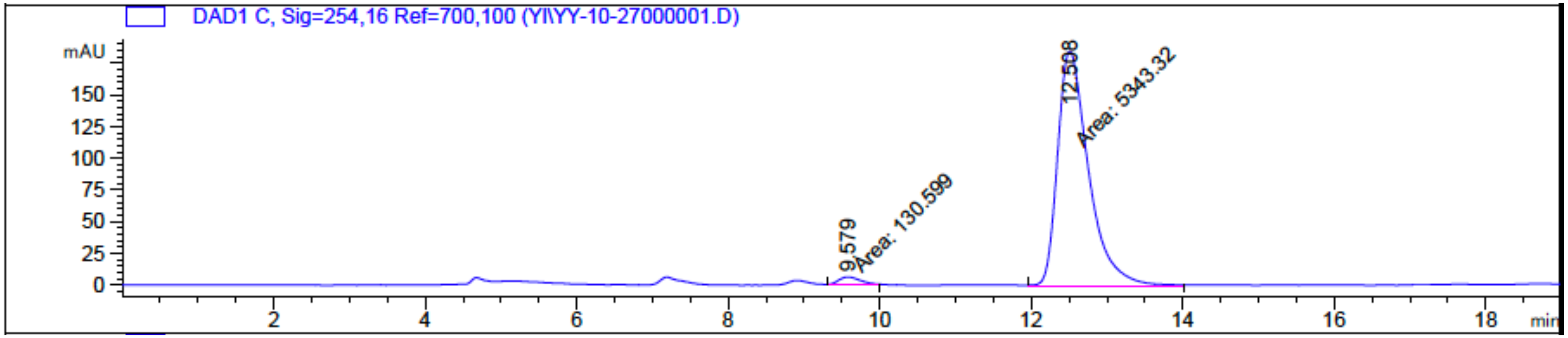

Signal 3: DAD1 C, Sig=254,16 Ref=700,100

Peak #	RetTime [min]	Type	Width [min]	Area [mAU*s]	Height [mAU]	Area %
1	9.579	MM	0.3439	130.59885	6.32883	2.3858
2	12.508	MM	0.4794	5343.32129	185.76498	97.6142

Totals : 5473.92014 192.09382

Compound 2c: (4*R*,5*S*,6*R*)-6-(4-bromophenyl)-4-((*E*)-4-bromostyryl)-5-chloro-2-phenyl-5,6-dihydro-4*H*-1,3-oxazine

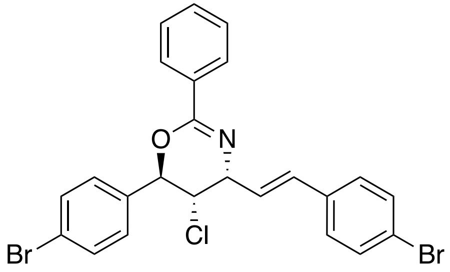
O
N
Br
Cl
Br

2c

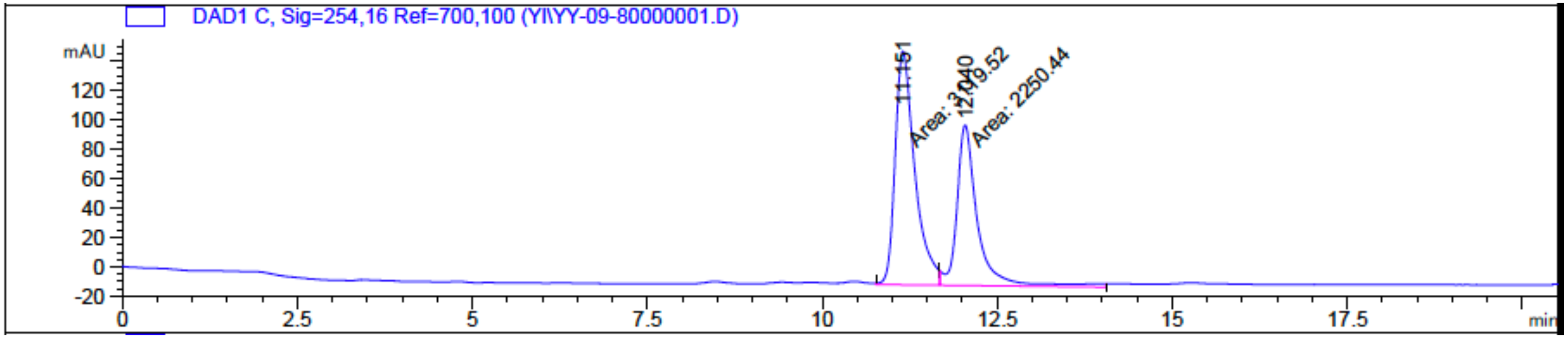
DAD1 C, Sig=254,16 Ref=700,100 (YI\YY-09-80000001.D)
mAU
Area: 2250.44
min

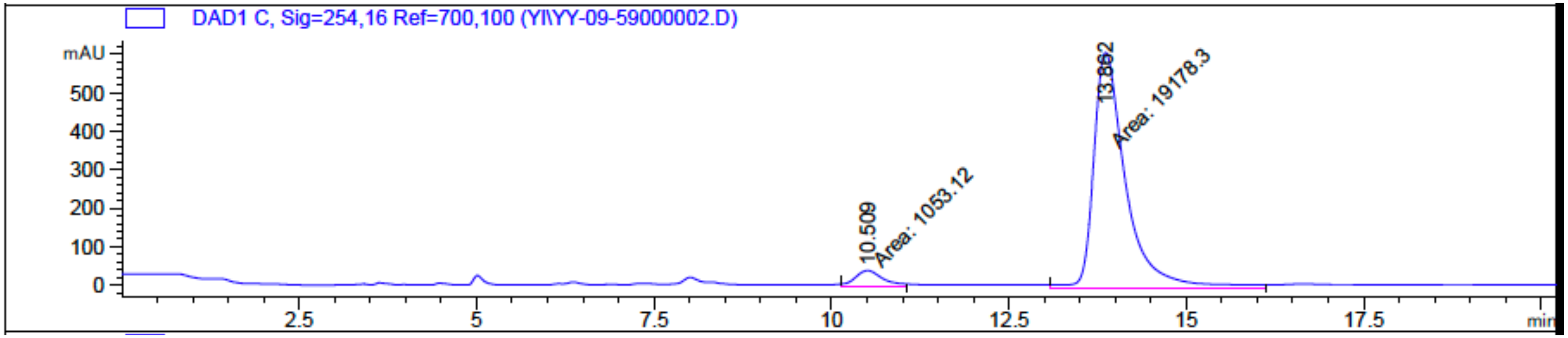
DAD1 C, Sig=254,16 Ref=700,100 (YI\YY-09-59000002.D)
mAU
10.509
Area: 1053.12
13.862
Area: 19178.3
min

Signal 3: DAD1 C, Sig=254,16 Ref=700,100

Peak #	RetTime [min]	Type	Width [min]	Area [mAU*s]	Height [mAU]	Area %
1	10.509	MM	0.4451	1053.12195	39.43406	5.2054
2	13.862	MM	0.5229	1.91783e4	611.31787	94.7946

Totals : 2.02315e4 650.75193

Compound 2d: (4*R*,5*S*,6*R*)-6-(3-bromophenyl)-4-((*E*)-3-bromostyryl)-5-chloro-2-phenyl-5,6-dihydro-4*H*-1,3-oxazine

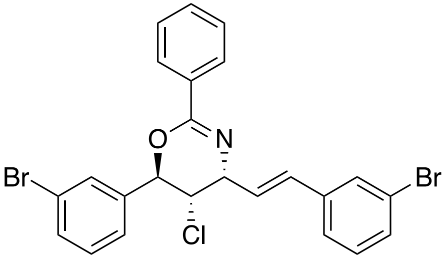

2d

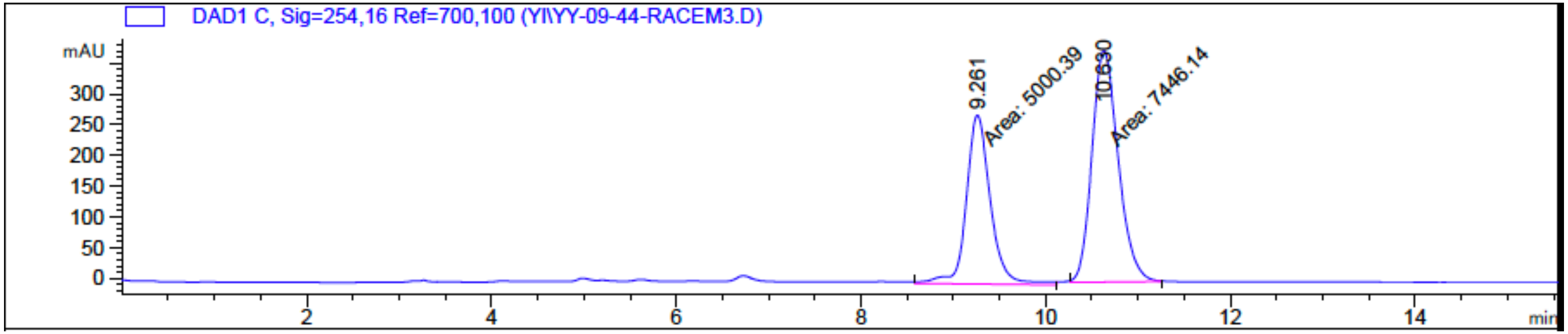

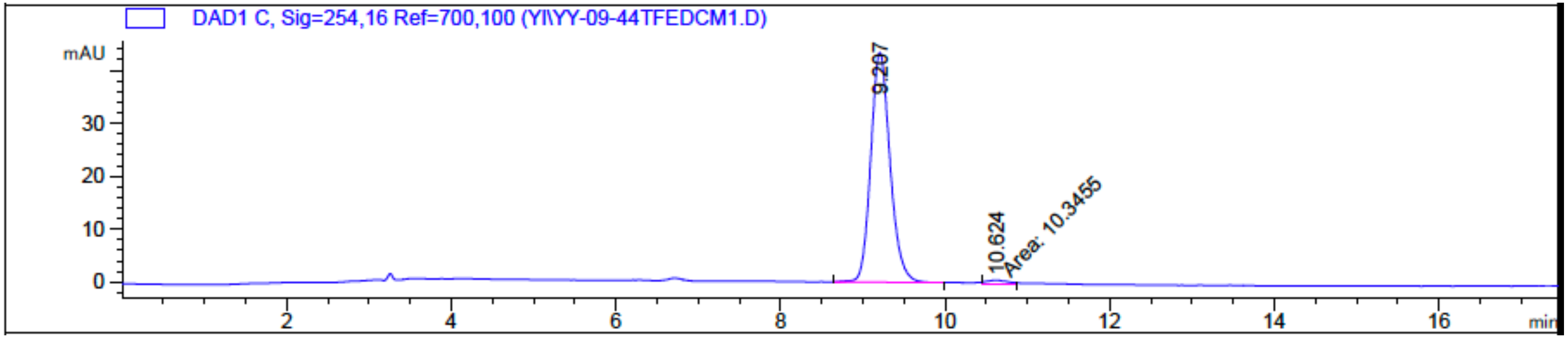

Signal 3: DAD1 C, Sig=254,16 Ref=700,100

Peak #	RetTime [min]	Type	Width [min]	Area [mAU*s]	Height [mAU]	Area %
1	9.207	BB	0.2500	706.37134	43.36895	98.5565
2	10.624	MM	0.2786	10.34545	6.18855e-1	1.4435

Totals : 716.71679 43.98780

Compound 2e: (4*R*,5*S*,6*R*)-5-chloro-6-(2-fluorophenyl)-4-((*E*)-2-fluorostyryl)-2-phenyl-5,6-dihydro-4*H*-1,3-oxazine

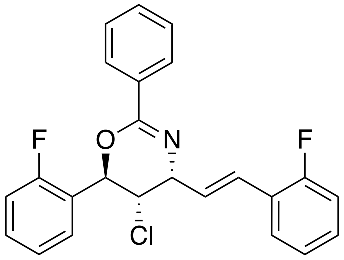

2e

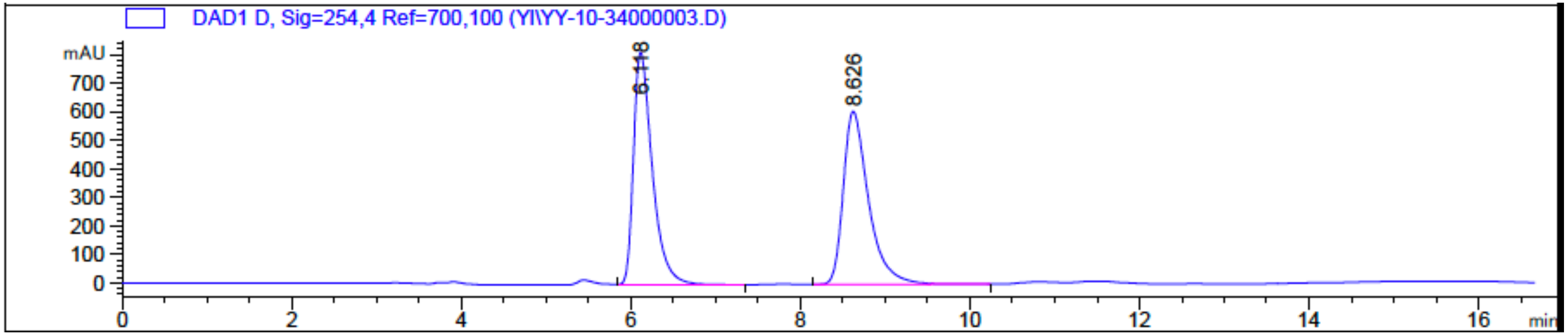

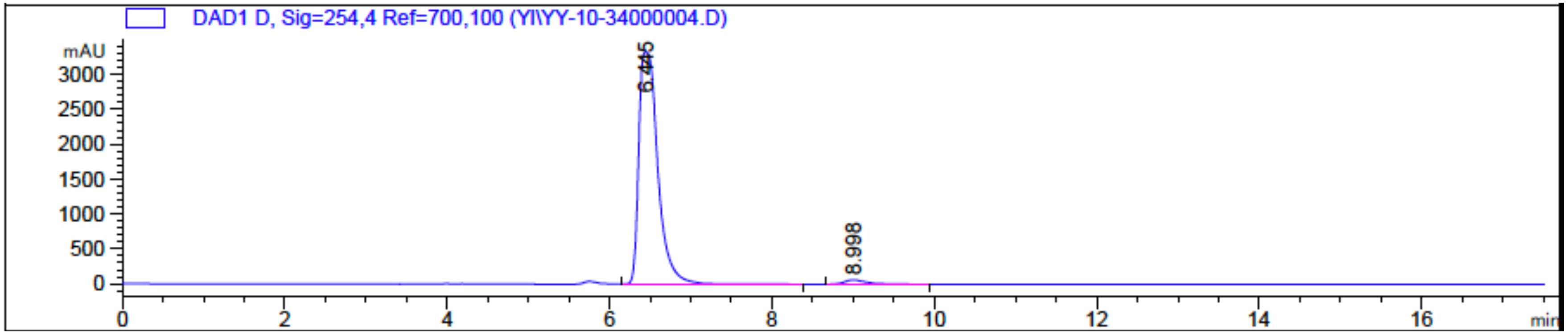

Signal 3: DAD1 D, Sig=254,4 Ref=700,100

Peak #	RetTime [min]	Type	Width [min]	Area [mAU*s]	Height [mAU]	Area %
1	6.445	VB	0.2514	5.41388e4	3334.32813	97.9946
2	8.998	BB	0.2763	1107.94592	60.32081	2.0054

Totals :				5.52468e4	3394.64894	

Compound 2f: (4*R*,5*S*,6*R*)-5-chloro-6-(4-fluorophenyl)-4-((*E*)-4-fluorostyryl)-2-phenyl-5,6-dihydro-4*H*-1,3-oxazine

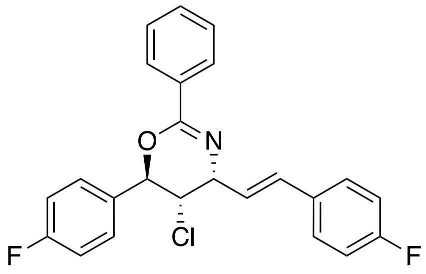

2f

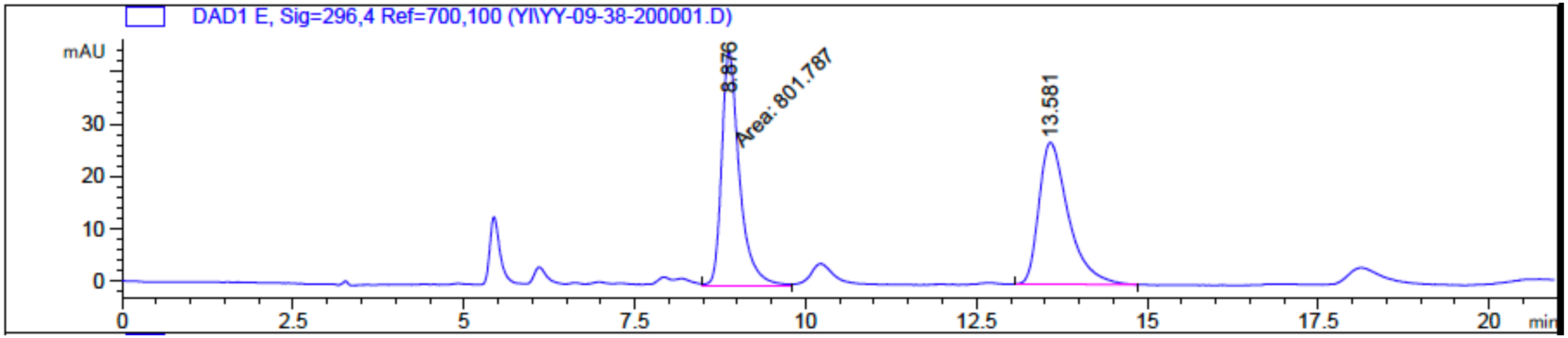

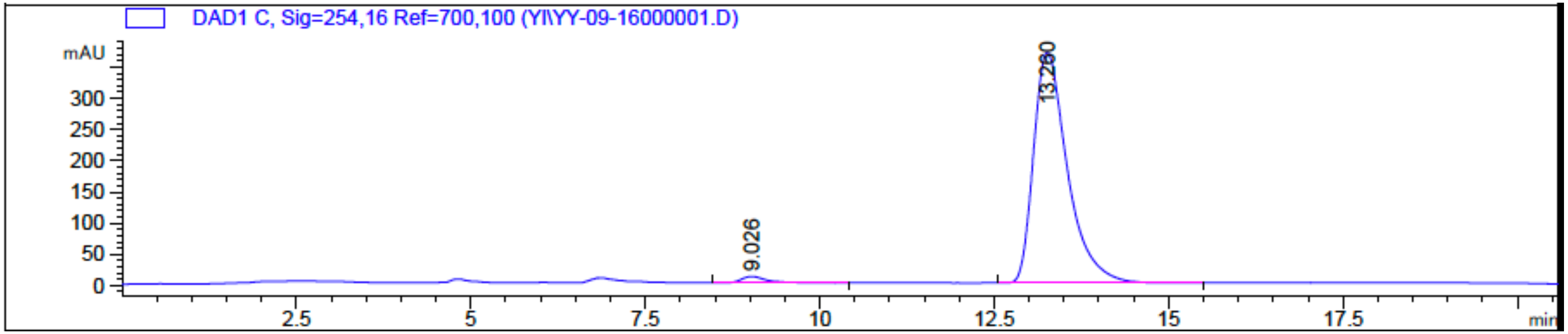

Signal 3: DAD1 C, Sig=254,16 Ref=700,100

Peak #	RetTime [min]	Type	Width [min]	Area [mAU*s]	Height [mAU]	Area %
1	9.026	BB	0.3890	261.64362	10.14223	2.0582
2	13.260	BB	0.5131	1.24507e4	369.54700	97.9418

Totals : 1.27123e4 379.68923

Compound 2g: (4*R*,5*S*,6*R*)-5-chloro-2-phenyl-6-(2,6-dichlorophenyl)-4-((*E*)-(2,6-dichloro)-styryl)-5,6-dihydro-4*H*-1,3-oxazine

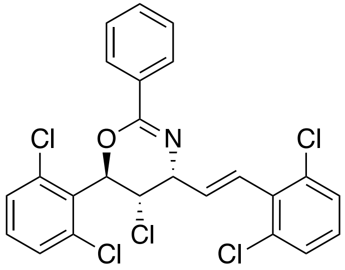

2g

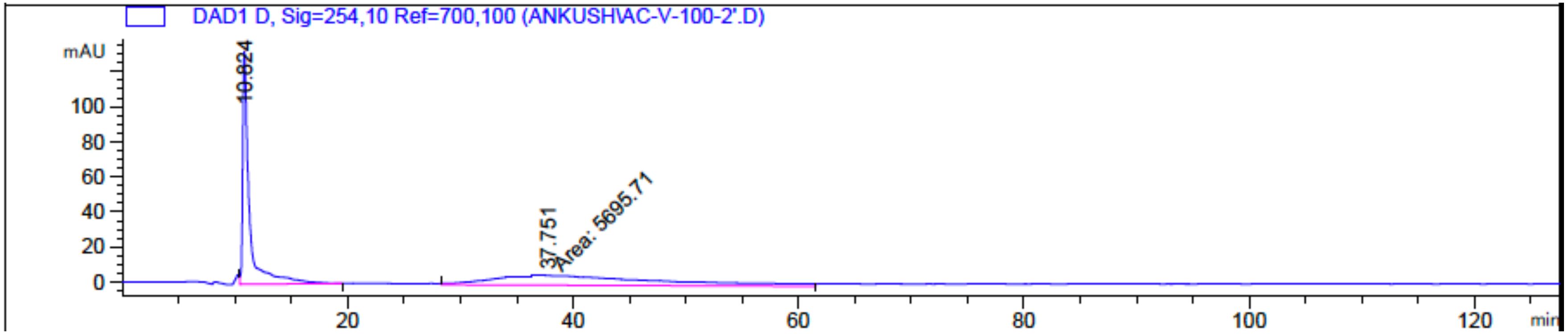

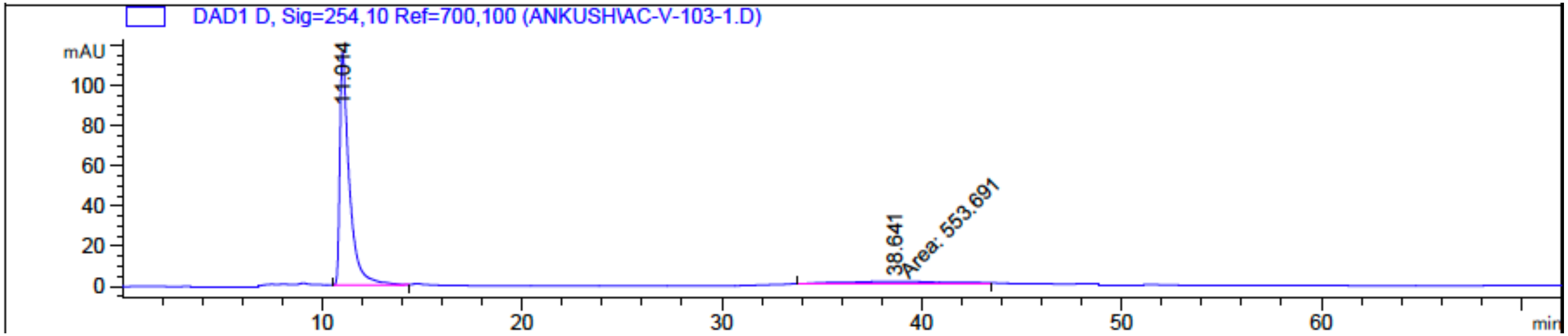

Signal 3: DAD1 D, Sig=254,10 Ref=700,100

Peak #	RetTime [min]	Type	Width [min]	Area [mAU*s]	Height [mAU]	Area %
1	11.014	BB	0.4662	3813.36255	115.79549	87.3212
2	38.641	MM	7.1937	553.69116	1.28282	12.6788

Totals : 4367.05371 117.07831

Compound 2h: (4*R*,5*S*,6*R*)-5-chloro-2-phenyl-6-(4-(trifluoromethyl)phenyl)-4-((*E*)-4-(trifluoromethyl)styryl)-5,6-dihydro-4*H*-1,3-oxazine

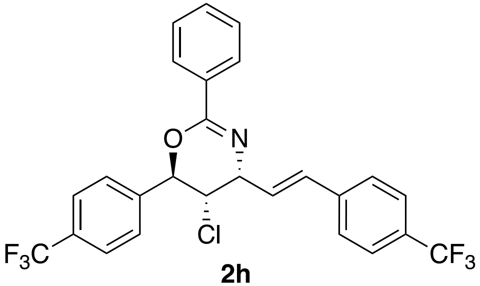

2h

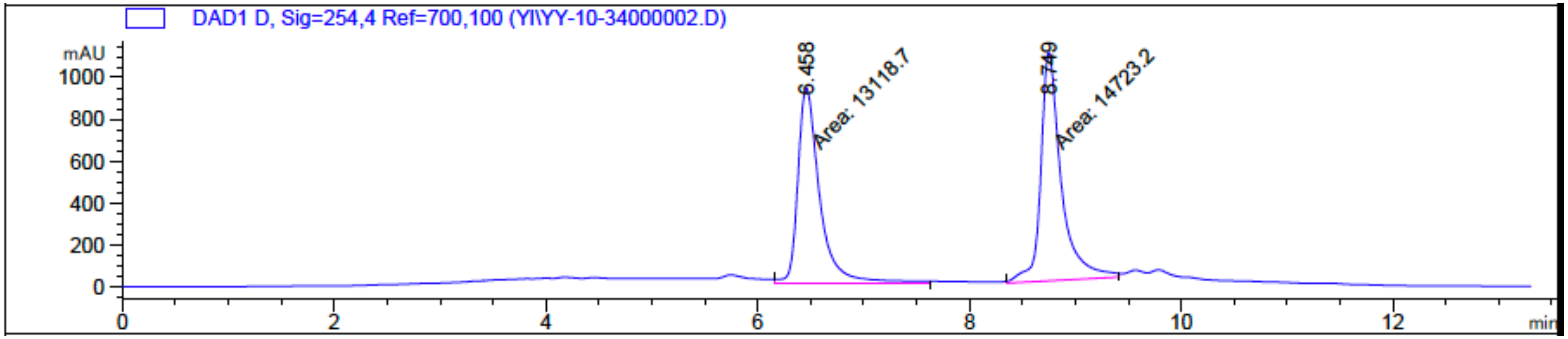

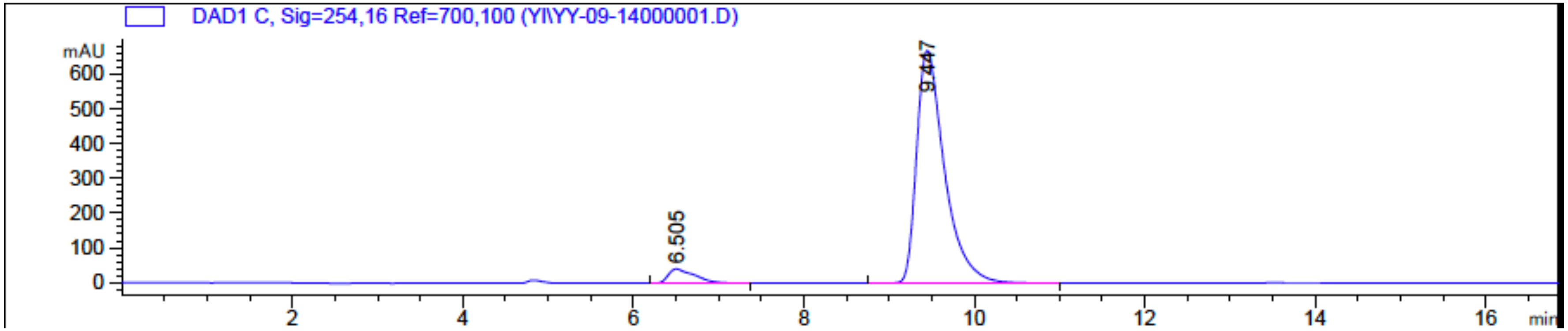

Signal 3: DAD1 C, Sig=254,16 Ref=700,100

Peak #	RetTime [min]	Type	Width [min]	Area [mAU*s]	Height [mAU]	Area %
1	6.505	BB	0.2941	856.10284	40.58813	5.3625
2	9.447	BB	0.3424	1.51084e4	666.61365	94.6375

Totals : 1.59645e4 707.20178

Compound 2i: (4*S*,5*R*,6*S*)-5-chloro-6-(3-methoxyphenyl)-4-((*E*)-3-methoxystyryl)-2-phenyl-5,6-dihydro-4*H*-1,3-oxazine

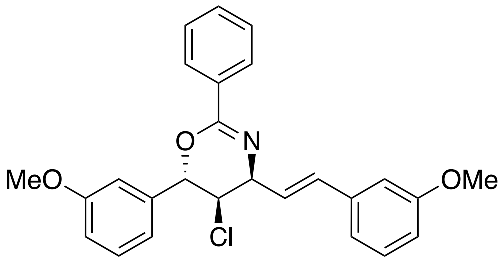
MeO
O
N
OMe
Cl

2i

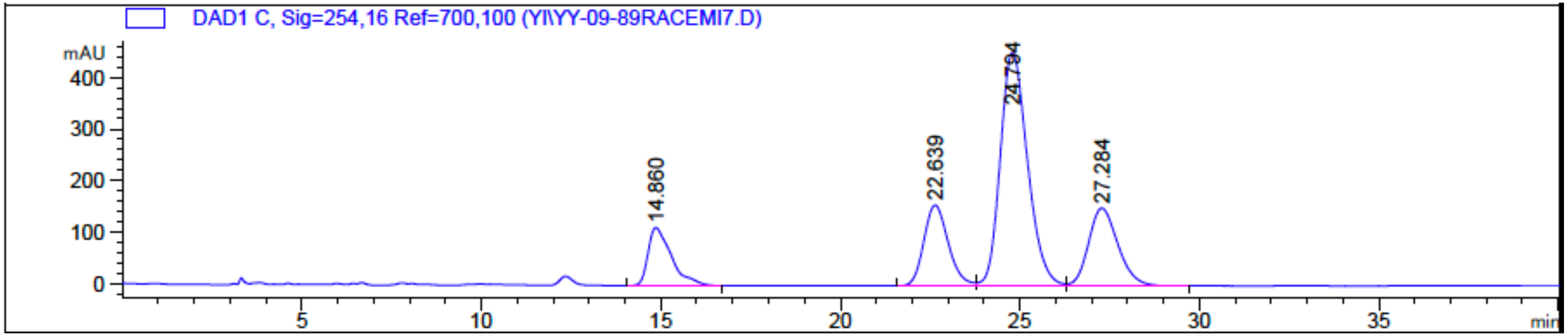
DAD1 C, Sig=254,16 Ref=700,100 (YI\YY-09-89RACEMI7.D)
mAU
400
300
200
100
0
14.860
22.639
24.794
27.284
5
10
15
20
25
30
35
min

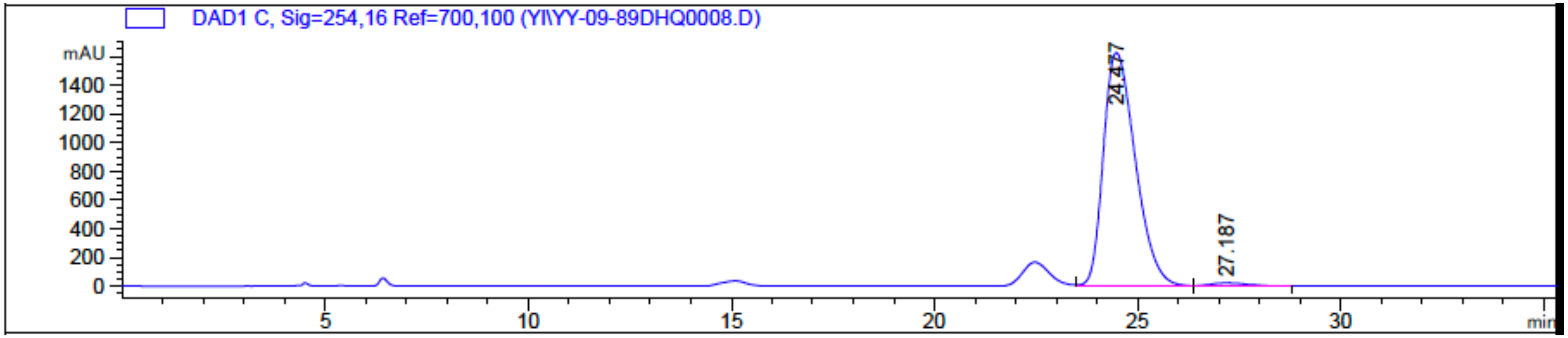
DAD1 C, Sig=254,16 Ref=700,100 (YI\YY-09-89DHQ0008.D)
mAU
1400
1200
1000
800
600
400
200
0
24.477
27.187
5
10
15
20
25
30
min

Signal 3: DAD1 C, Sig=254,16 Ref=700,100

Peak #	RetTime [min]	Type	Width [min]	Area [mAU*s]	Height [mAU]	Area %
1	24.477	VB	0.8560	8.87728e4	1623.35596	98.6793
2	27.187	BB	0.8728	1188.08826	20.00604	1.3207
Totals :				8.99609e4	1643.36200	

Compound 2j: (4*R*,5*S*,6*R*)-5-chloro-4-((*E*)-2-methylstyryl)-2-phenyl-6-(*o*-tolyl)-5,6-dihydro-4*H*-1,3-oxazine

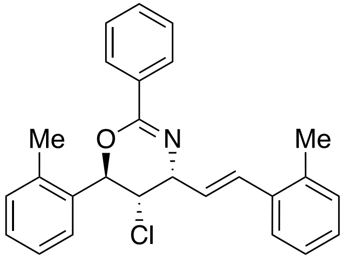

2j

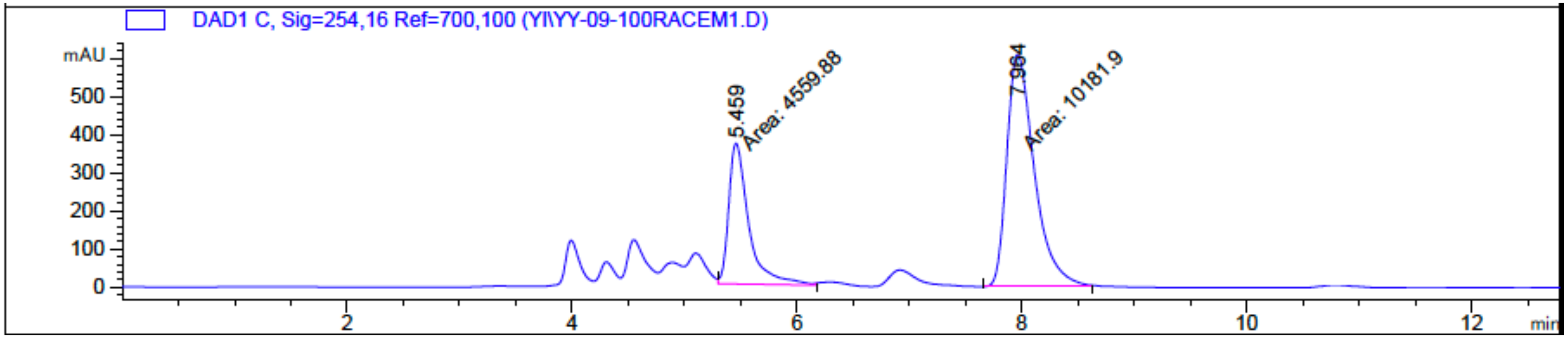

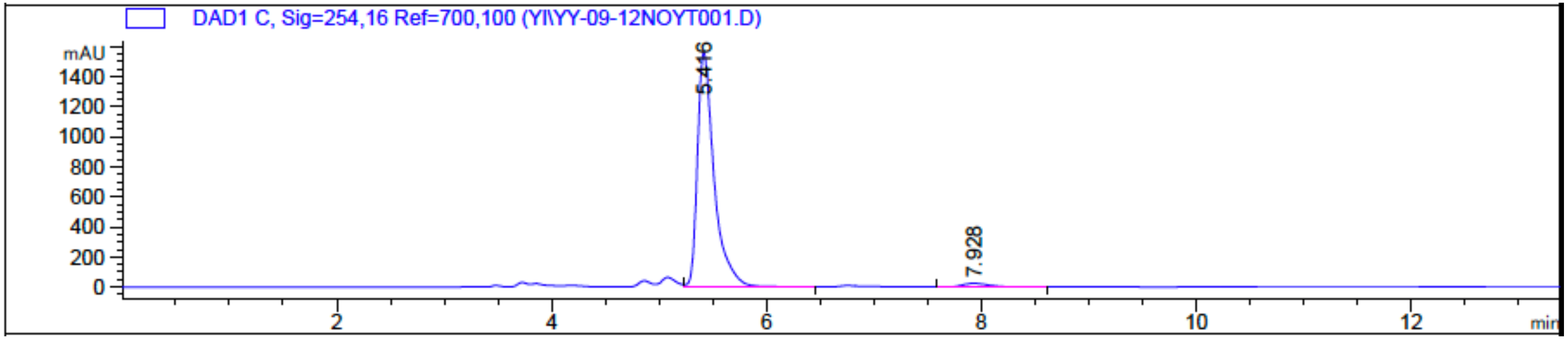

Signal 3: DAD1 C, Sig=254,16 Ref=700,100

Peak #	RetTime [min]	Type	Width [min]	Area [mAU*s]	Height [mAU]	Area %
1	5.416	VB	0.1621	1.68538e4	1558.91626	97.5963
2	7.928	BB	0.2568	415.09045	24.11980	2.4037

Totals : 1.72689e4 1583.03606

Compound 2k: (4*R*,5*S*,6*R*)-5-chloro-4-((*E*)-4-methylstyryl)-2-phenyl-6-(*p*-tolyl)-5,6-dihydro-4*H*-1,3-oxazine

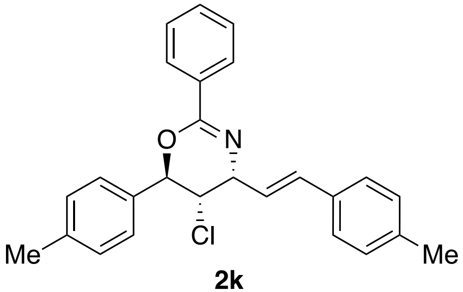

2k

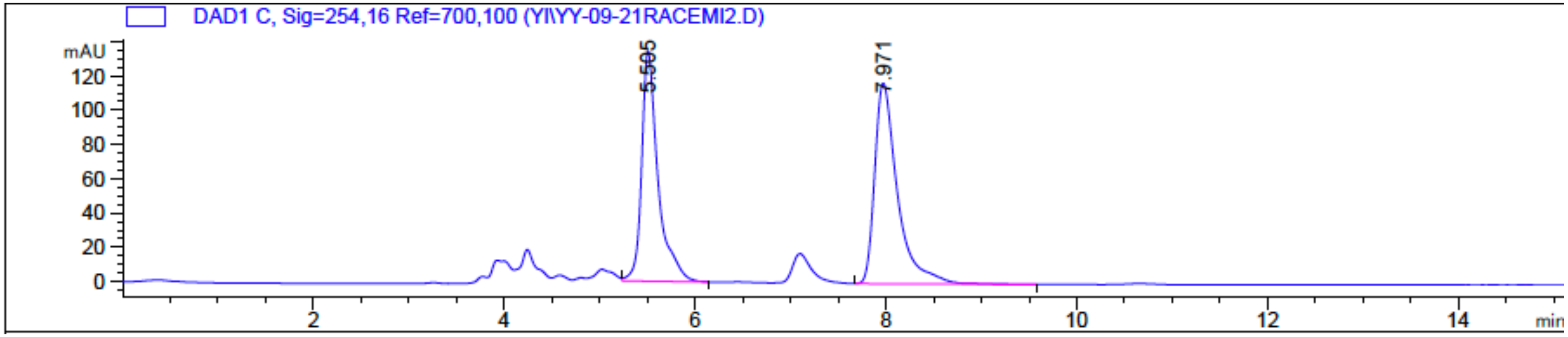

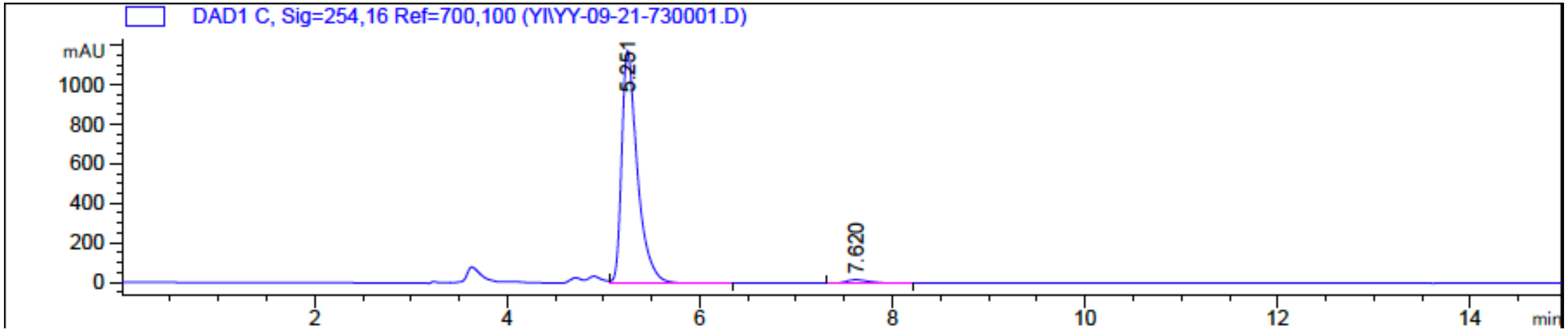

Signal 3: DAD1 C, Sig=254,16 Ref=700,100

Peak #	RetTime [min]	Type	Width [min]	Area [mAU*s]	Height [mAU]	Area %
1	5.251	VB	0.1674	1.32695e4	1178.09302	97.9867
2	7.620	BB	0.2373	272.64371	17.35245	2.0133

Totals : 1.35421e4 1195.44547

Compound 2l: (4*R*,5*S*,6*R*)-5-chloro-6-(4-methoxyphenyl)-4-((*E*)-4-methoxystyryl)-2-phenyl-5,6-dihydro-4*H*-1,3-oxazine

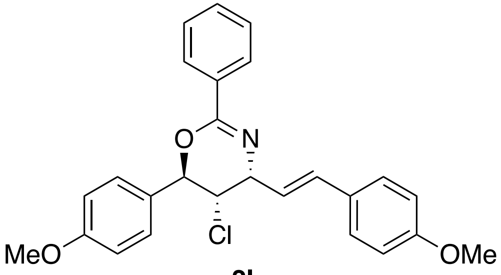

2l

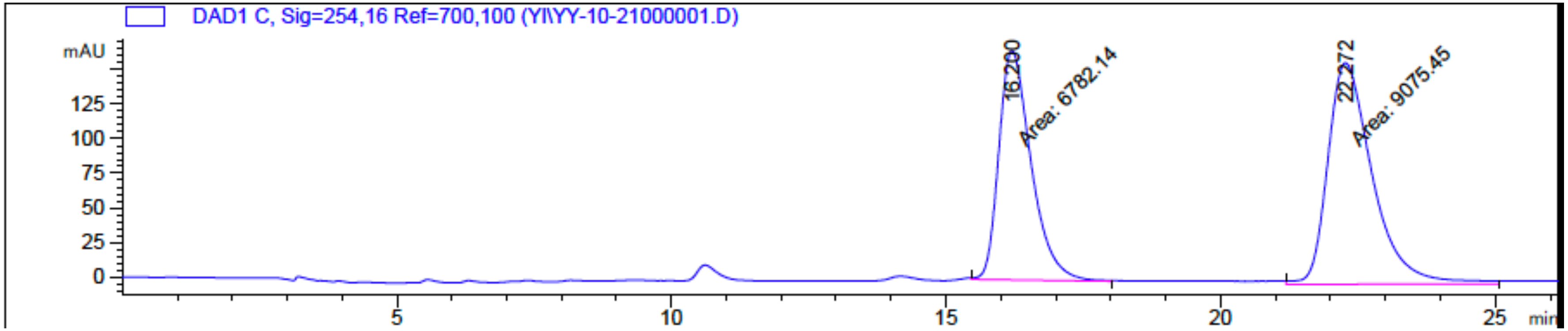

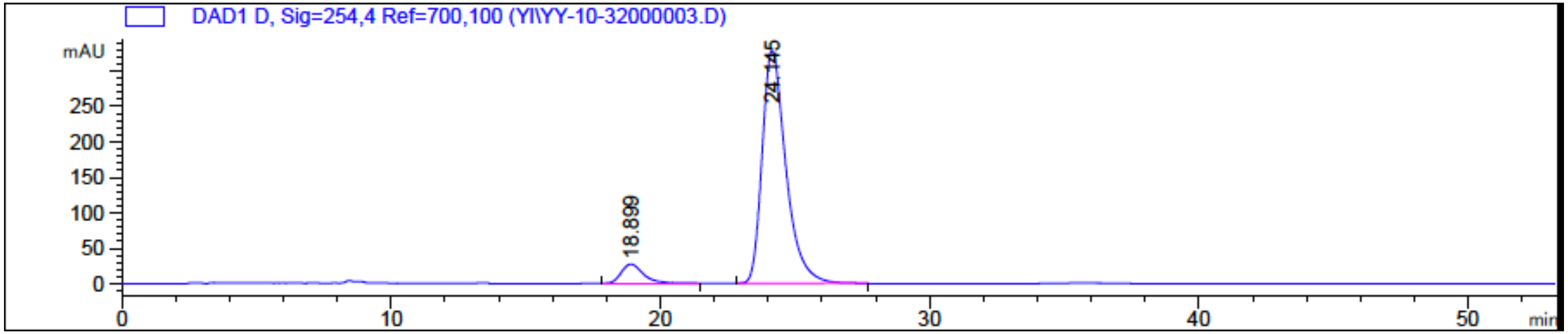

Signal 3: DAD1 D, Sig=254,4 Ref=700,100

Peak #	RetTime [min]	Type	Width [min]	Area [mAU*s]	Height [mAU]	Area %
1	18.899	BB	0.8439	1515.50952	26.79282	7.0249
2	24.145	BB	0.9340	2.00579e4	327.76486	92.9751
Totals :				2.15734e4	354.55769	

Compound 2m: (4*R*,5*S*,6*R*)-6-(4-(*tert*-butyl)phenyl)-4-((*E*)-4-(*tert*-butyl)styryl)-5-chloro-2-phenyl-5,6-dihydro-4*H*-1,3-oxazine

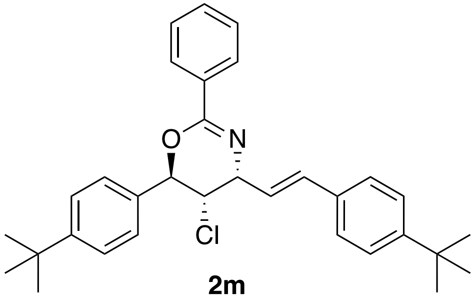

2m

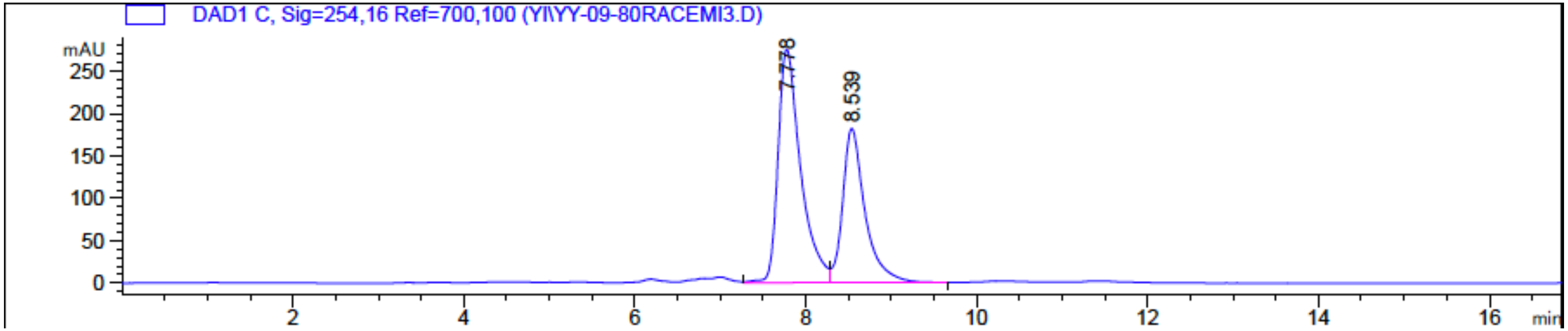

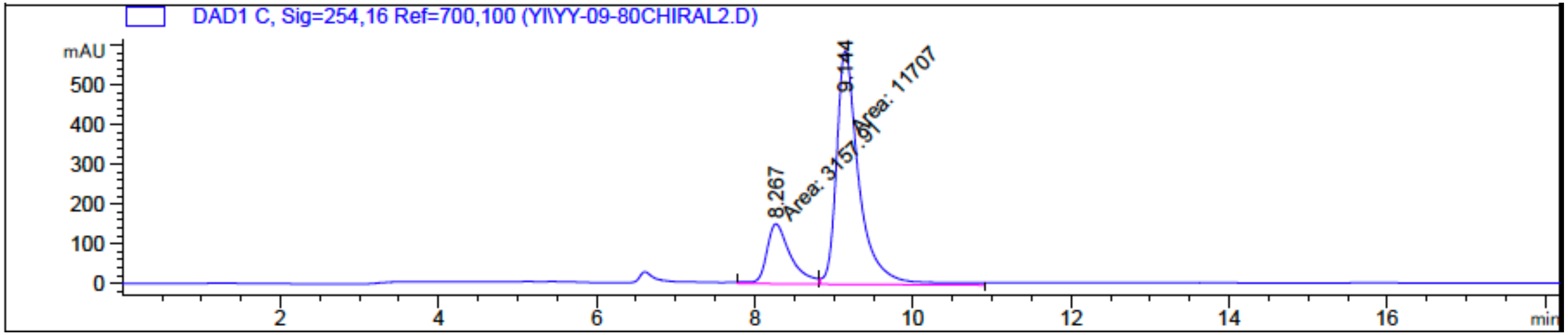

Signal 3: DAD1 C, Sig=254,16 Ref=700,100

Peak #	RetTime [min]	Type	Width [min]	Area [mAU*s]	Height [mAU]	Area %
1	8.267	MF	0.3502	3157.91406	150.29138	21.2441
2	9.144	FM	0.3329	1.17070e4	586.07489	78.7559

Totals : 1.48649e4 736.36627

Compound 2n: (4*R*,5*S*,6*R*)-5-chloro-2-phenyl-6-(thiophen-2-yl)-4-((*E*)-2-(thiophen-2-yl)vinyl)-5,6-dihydro-4*H*-1,3-oxazine

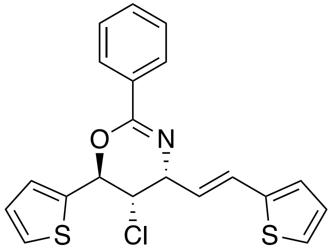

2n

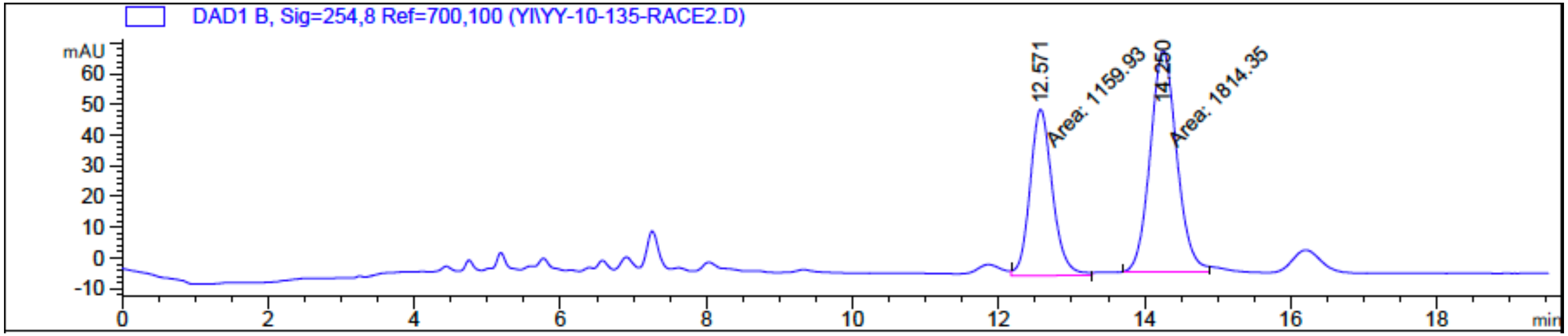

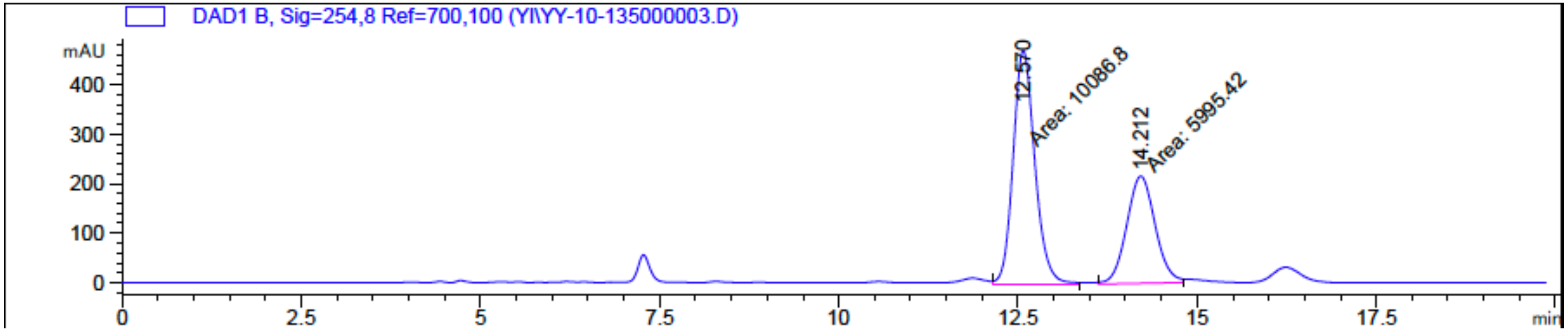

Signal 2: DAD1 B, Sig=254,8 Ref=700,100

Peak #	RetTime [min]	Type	Width [min]	Area [mAU*s]	Height [mAU]	Area %
1	12.570	MM	0.3543	1.00868e4	474.45078	62.7202
2	14.212	MM	0.4602	5995.42041	217.13521	37.2798
Totals :				1.60822e4	691.58598	

Compound 2o: (4*R*,5*S*,6*R*)-6-butyl-5-chloro-4-((*E*)-hex-1-en-1-yl)-2-phenyl-5,6-dihydro-4*H*-1,3-oxazine

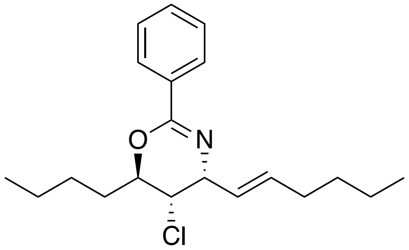

2o

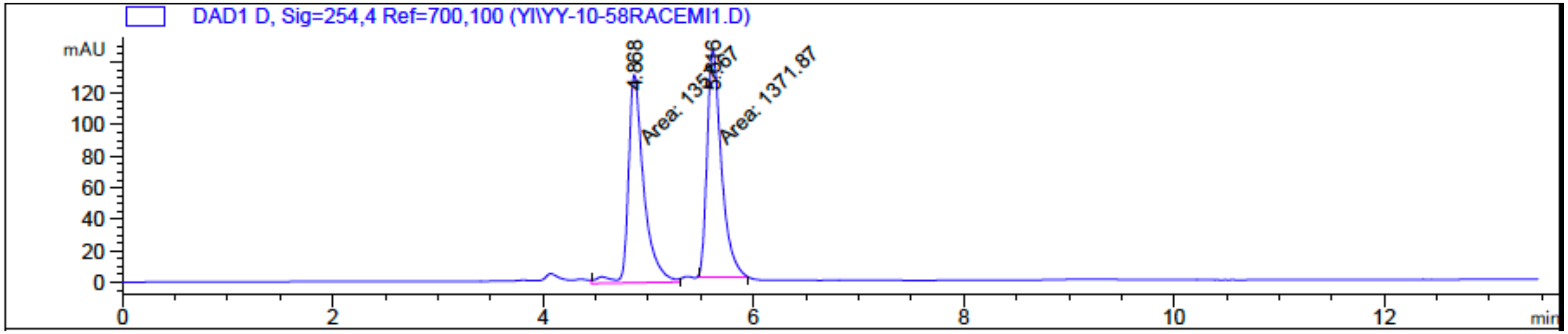

DAD1 D, Sig=254,4 Ref=700,100 (YI\YY-10-58RACEMI1.D)
mAU
120
100
80
60
40
20
0
4.868
Area: 135
5.667
Area: 1371.87
0
2
4
6
8
10
12
min

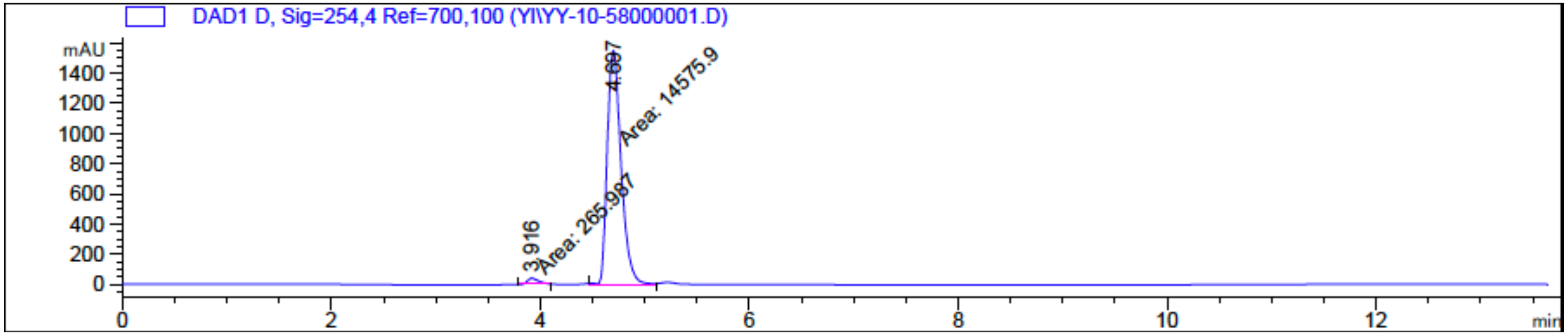

DAD1 D, Sig=254,4 Ref=700,100 (YI\YY-10-58000001.D)
mAU
1400
1200
1000
800
600
400
200
0
3.916
Area: 265.987
4.697
Area: 14575.9
0
2
4
6
8
10
12
min

Signal 3: DAD1 D, Sig=254,4 Ref=700,100

Peak #	RetTime [min]	Type	Width [min]	Area [mAU*s]	Height [mAU]	Area %
1	3.916	MM	0.1179	265.98718	37.61121	1.7921
2	4.697	MM	0.1558	1.45759e4	1559.43445	98.2079
Totals :				1.48419e4	1597.04566	

Compound 2p: (4*R*,5*S*,6*R*)-5-chloro-6-cyclohexyl-4-((*E*)-2-cyclohexylvinyl)-2-phenyl-5,6-dihydro-4*H*-1,3-oxazine

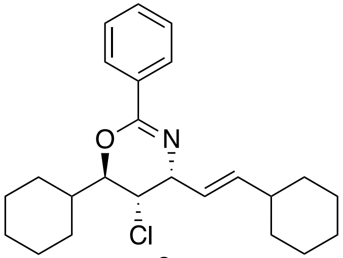

2p

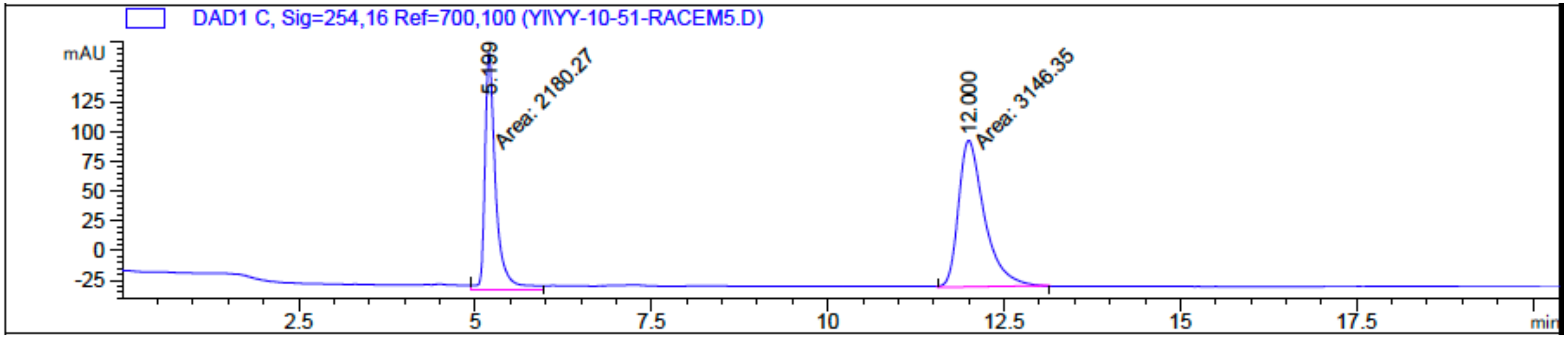

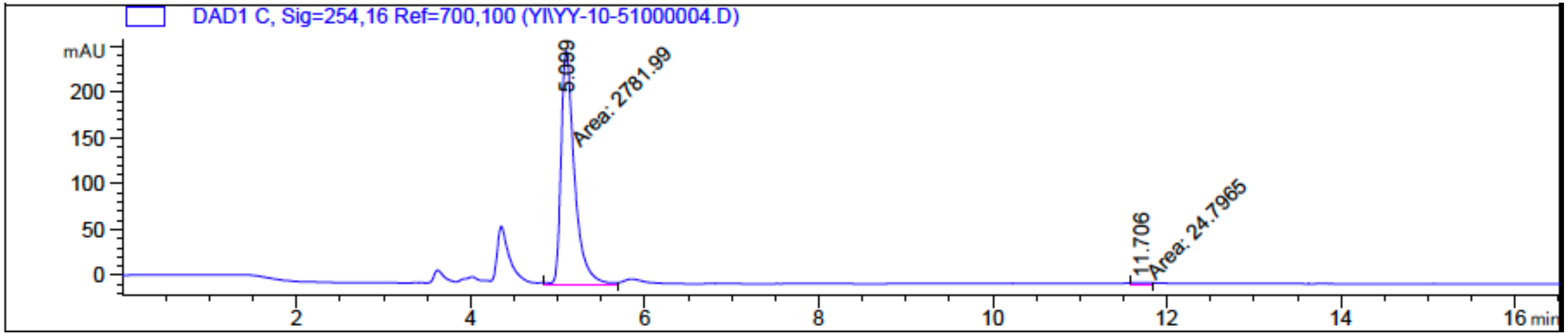

Signal 3: DAD1 C, Sig=254,16 Ref=700,100

Peak #	RetTime [min]	Type	Width [min]	Area [mAU*s]	Height [mAU]	Area %
1	5.099	MM	0.1807	2781.99170	256.59854	99.1166
2	11.706	MM	0.2262	24.79651	1.82666	0.8834

Totals : 2806.78821 258.42521

Compound 2q: (4*R*,5*S*,6*R*)-5-chloro-6-tert-butyl-4-((*E*)-2-tert-butyl-vinyl)-2-phenyl-5,6-dihydro-4*H*-1,3-oxazine

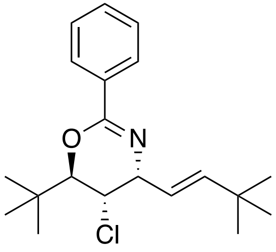

2q

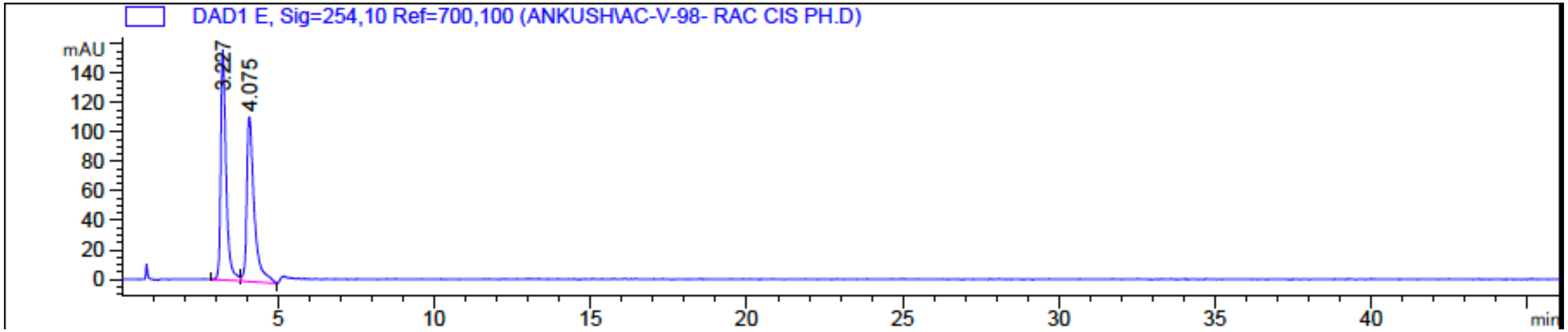

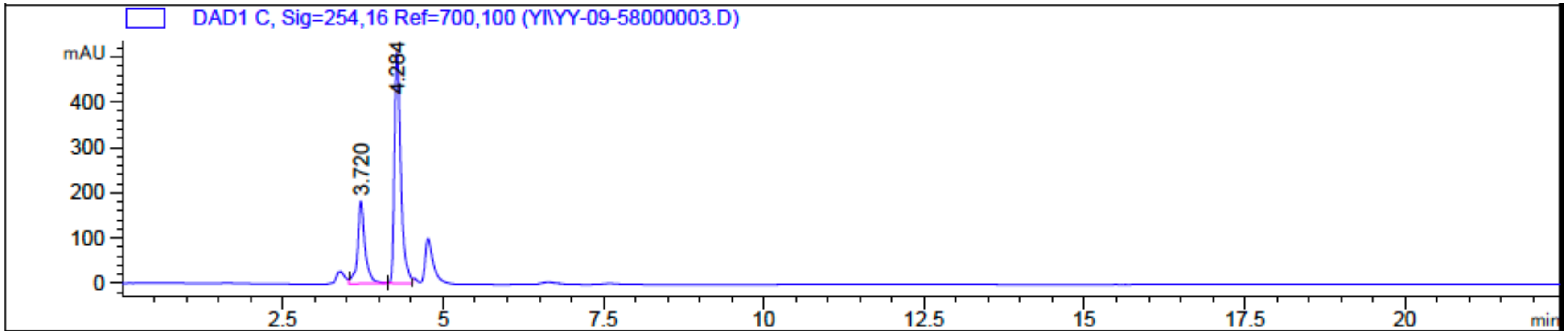

Signal 3: DAD1 C, Sig=254,16 Ref=700,100

Peak #	RetTime [min]	Type	Width [min]	Area [mAU*s]	Height [mAU]	Area %
1	3.720	VB	0.1176	1466.13879	182.71669	29.3686
2	4.284	BV	0.1026	3526.05859	510.20370	70.6314
Totals :				4992.19739	692.92039	

Compound 2r: (4*R*,5*S*,6*R*)-5-chloro-2-isopropyl-6-phenyl-4-((*E*)-styryl)-5,6-dihydro-4*H*-1,3-oxazine

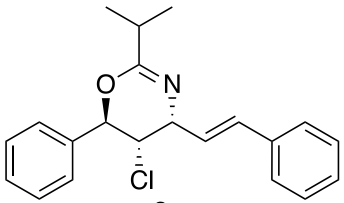

2r

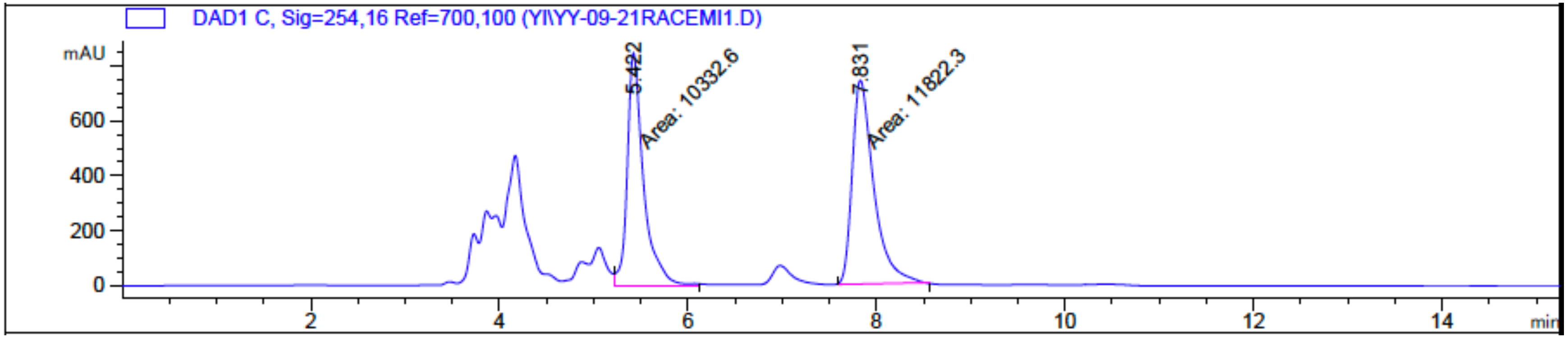
DAD1 C, Sig=254,16 Ref=700,100 (YI\YY-09-21RACEMI1.D)
mAU
600
400
200
0
5.422
Area: 10332.6
7.831
Area: 11822.3
2
4
6
8
10
12
14
min

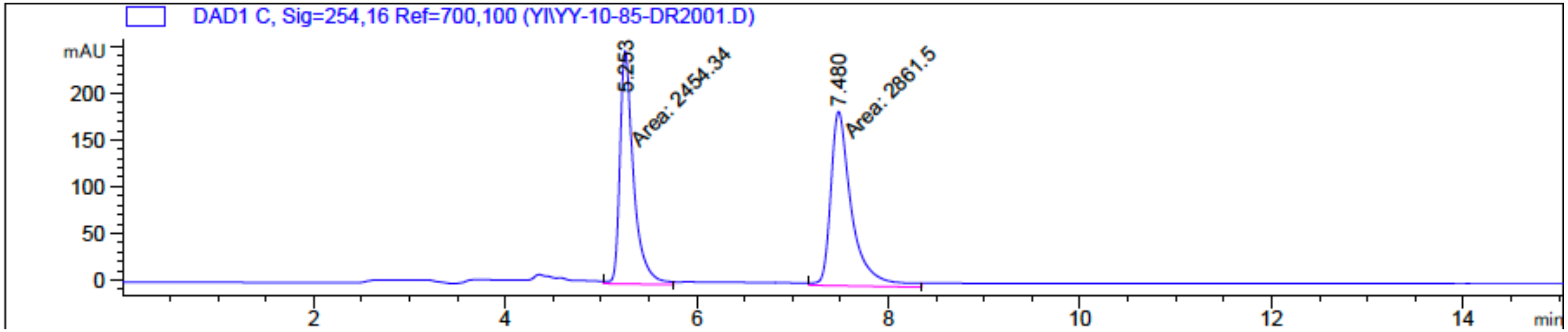
DAD1 C, Sig=254,16 Ref=700,100 (YI\YY-10-85-DR2001.D)
mAU
200
150
100
50
0
5.253
Area: 2454.34
7.480
Area: 2861.5
2
4
6
8
10
12
14
min

Signal 3: DAD1 C, Sig=254,16 Ref=700,100

Peak #	RetTime [min]	Type	Width [min]	Area [mAU*s]	Height [mAU]	Area %
1	5.253	MM	0.1636	2454.34204	249.96342	46.1704
2	7.480	MM	0.2554	2861.49512	186.74763	53.8296

Totals : 5315.83716 436.71106

Compound 2s: (4*R*,5*S*,6*R*)-5-chloro-2-(4-methoxyphenyl)-6-phenyl-4-((*E*)-styryl)-5,6-dihydro-4*H*-1,3-oxazine

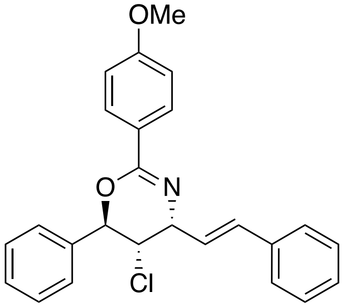

2s

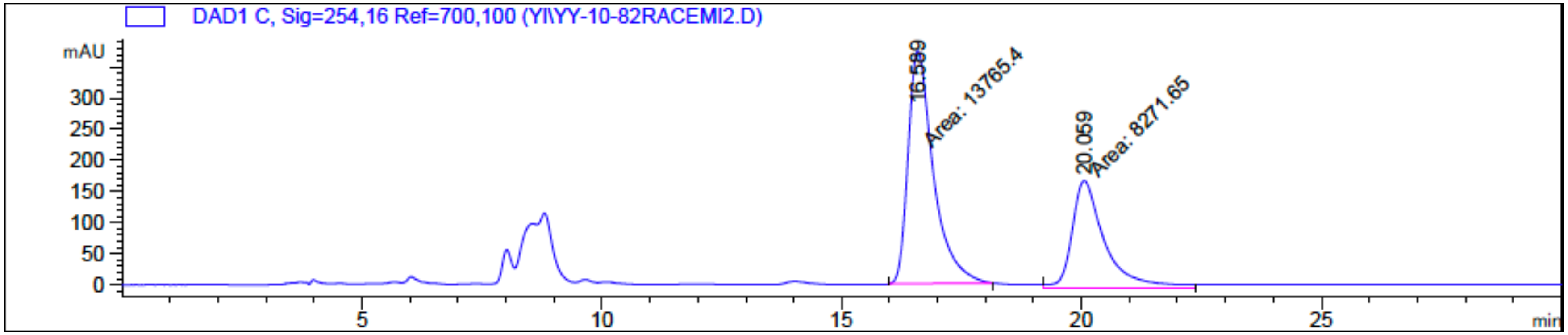

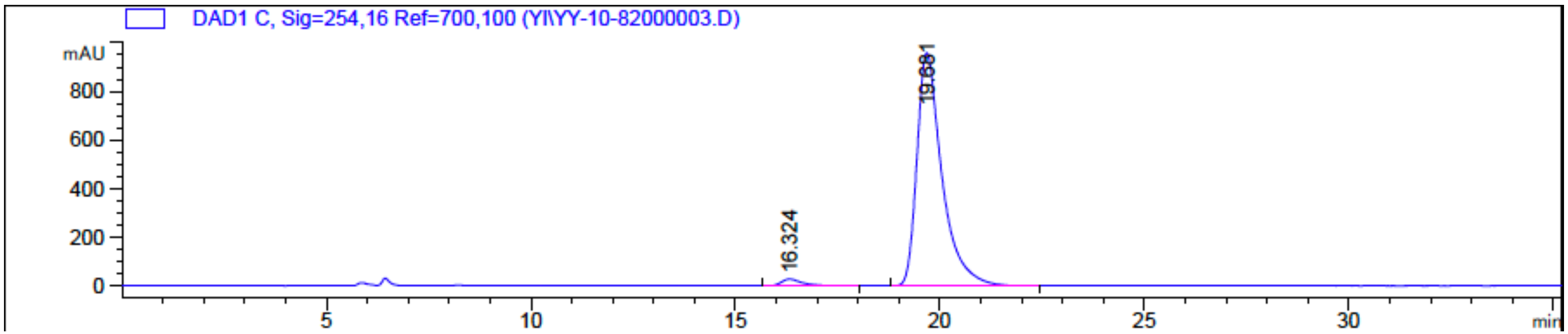


```
Signal 3: DAD1 C, Sig=254,16 Ref=700,100

Peak RetTime Type  Width     Area      Height     Area
  #   [min]        [min]   [mAU*s]     [mAU]       %
----|-------|----|-------|----------|----------|--------|
   1  16.324 BB    0.5208  979.67999   27.68561   2.3538
   2  19.681 BB    0.6251 4.06413e4  957.63330  97.6462

Totals :                  4.16210e4  985.31891
```

Compound 2t: (4*R*,5*S*,6*R*)-5-chloro-2-(4-bromophenyl)-6-phenyl-4-((*E*)-styryl)-5,6-dihydro-4*H*-1,3-oxazine

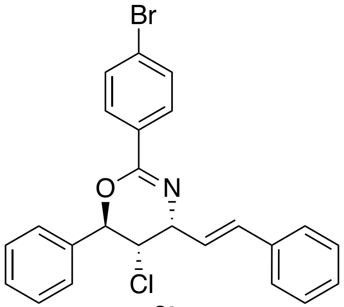

2t

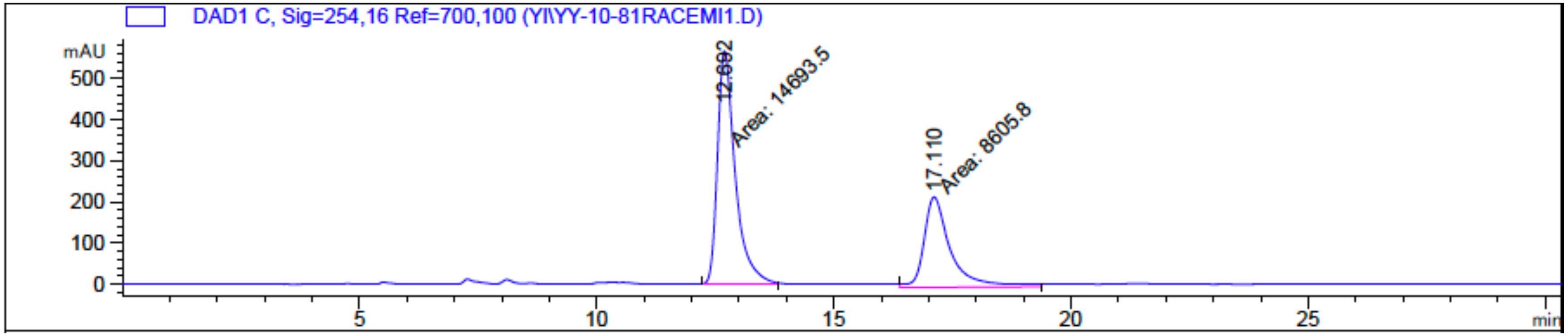

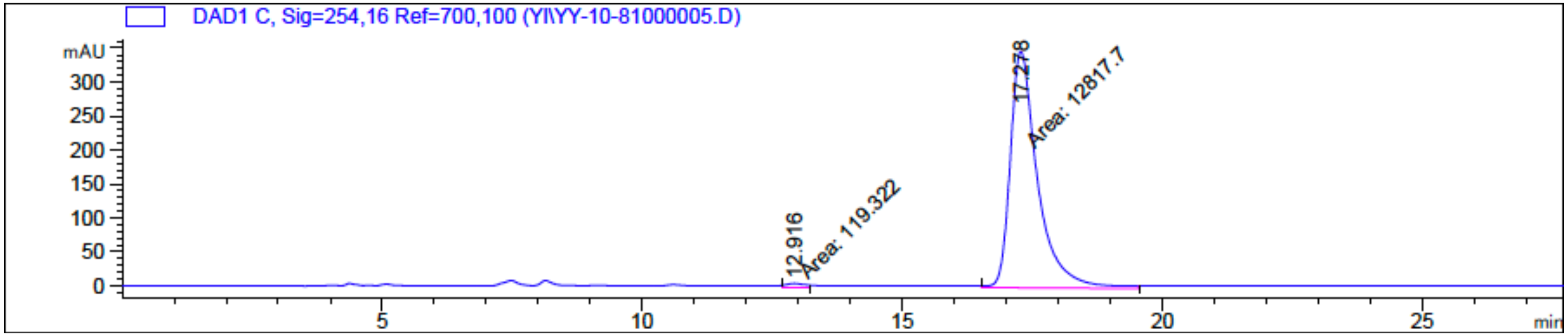

Signal 3: DAD1 C, Sig=254,16 Ref=700,100

Peak #	RetTime [min]	Type	Width [min]	Area [mAU*s]	Height [mAU]	Area %
1	12.916	MM	0.4151	119.32244	4.79092	0.9223
2	17.278	MM	0.6132	1.28177e4	348.38794	99.0777

Totals : 1.29370e4 353.17886

12. NMR SPECTRA:

Compound 1a: *N*-((1*E*,4*E*)-1,5-diphenylpenta-1,4-dien-3-yl)benzamide- ^{1}H NMR (500 MHz, $CDCl_3$); $^{13}C\{^{1}H\}$ NMR (126 MHz, $CDCl_3$)

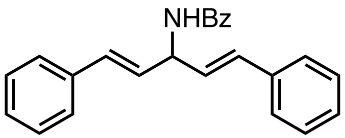

1a

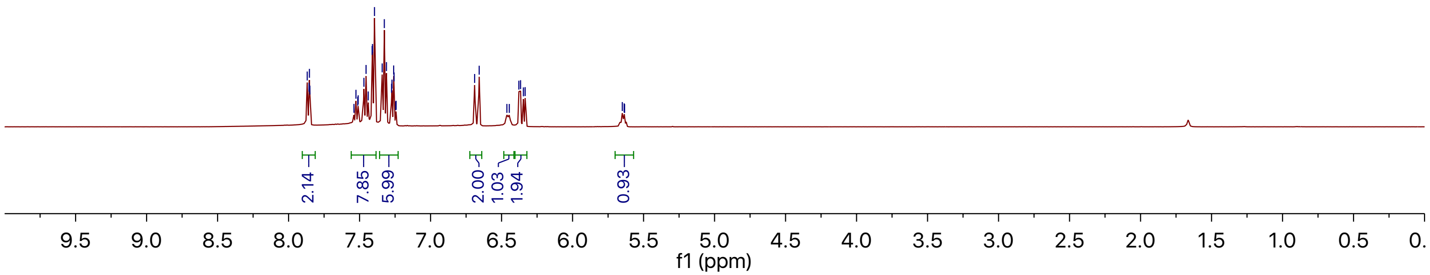

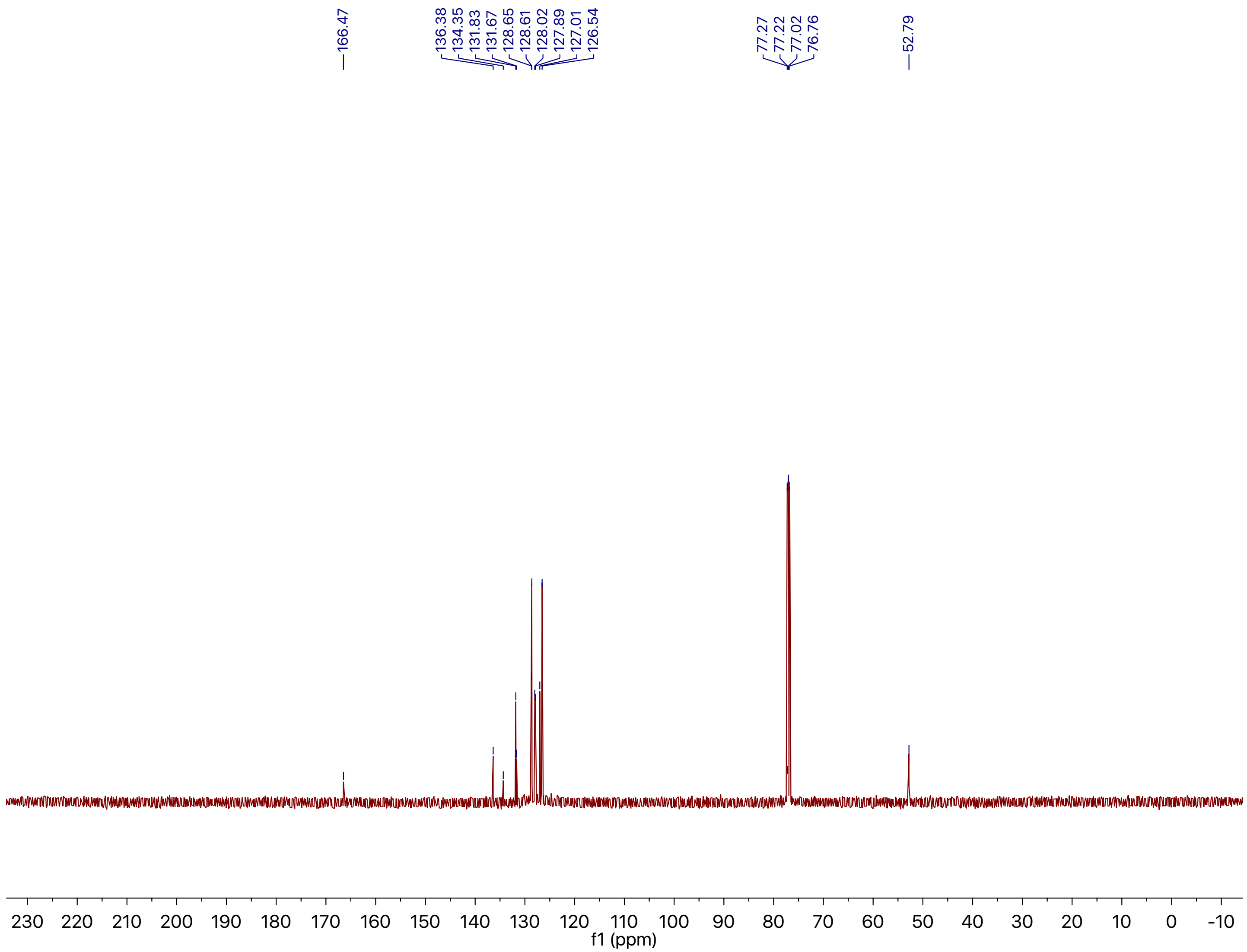
166.47
136.38
134.35
131.83
131.67
128.65
128.61
128.02
127.89
127.01
126.54
77.27
77.22
77.02
76.76
52.79
230 220 210 200 190 180 170 160 150 140 130 120 110 100 90 80 70 60 50 40 30 20 10 0 -10
f1 (ppm)

Compound 1b: *N*-((1*E*,4*E*)-1,5-bis(4-chlorophenyl)penta-1,4-dien-3-yl)benzamide- ^{1}H NMR (500 MHz, $CDCl_3$); ^{13}C{^{1}H} NMR (126 MHz, $CDCl_3$)

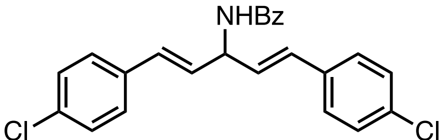

1b

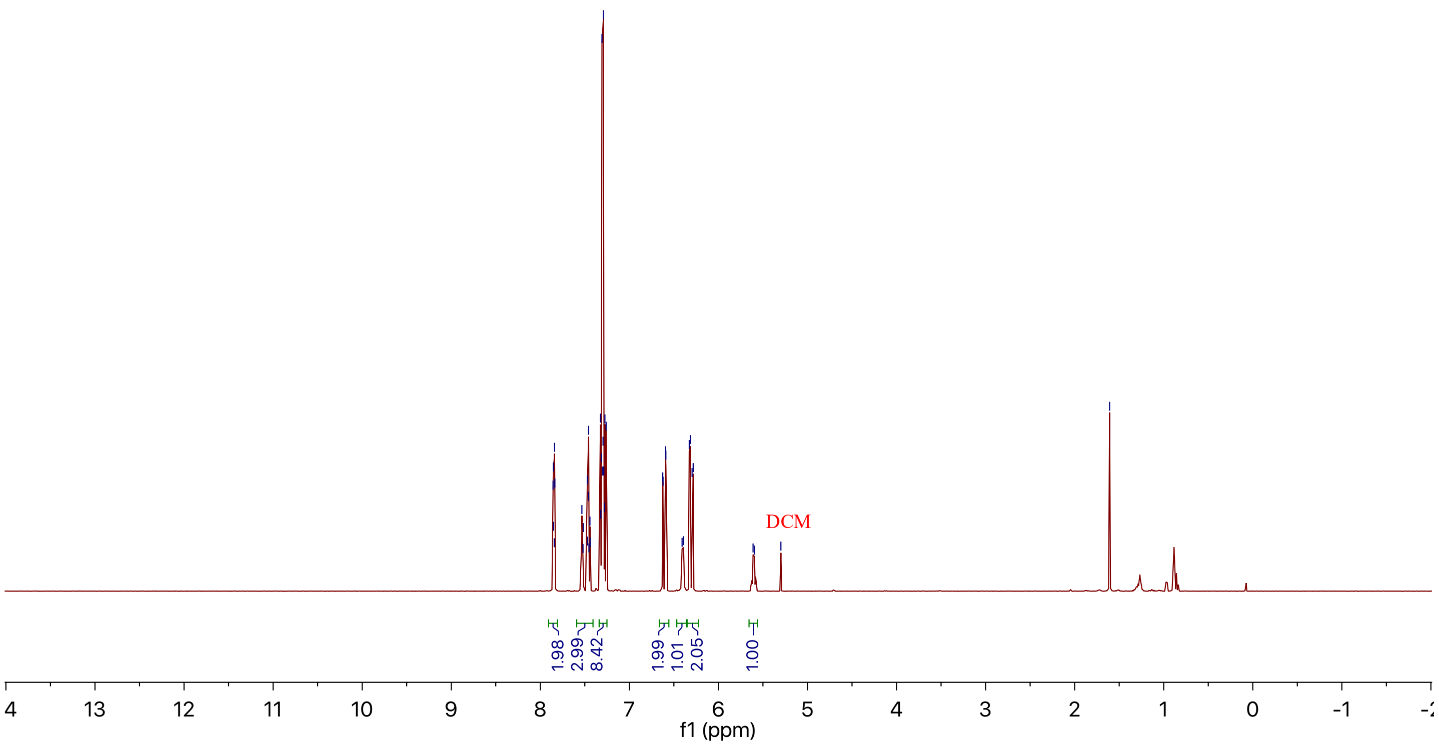

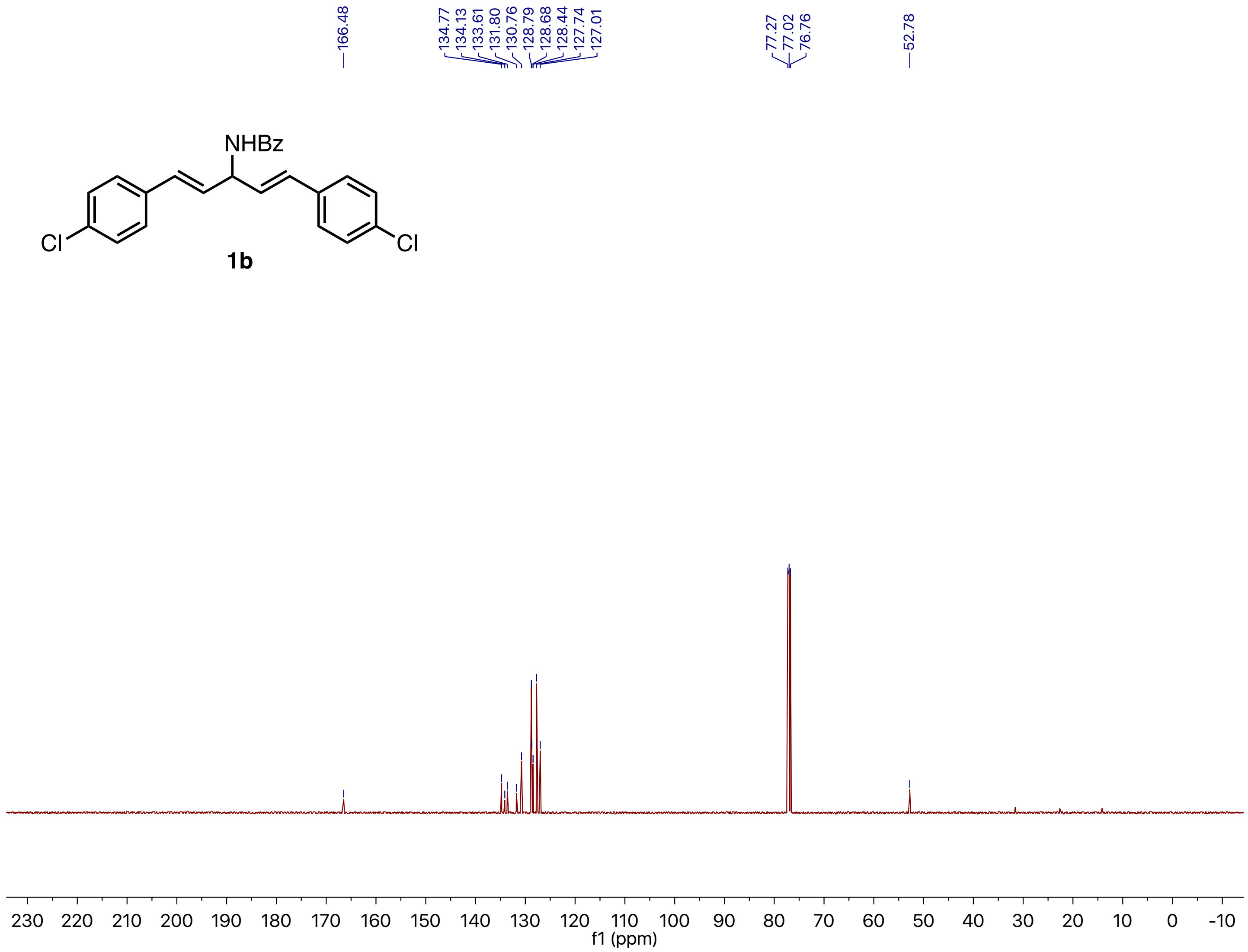
166.48
134.77
134.13
133.61
131.80
130.76
128.79
128.68
128.44
127.74
127.01
77.27
77.02
76.76
52.78
NHBz
Cl
Cl
1b
230 220 210 200 190 180 170 160 150 140 130 120 110 100 90 80 70 60 50 40 30 20 10 0 -10
f1 (ppm)

Compound 1c*:* *N*-((1*E*,4*E*)-1,5-bis(4-bromophenyl)penta-1,4-dien-3-yl)benzamide **1c**-^{1}H NMR (500 MHz, $CDCl_3$); $^{13}C\{^{1}H\}$ NMR (126 MHz, $CDCl_3$)

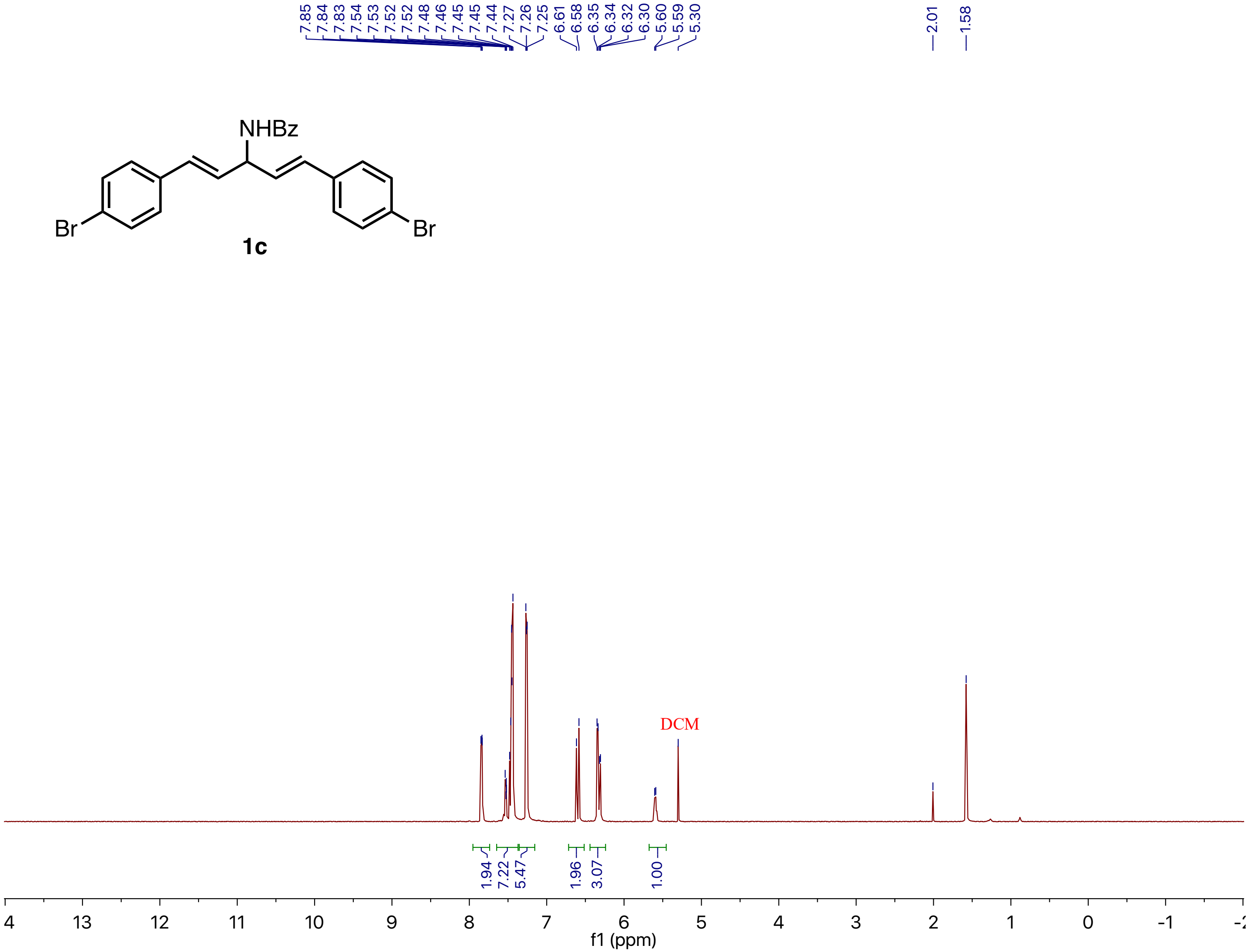

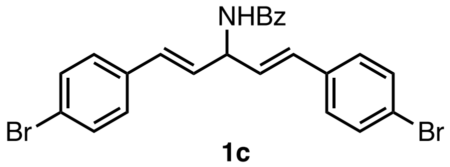
NHBz
Br
Br
1c

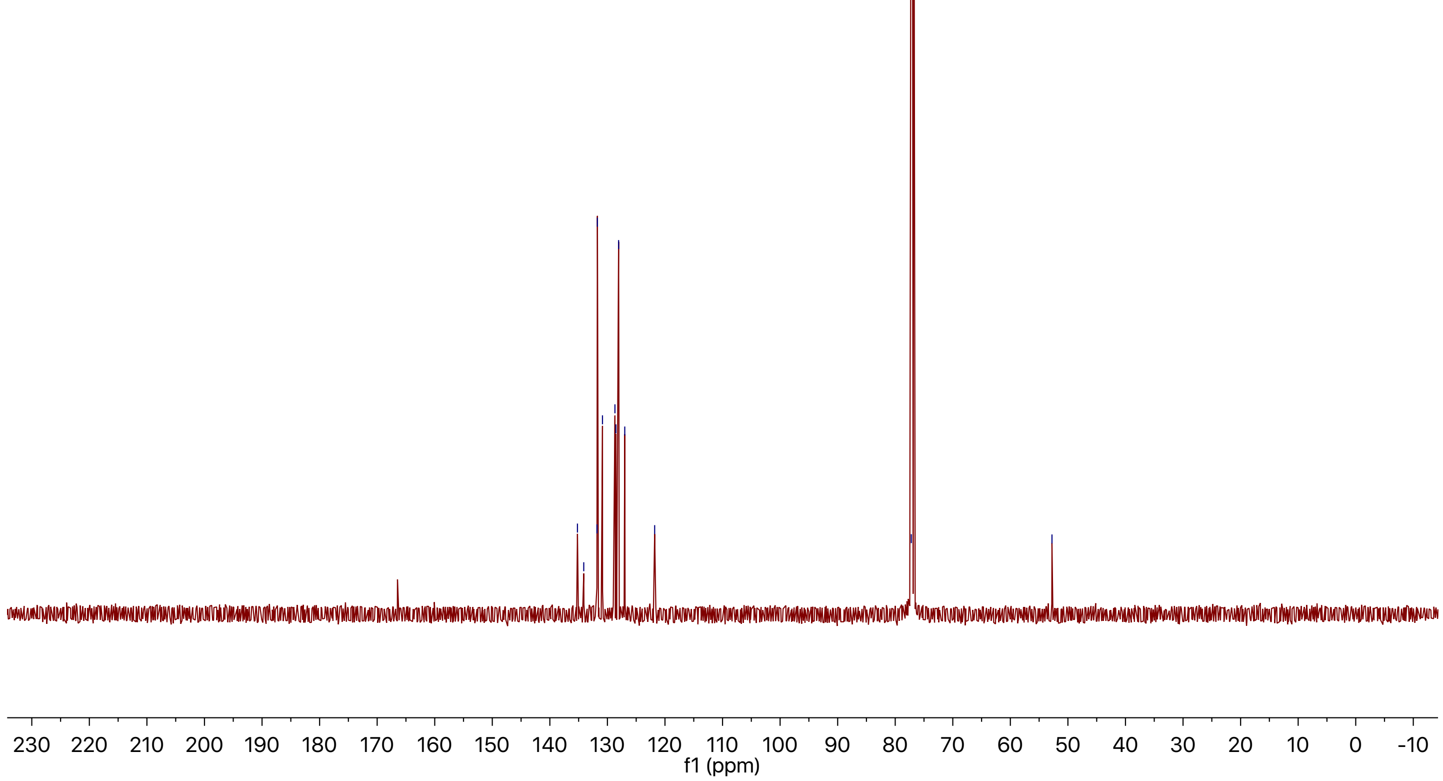
135.20
134.11
131.81
131.74
130.86
128.69
128.53
128.05
126.99
121.79
77.26
77.21
77.00
76.75
52.77
230 220 210 200 190 180 170 160 150 140 130 120 110 100 90 80 70 60 50 40 30 20 10 0 -10
f1 (ppm)

Compound 1d: *N*-((1*E*,4*E*)-1,5-bis(3-bromophenyl)penta-1,4-dien-3-yl)benzamide- ^{1}H NMR (500 MHz, $CDCl_3$); ^{13}C{^{1}H} NMR (126 MHz, $CDCl_3$)

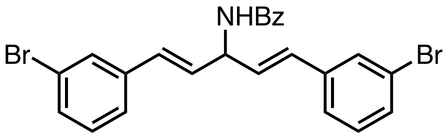

1d

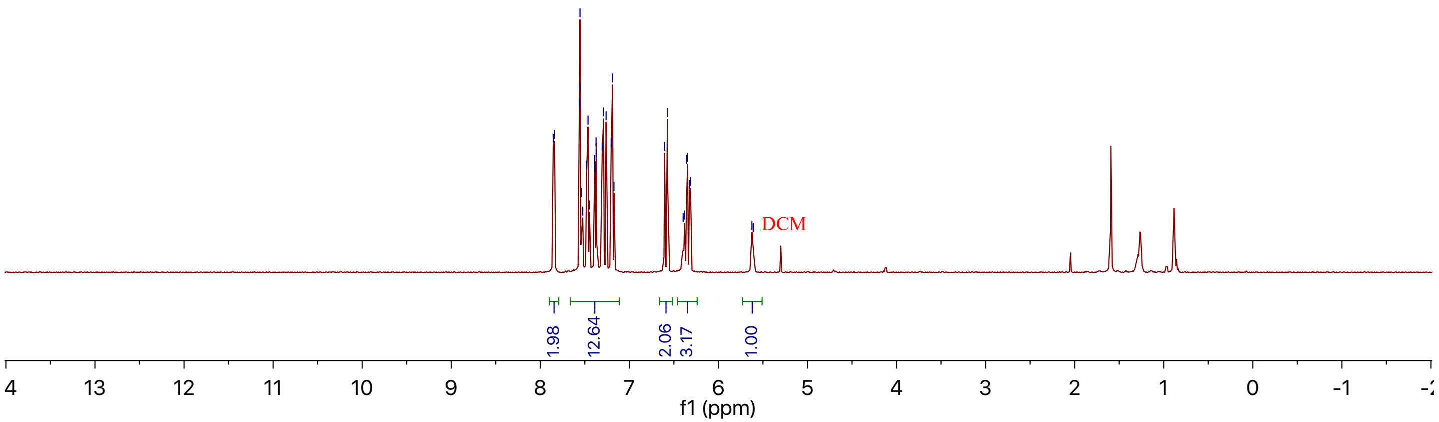

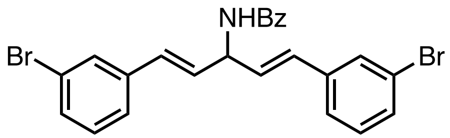
NHBz
Br
Br

1d

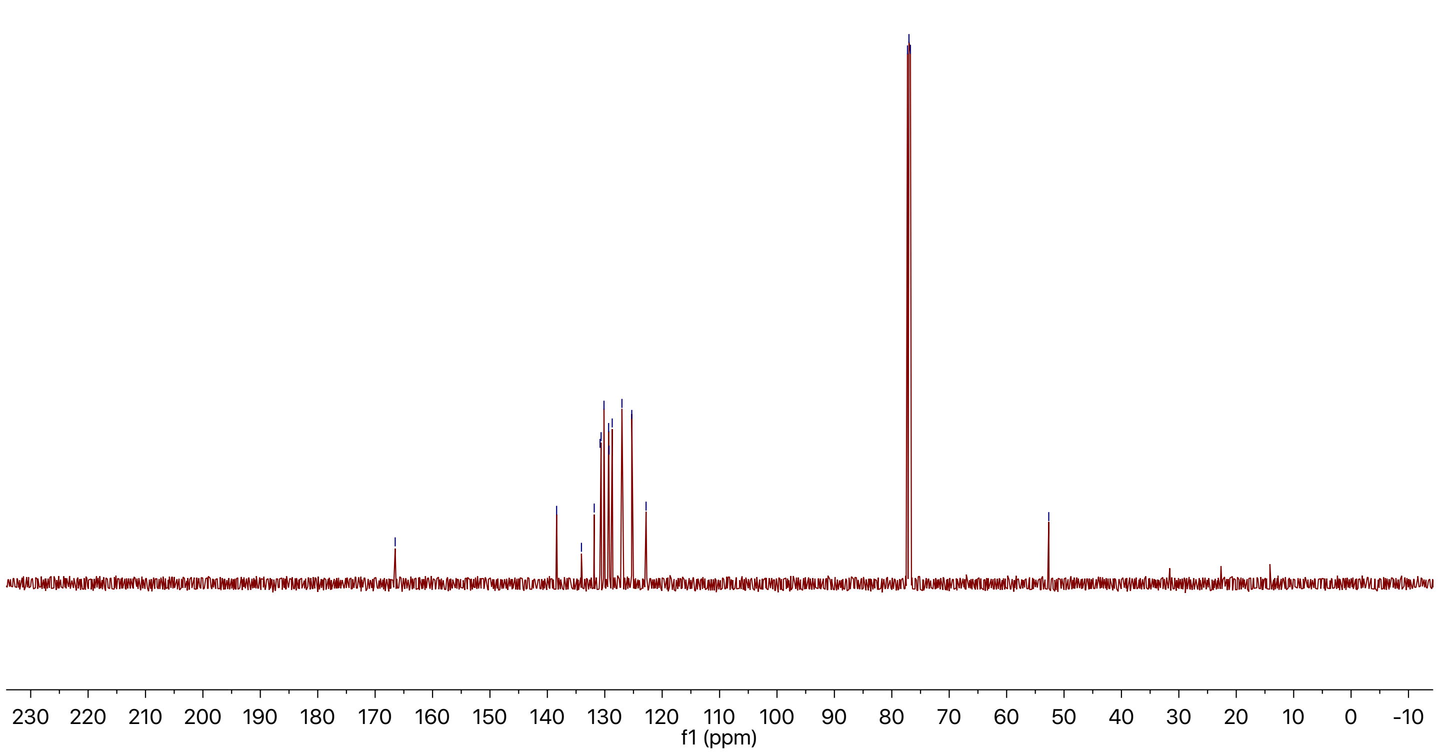
166.51
138.39
134.06
131.84
130.82
130.64
130.14
129.33
129.25
128.70
127.01
125.30
122.80
77.27
77.01
76.76
52.66
230 220 210 200 190 180 170 160 150 140 130 120 110 100 90 80 70 60 50 40 30 20 10 0 -10
f1 (ppm)

Compound 1e: *N*-((1*E*,4*E*)-1,5-bis(2-fluorophenyl)penta-1,4-dien-3-yl)benzamide- ^{1}H NMR (500 MHz, $CDCl_3$); $^{13}C\{^1H\}$ NMR (126 MHz, $CDCl_3$)

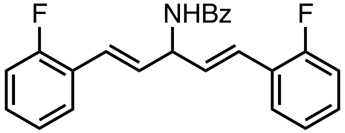

1e

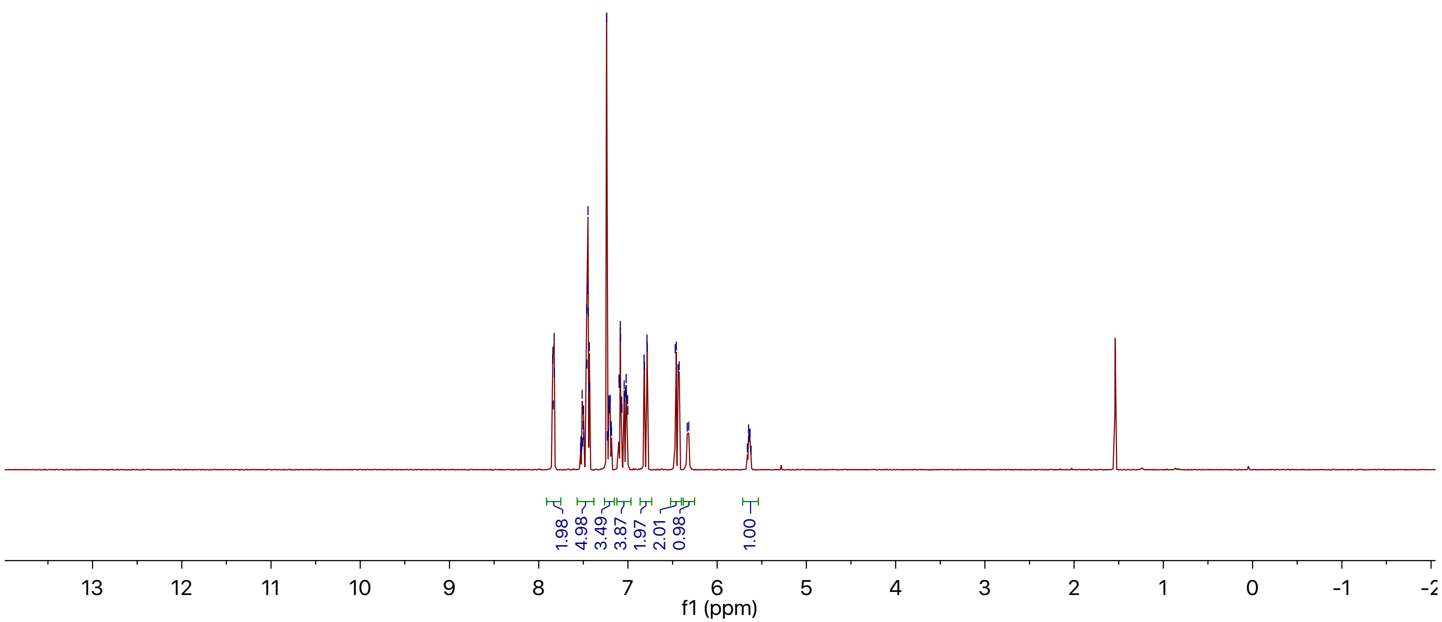

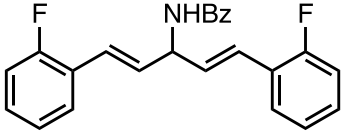
F
NHBz
F

1e

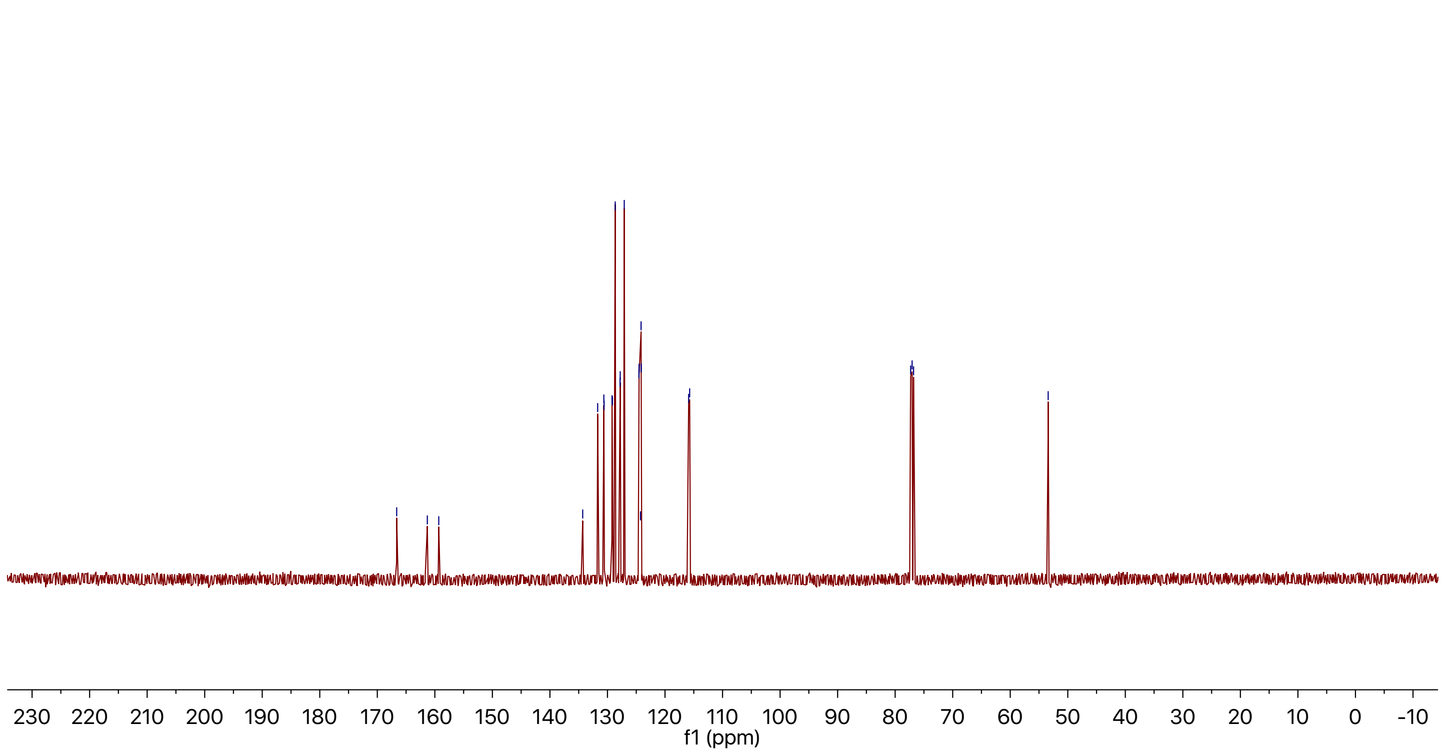
166.63
161.31
159.32
134.30
131.70
130.63
130.58
129.21
129.14
128.65
127.81
127.78
127.08
124.53
124.51
124.25
124.16
124.13
115.89
115.71
77.31
77.05
76.80
53.41
230 220 210 200 190 180 170 160 150 140 130 120 110 100 90 80 70 60 50 40 30 20 10 0 -10
f1 (ppm)

Compound 1f: *N*-((1*E*,4*E*)-1,5-bis(4-fluorophenyl)penta-1,4-dien-3-yl)benzamide- ^{1}H NMR (500 MHz, $CDCl_3$); $^{13}C\{^{1}H\}$ NMR (126 MHz, $CDCl_3$)

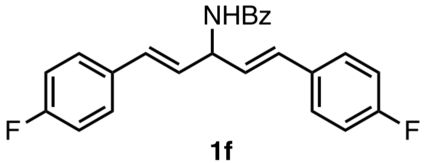

1f

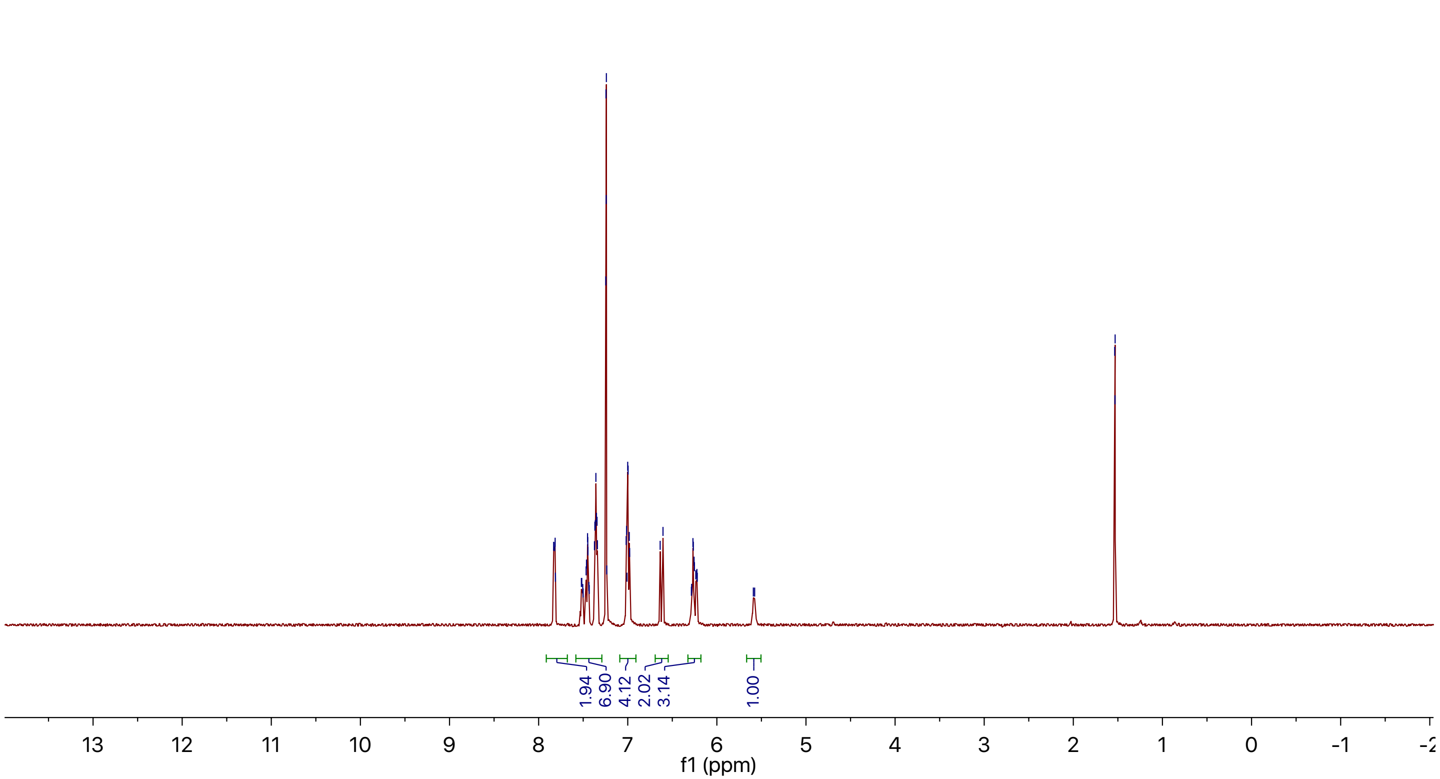

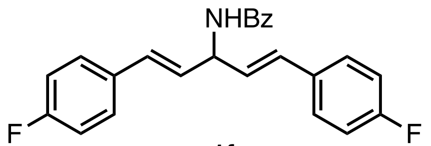

NHBz
F
F

1f

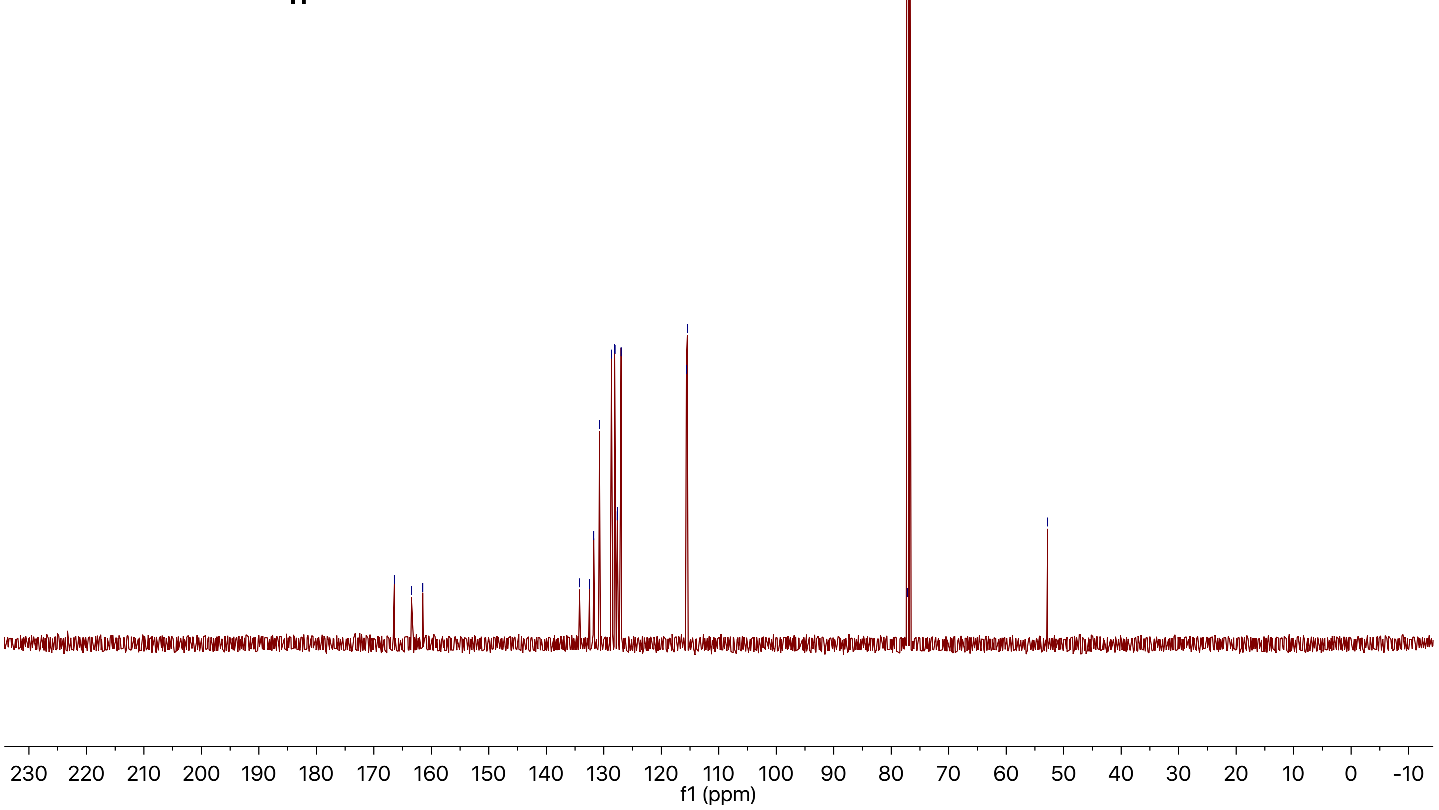

166.44
163.46
161.49
134.22
132.50
132.47
131.75
130.75
128.67
128.11
128.05
127.66
127.65
126.98
115.64
115.47
77.26
77.21
77.01
76.76
52.80
230 220 210 200 190 180 170 160 150 140 130 120 110 100 90 80 70 60 50 40 30 20 10 0 -10
f1 (ppm)

Compound 1g: *N*-((1*E*,4*E*)-1,5-bis(2,6-dichlorophenyl)penta-1,4-dien-3-yl)benzamide- ^{1}H NMR (500 MHz, $CDCl_3$), $^{13}C\{^{1}H\}$ NMR (126 MHz, $CDCl_3$)

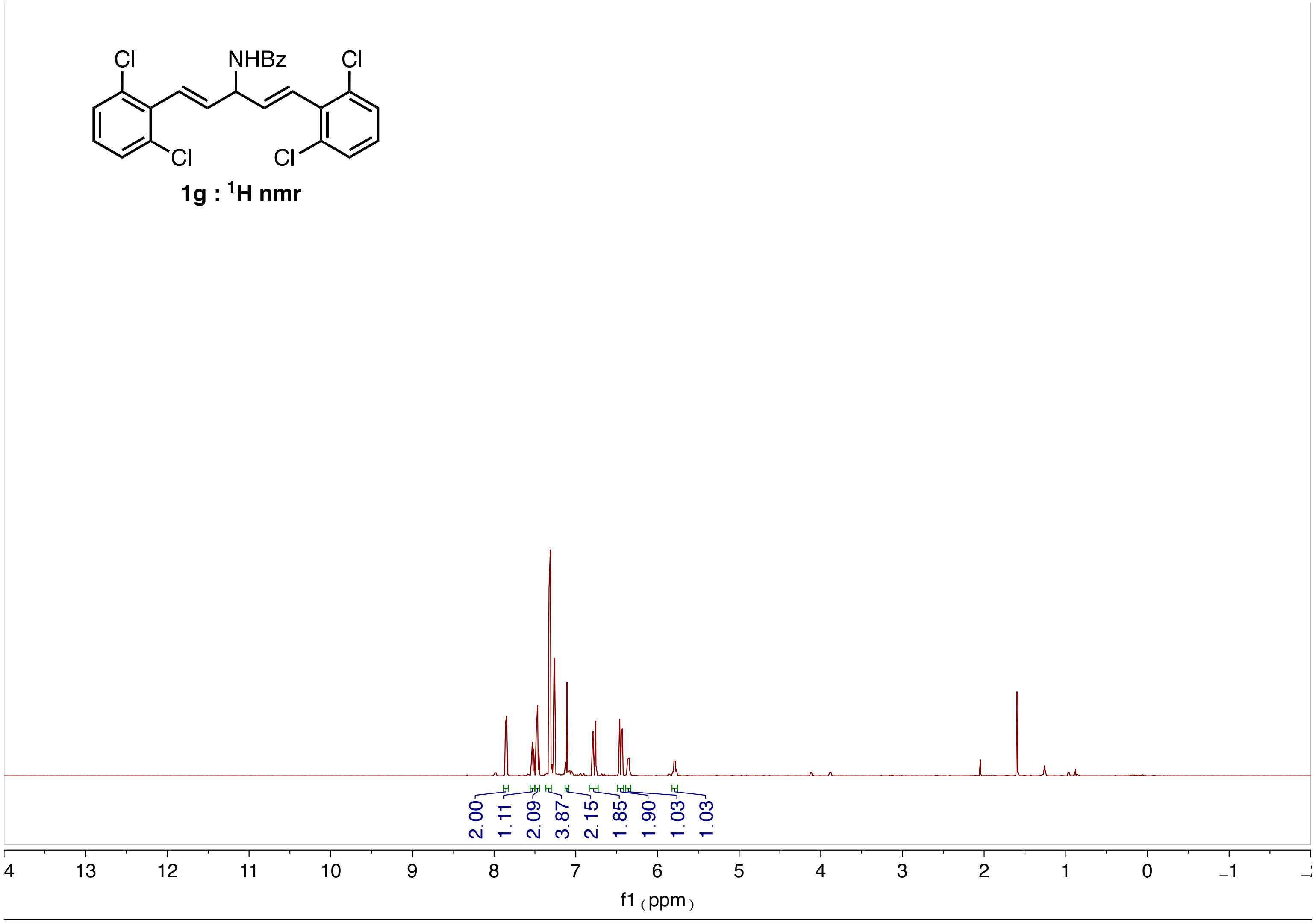

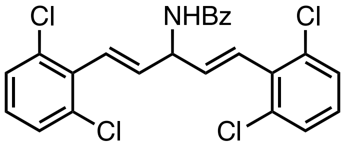
Cl
NHBz
Cl
Cl
Cl

1g : ^{13}C nmr

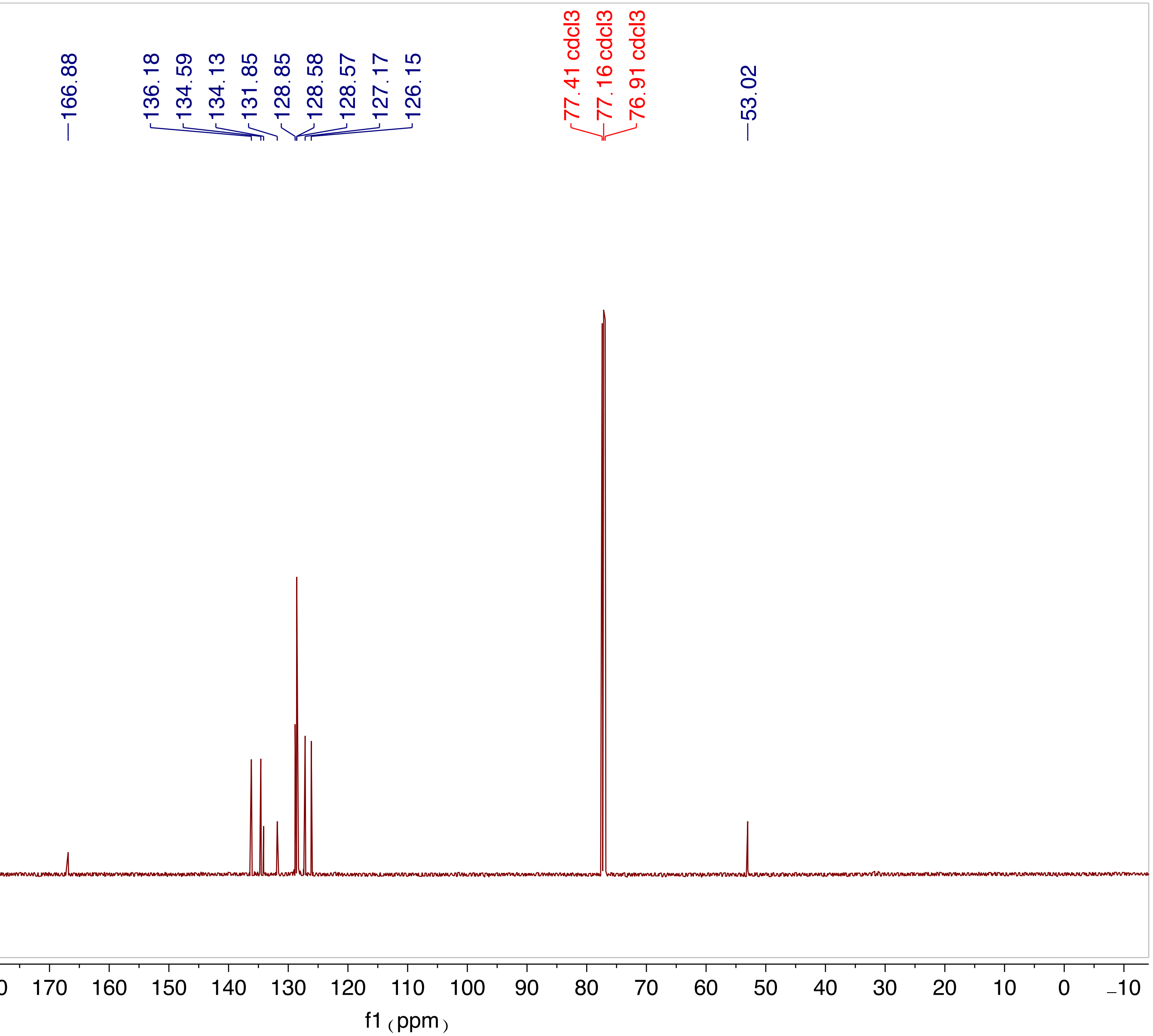
166.88
136.18
134.59
134.13
131.85
128.85
128.58
128.57
127.17
126.15
77.41 cdcl3
77.16 cdcl3
76.91 cdcl3
53.02
230 220 210 200 190 180 170 160 150 140 130 120 110 100 90 80 70 60 50 40 30 20 10 0 -10
f1 (ppm)

Compound 1h: *N*-((1*E*,4*E*)-1,5-bis(4-(trifluoromethyl)phenyl)penta-1,4-dien-3-yl)benzamide- ^{1}H NMR (500 MHz, $CDCl_3$); $^{13}C\{^{1}H\}$ NMR (126 MHz, $CDCl_3$)

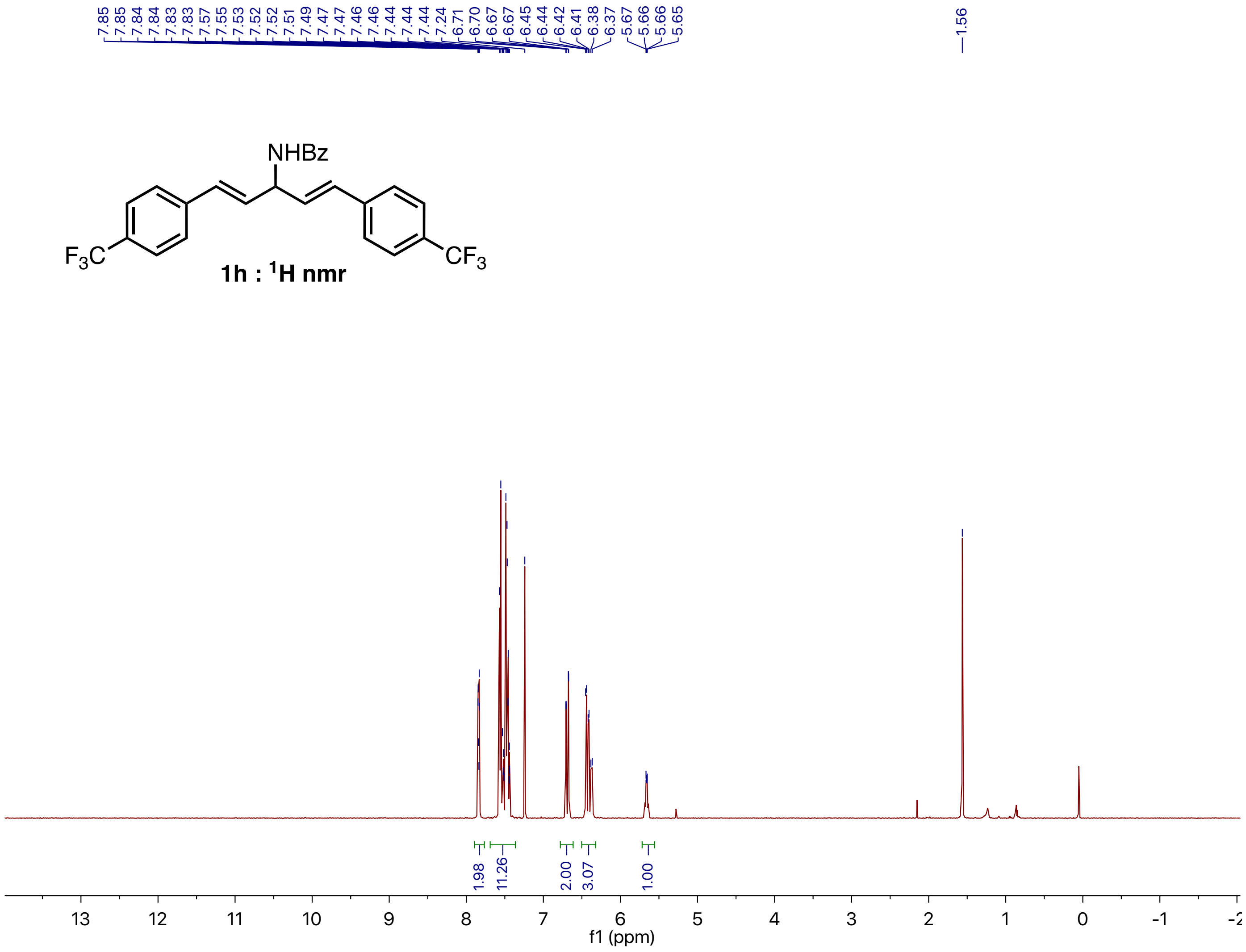

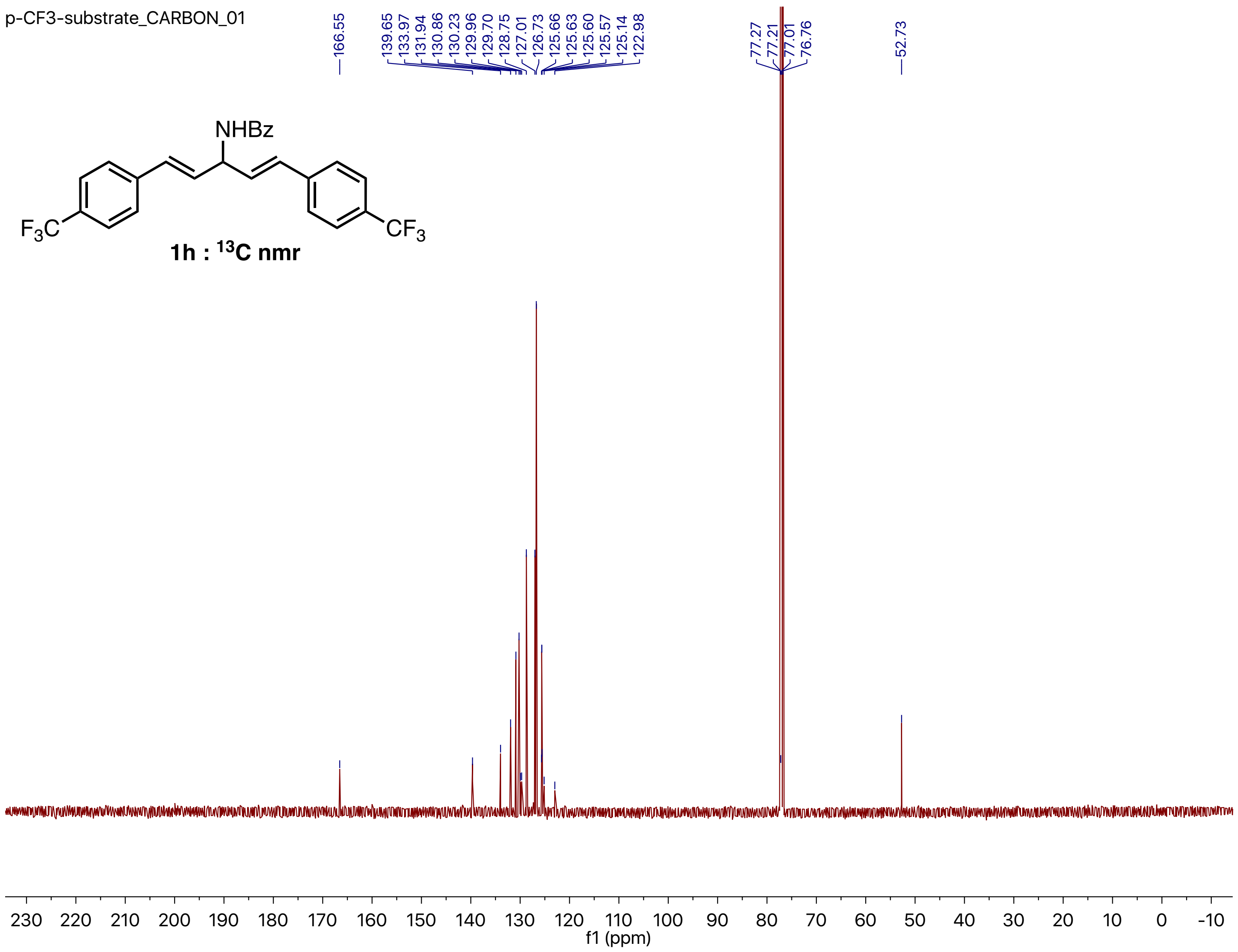
p-CF3-substrate_CARBON_01
166.55
139.65
133.97
131.94
130.86
130.23
129.96
129.70
128.75
127.01
126.73
125.66
125.63
125.60
125.57
125.14
122.98
77.27
77.21
77.01
76.76
52.73
NHBz
F_3C
CF_3
1h : ^{13}C nmr
230 220 210 200 190 180 170 160 150 140 130 120 110 100 90 80 70 60 50 40 30 20 10 0 -10
f1 (ppm)

Compound 1i: *N*-((1*E*,4*E*)-1,5-bis(3-methoxyphenyl)penta-1,4-dien-3-yl)benzamide- ^{1}H NMR (500 MHz, $CDCl_3$); $^{13}C\{^{1}H\}$ NMR (126 MHz, $CDCl_3$)

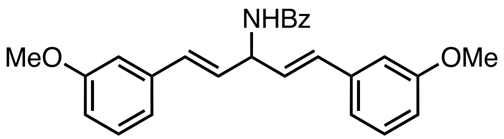

1i : ^{1}H nmr

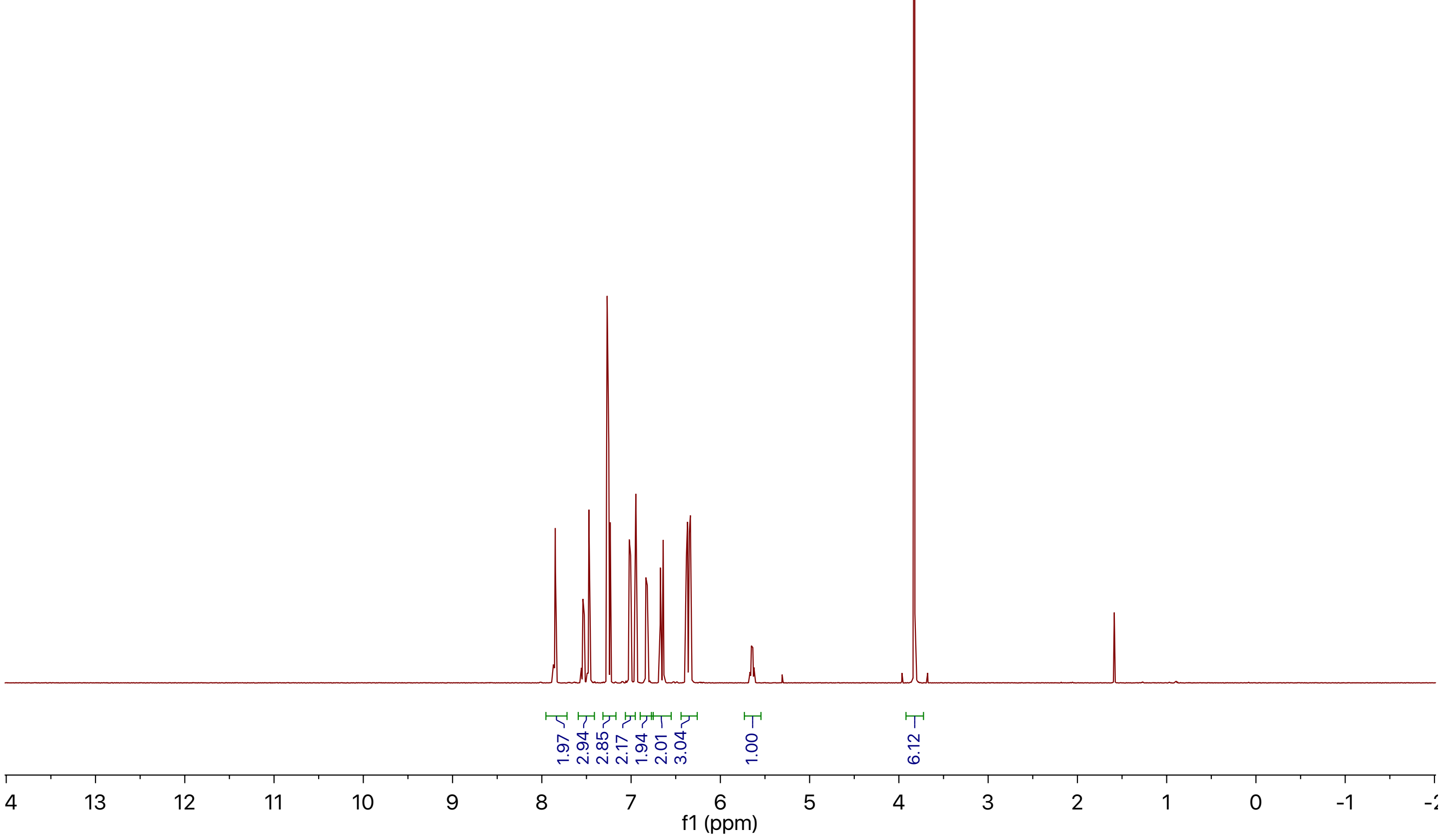

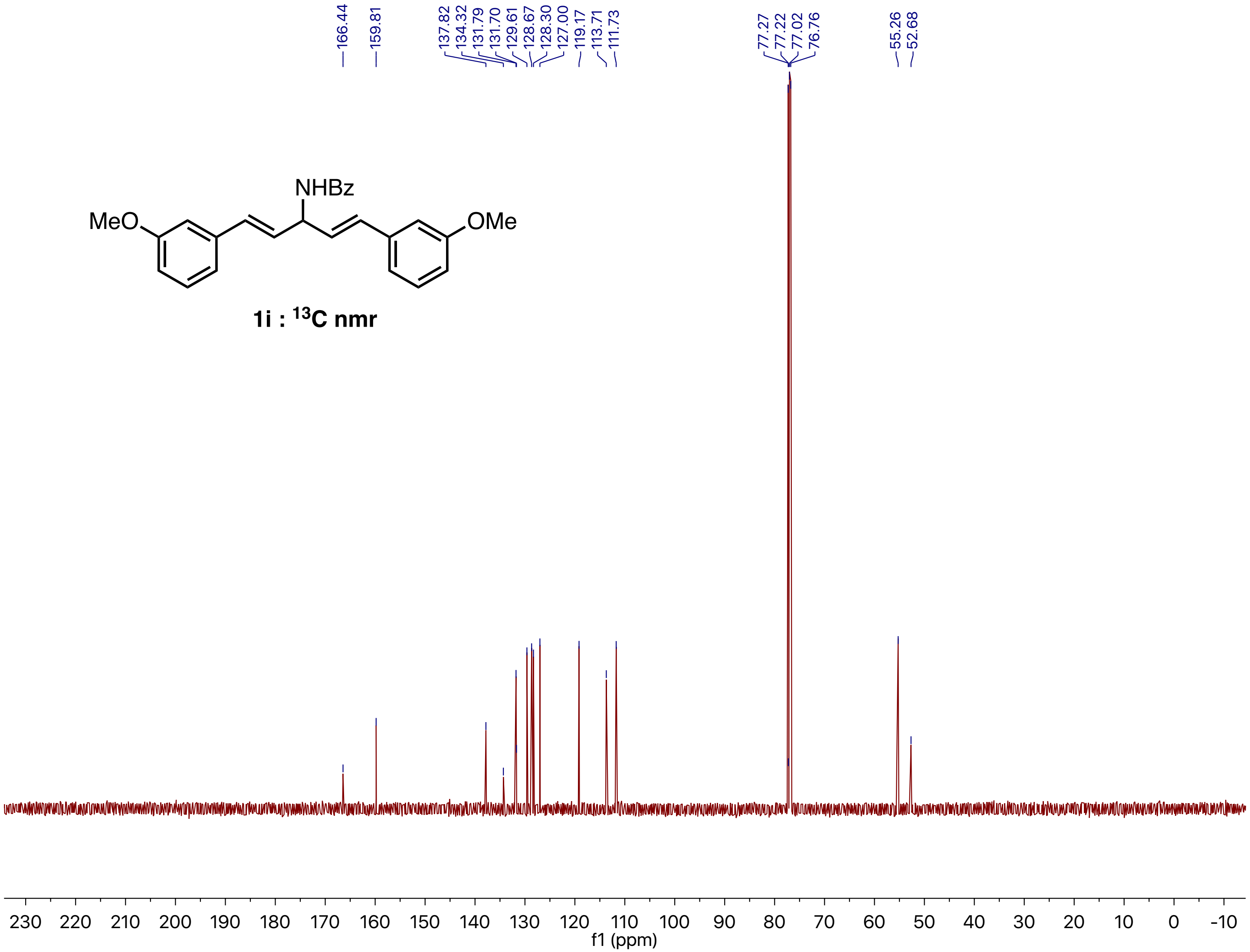
NHBz
MeO
OMe
166.44
159.81
137.82
134.32
131.79
131.70
129.61
128.67
128.30
127.00
119.17
113.71
111.73
77.27
77.22
77.02
76.76
55.26
52.68
230 220 210 200 190 180 170 160 150 140 130 120 110 100 90 80 70 60 50 40 30 20 10 0 -10
f1 (ppm)

1i : ^{13}C nmr

Compound 1j: *N-((1E,4E)-1,5-di-o-tolylpenta-1,4-dien-3-yl)benzamide*- ^{1}H NMR (500 MHz, $CDCl_3$); ^{13}C{^{1}H} NMR (126 MHz, $CDCl_3$)

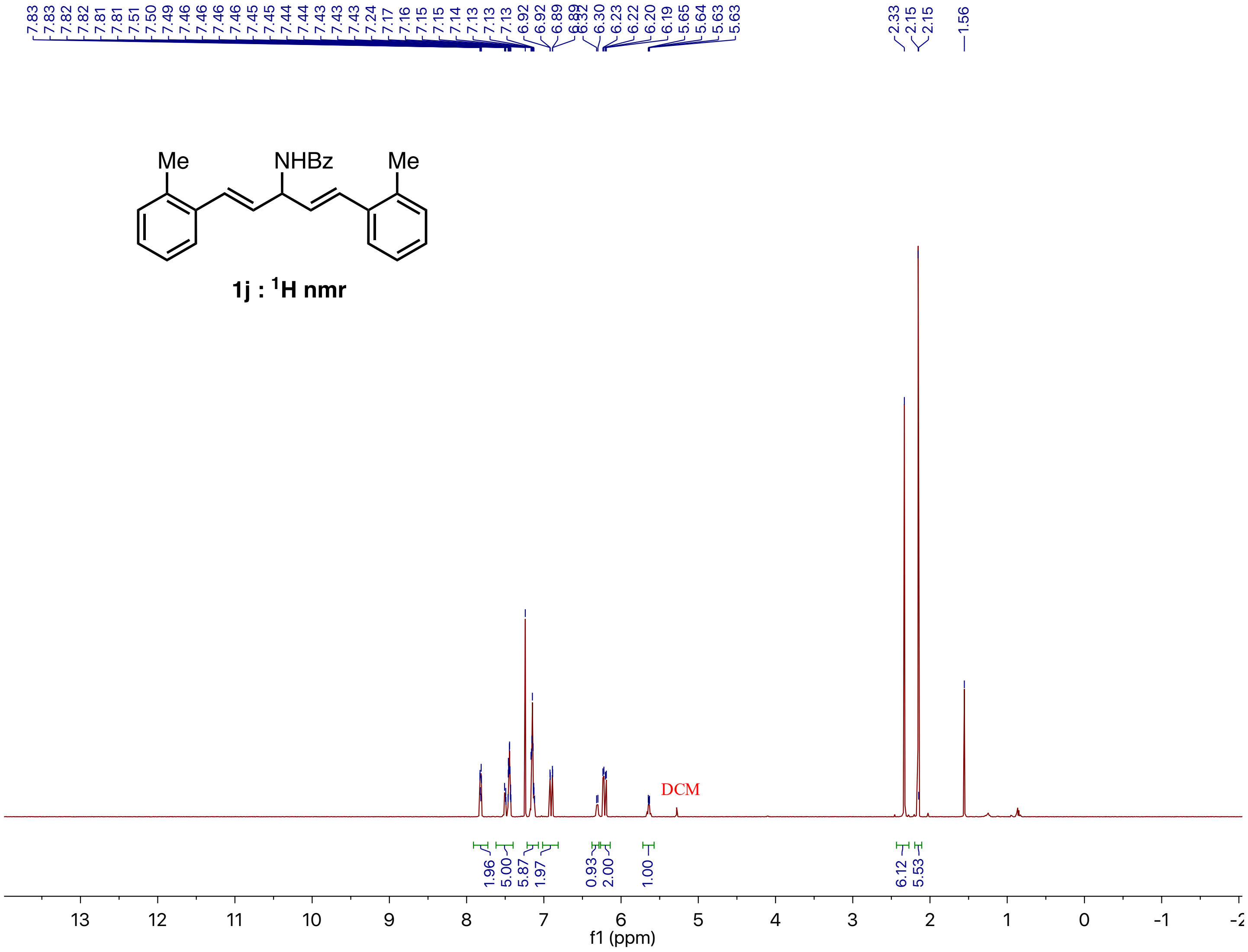

o-Me-substrate_CARBON_01

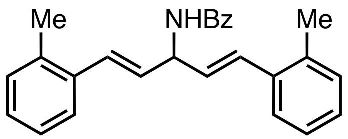

1j : ^{13}C nmr

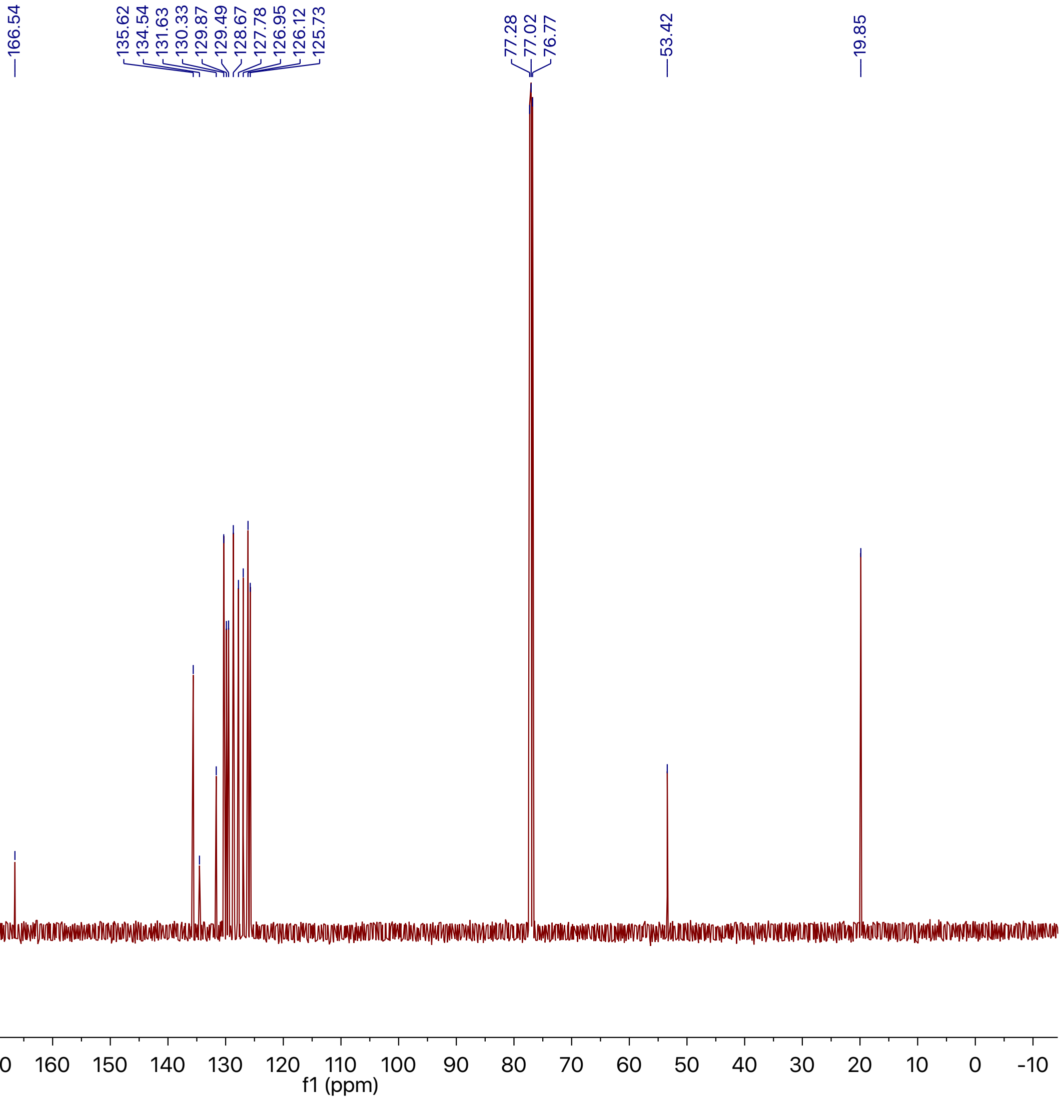

Compound 1k: *N-((1E,4E)-1,5-di-p-tolylpenta-1,4-dien-3-yl)benzamide*- ^{1}H NMR (500 MHz, $CDCl_3$); $^{13}C\{^1H\}$ NMR (126 MHz, $CDCl_3$)

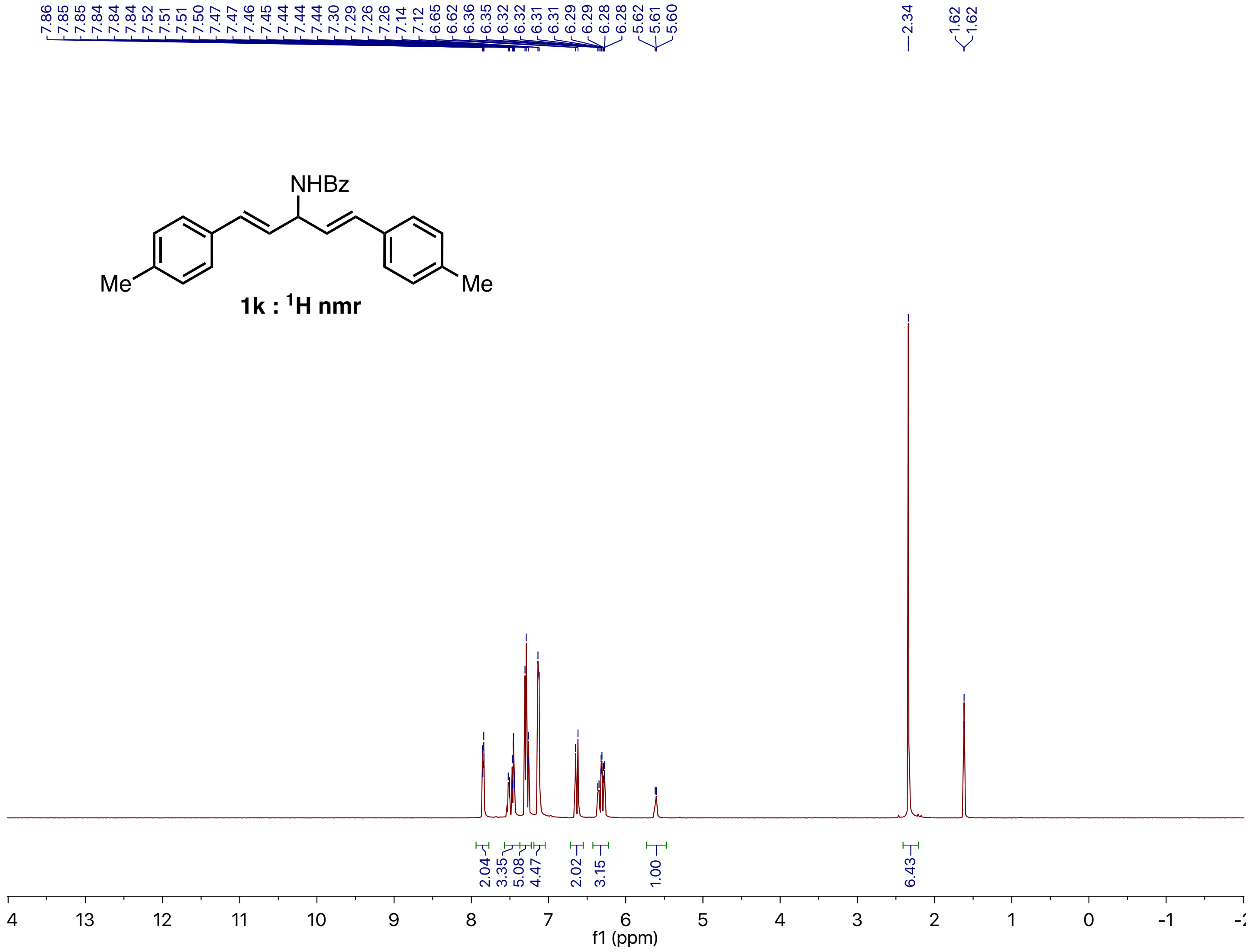

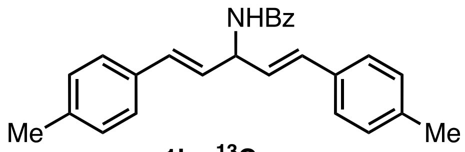
NHBz
Me
Me

1k : ^{13}C nmr

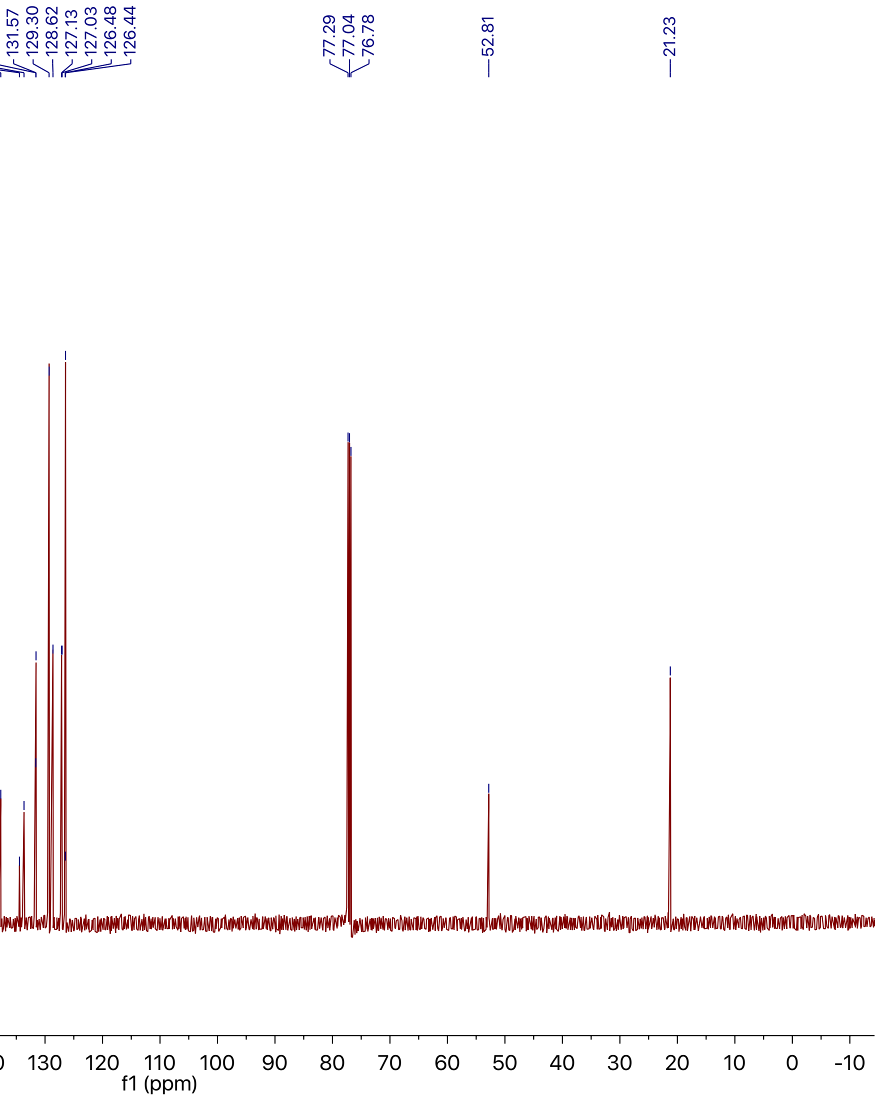
166.44
137.70
134.45
133.66
131.60
131.57
129.30
128.62
127.13
127.03
126.48
126.44
77.29
77.04
76.78
52.81
21.23
230 220 210 200 190 180 170 160 150 140 130 120 110 100 90 80 70 60 50 40 30 20 10 0 -10
f1 (ppm)

Compound 1l: *N*-((1*E*,4*E*)-1,5-bis(4-methoxyphenyl)penta-1,4-dien-3-yl)benzamide **1l**- ^{1}H NMR (500 MHz, $CDCl_3$); $^{13}C\{^{1}H\}$ NMR (126 MHz, $CDCl_3$)

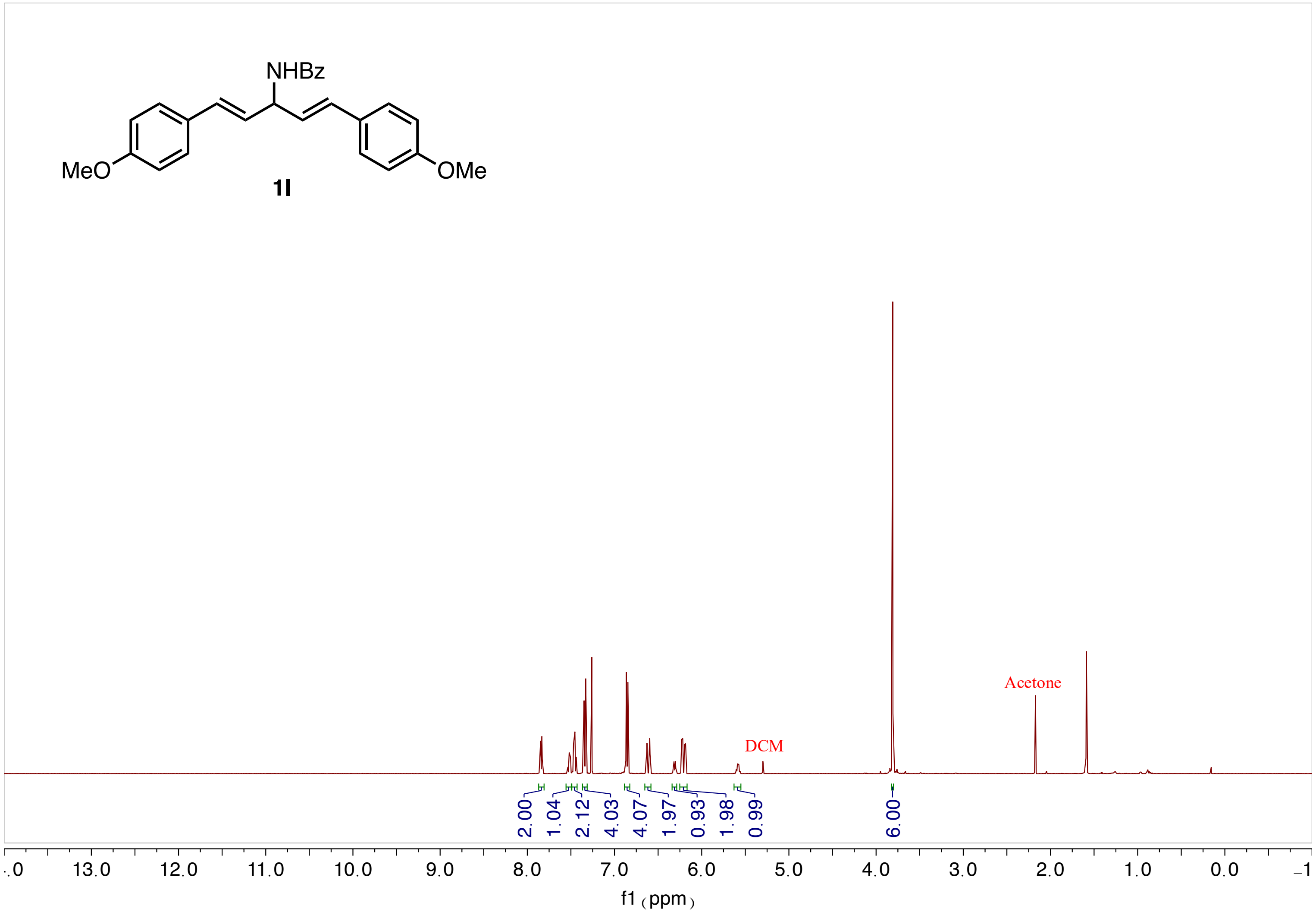

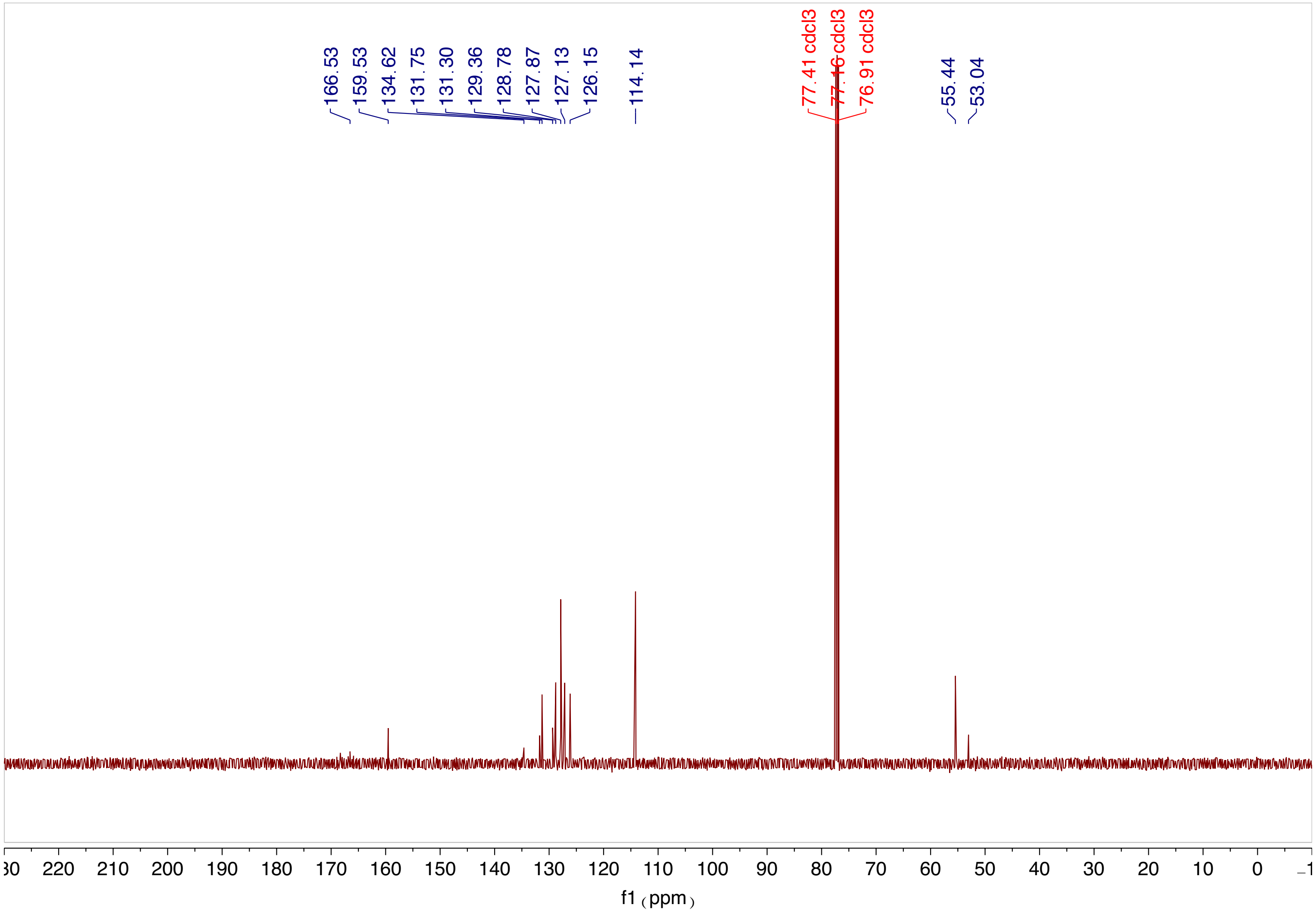

166.53
159.53
134.62
131.75
131.30
129.36
128.78
127.87
127.13
126.15
114.14
77.41 cdcl3
77.16 cdcl3
76.91 cdcl3
55.44
53.04
f1 (ppm)

Compound 1m: *N*-((1*E*,4*E*)-1,5-bis(4-(*tert*-butyl)phenyl)penta-1,4-dien-3-yl)benzamide- ^{1}H NMR (500 MHz, $CDCl_3$); $^{13}C\{^{1}H\}$ NMR (126 MHz, $CDCl_3$)

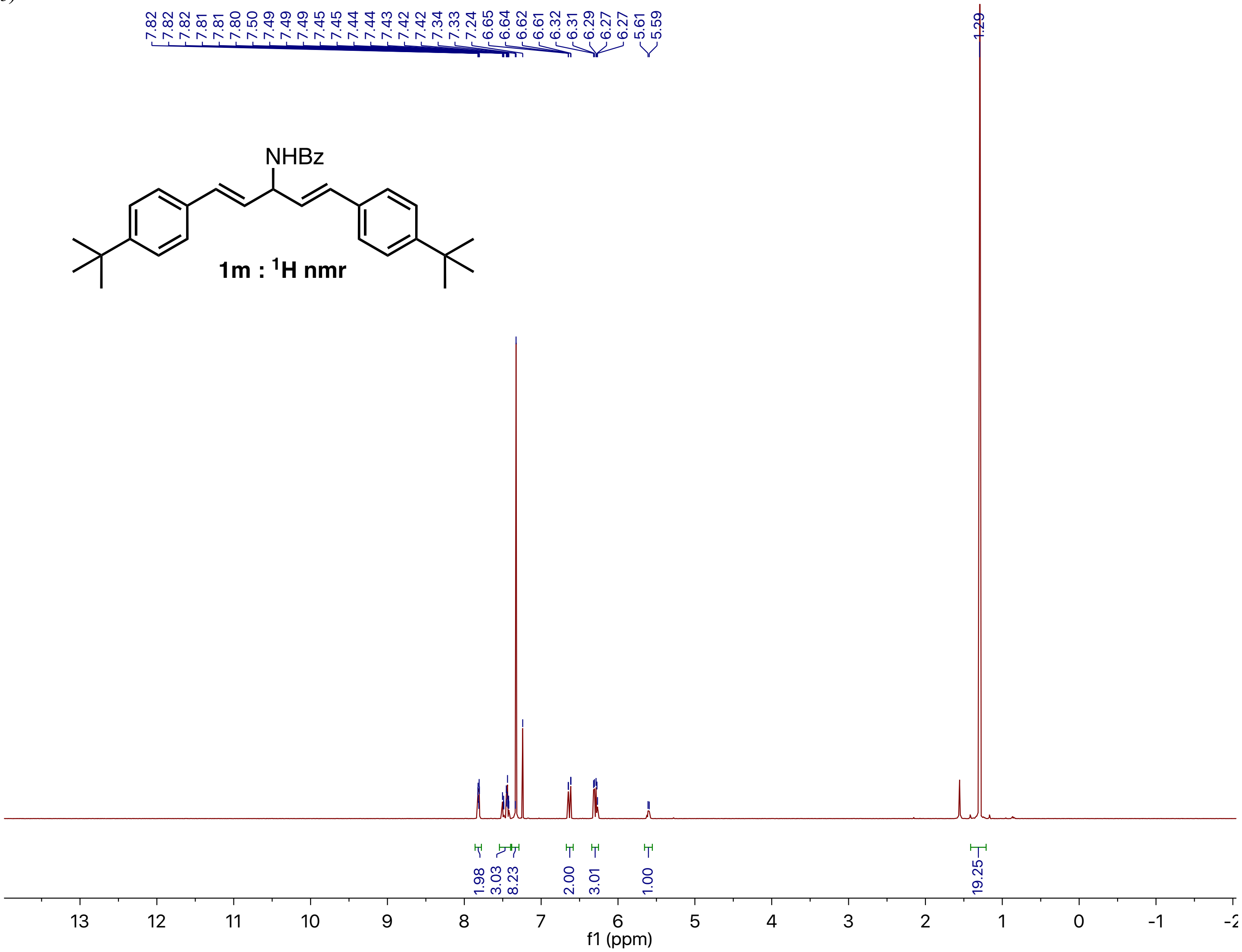

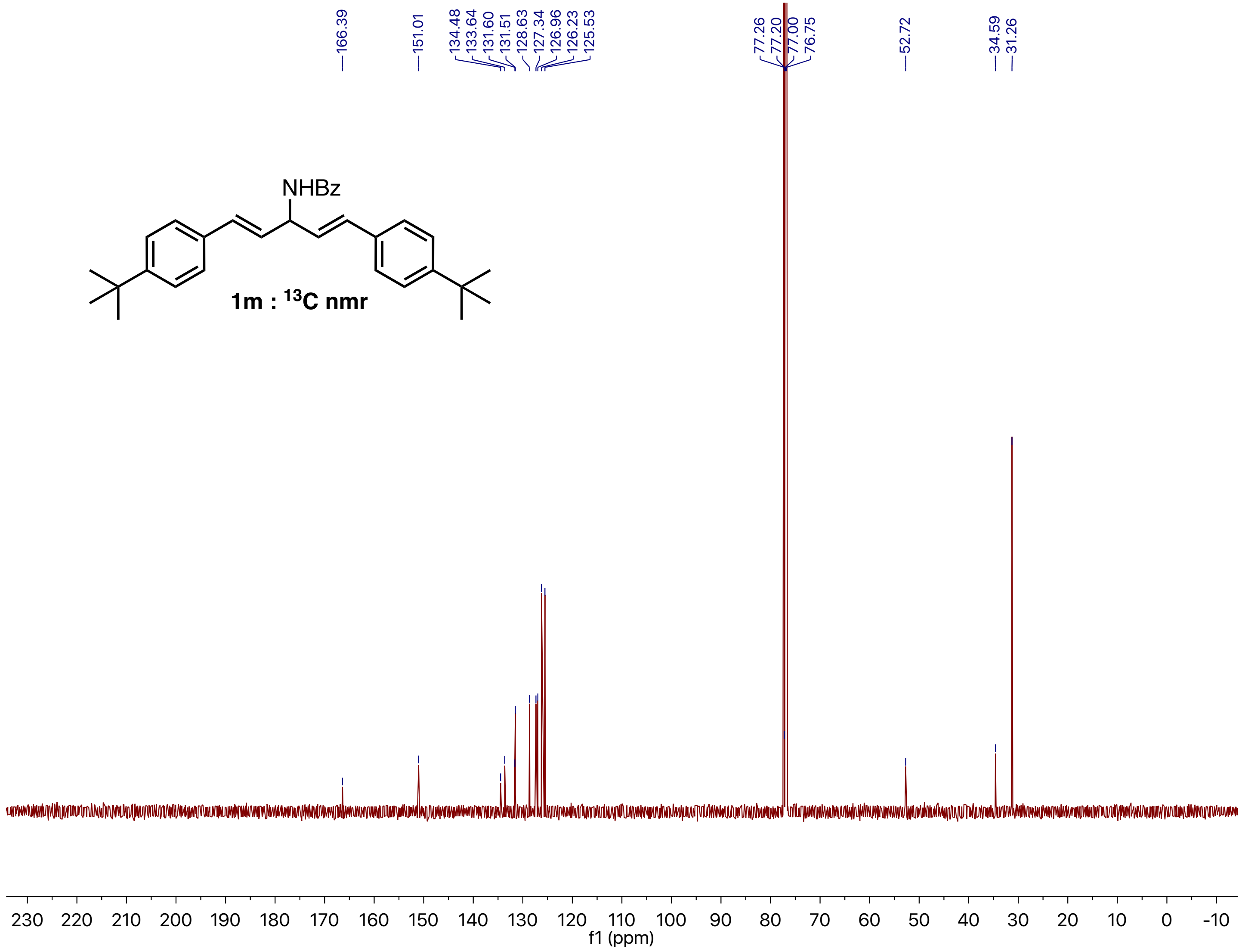
NHBz
1m : 13C nmr
166.39
151.01
134.48
133.64
131.60
131.51
128.63
127.34
126.96
126.23
125.53
77.26
77.20
77.00
76.75
52.72
34.59
31.26
230 220 210 200 190 180 170 160 150 140 130 120 110 100 90 80 70 60 50 40 30 20 10 0 -10
f1 (ppm)

Compound **1n**: *N*-((1*E*,4*E*)-1,5-di(thiophen-2-yl)penta-1,4-dien-3-yl)benzamide, ^{1}H NMR (500 MHz, $CDCl_3$); ^{13}C{^{1}H} NMR (126 MHz, $CDCl_3$)

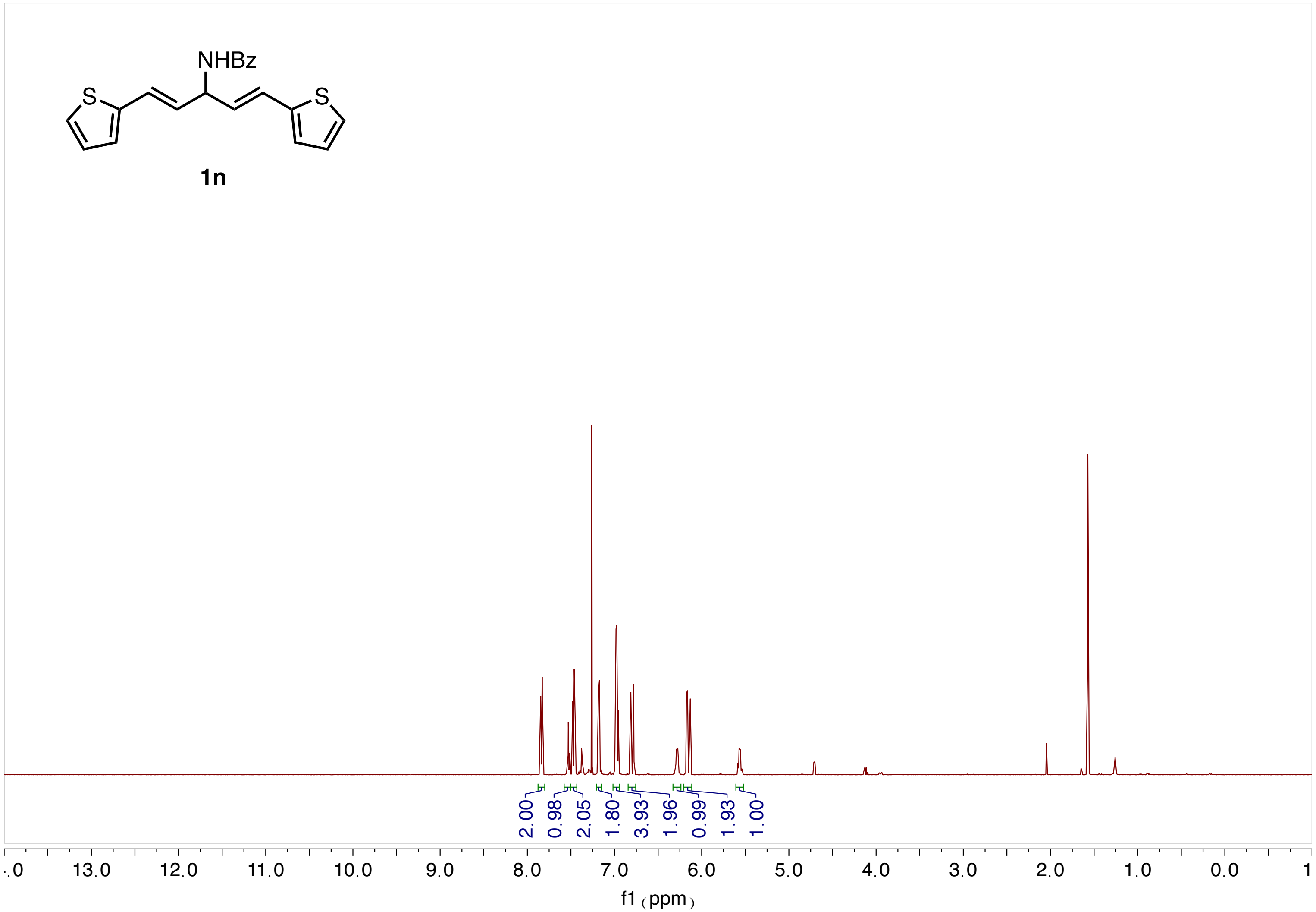

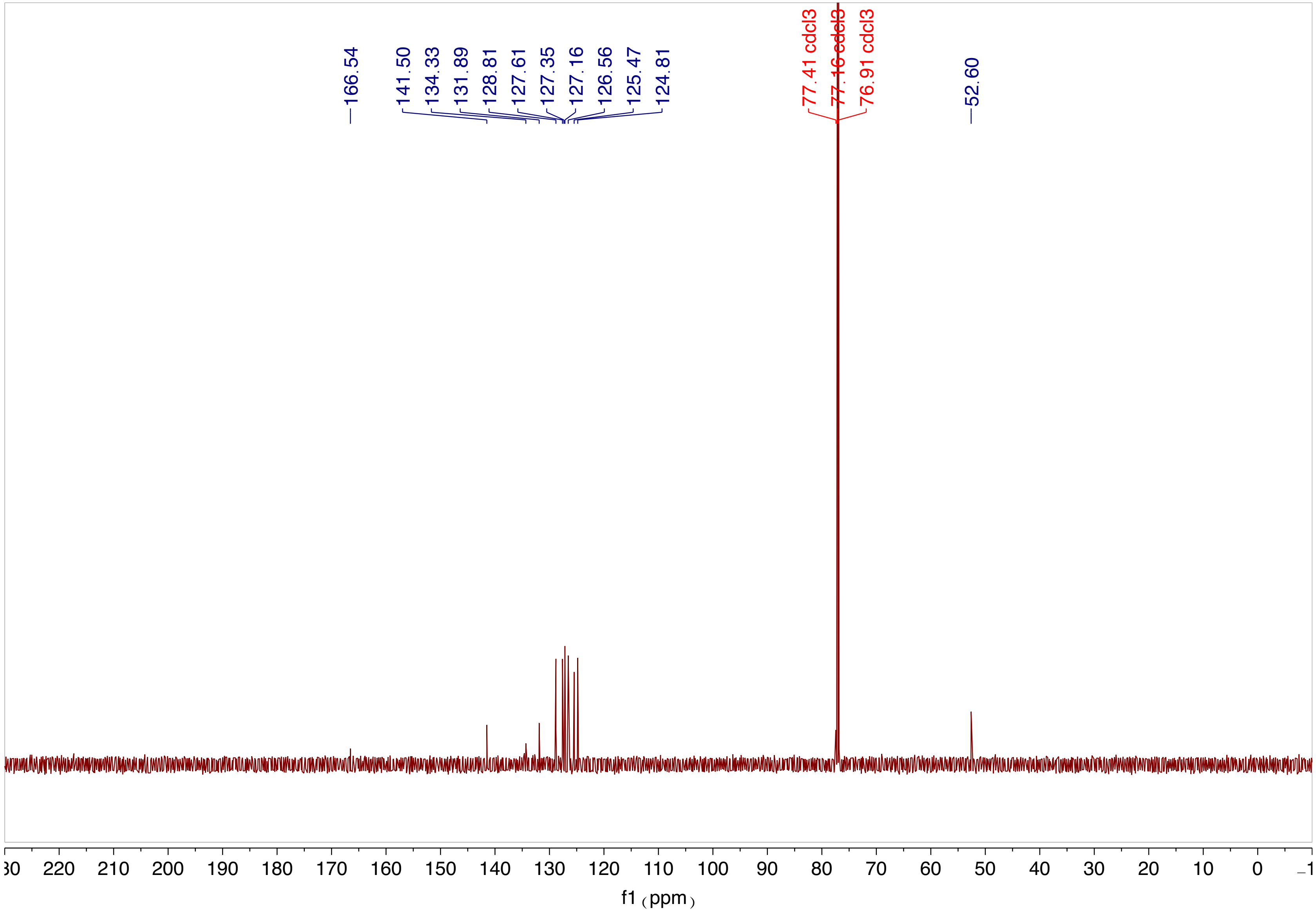

166.54
141.50
134.33
131.89
128.81
127.61
127.35
127.16
126.56
125.47
124.81
77.41 cdcl3
77.16 cdcl3
76.91 cdcl3
52.60
30
220
210
200
190
180
170
160
150
140
130
120
110
100
90
80
70
60
50
40
30
20
10
0
–1
f1 (ppm)

Compound 1o: *N*-((5*E*,8*E*)-trideca-5,8-dien-7-yl)benzamide- ^{1}H NMR (500 MHz, $CDCl_3$); ^{13}C{^{1}H} NMR (126 MHz, $CDCl_3$)

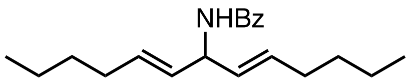

1o

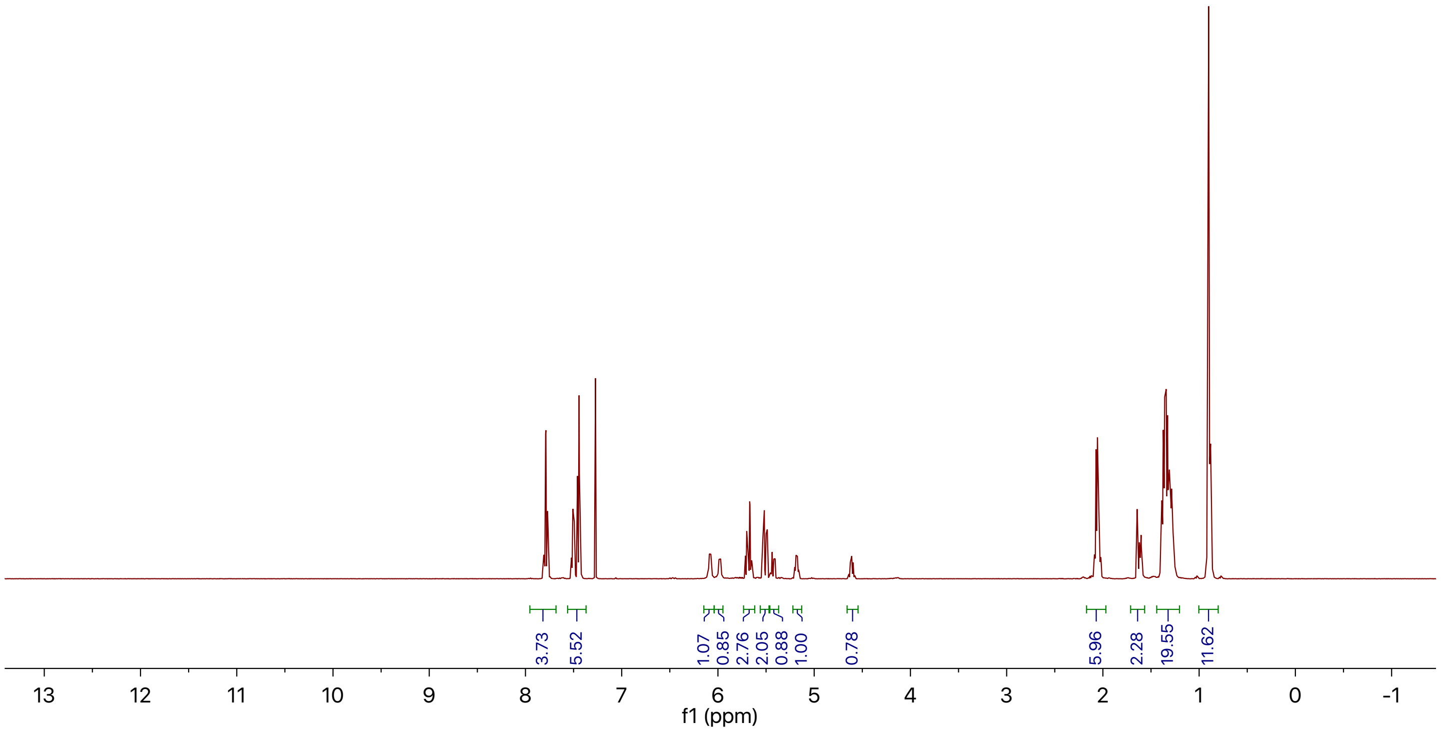

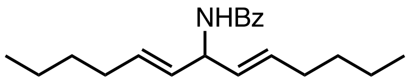
NHBz

1o

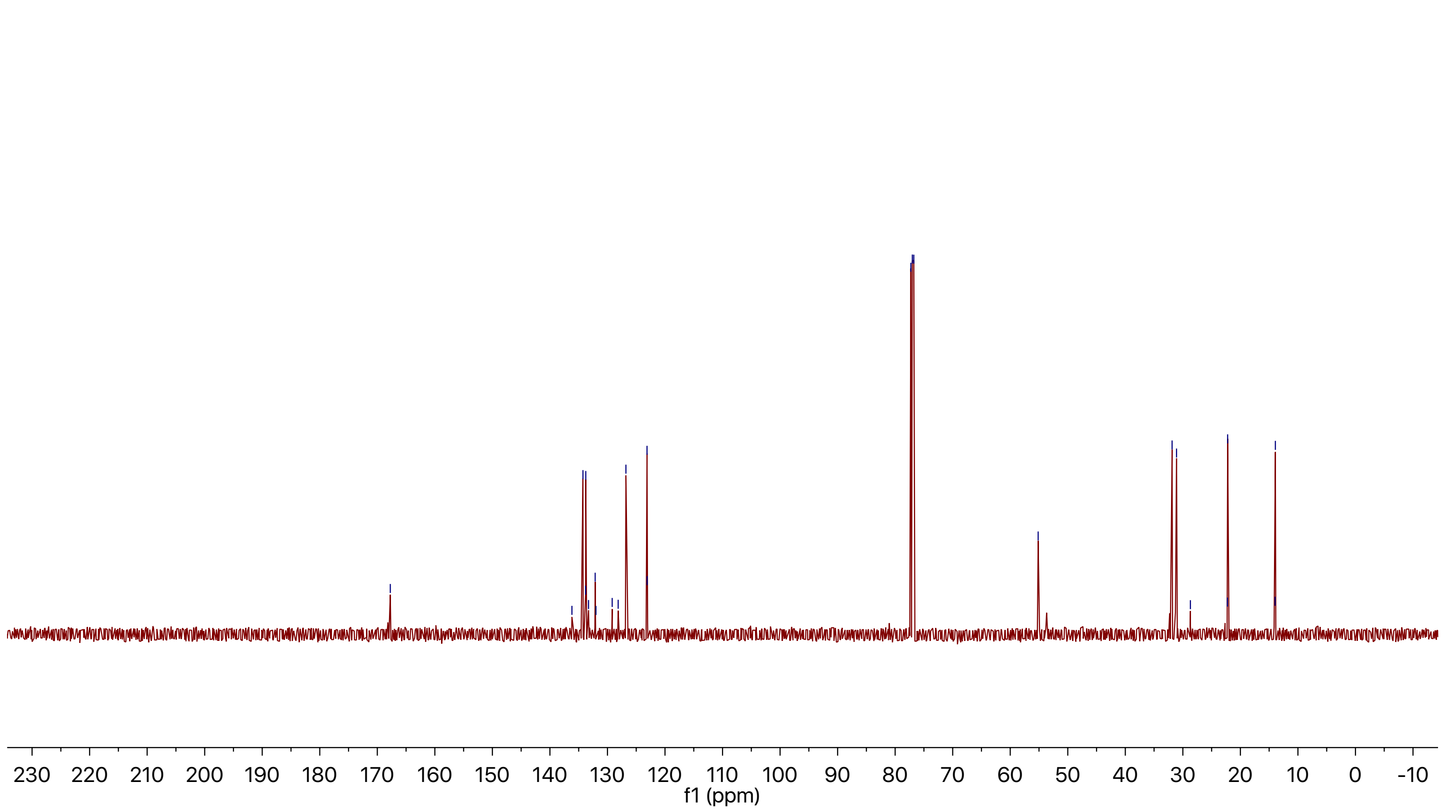
167.74
136.17
134.26
133.80
133.77
133.29
132.13
132.00
129.18
128.14
126.79
123.12
123.10
77.27
77.01
76.76
55.15
31.87
31.10
28.69
22.26
22.22
13.97
13.92
230 220 210 200 190 180 170 160 150 140 130 120 110 100 90 80 70 60 50 40 30 20 10 0 -10
f1 (ppm)

Compound 1p: N-((1*E*,4*E*)-1,5-dicyclohexylpenta-1,4-dien-3-yl)benzamide- ^{1}H NMR (500 MHz, $CDCl_3$); $^{13}C\{^{1}H\}$ NMR (126 MHz, $CDCl_3$)

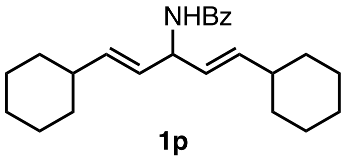

1p

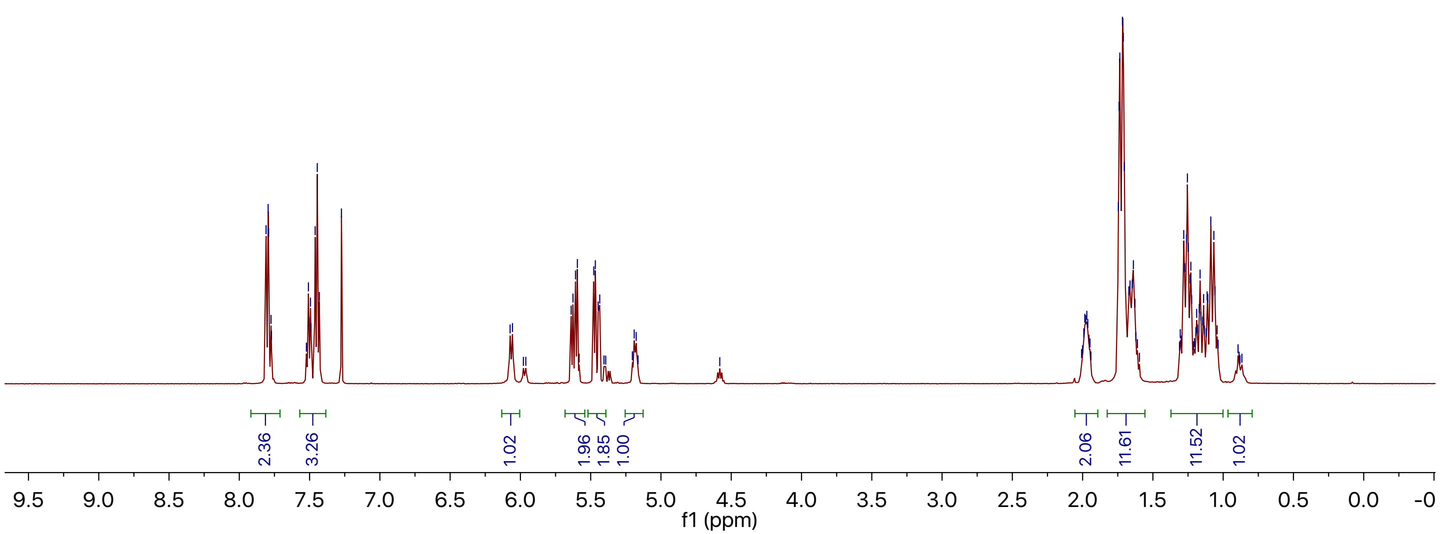

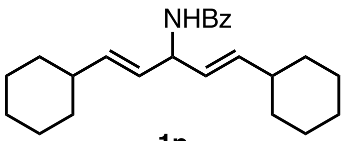
NHBz

1p

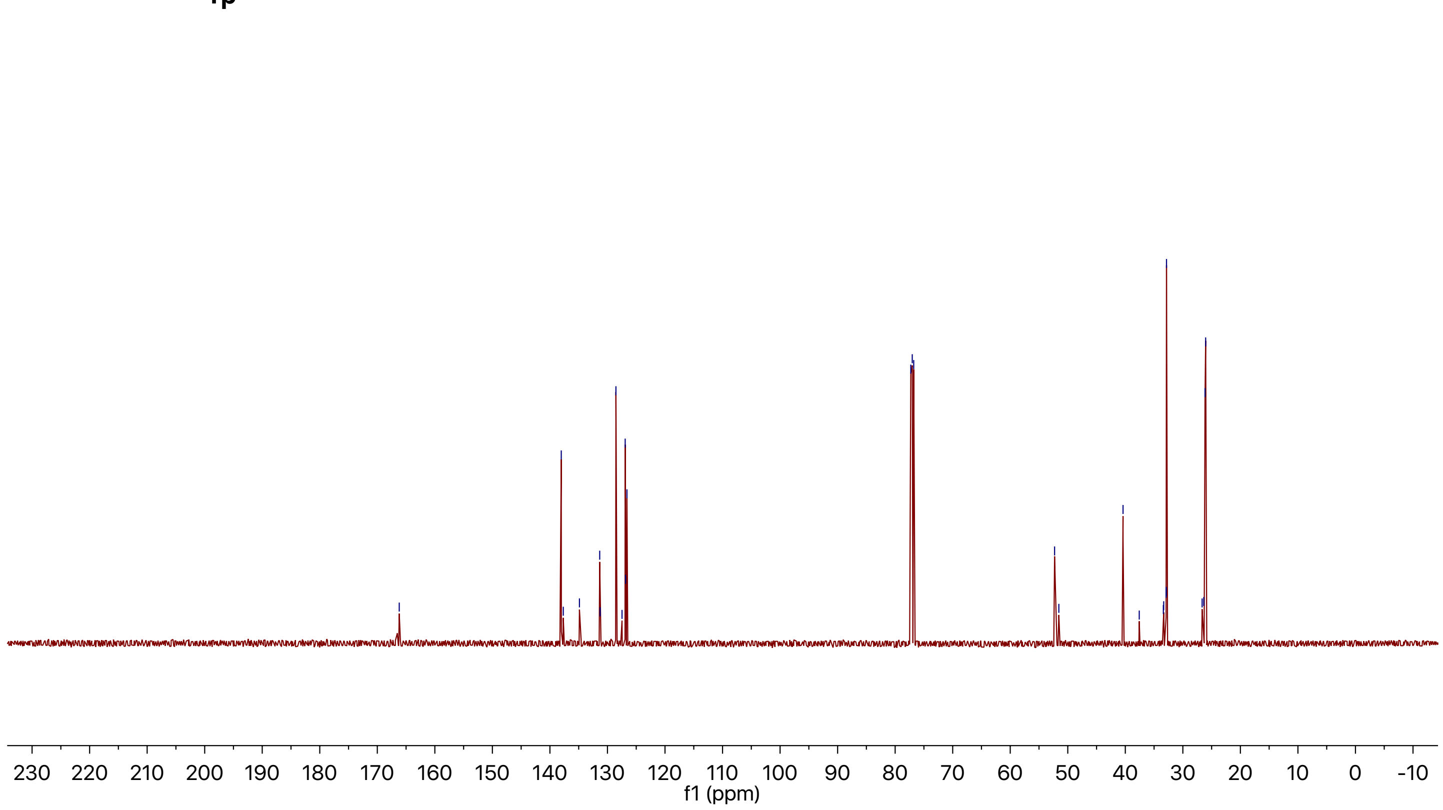
166.19
138.03
137.68
134.87
131.35
131.27
128.53
127.47
126.92
126.85
126.61
77.28
77.03
76.77
52.29
51.55
40.40
37.58
33.39
33.33
32.91
32.89
32.84
26.65
26.37
26.15
26.04
230 220 210 200 190 180 170 160 150 140 130 120 110 100 90 80 70 60 50 40 30 20 10 0 -10
f1 (ppm)

Compound 1q: *N*-((3*E*,6*E*)-2,2,8,8-tetramethylnona-3,6-dien-5-yl)benzamide- ^{1}H NMR (500 MHz, $CDCl_3$); $^{13}C\{^1H\}$ NMR (126 MHz, $CDCl_3$)

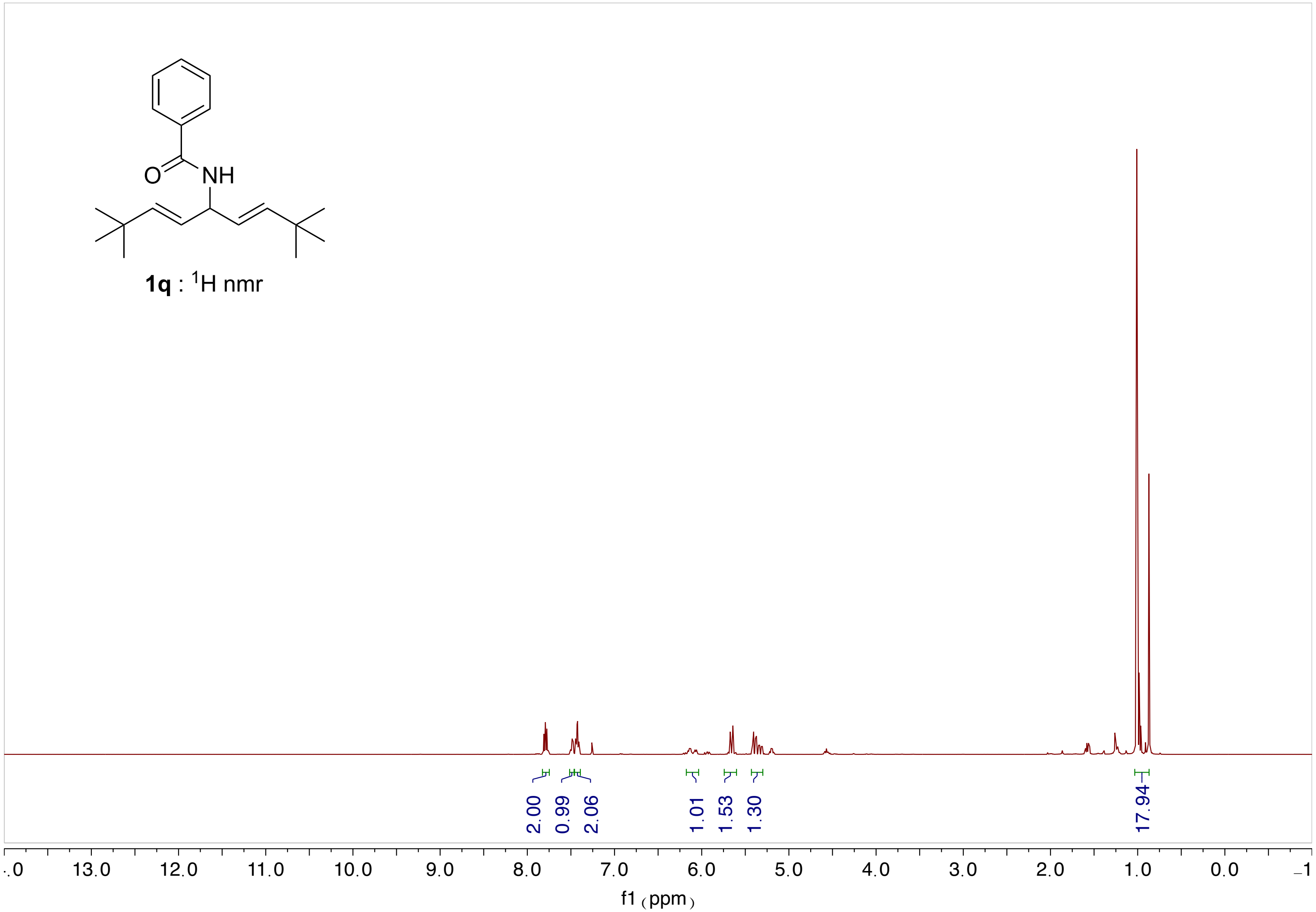

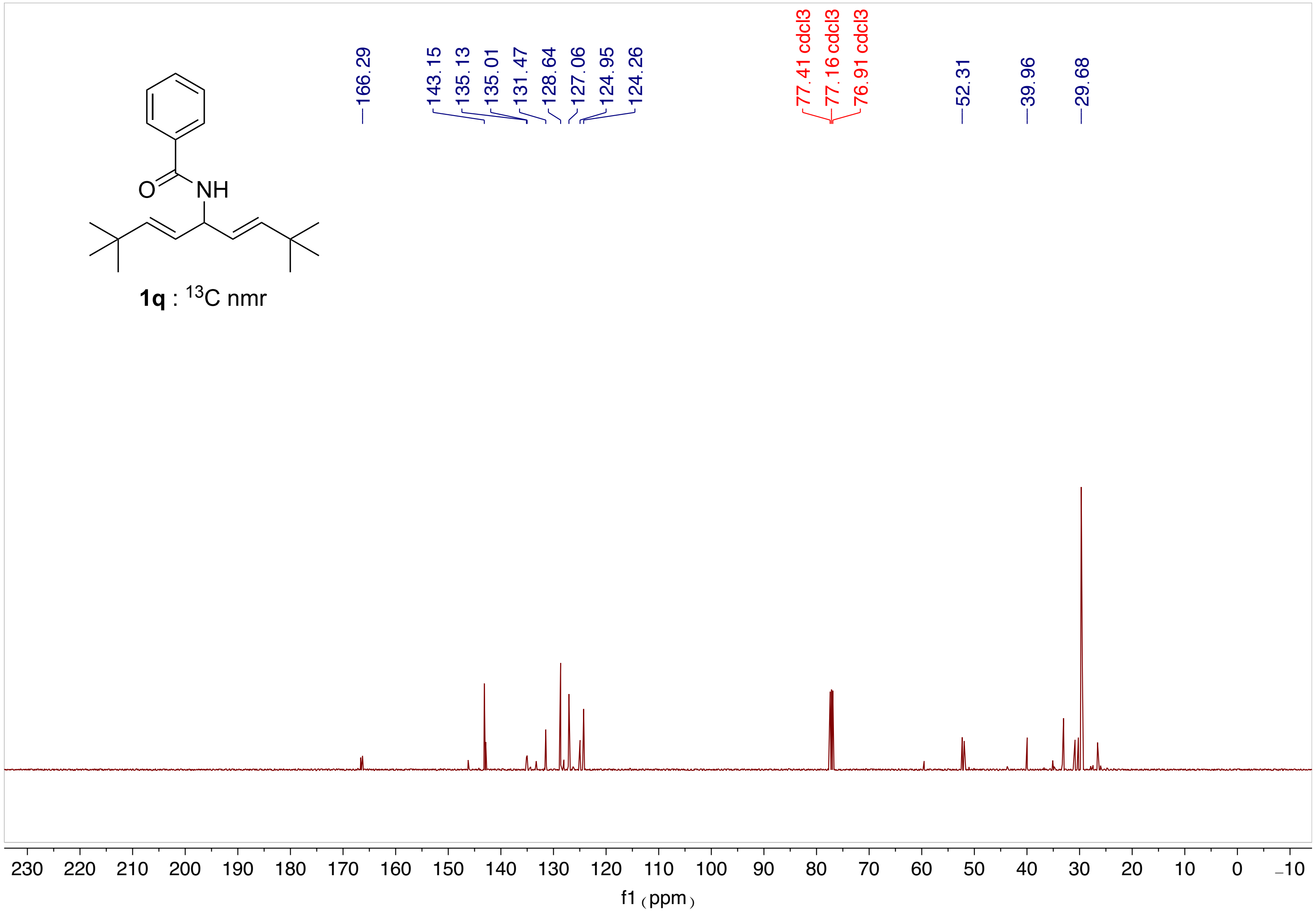

O
NH
1q : ^{13}C nmr
166.29
143.15
135.13
135.01
131.47
128.64
127.06
124.95
124.26
77.41 cdcl3
77.16 cdcl3
76.91 cdcl3
52.31
39.96
29.68
230 220 210 200 190 180 170 160 150 140 130 120 110 100 90 80 70 60 50 40 30 20 10 0 -10
f1 (ppm)

Compound 1r: *N*-((1*E*,4*E*)-1,5-diphenylpenta-1,4-dien-3-yl)isobutyramide- ^{1}H NMR (500 MHz, $CDCl_3$); ^{13}C{^{1}H} NMR (126 MHz, $CDCl_3$)

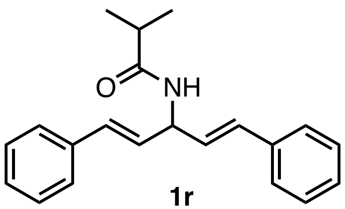

1r

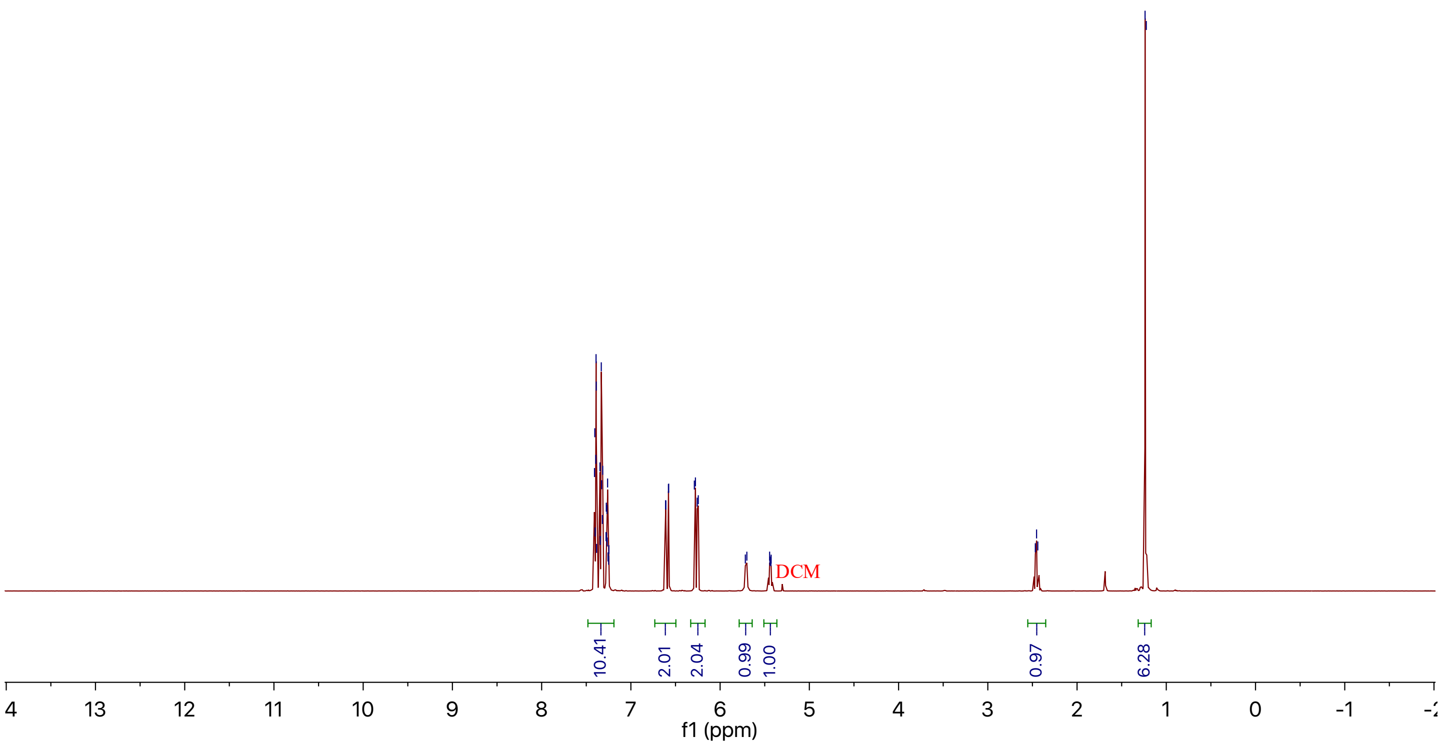

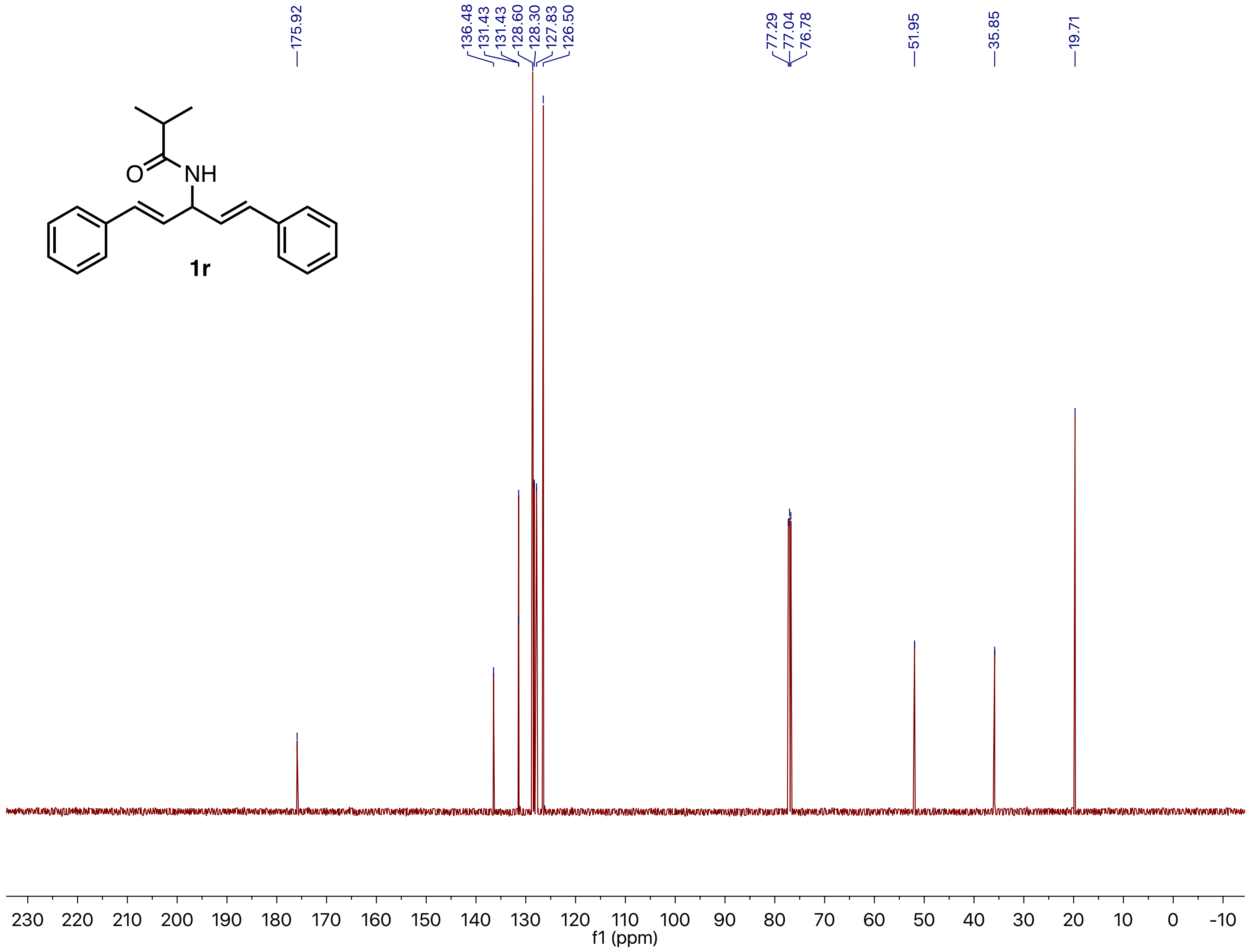
175.92
136.48
131.43
131.43
128.60
128.30
127.83
126.50
77.29
77.04
76.78
51.95
35.85
19.71
O
NH
1r
230 220 210 200 190 180 170 160 150 140 130 120 110 100 90 80 70 60 50 40 30 20 10 0 -10
f1 (ppm)

Compound *1s*: *N*-((1*E*,4*E*)-1,5-diphenylpenta-1,4-dien-3-yl)-4-methoxybenzamide **1s**- ^{1}H NMR (500 MHz, CDCl$_3$); ^{13}C{^{1}H} NMR (126 MHz, CDCl$_3$)

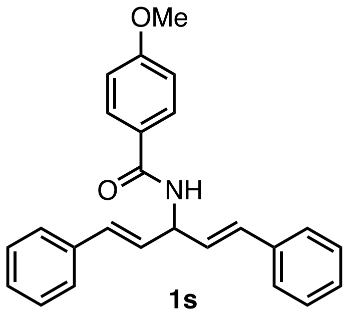

1s

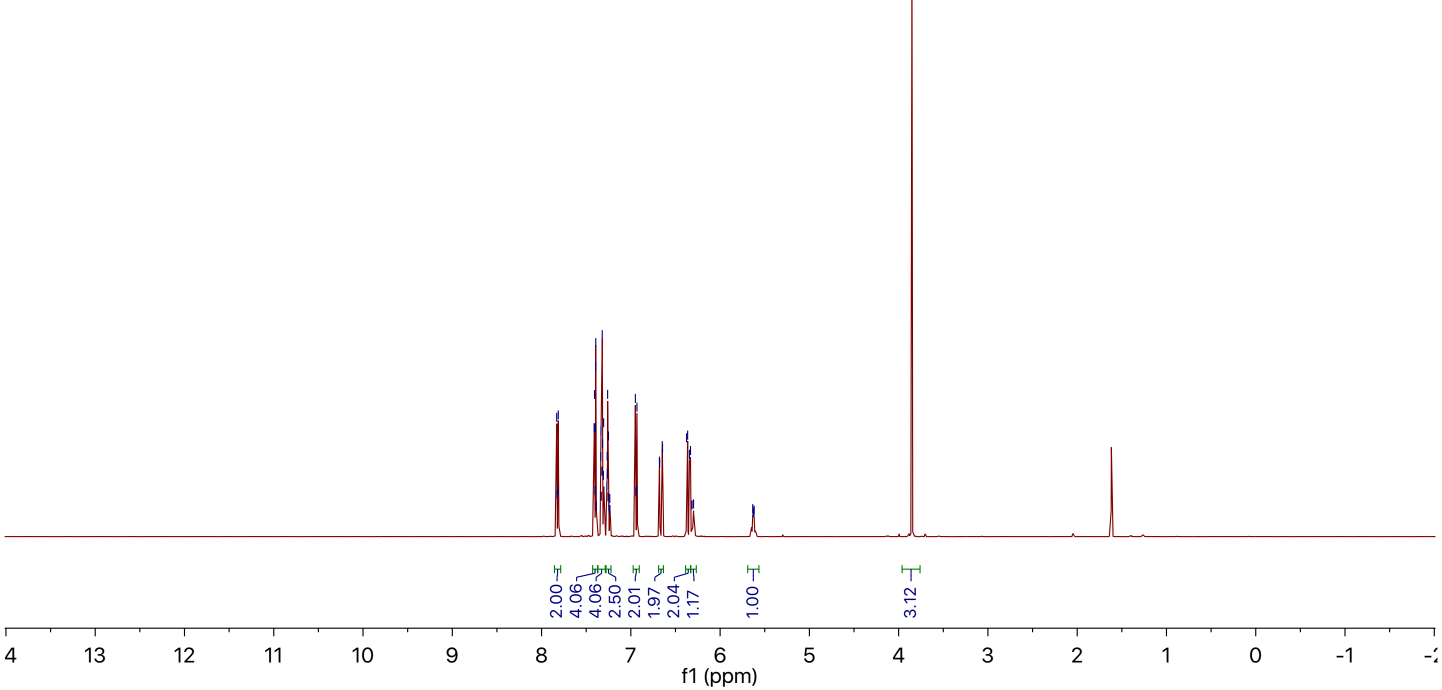

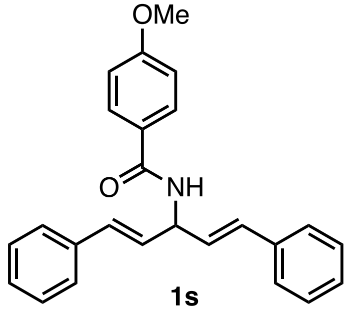
OMe
O
NH

1s

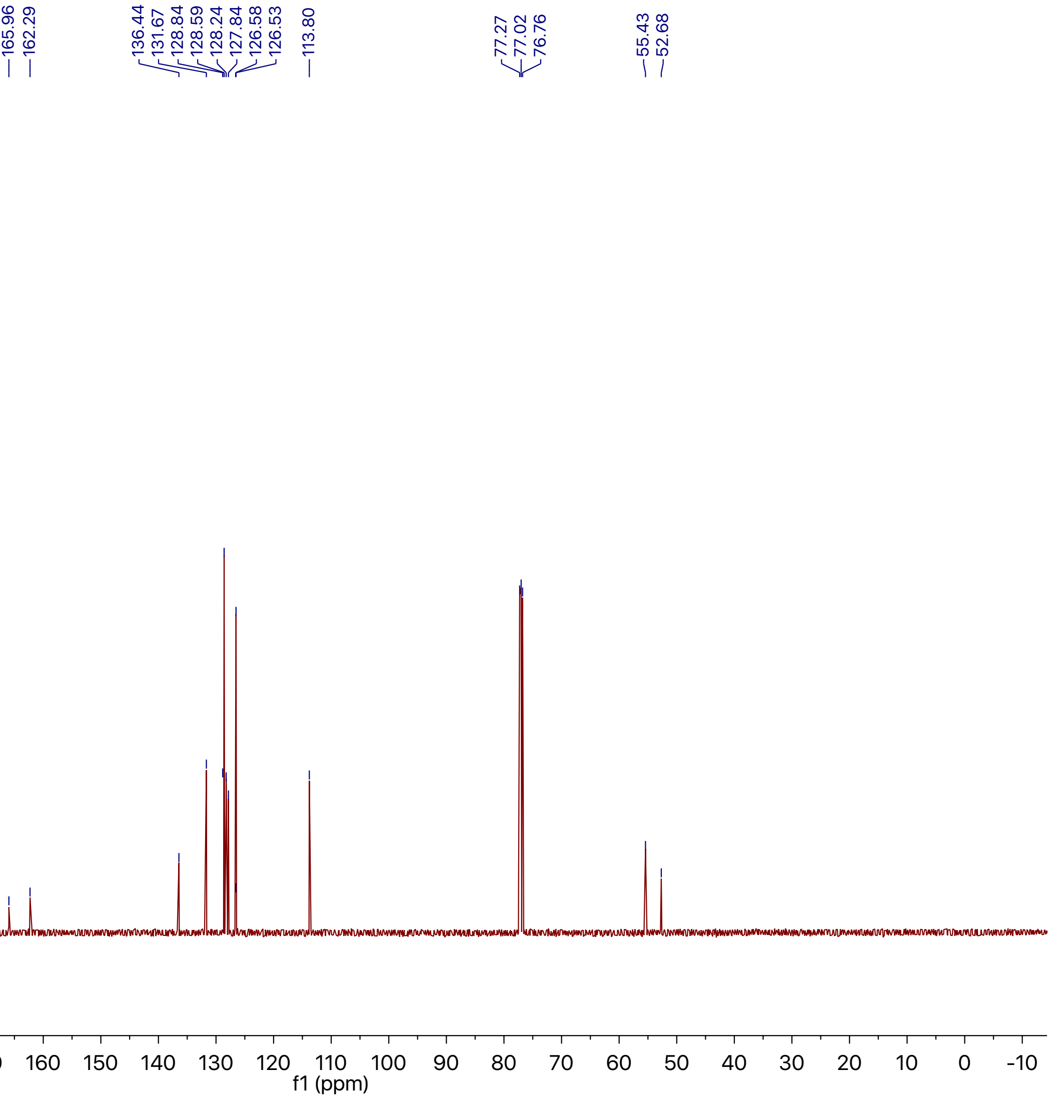
165.96
162.29
136.44
131.67
128.84
128.59
128.24
127.84
126.58
126.53
113.80
77.27
77.02
76.76
55.43
52.68
230 220 210 200 190 180 170 160 150 140 130 120 110 100 90 80 70 60 50 40 30 20 10 0 -10
f1 (ppm)

Compound 1t: 4-bromo-*N*-((1*E*,4*E*)-1,5-diphenylpenta-1,4-dien-3-yl)benzamide- ^{1}H NMR (500 MHz, $CDCl_3$); ^{13}C{^{1}H} NMR (126 MHz, $CDCl_3$)

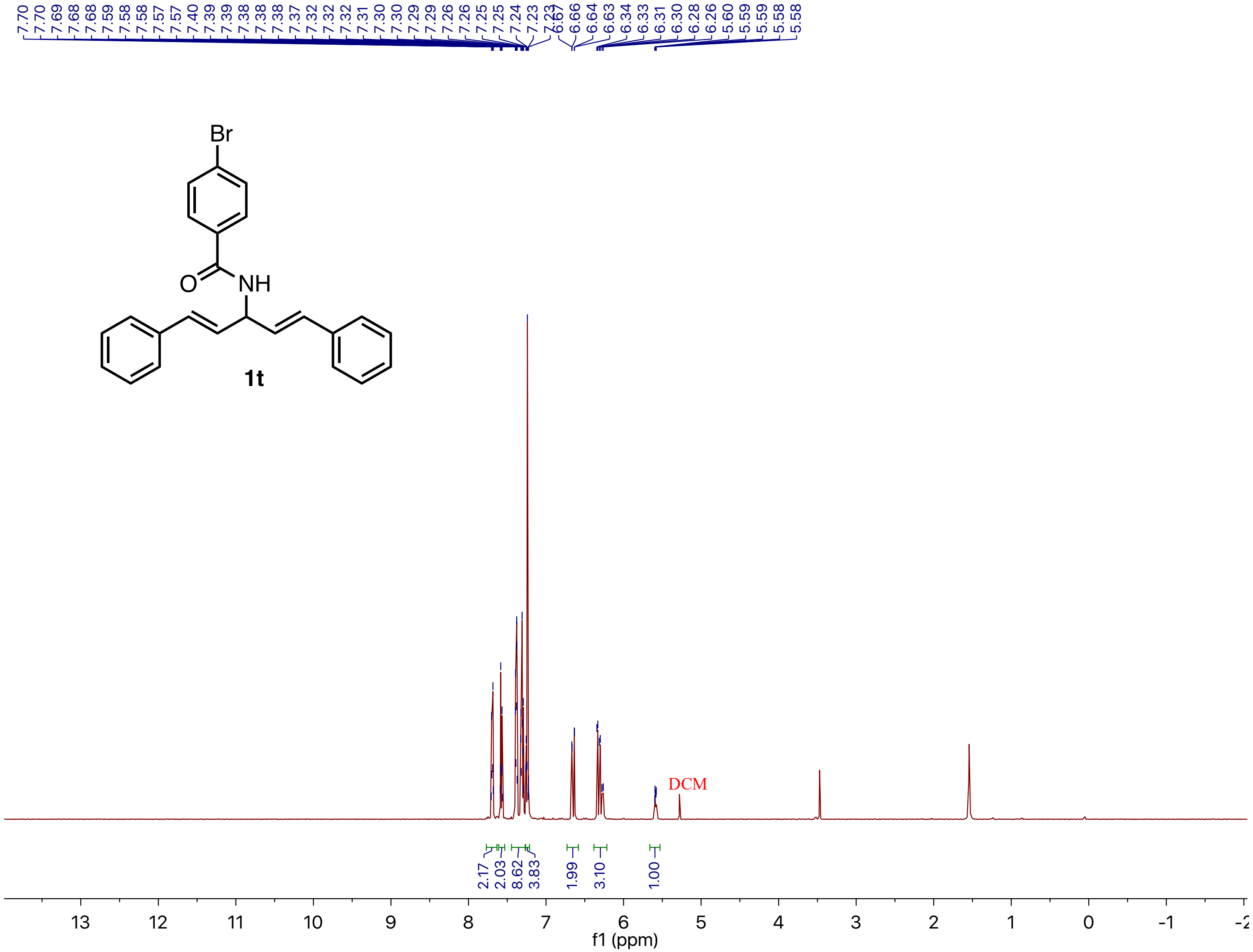

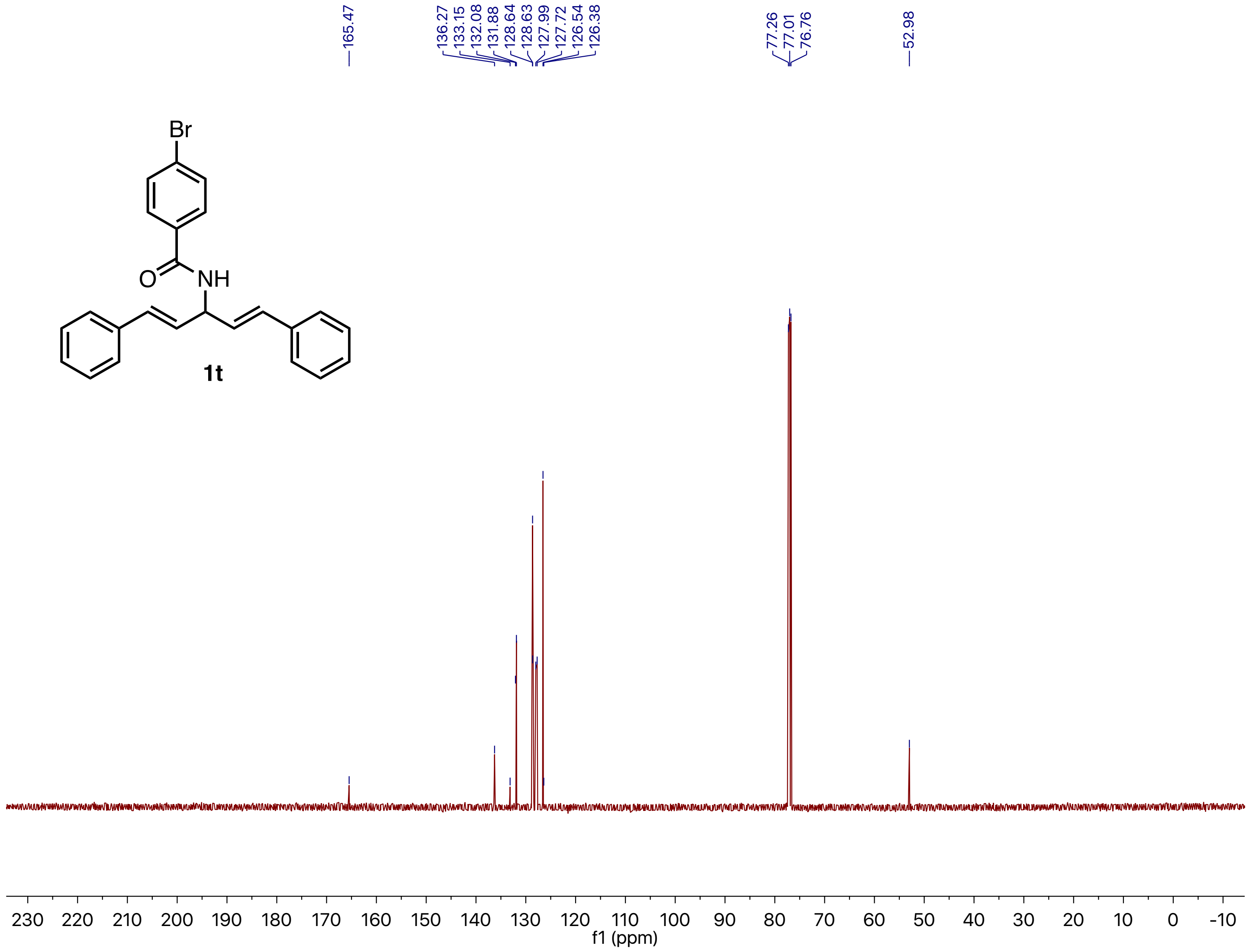
165.47
136.27
133.15
132.08
131.88
128.64
128.63
127.99
127.72
126.54
126.38
77.26
77.01
76.76
52.98
Br
O
NH
1t
230 220 210 200 190 180 170 160 150 140 130 120 110 100 90 80 70 60 50 40 30 20 10 0 -10
f1 (ppm)

Compound 2a: (4*R*,5*S*,6*R*)-5-chloro-2,6-diphenyl-4-((*E*)-styryl)-5,6-dihydro-4*H*-1,3-oxazine- ^{1}H NMR (500 MHz, CDCl$_3$); ^{13}C{^{1}H} NMR (126 MHz, CDCl$_3$)

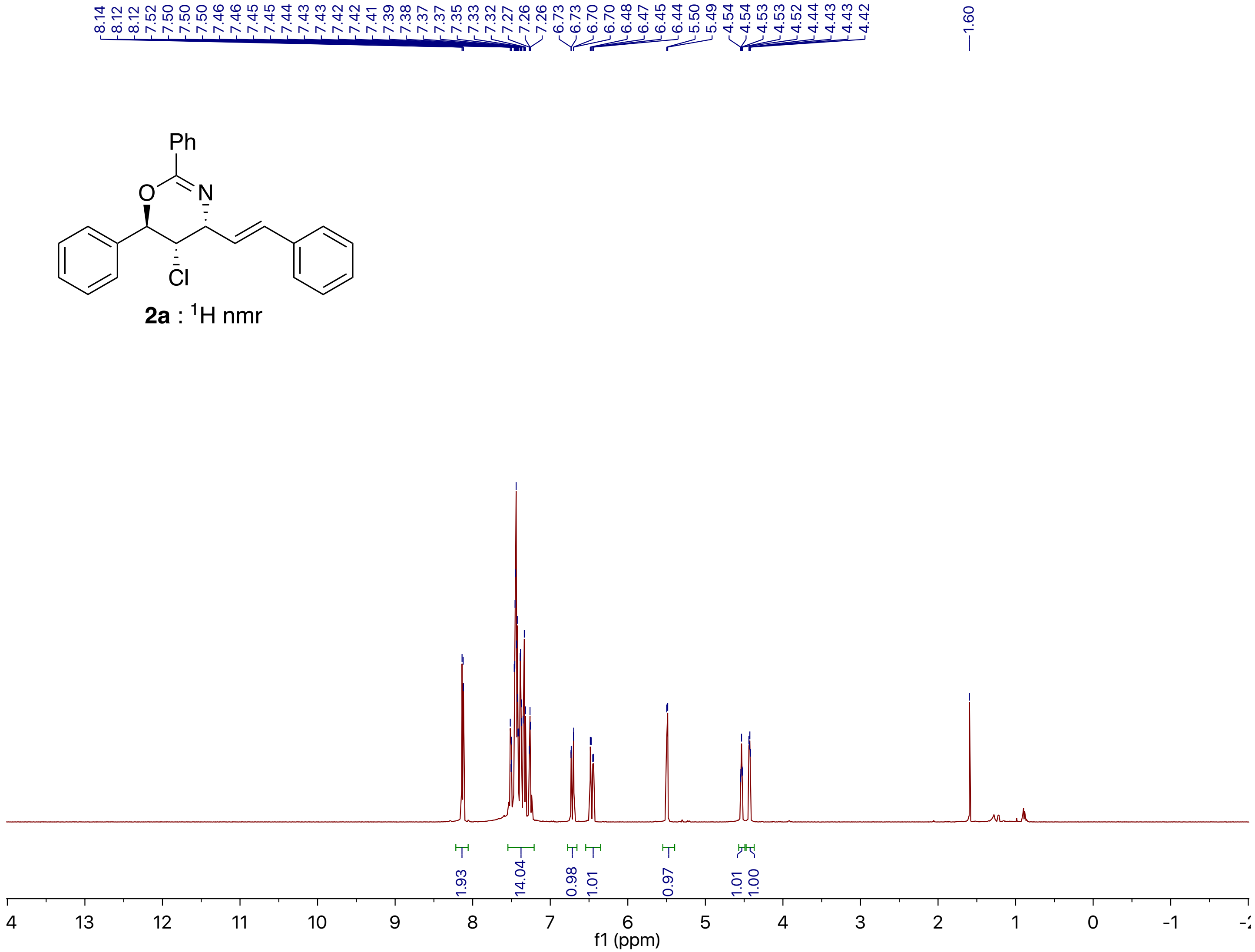

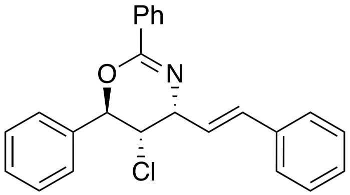
Ph
O
N
Cl

2a : ^{13}C nmr

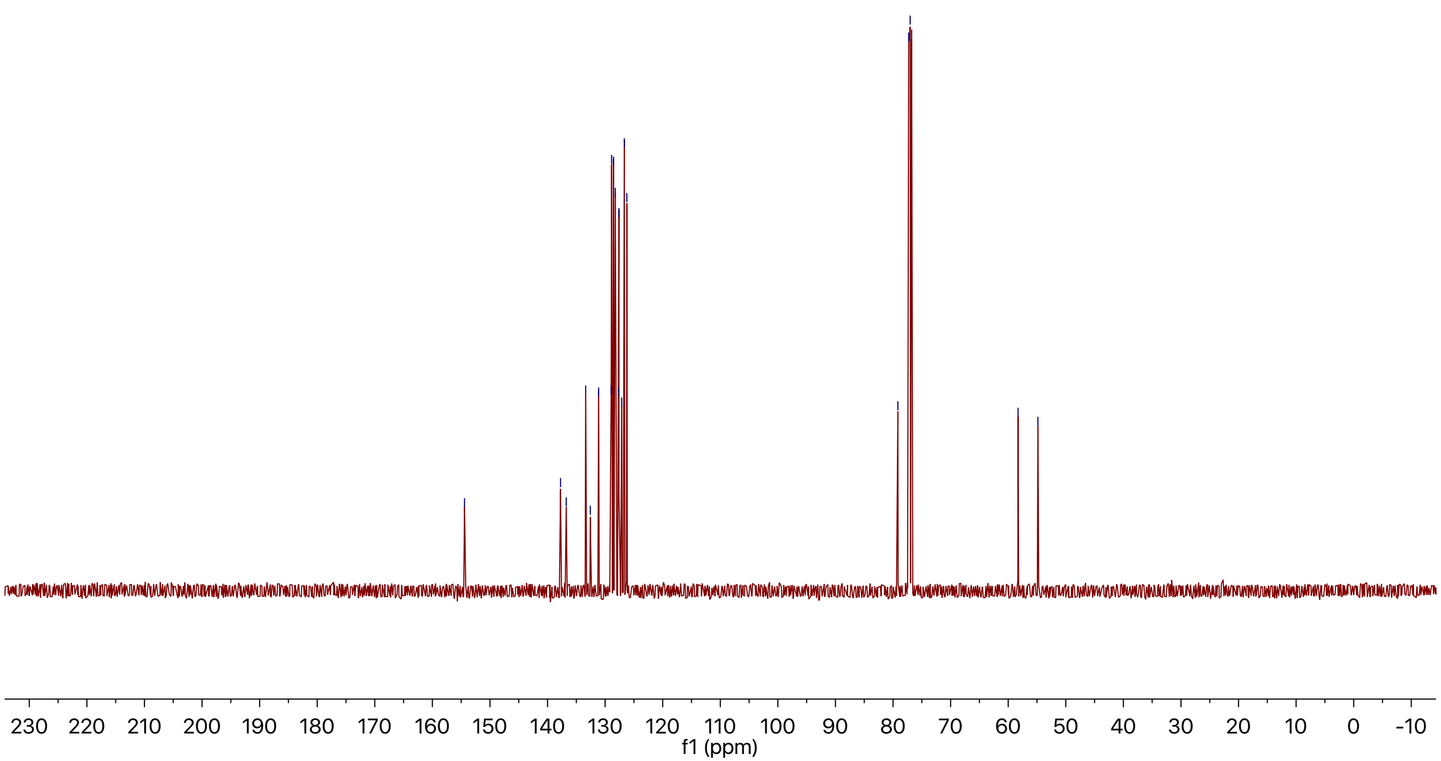
154.42
137.74
136.75
133.38
132.58
131.12
128.95
128.88
128.53
128.24
127.69
127.60
127.11
126.67
126.23
79.16
77.28
77.03
76.77
58.29
54.84
230 220 210 200 190 180 170 160 150 140 130 120 110 100 90 80 70 60 50 40 30 20 10 0 -10
f1 (ppm)

Compound 2b: (4*R*,5*S*,6*R*)-5-chloro-6-(4-chlorophenyl)-4-((*E*)-4-chlorostyryl)-2-phenyl-5,6-dihydro-4*H*-1,3-oxazine- ^{1}H NMR (500 MHz, $CDCl_3$); $^{13}C\{^{1}H\}$ NMR (126 MHz, $CDCl_3$)

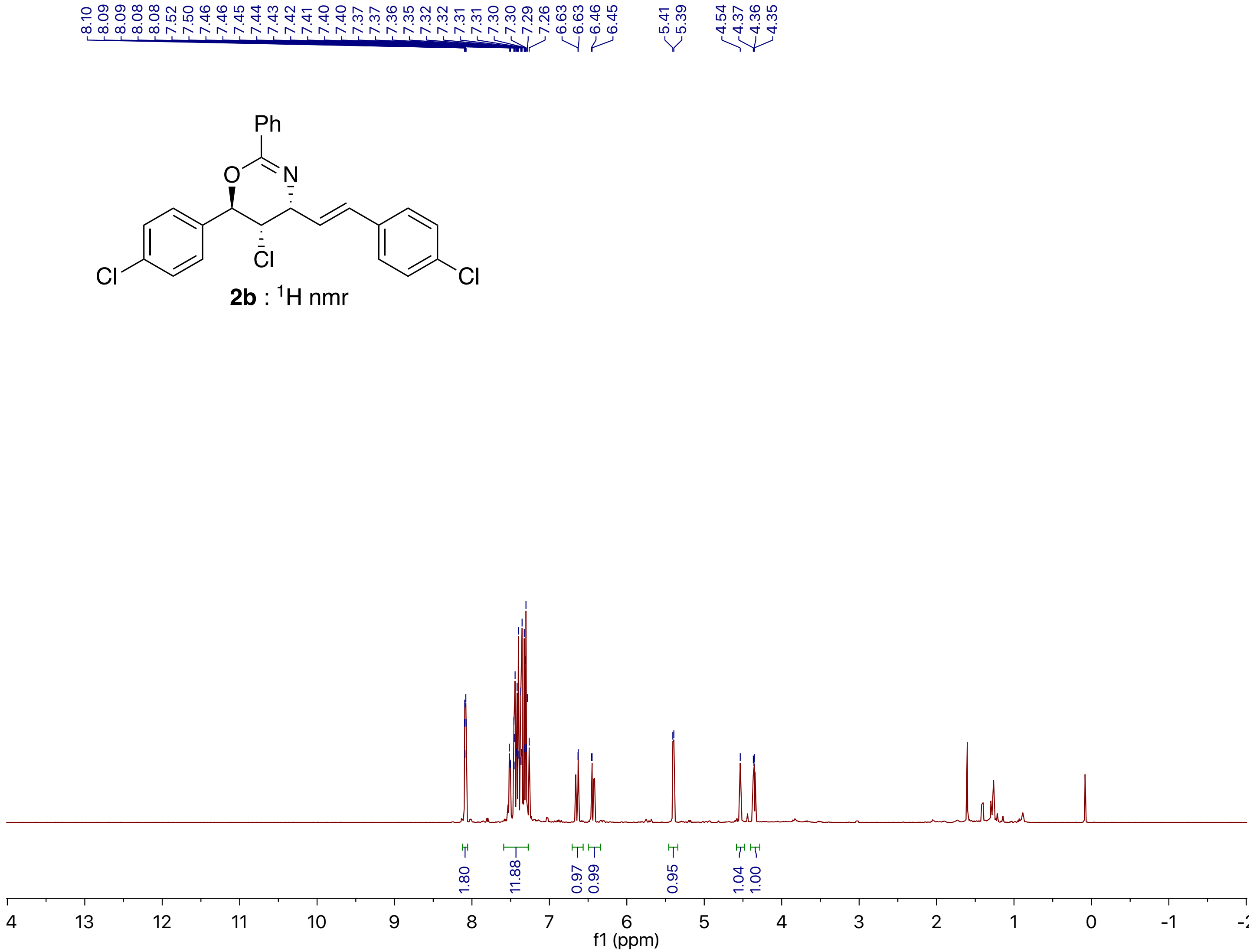

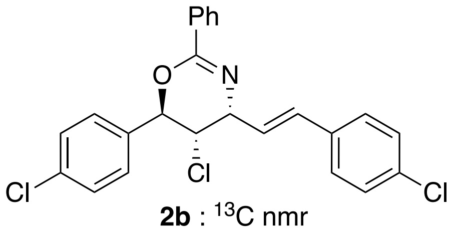
Ph
O
N
Cl
Cl
Cl

2b : ^{13}C nmr

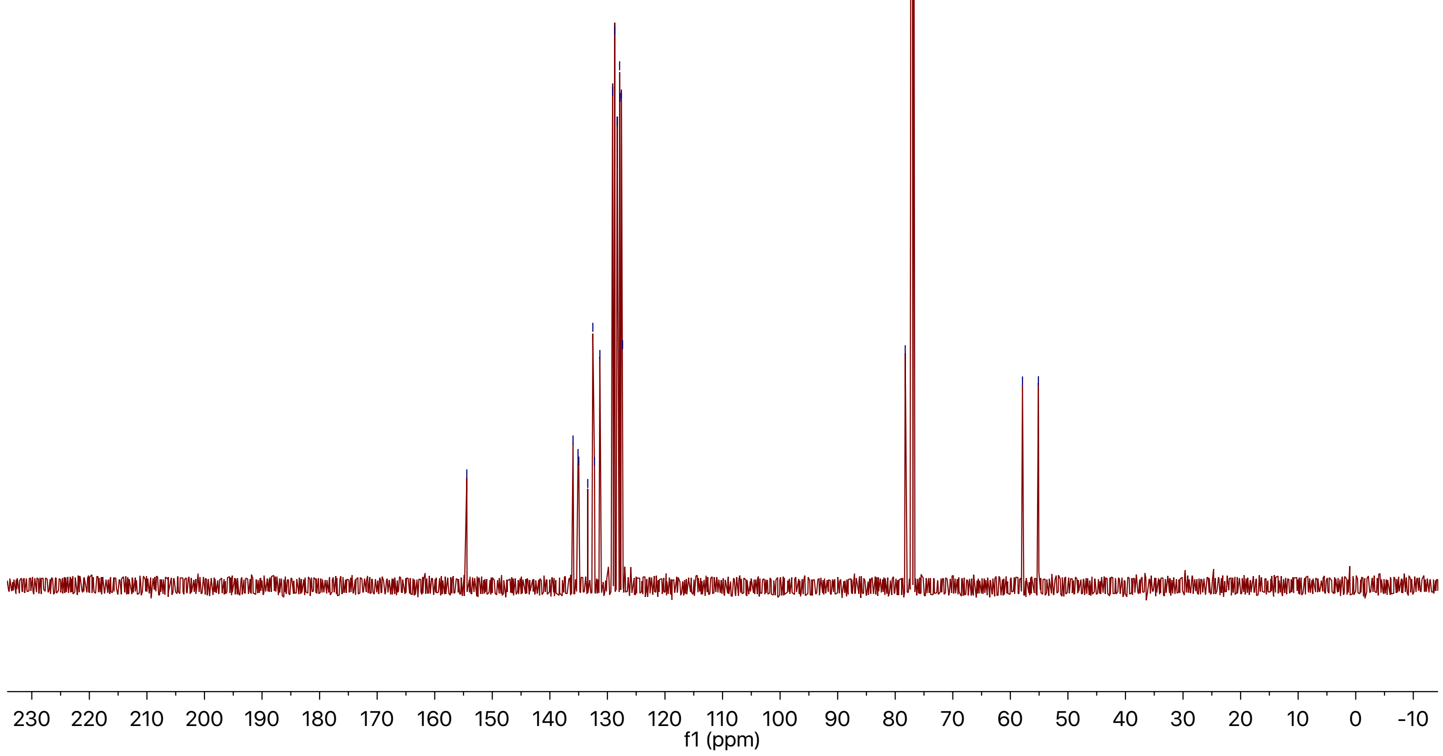
154.43
135.97
135.10
134.97
133.42
132.53
132.24
131.31
129.10
128.71
128.30
127.88
127.81
127.56
127.36
78.26
77.28
77.02
76.77
57.89
55.13
230 220 210 200 190 180 170 160 150 140 130 120 110 100 90 80 70 60 50 40 30 20 10 0 -10
f1 (ppm)

Compound 2c: (4*R*,5*S*,6*R*)-6-(4-bromophenyl)-4-((*E*)-4-bromostyryl)-5-chloro-2-phenyl-5,6-dihydro-4*H*-1,3-oxazine- ^{1}H NMR (500 MHz, $CDCl_3$); $^{13}C\{^{1}H\}$ NMR (126 MHz, $CDCl_3$)

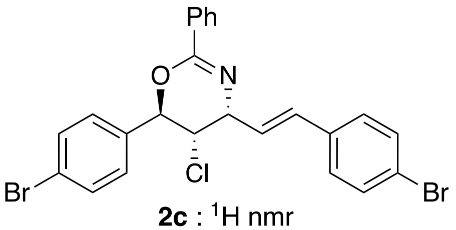

2c : ^{1}H nmr

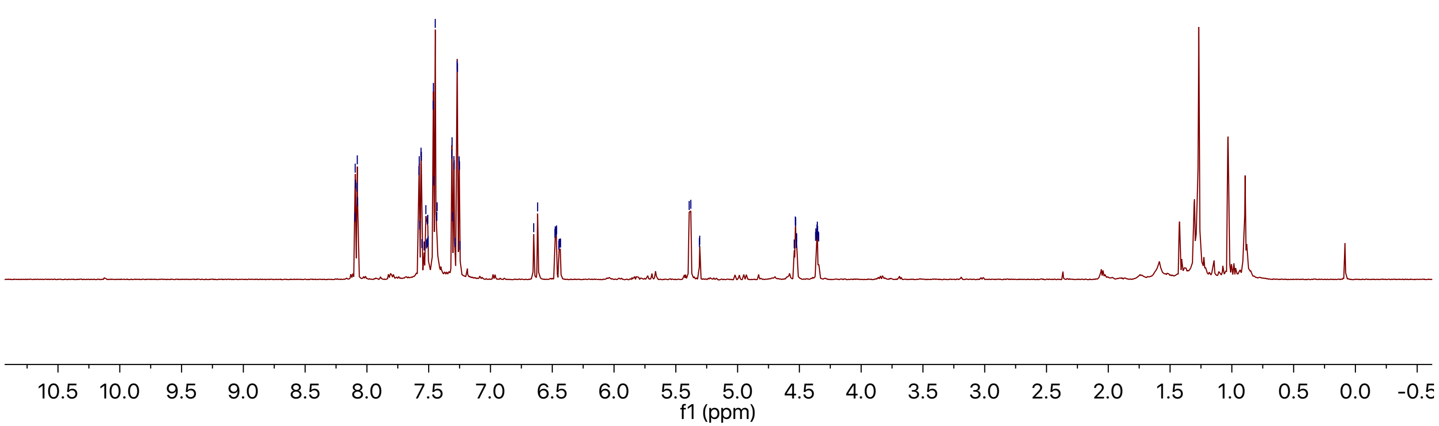

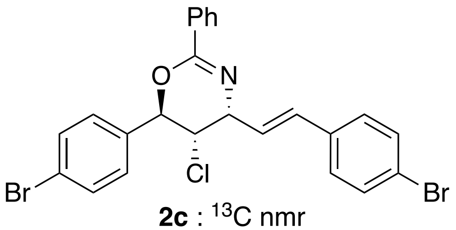
Ph
O
N
Br
Cl
Br

2c : ^{13}C nmr

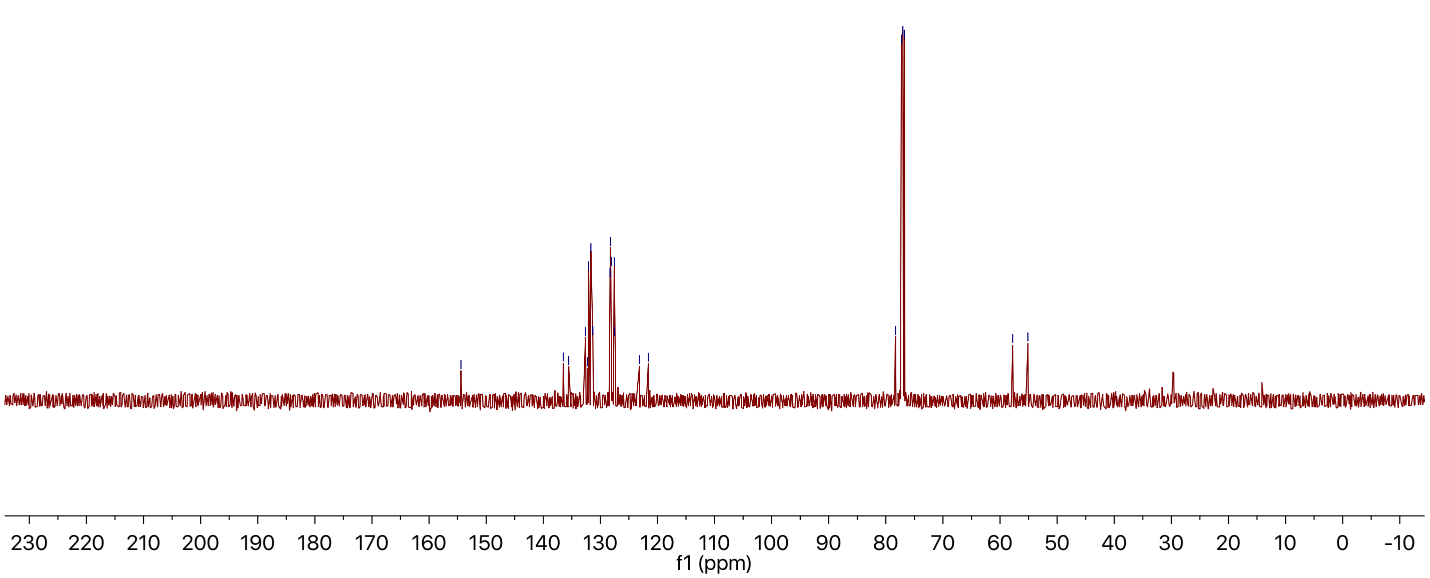
154.41
136.50
135.55
132.60
132.22
132.05
131.66
131.32
128.31
128.21
128.10
127.56
127.49
123.14
121.60
78.32
77.27
77.02
76.77
57.78
55.12
230 220 210 200 190 180 170 160 150 140 130 120 110 100 90 80 70 60 50 40 30 20 10 0 -10
f1 (ppm)

Compound 2d: (4*R*,5*S*,6*R*)-6-(3-bromophenyl)-4-((*E*)-3-bromostyryl)-5-chloro-2-phenyl-5,6-dihydro-4*H*-1,3-oxazine- ^{1}H NMR (500 MHz, CDCl$_3$); ^{13}C{^{1}H} NMR (126 MHz, CDCl$_3$)

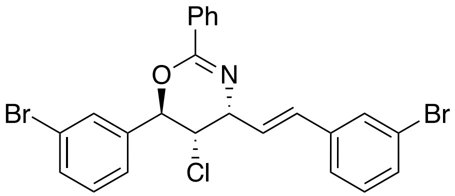

2d : ^{1}H nmr

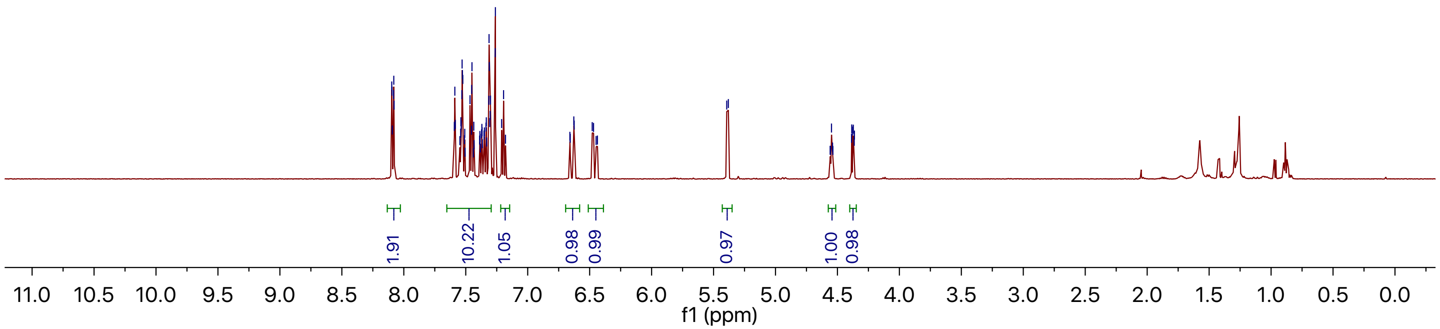

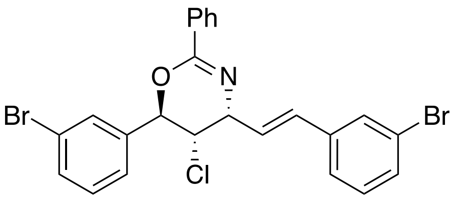

2d : ^{13}C nmr

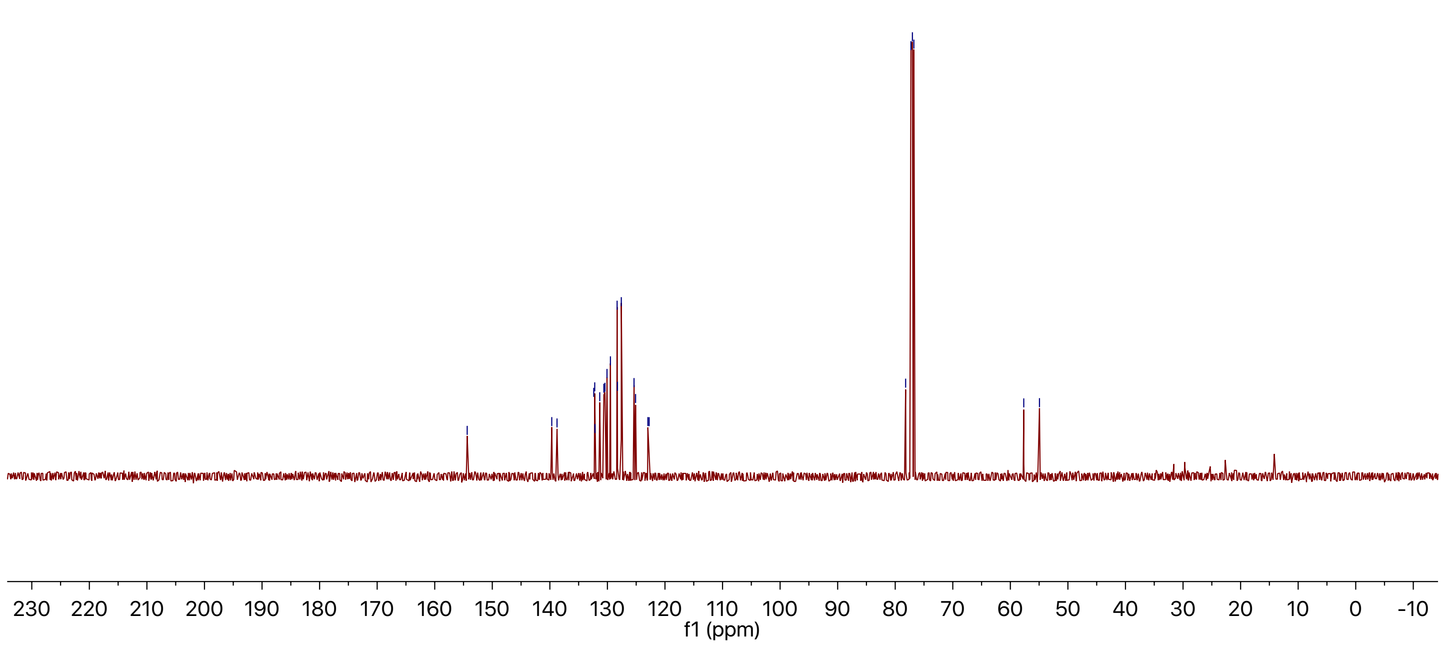
154.36
139.67
138.75
132.37
132.20
132.16
131.33
130.62
130.43
130.07
129.47
128.32
128.25
127.59
125.38
125.11
122.97
122.75
78.19
77.27
77.01
76.76
57.67
54.94
230 220 210 200 190 180 170 160 150 140 130 120 110 100 90 80 70 60 50 40 30 20 10 0 -10
f1 (ppm)

Compound 2e: (4*R*,5*S*,6*R*)-5-chloro-6-(2-fluorophenyl)-4-((*E*)-2-fluorostyryl)-2-phenyl-5,6-dihydro-4*H*-1,3-oxazine- ^{1}H NMR (500 MHz, $CDCl_3$); $^{13}C\{^{1}H\}$ NMR (126 MHz, $CDCl_3$)

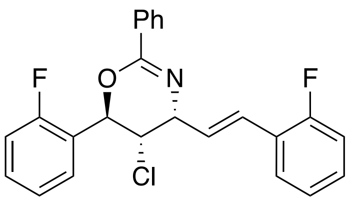

2e : ^{1}H nmr

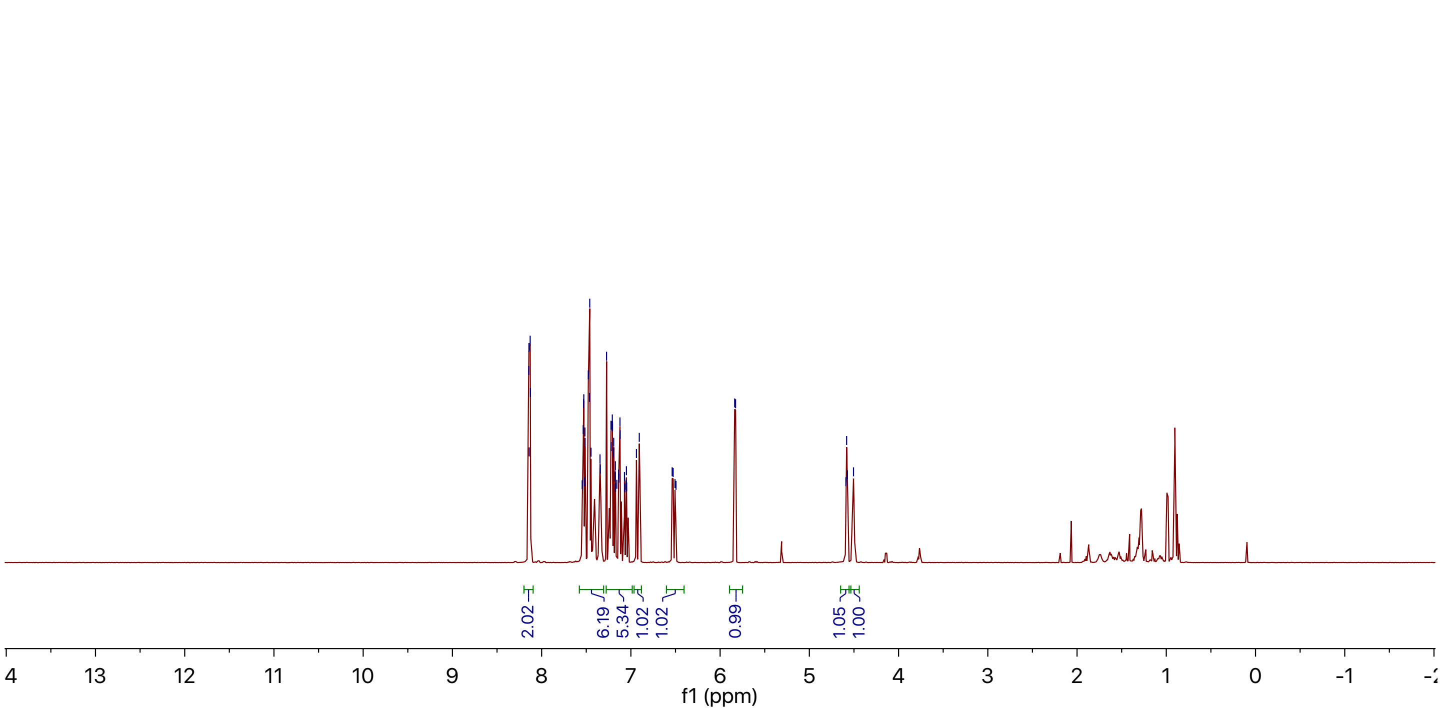

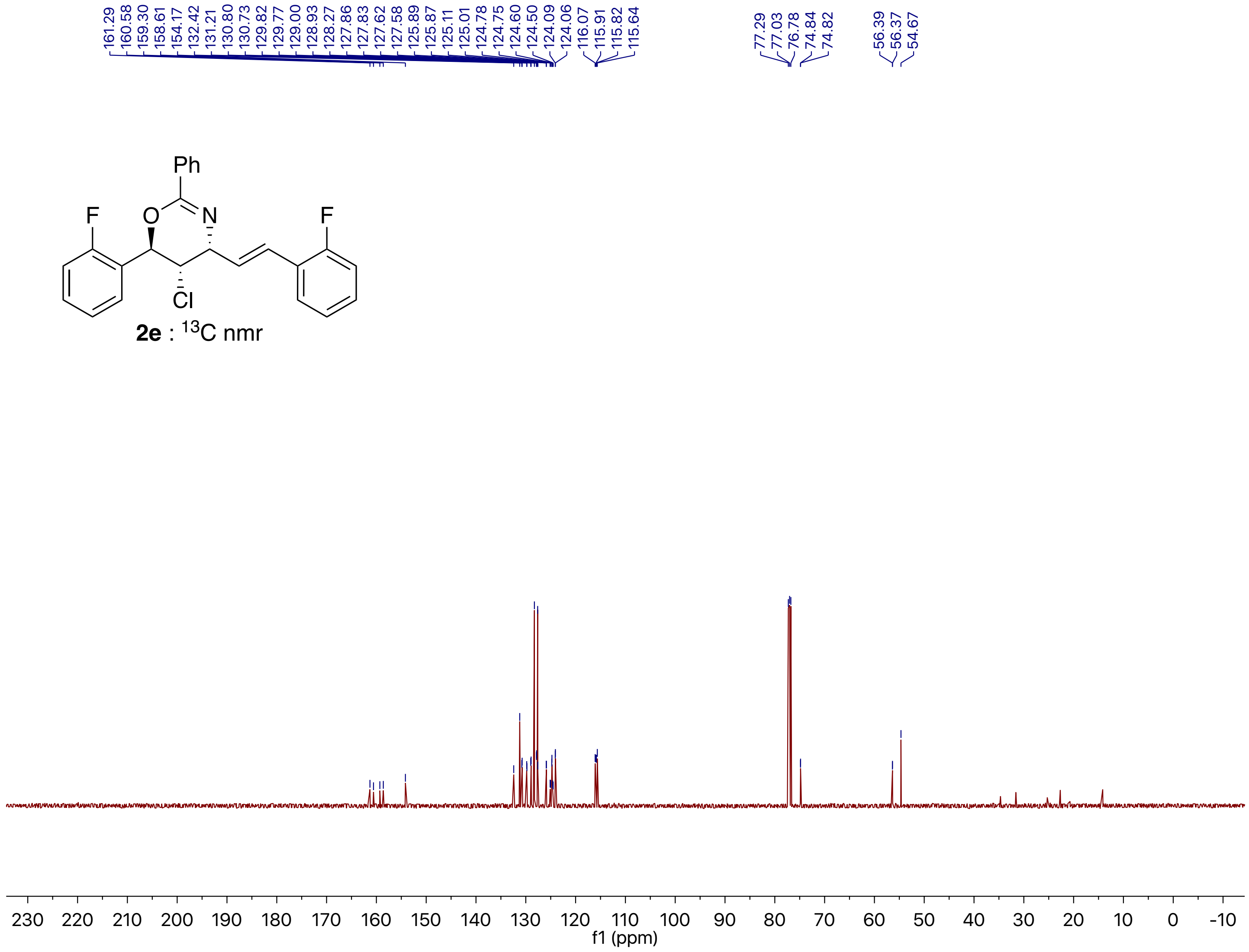
161.29
160.58
159.30
158.61
154.17
132.42
131.21
130.80
130.73
129.82
129.77
129.00
128.93
128.27
127.86
127.83
127.62
127.58
125.89
125.87
125.11
125.01
124.78
124.75
124.60
124.50
124.09
124.06
116.07
115.91
115.82
115.64
77.29
77.03
76.78
74.84
74.82
56.39
56.37
54.67
Ph
O
N
F
F
Cl
f1 (ppm)
230 220 210 200 190 180 170 160 150 140 130 120 110 100 90 80 70 60 50 40 30 20 10 0 -10

2e : ^{13}C nmr

Compound 2f: (4*R*,5*S*,6*R*)-5-chloro-6-(4-fluorophenyl)-4-((*E*)-4-fluorostyryl)-2-phenyl-5,6-dihydro-4*H*-1,3-oxazine- ^{1}H NMR (500 MHz, $CDCl_3$); $^{13}C\{^1H\}$ NMR (126 MHz, $CDCl_3$)

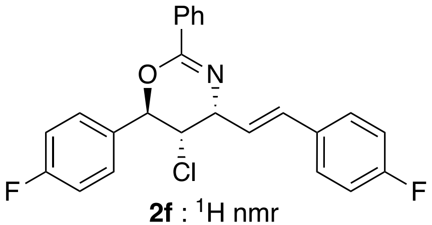

2f : [1]H nmr

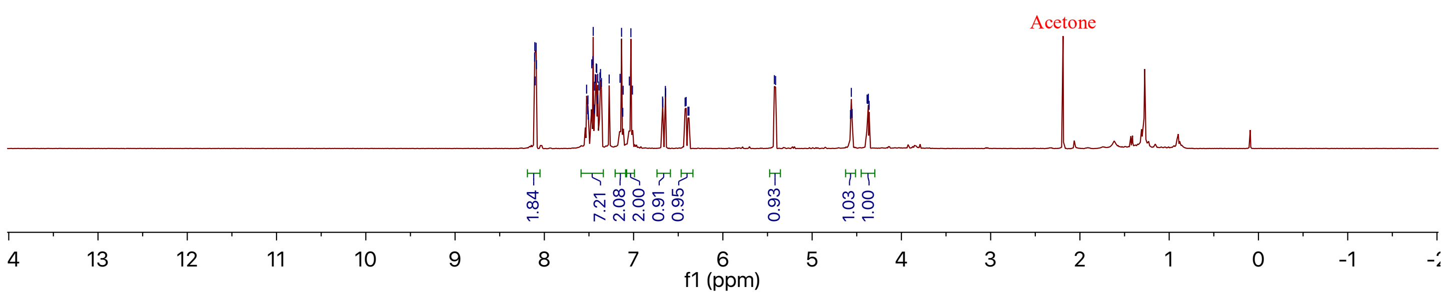

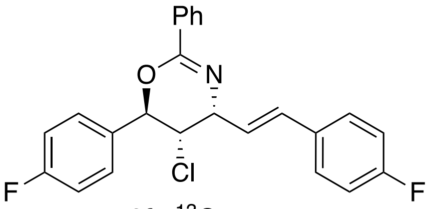
Ph
O
N
Cl
F
F

2f : ^{13}C nmr

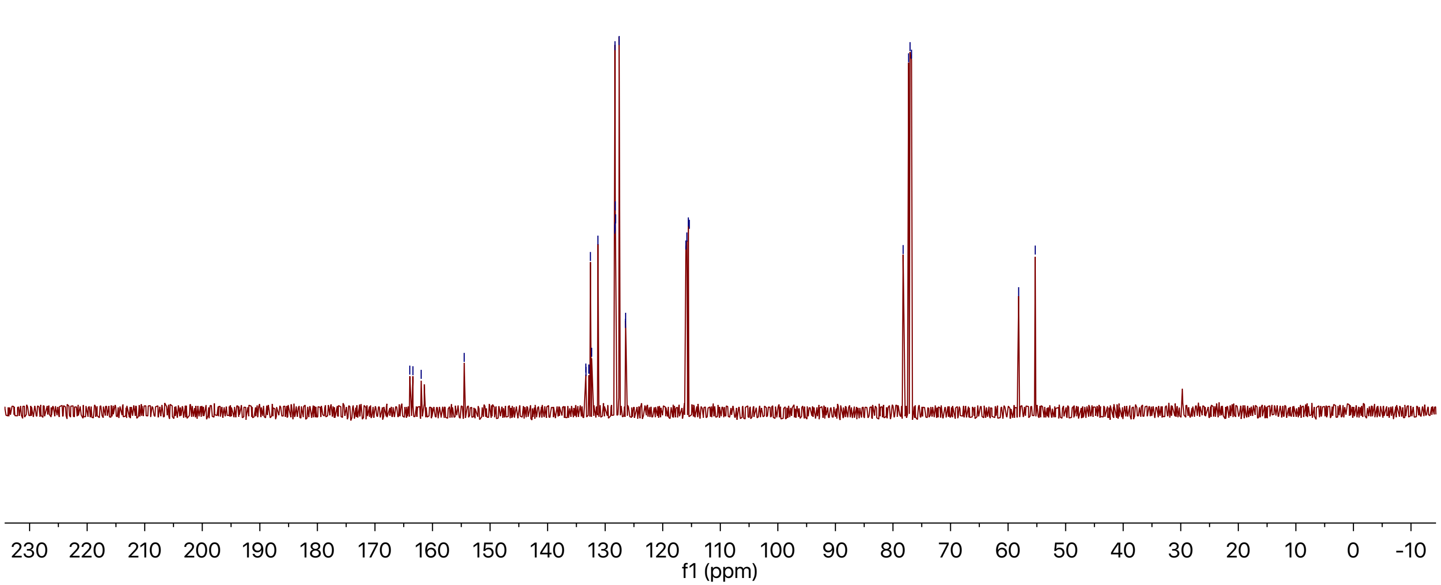
163.94
163.40
161.97
154.49
133.36
133.33
132.83
132.80
132.57
132.34
131.26
128.33
128.29
128.26
128.25
128.18
127.57
126.46
126.44
115.97
115.80
115.56
115.38
78.23
77.28
77.03
76.77
58.16
55.28
230 220 210 200 190 180 170 160 150 140 130 120 110 100 90 80 70 60 50 40 30 20 10 0 -10
f1 (ppm)

Compound 2g: (4*R*,5*S*,6*R*)-5-chloro-2-phenyl-6-(2,6-dichlorophenyl)-4-((*E*)-(2,6-dichloro)styryl)-5,6-dihydro-4*H*-1,3-oxazine-^{1}H NMR (500 MHz, $CDCl_3$); $^{13}C\{^{1}H\}$ NMR (126 MHz, $CDCl_3$)

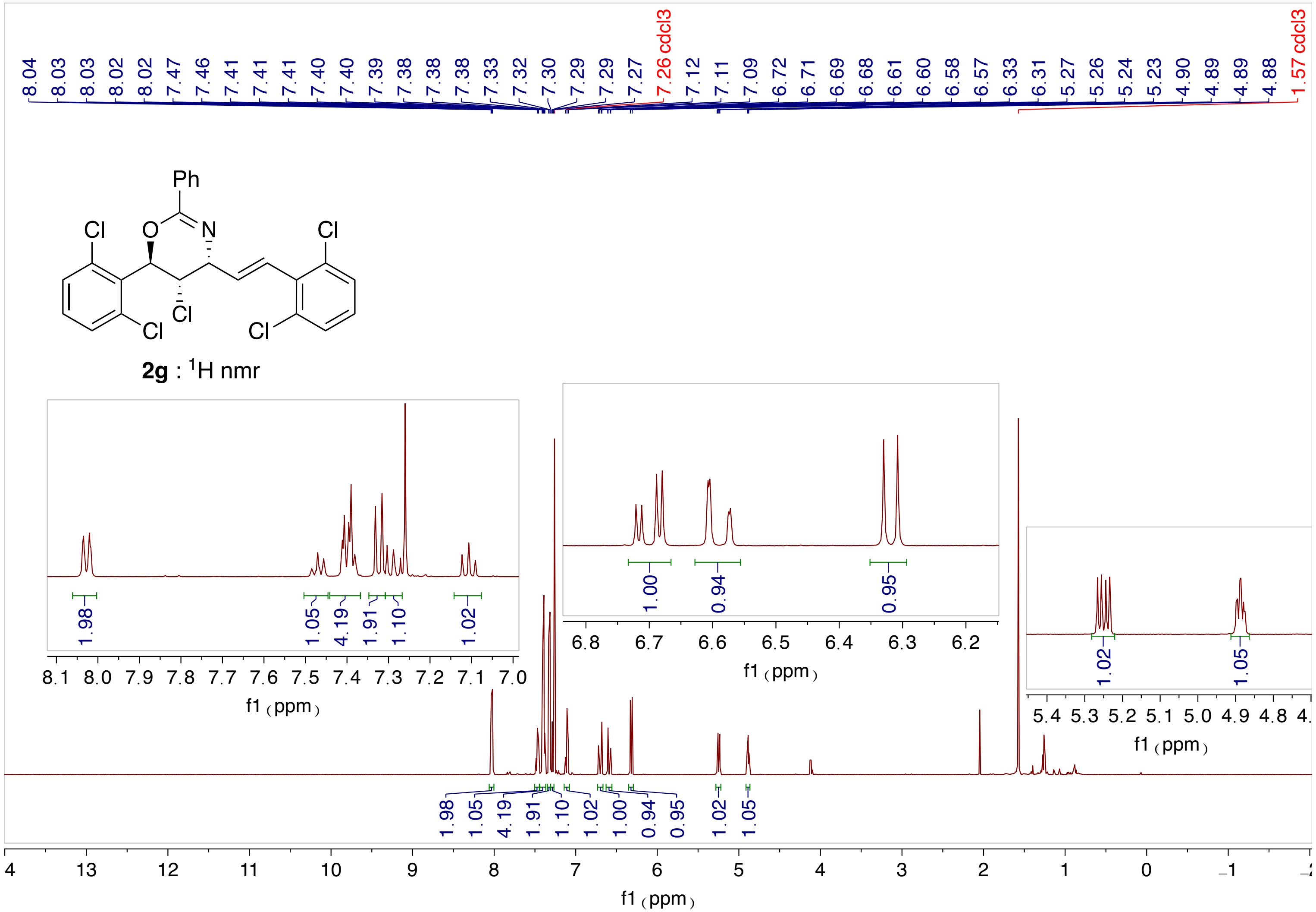

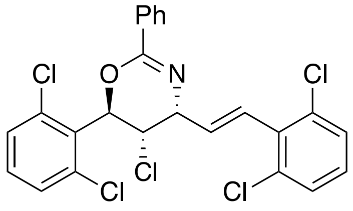

Ph
O
N
Cl
Cl
Cl
Cl
Cl

2g : ^{13}C nmr

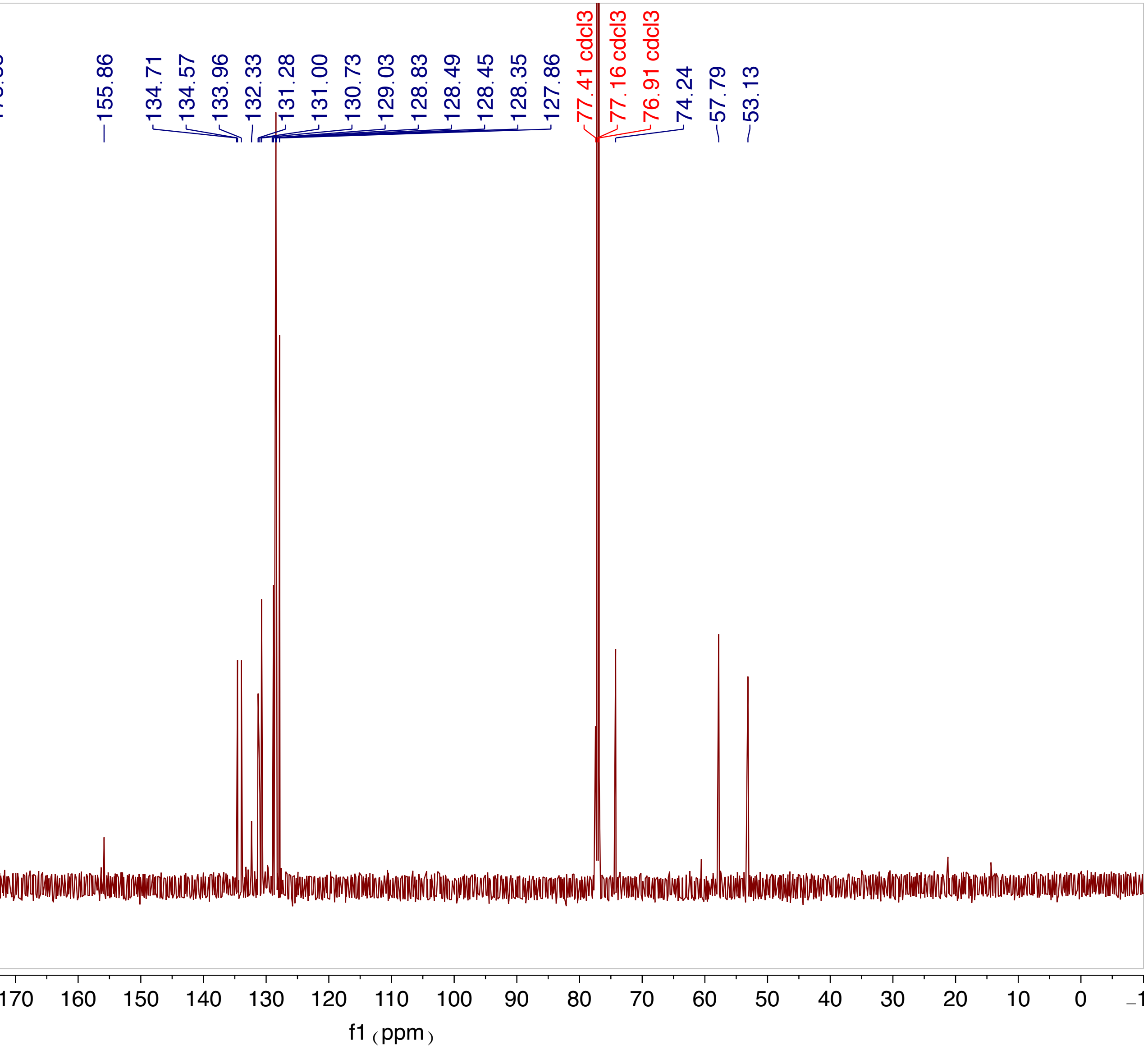

173.35
155.86
134.71
134.57
133.96
132.33
131.28
131.00
130.73
129.03
128.83
128.49
128.45
128.35
127.86
77.41 cdcl3
77.16 cdcl3
76.91 cdcl3
74.24
57.79
53.13
f1 (ppm)

Compound 2h: (4*R*,5*S*,6*R*)-5-chloro-2-phenyl-6-(4-(trifluoromethyl)phenyl)-4-((*E*)-4-(trifluoromethyl)styryl)-5,6-dihydro-4*H*-1,3-oxazine-^{1}H NMR (500 MHz, $CDCl_3$); $^{13}C\{^{1}H\}$ NMR (126 MHz, $CDCl_3$)

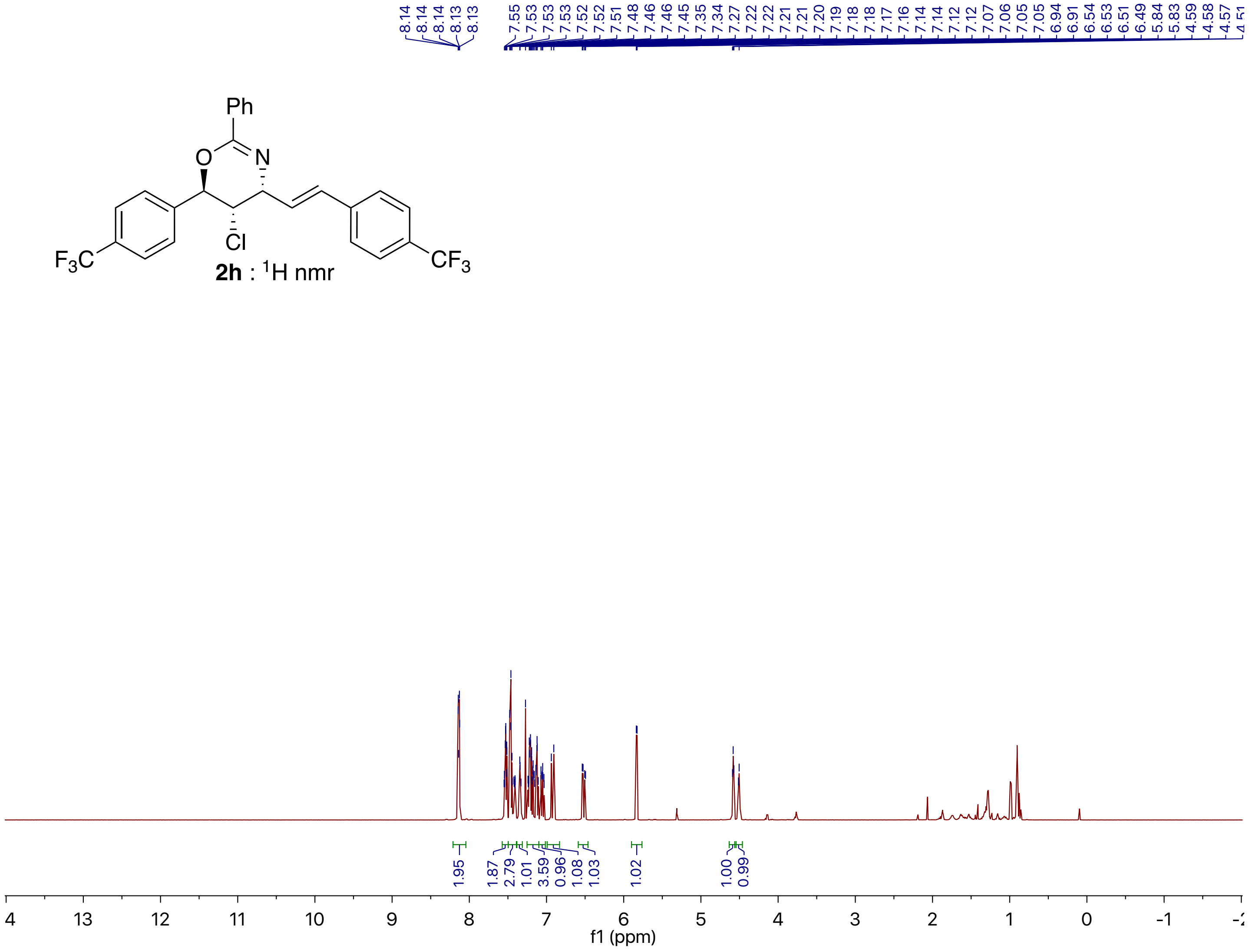

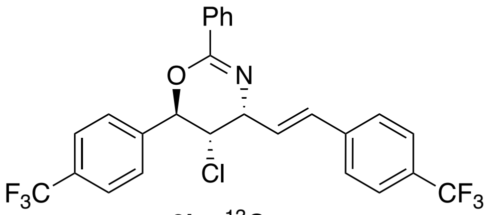
Ph
O
N
Cl
F3C
CF3

2h : ^{13}C nmr

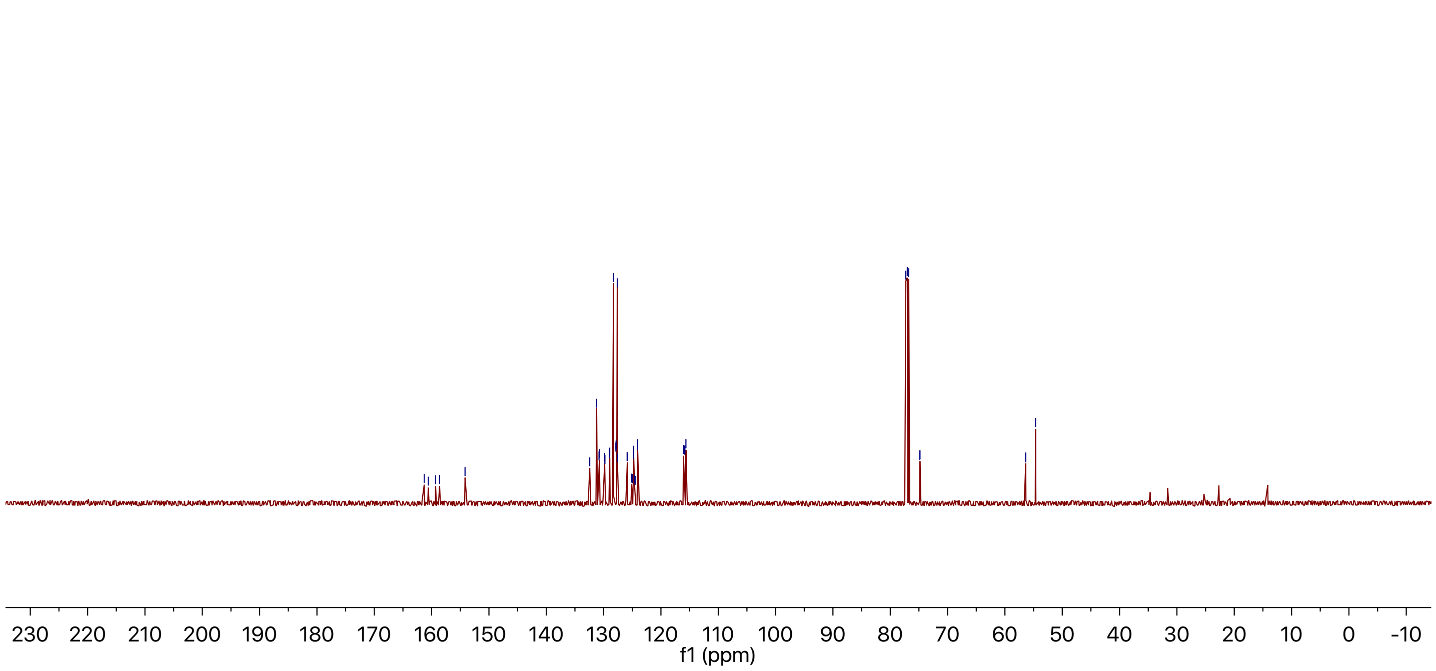
161.29
160.58
159.30
158.61
154.17
132.42
131.21
130.80
130.73
129.82
129.77
129.00
128.93
128.27
127.86
127.83
127.62
127.58
125.89
125.87
125.11
125.01
124.78
124.75
124.60
124.50
124.09
124.06
116.07
115.91
115.82
115.64
77.29
77.03
76.78
74.84
74.82
56.39
56.37
54.67
230 220 210 200 190 180 170 160 150 140 130 120 110 100 90 80 70 60 50 40 30 20 10 0 -10
f1 (ppm)

Compound 2i: (4*S*,5*R*,6*S*)-5-chloro-6-(3-methoxyphenyl)-4-((*E*)-3-methoxystyryl)-2-phenyl-5,6-dihydro-4*H*-1,3-oxazine- ^{1}H NMR (500 MHz, $CDCl_3$); $^{13}C\{^{1}H\}$ NMR (126 MHz, $CDCl_3$)

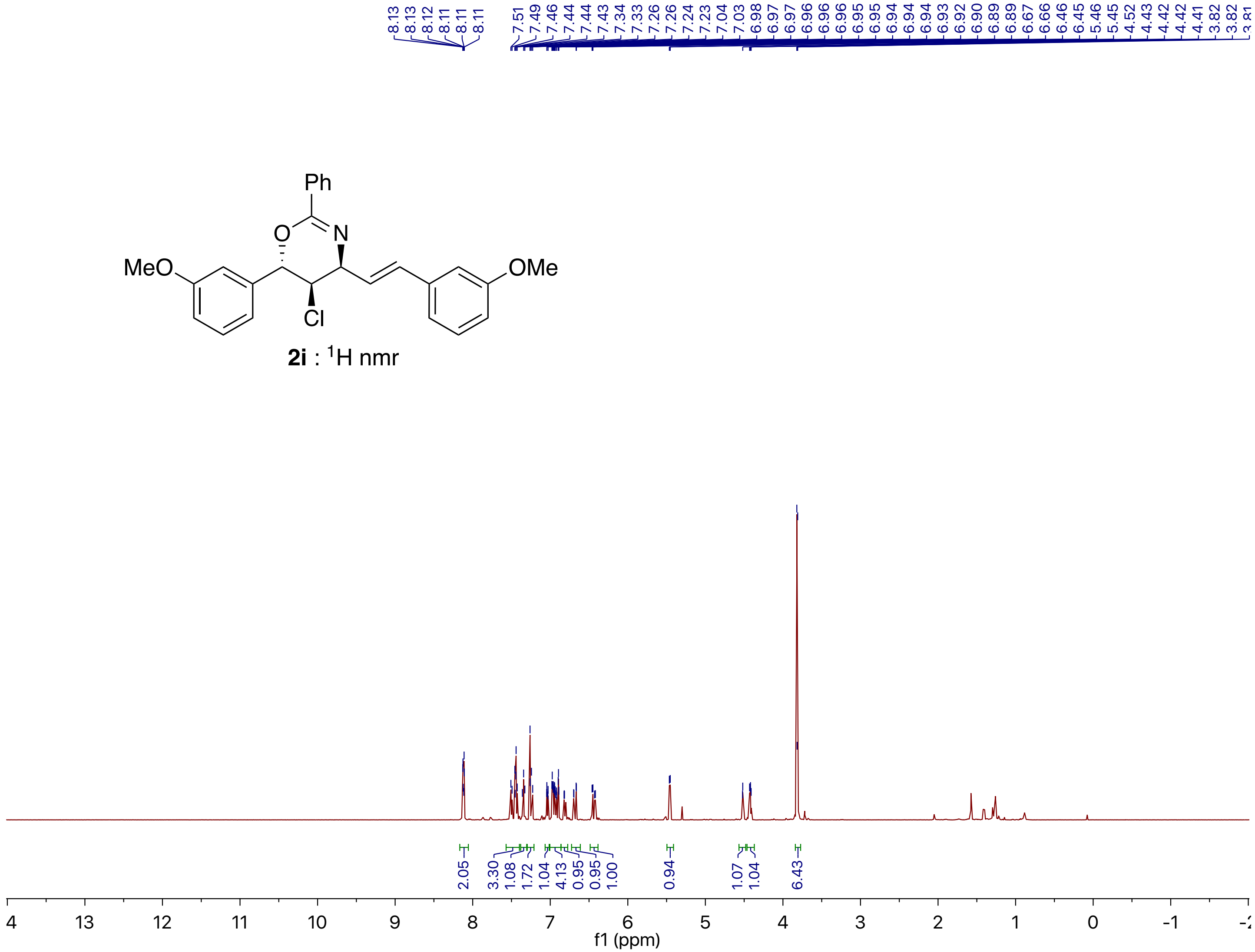

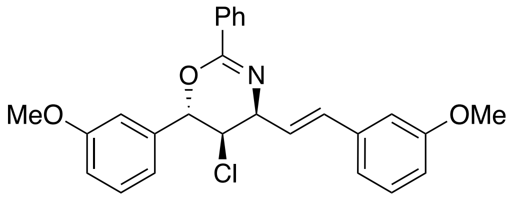

2i : ^{13}C nmr

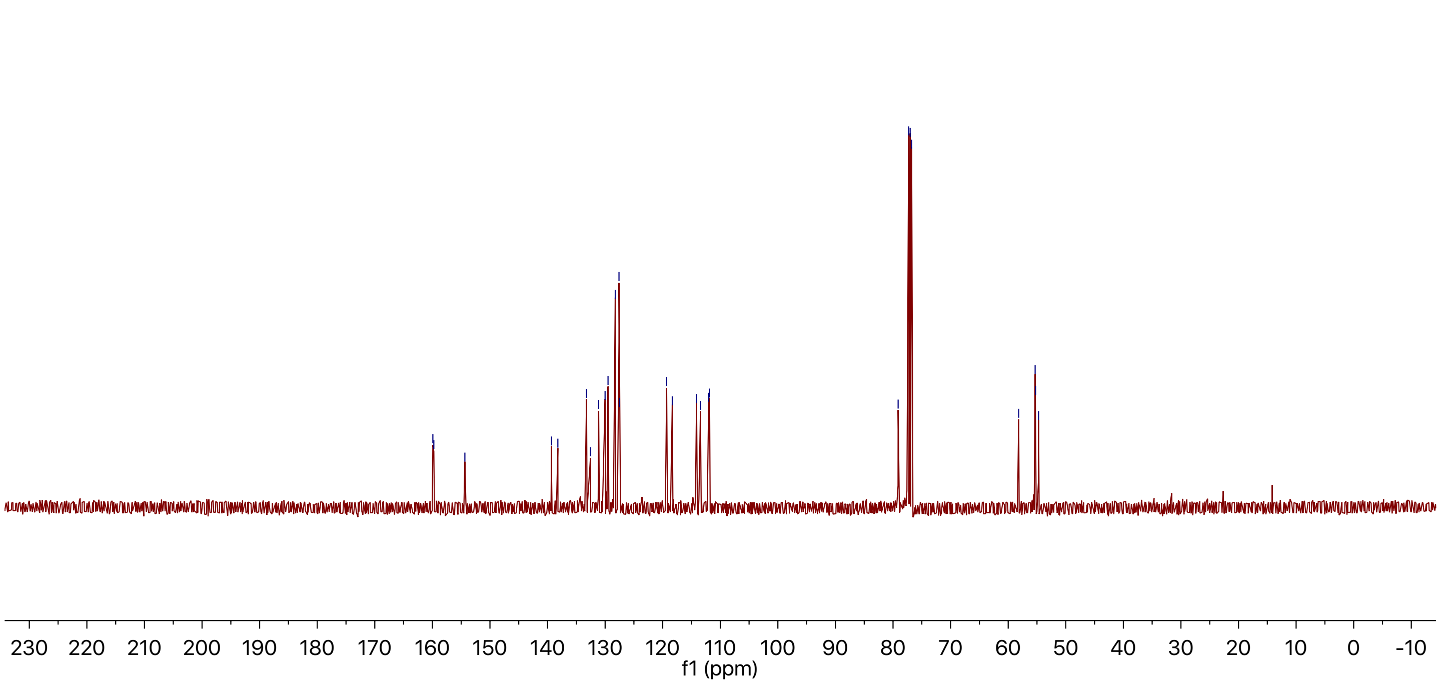
159.92
159.75
154.36
139.31
138.21
133.23
132.55
131.12
130.01
129.50
128.24
127.59
127.51
119.31
118.34
114.12
113.45
112.02
111.86
79.12
77.28
77.03
76.77
58.17
55.32
55.25
54.72
230 220 210 200 190 180 170 160 150 140 130 120 110 100 90 80 70 60 50 40 30 20 10 0 -10
f1 (ppm)

Compound 2j: (4*R*,5*S*,6*R*)-5-chloro-4-((*E*)-2-methylstyryl)-2-phenyl-6-(*o*-tolyl)-5,6-dihydro-4*H*-1,3-oxazine- ^{1}H NMR (500 MHz, CDCl$_3$); ^{13}C{^{1}H} NMR (126 MHz, CDCl$_3$)

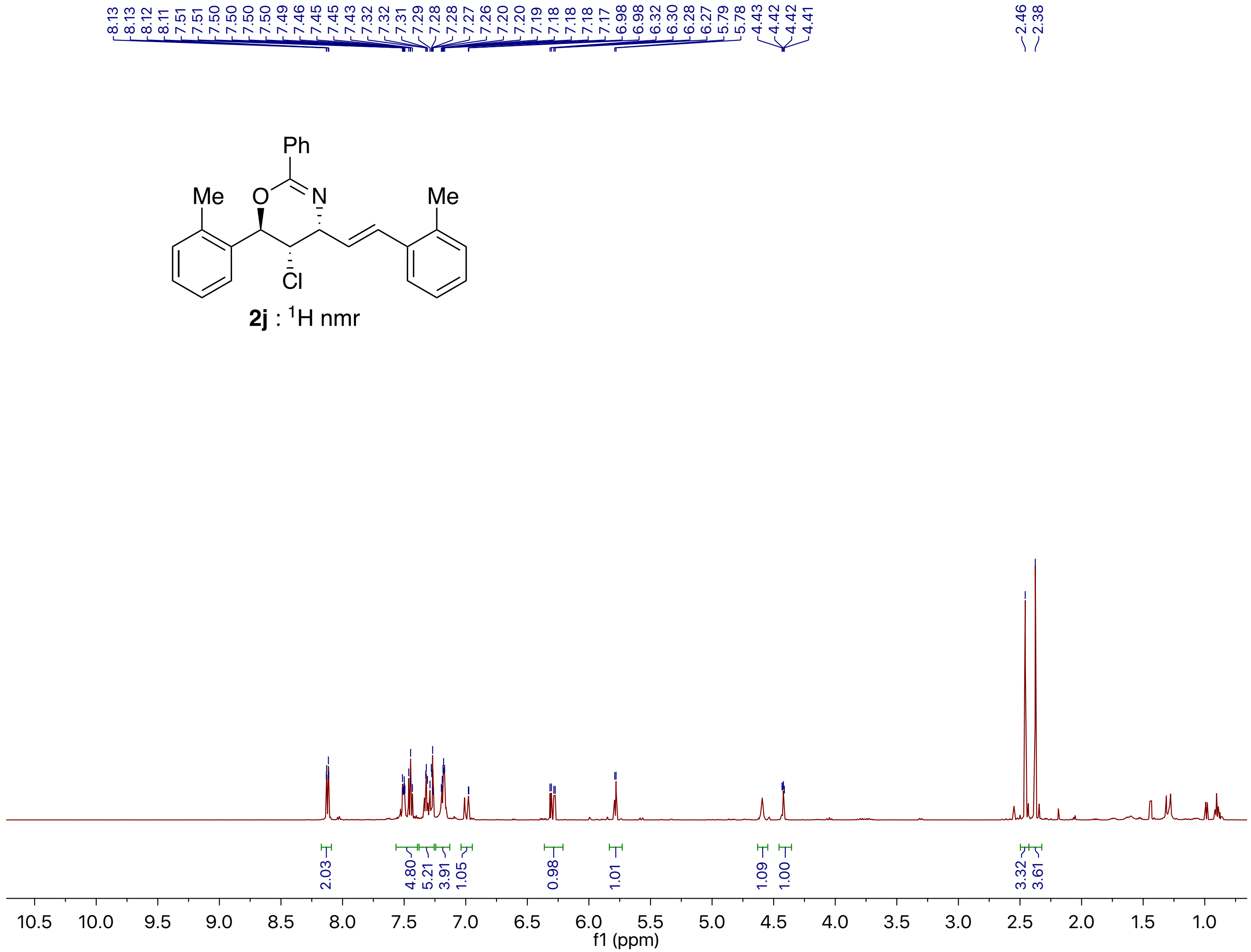

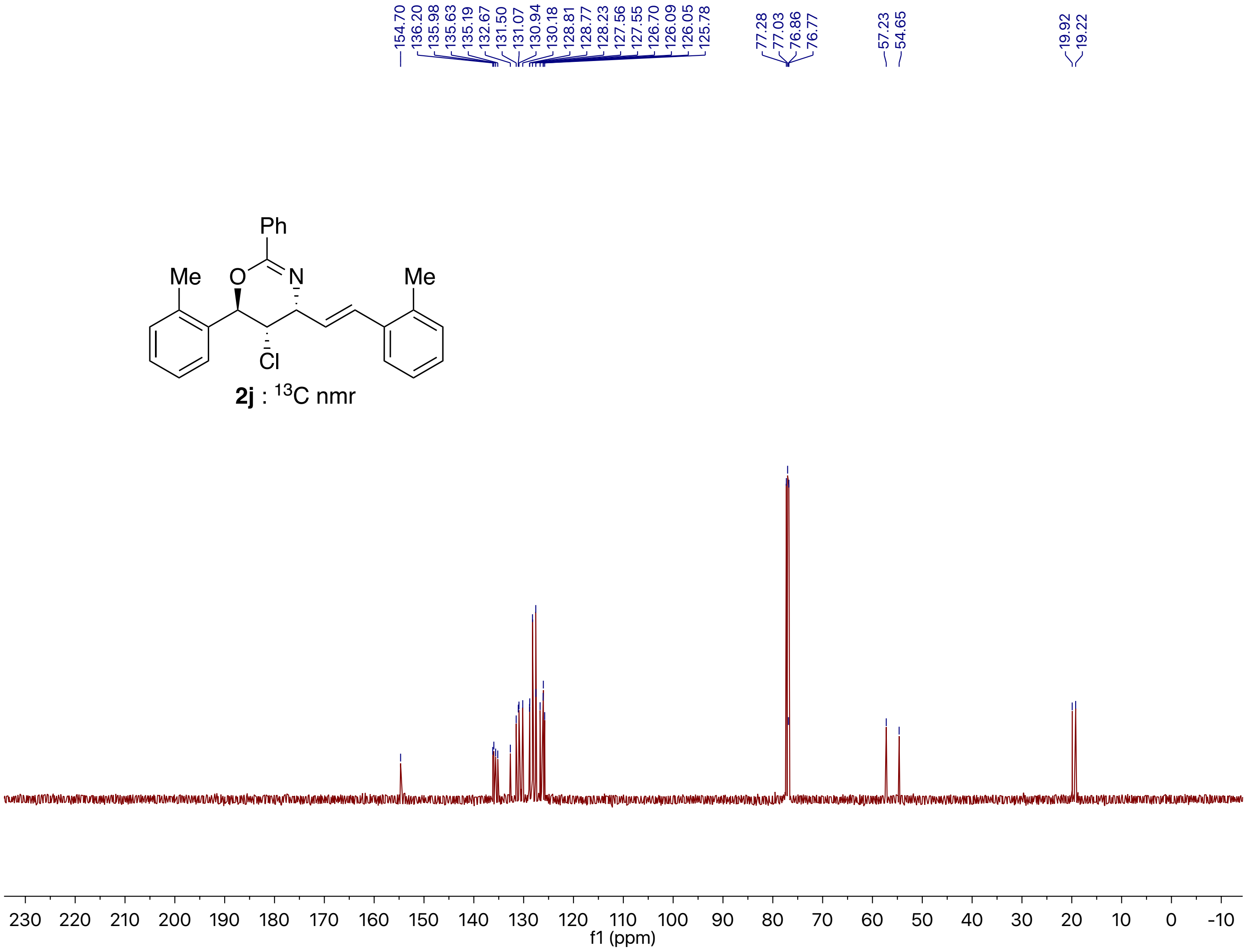
Ph
O
N
Me
Me
Cl
154.70
136.20
135.98
135.63
135.19
132.67
131.50
131.07
130.94
130.18
128.81
128.77
128.23
127.56
127.55
126.70
126.09
126.05
125.78
77.28
77.03
76.86
76.77
57.23
54.65
19.92
19.22
230 220 210 200 190 180 170 160 150 140 130 120 110 100 90 80 70 60 50 40 30 20 10 0 -10
f1 (ppm)

2j : ^{13}C nmr

Compound 2k: (4*R*,5*S*,6*R*)-5-chloro-4-((*E*)-4-methylstyryl)-2-phenyl-6-(*p*-tolyl)-5,6-dihydro-4*H*-1,3-oxazine- ^{1}H NMR (500 MHz, $CDCl_3$); $^{13}C\{^{1}H\}$ NMR (126 MHz, $CDCl_3$)

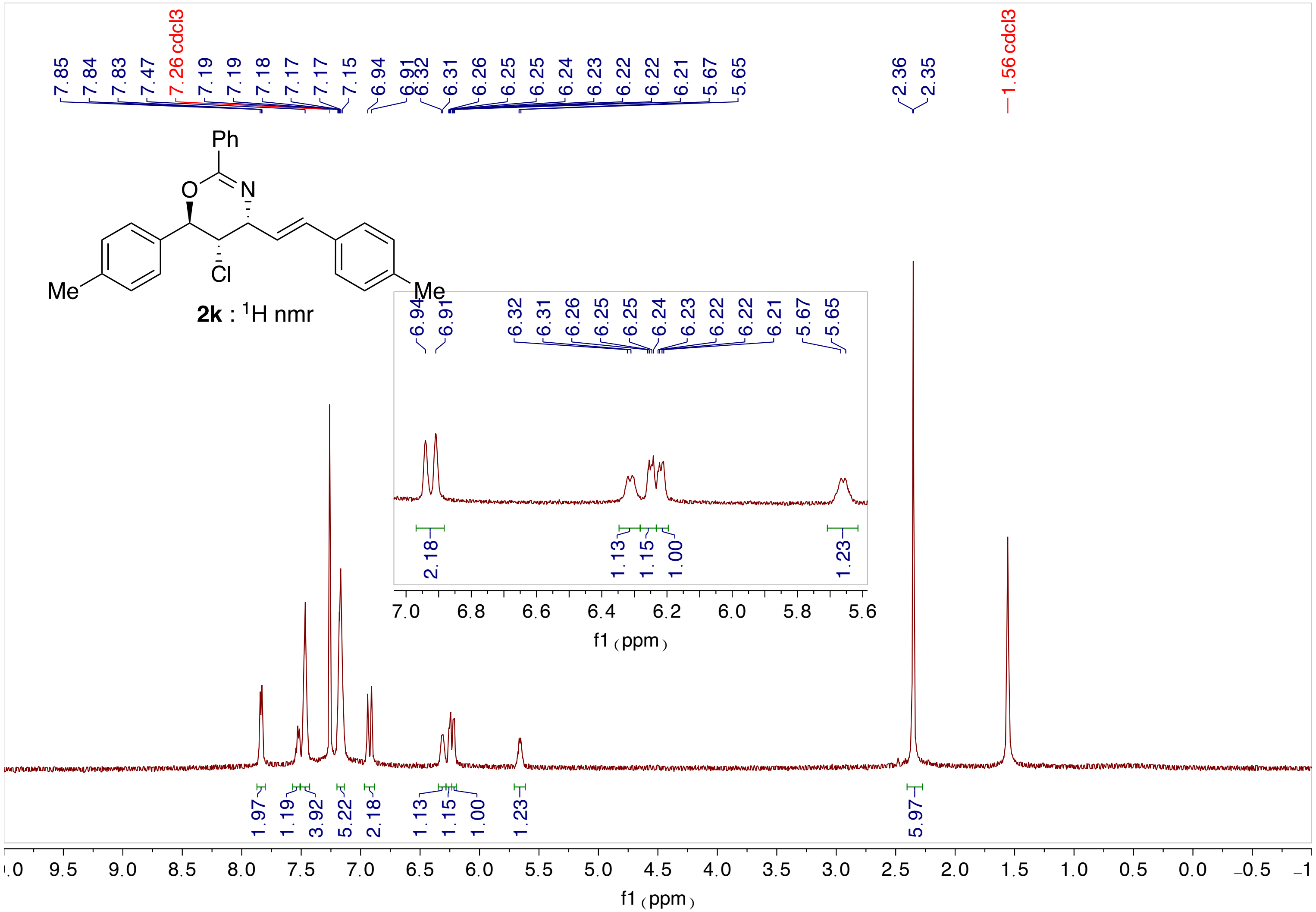

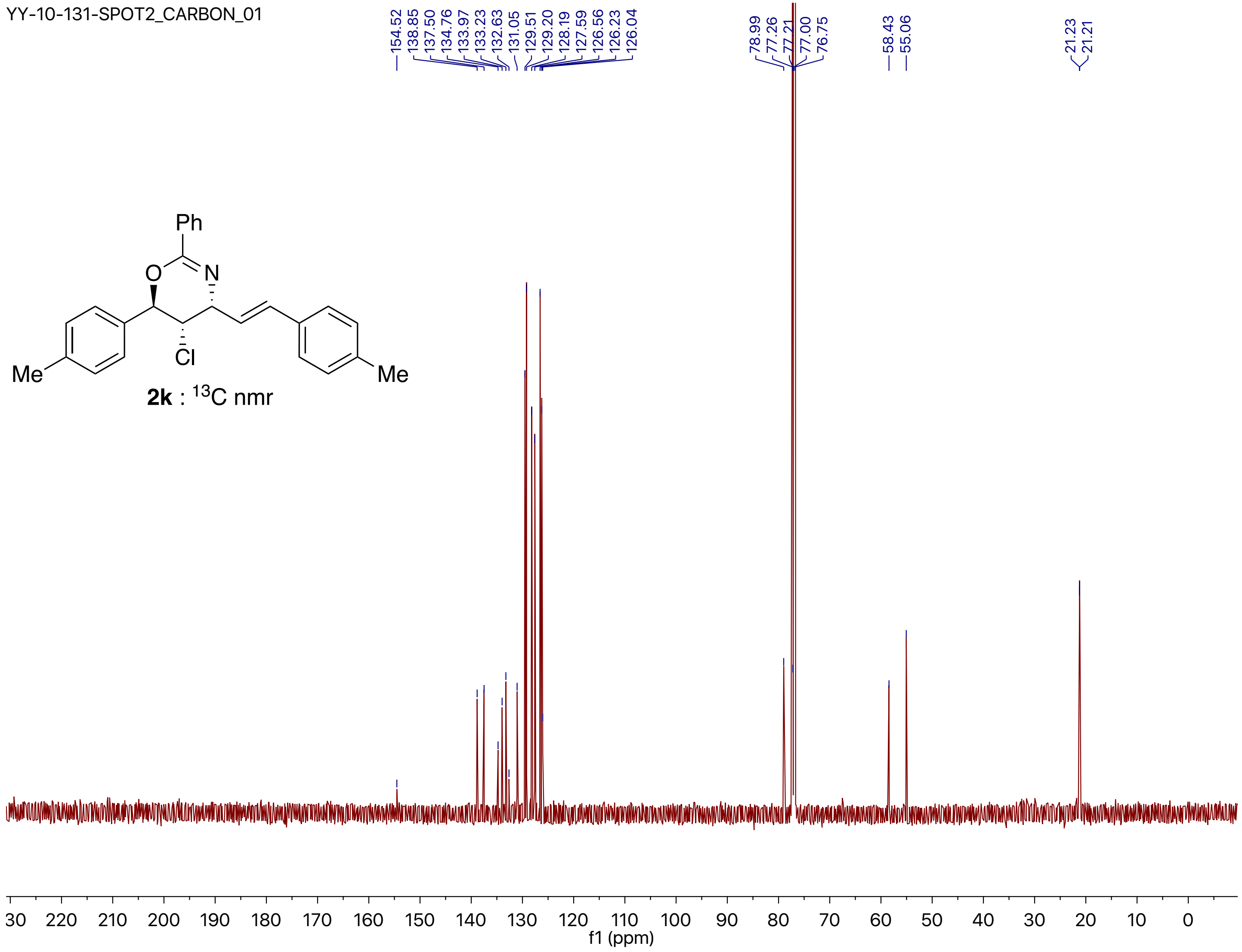
YY-10-131-SPOT2_CARBON_01
154.52
138.85
137.50
134.76
133.97
133.23
132.63
131.05
129.51
129.20
128.19
127.59
126.56
126.23
126.04
78.99
77.26
77.21
77.00
76.75
58.43
55.06
21.23
21.21
Ph
O
N
Cl
Me
Me
2k : ^{13}C nmr
30 220 210 200 190 180 170 160 150 140 130 120 110 100 90 80 70 60 50 40 30 20 10 0
f1 (ppm)

Compound 2l: (4*R*,5*S*,6*R*)-5-chloro-6-(4-methoxyphenyl)-4-((*E*)-4-methoxystyryl)-2-phenyl-5,6-dihydro-4*H*-1,3-oxazine- ^{1}H NMR (500 MHz, CDCl$_3$); ^{13}C{^{1}H} NMR (126 MHz, CDCl$_3$)

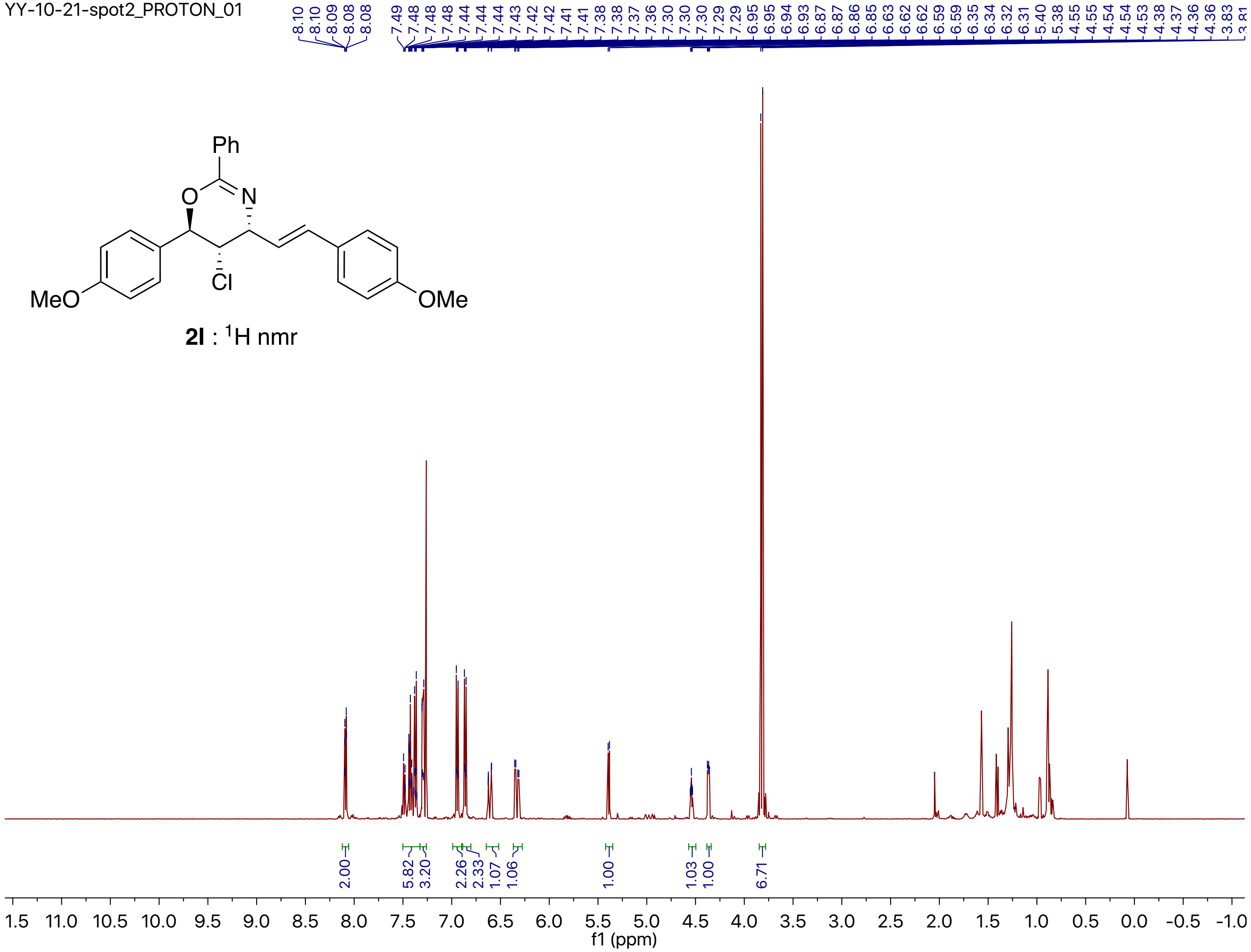

2l : ^{1}H nmr

YY-10-21-spot2_CARBON_01

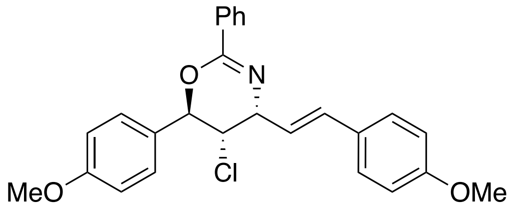

2l : ^{13}C nmr

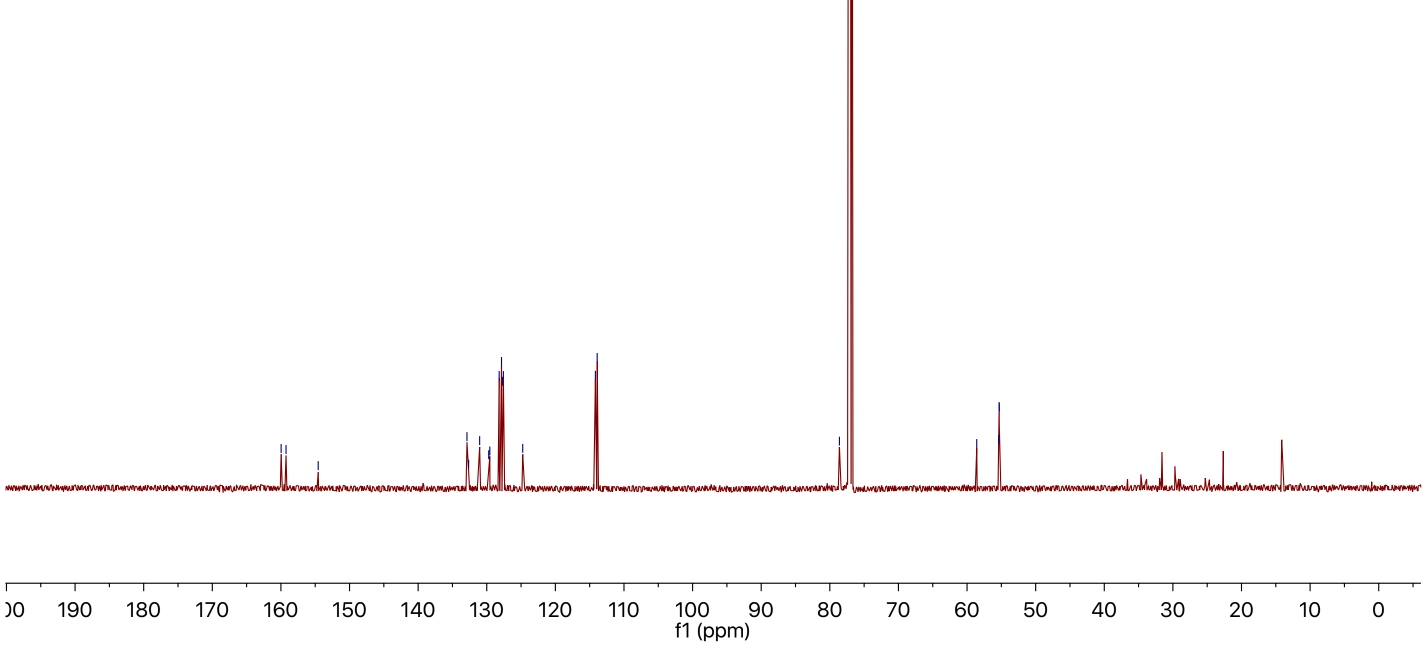

Compound 2m: (4*R*,5*S*,6*R*)-6-(4-(*tert*-butyl)phenyl)-4-((*E*)-4-(*tert*-butyl)styryl)-5-chloro-2-phenyl-5,6-dihydro-4*H*-1,3-oxazine- ^{1}H NMR (500 MHz, $CDCl_3$); $^{13}C\{^{1}H\}$ NMR (126 MHz, $CDCl_3$)

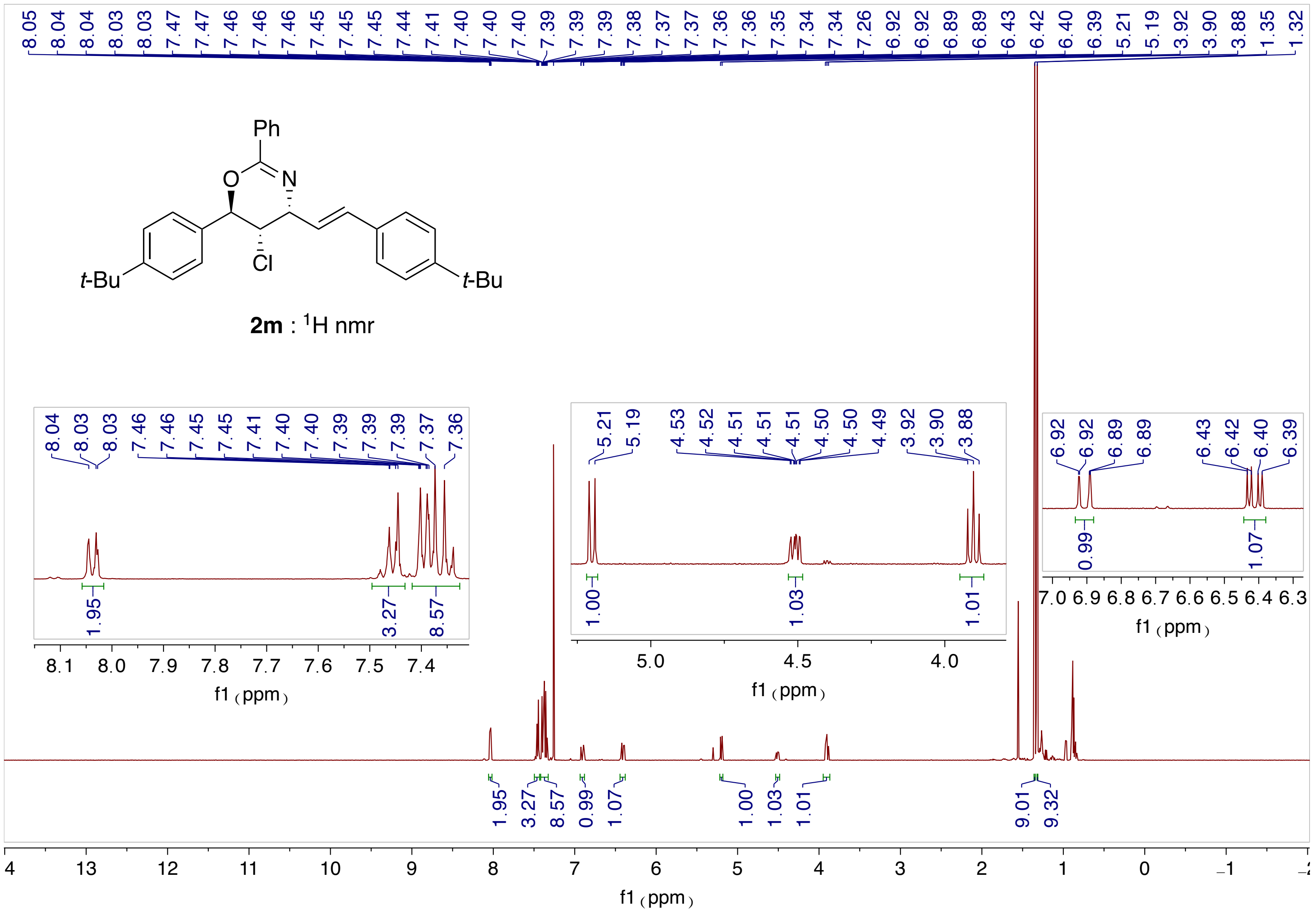

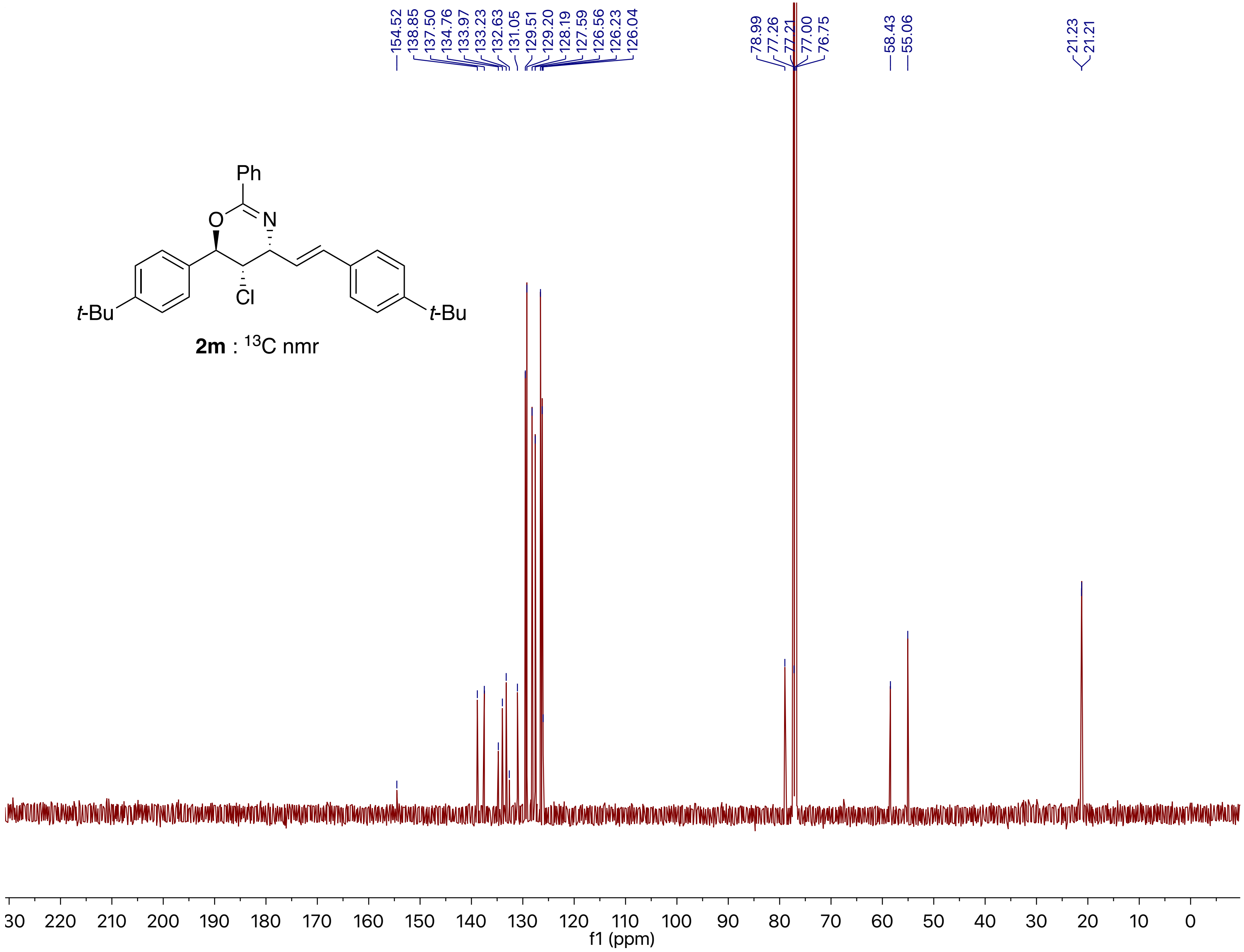
154.52
138.85
137.50
134.76
133.97
133.23
132.63
131.05
129.51
129.20
128.19
127.59
126.56
126.23
126.04
78.99
77.26
77.21
77.00
76.75
58.43
55.06
21.23
21.21
Ph
O
N
t-Bu
Cl
t-Bu
2m : 13C nmr
f1 (ppm)

Compound 2n: (4*R*,5*S*,6*R*)-5-chloro-2-phenyl-6-(thiophen-2-yl)-4-((*E*)-2-(thiophen-2-yl)vinyl)-5,6-dihydro-4*H*-1,3-oxazine- ^{1}H NMR (500 MHz, $CDCl_3$); $^{13}C\{^1H\}$ NMR (126 MHz, $CDCl_3$)

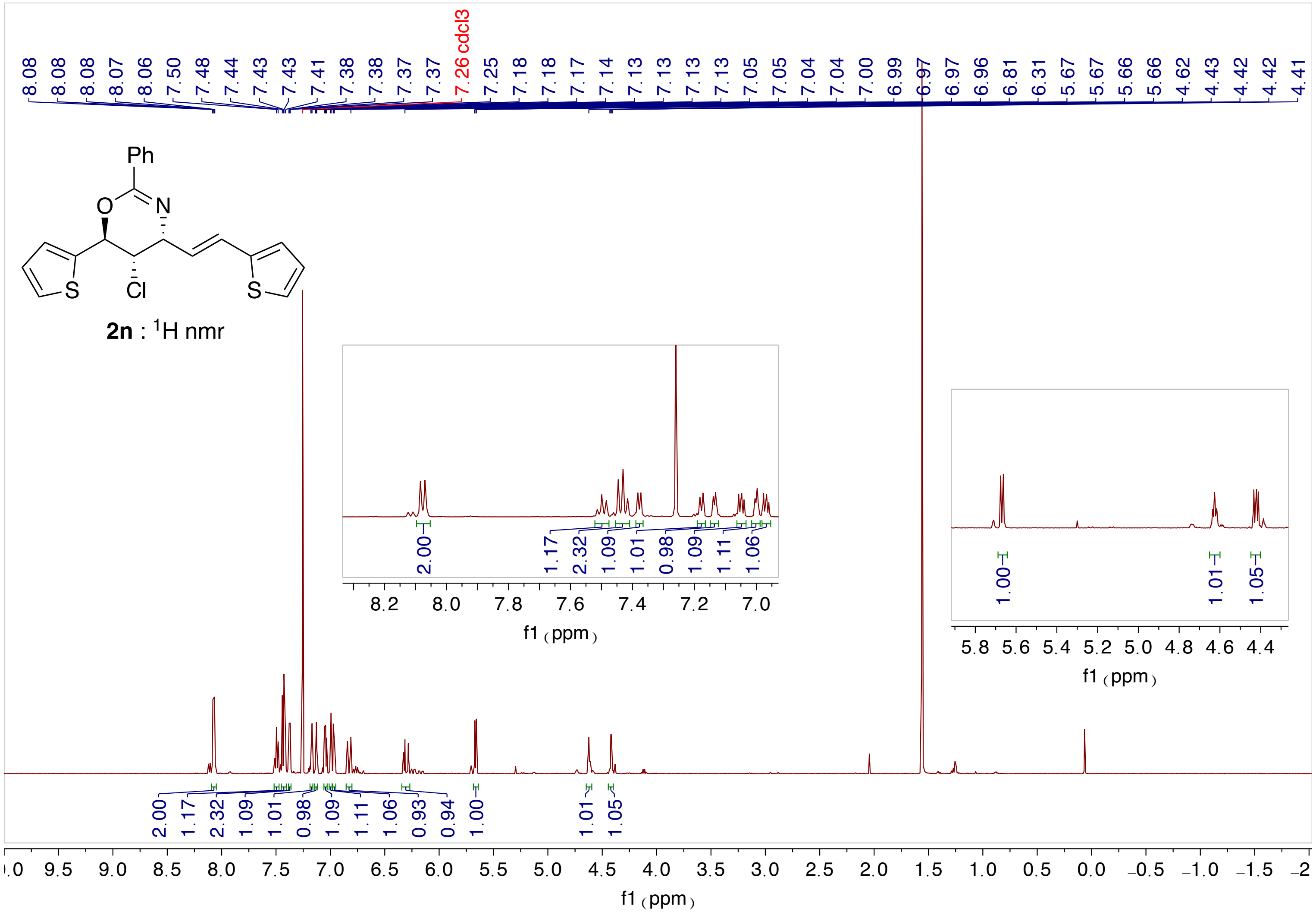

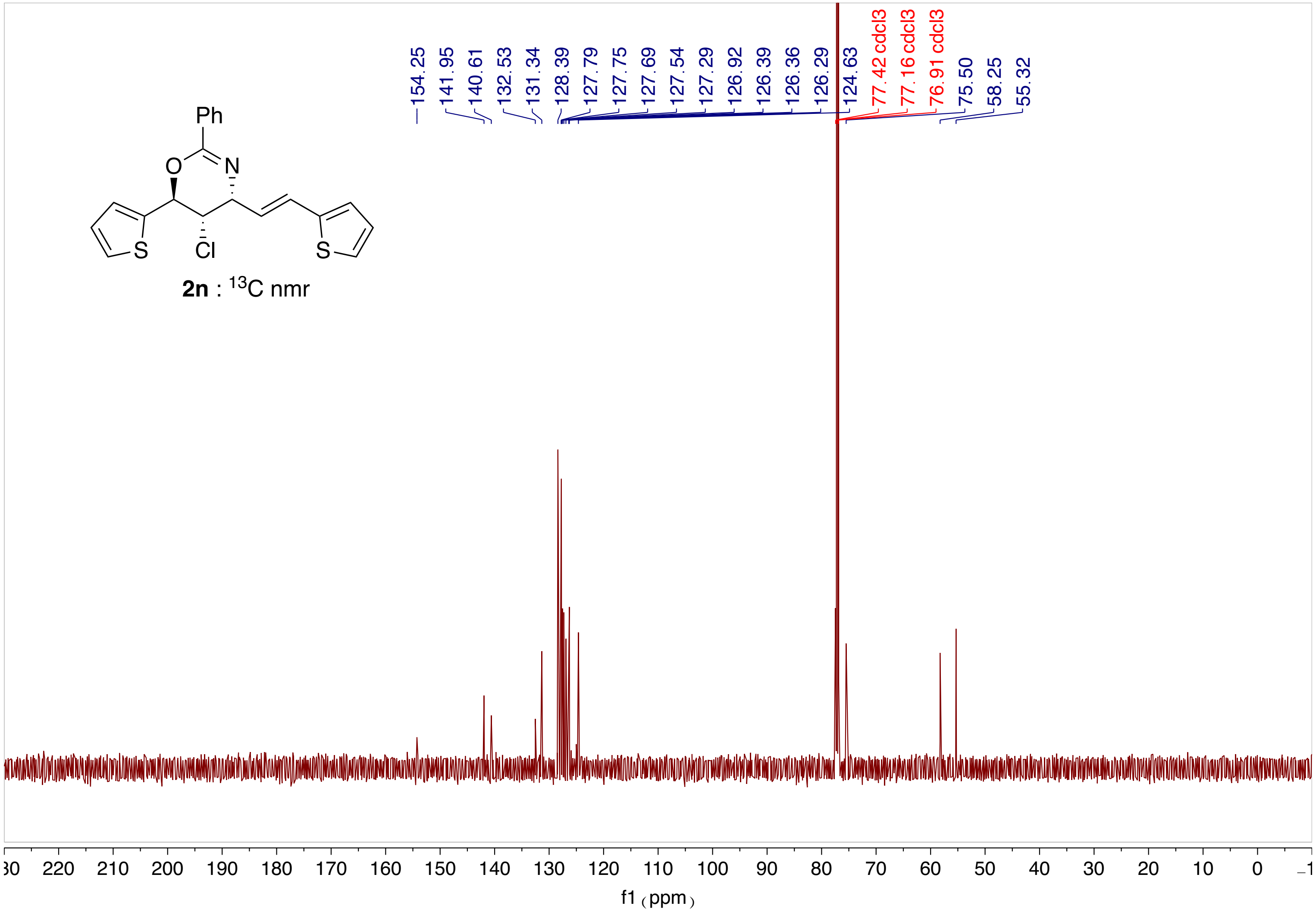
Ph
O
N
S
Cl
S
2n : 13C nmr
154.25
141.95
140.61
132.53
131.34
128.39
127.79
127.75
127.69
127.54
127.29
126.92
126.39
126.36
126.29
124.63
77.42 cdcl3
77.16 cdcl3
76.91 cdcl3
75.50
58.25
55.32
f1 (ppm)

Compound 2o: (4*R*,5*S*,6*R*)-6-butyl-5-chloro-4-((*E*)-hex-1-en-1-yl)-2-phenyl-5,6-dihydro-4*H*-1,3-oxazine- ^{1}H NMR (500 MHz, $CDCl_3$); $^{13}C\{^{1}H\}$ NMR (126 MHz, $CDCl_3$)

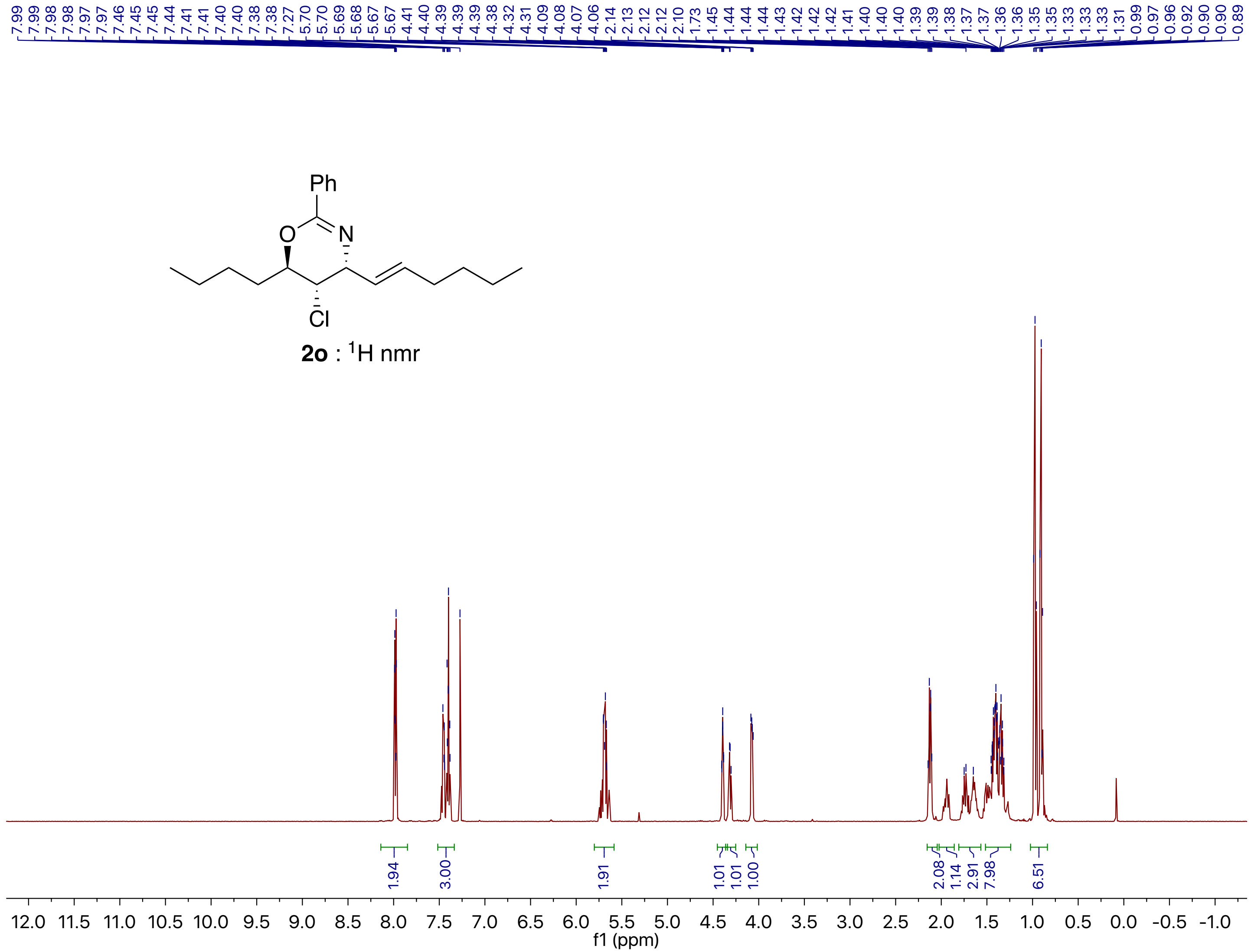

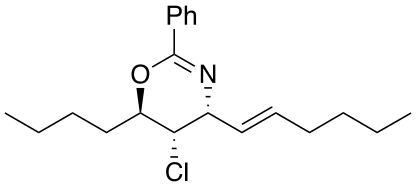

2o : ^{13}C nmr

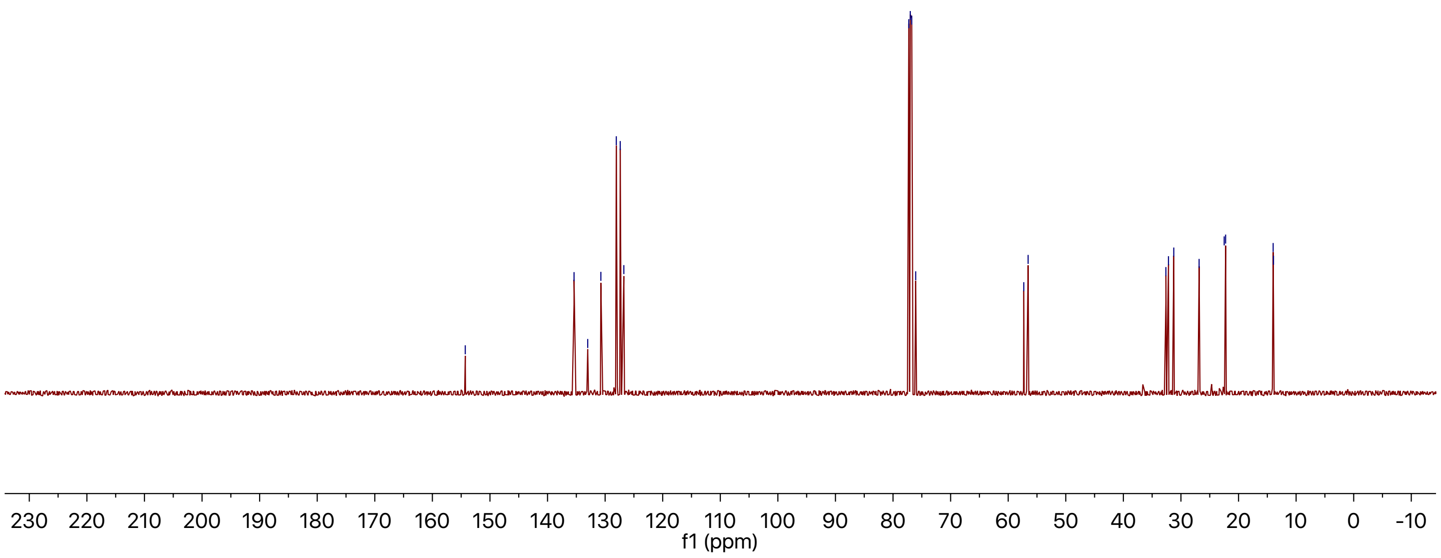
154.29
135.40
133.02
130.74
128.06
127.38
126.75
77.26
77.00
76.75
76.06
57.29
56.54
32.61
32.17
31.24
26.85
22.49
22.24
13.99
13.93
230 220 210 200 190 180 170 160 150 140 130 120 110 100 90 80 70 60 50 40 30 20 10 0 -10
f1 (ppm)

Compound 2p: (4*R*,5*S*,6*R*)-5-chloro-6-cyclohexyl-4-((*E*)-2-cyclohexylvinyl)-2-phenyl-5,6-dihydro-4*H*-1,3-oxazine- ^{1}H NMR (500 MHz, $CDCl_3$); $^{13}C\{^{1}H\}$ NMR (126 MHz, $CDCl_3$)

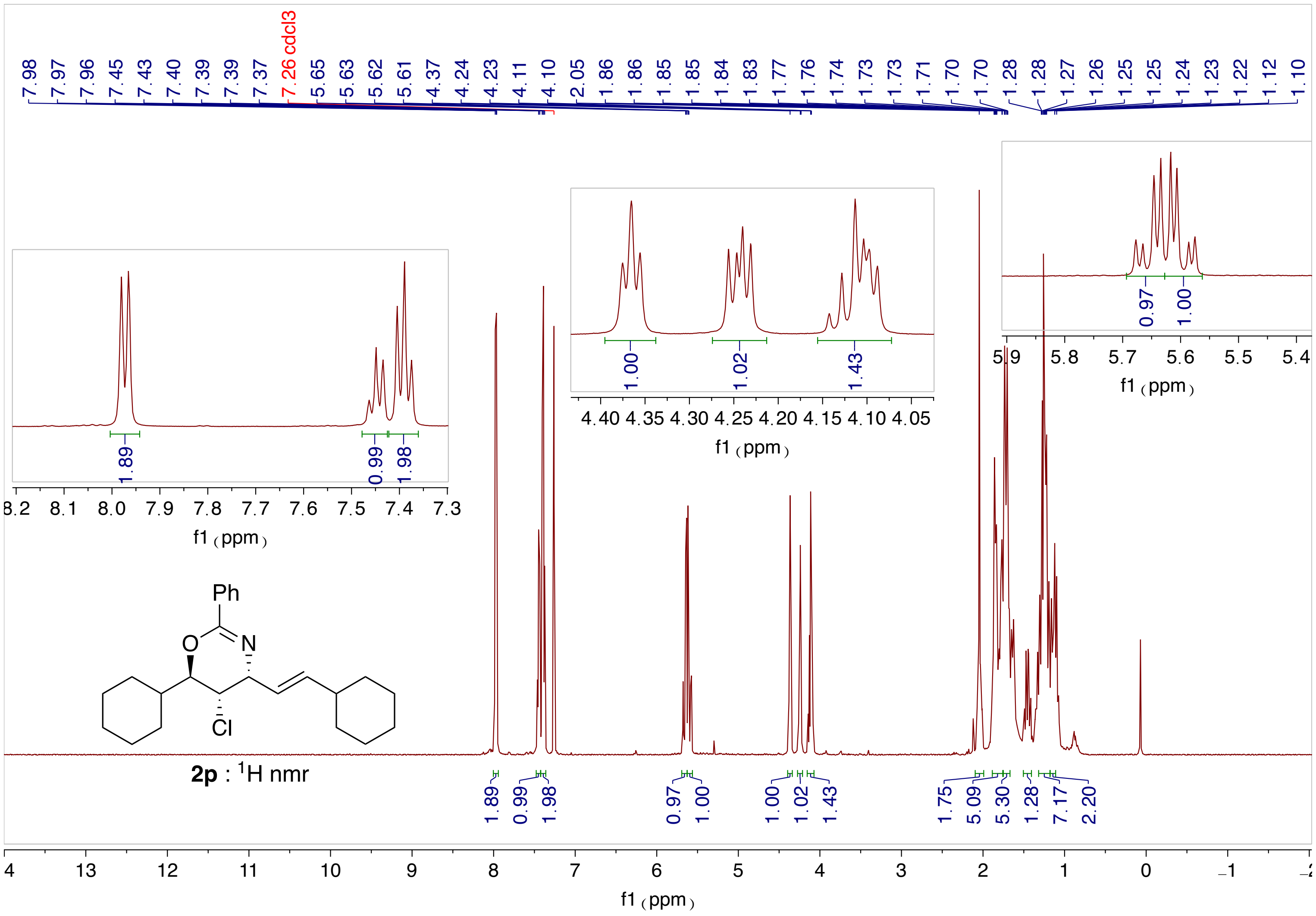

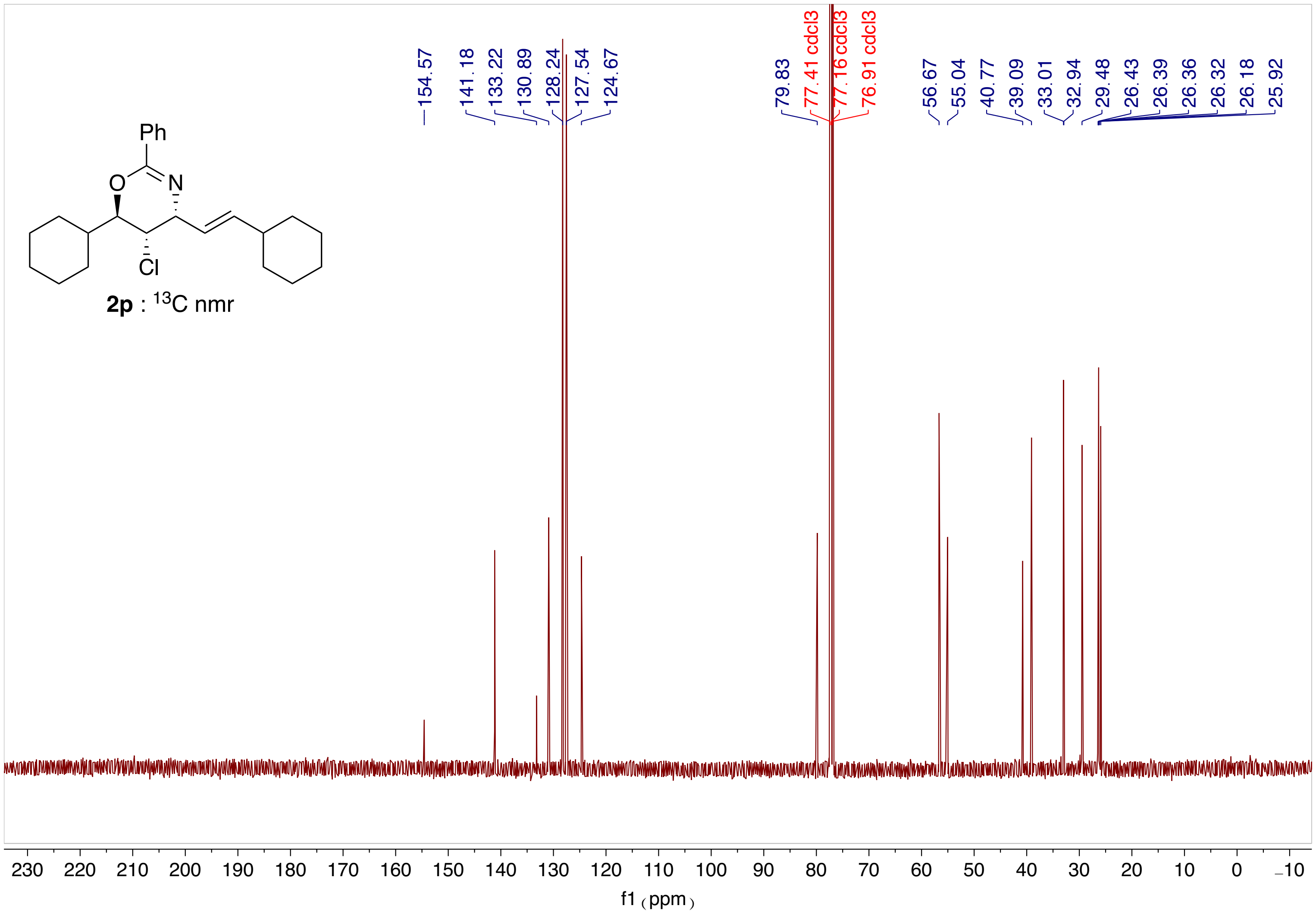
Ph
O
N
Cl
2p : ¹³C nmr
154.57
141.18
133.22
130.89
128.24
127.54
124.67
79.83
77.41 cdcl3
77.16 cdcl3
76.91 cdcl3
56.67
55.04
40.77
39.09
33.01
32.94
29.48
26.43
26.39
26.36
26.32
26.18
25.92
230 220 210 200 190 180 170 160 150 140 130 120 110 100 90 80 70 60 50 40 30 20 10 0 -10
f1 (ppm)

Compound 2r: (4*R*,5*S*,6*R*)-5-chloro-2-isopropyl-6-phenyl-4-((*E*)-styryl)-5,6-dihydro-4*H*-1,3-oxazine- ^{1}H NMR (500 MHz, CDCl$_3$); ^{13}C{^{1}H} NMR (126 MHz, CDCl$_3$)

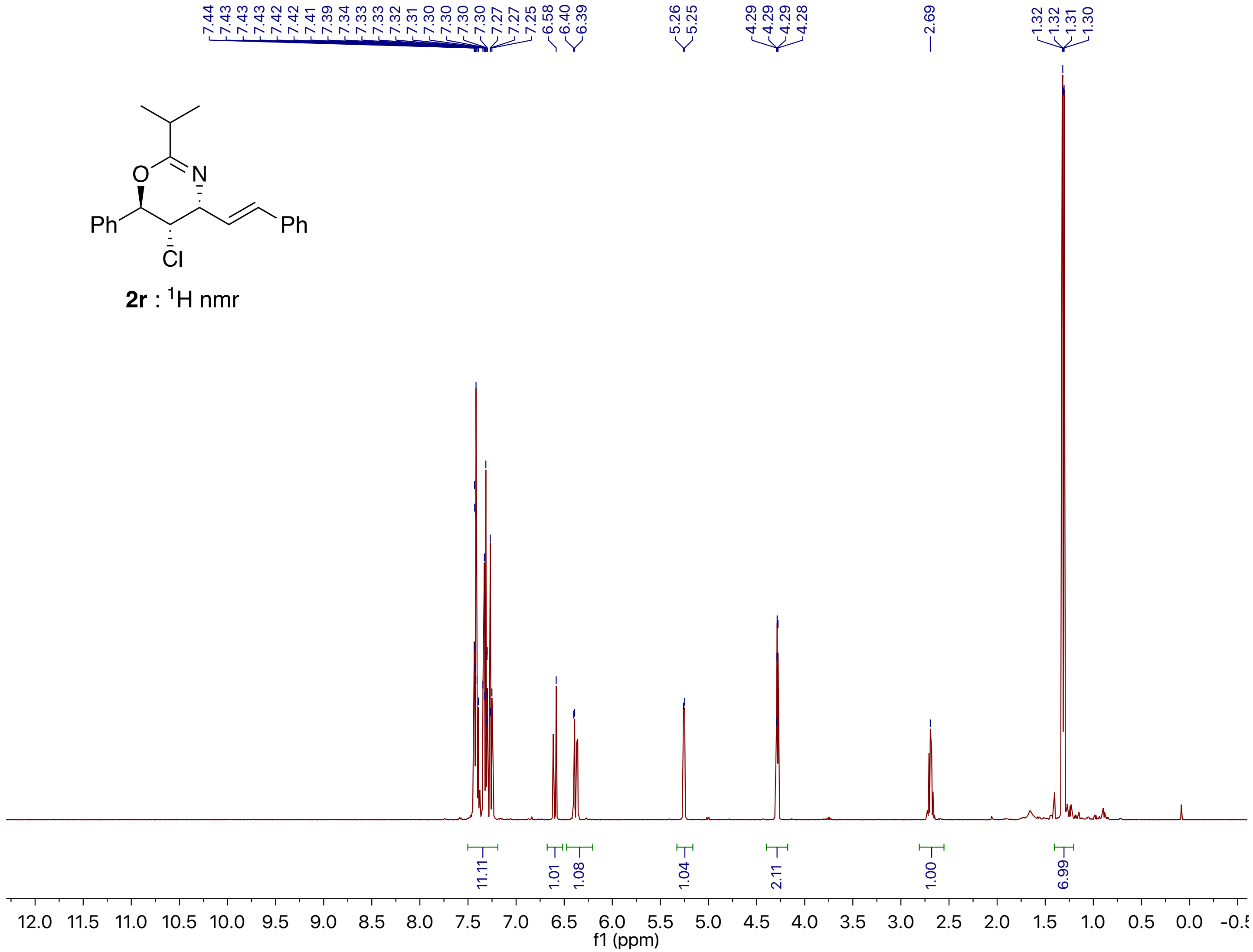

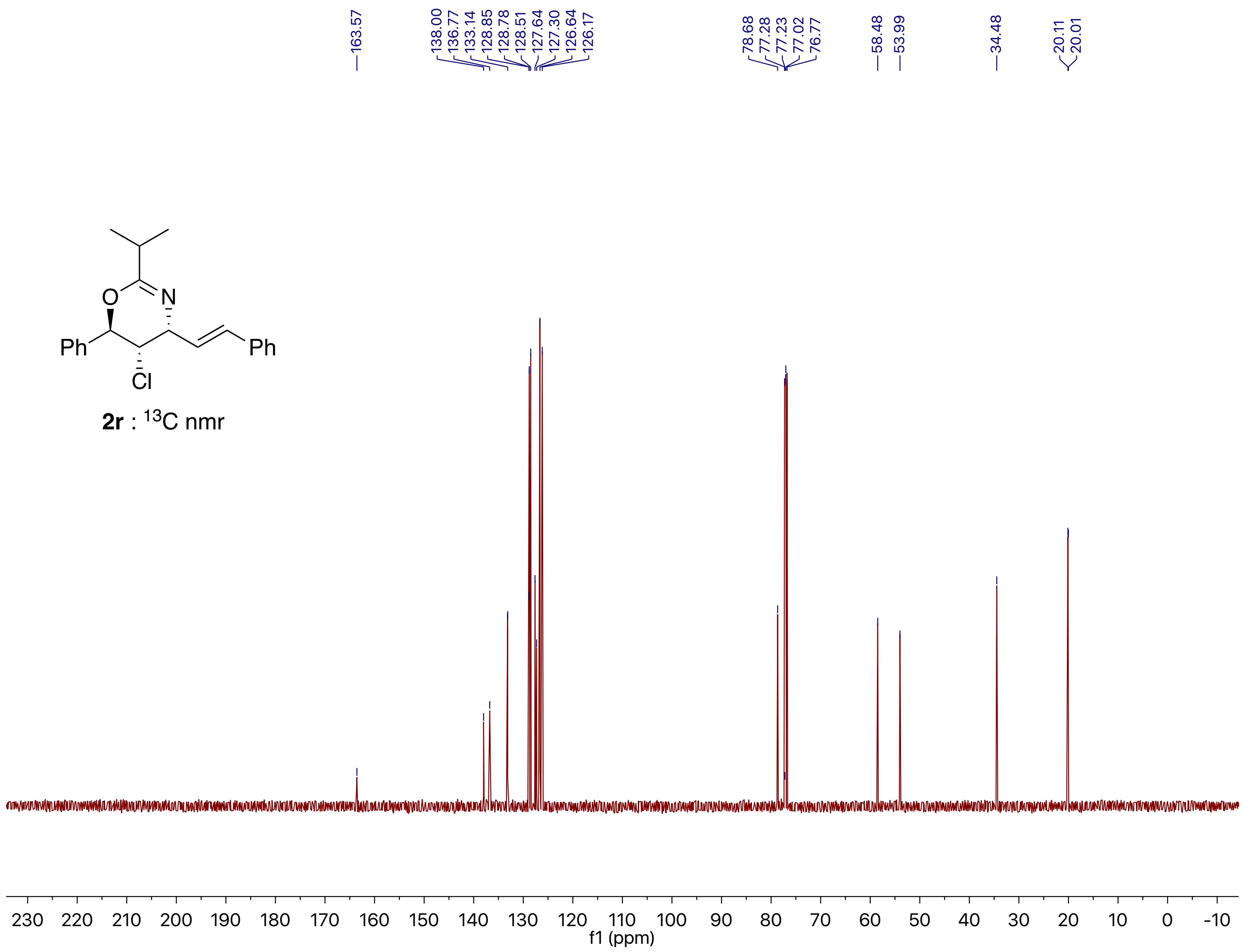
163.57
138.00
136.77
133.14
128.85
128.78
128.51
127.64
127.30
126.64
126.17
78.68
77.28
77.23
77.02
76.77
58.48
53.99
34.48
20.11
20.01
O
N
Ph
Ph
Cl
2r : 13C nmr
230 220 210 200 190 180 170 160 150 140 130 120 110 100 90 80 70 60 50 40 30 20 10 0 -10
f1 (ppm)

Compound 2s: (4*R*,5*S*,6*R*)-5-chloro-2-(4-methoxyphenyl)-6-phenyl-4-((*E*)-styryl)-5,6-dihydro-4*H*-1,3-oxazine- ^{1}H NMR (500 MHz, $CDCl_3$); $^{13}C\{^{1}H\}$ NMR (126 MHz, $CDCl_3$)

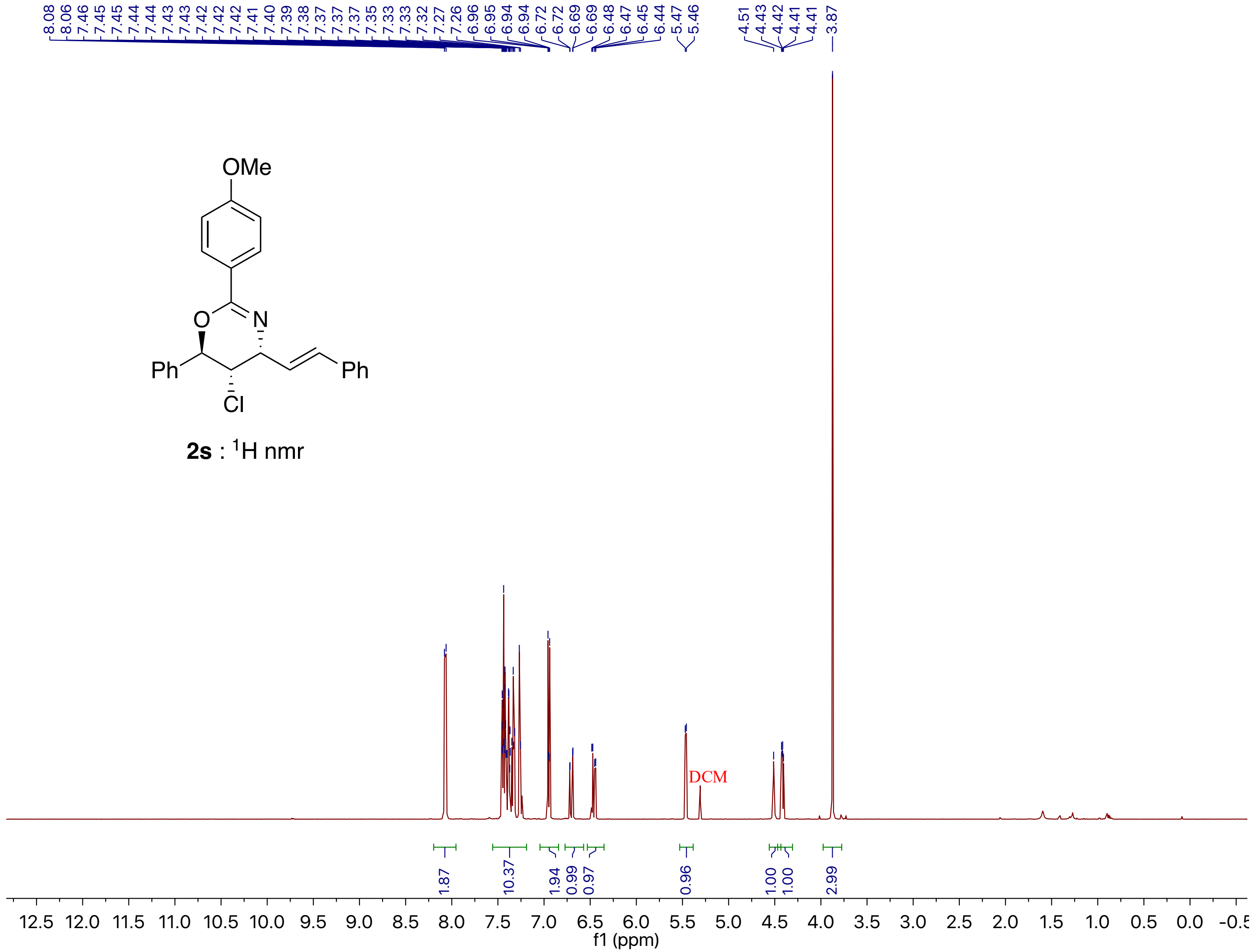

2s : ^{1}H nmr

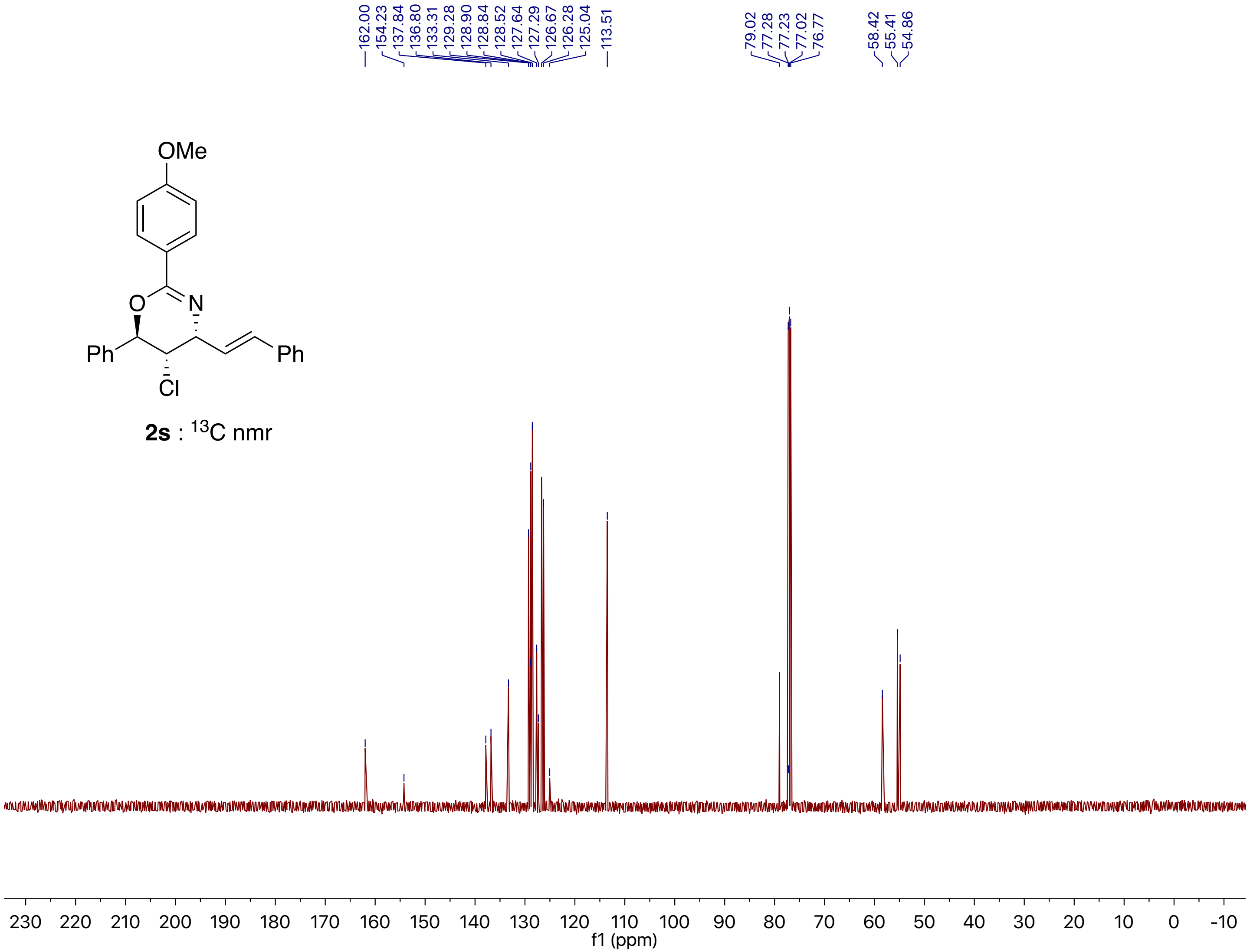
OMe
O
N
Ph
Ph
Cl
162.00
154.23
137.84
136.80
133.31
129.28
128.90
128.84
128.52
127.64
127.29
126.67
126.28
125.04
113.51
79.02
77.28
77.23
77.02
76.77
58.42
55.41
54.86
230 220 210 200 190 180 170 160 150 140 130 120 110 100 90 80 70 60 50 40 30 20 10 0 -10
f1 (ppm)

2s : ^{13}C nmr

Compound 2t: (4*R*,5*S*,6*R*)-2-(4-bromophenyl)-5-chloro-6-phenyl-4-((*E*)-styryl)-5,6-dihydro-4*H*-1,3-oxazine- ^{1}H NMR (500 MHz, $CDCl_3$); $^{13}C\{^{1}H\}$ NMR (126 MHz, $CDCl_3$)

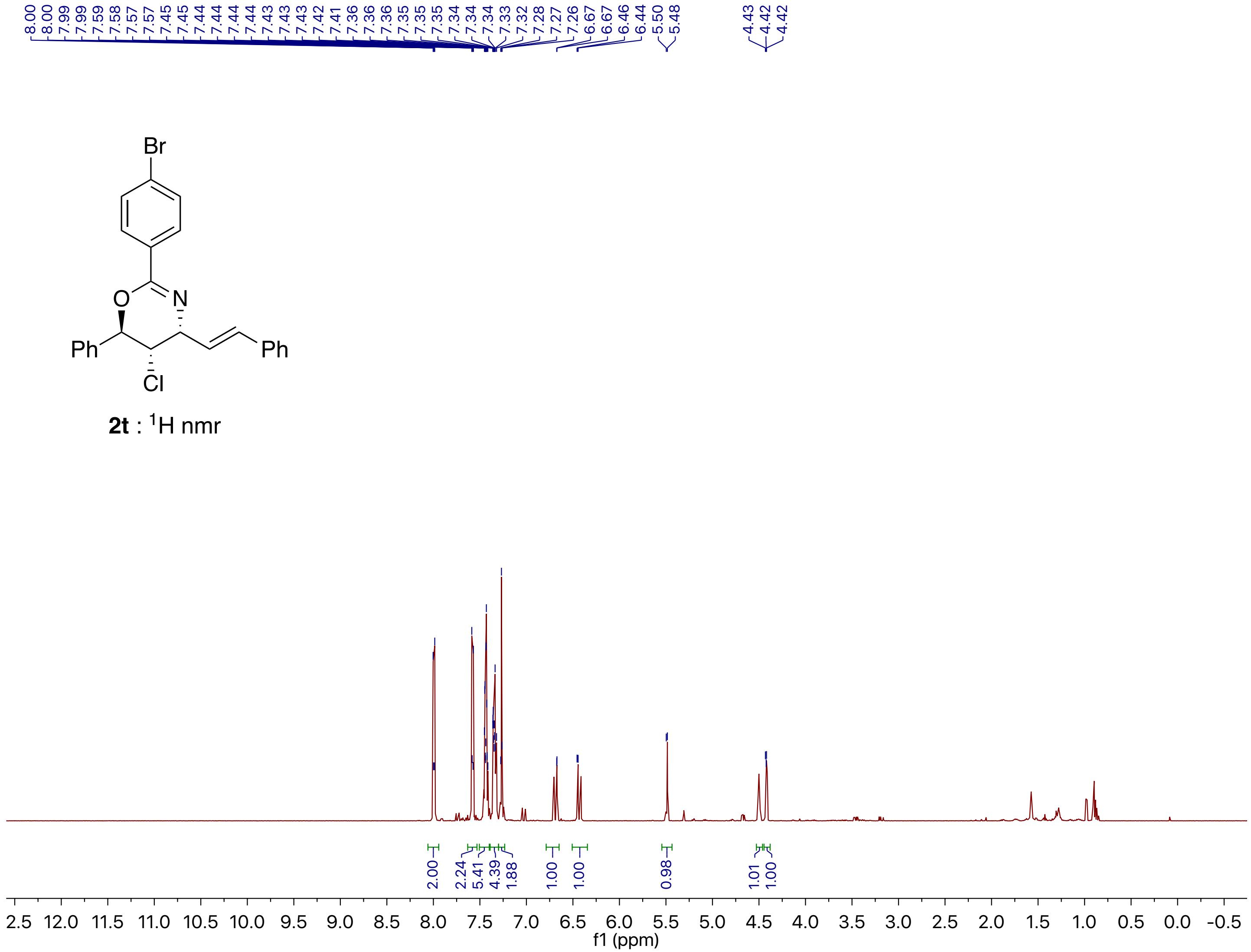

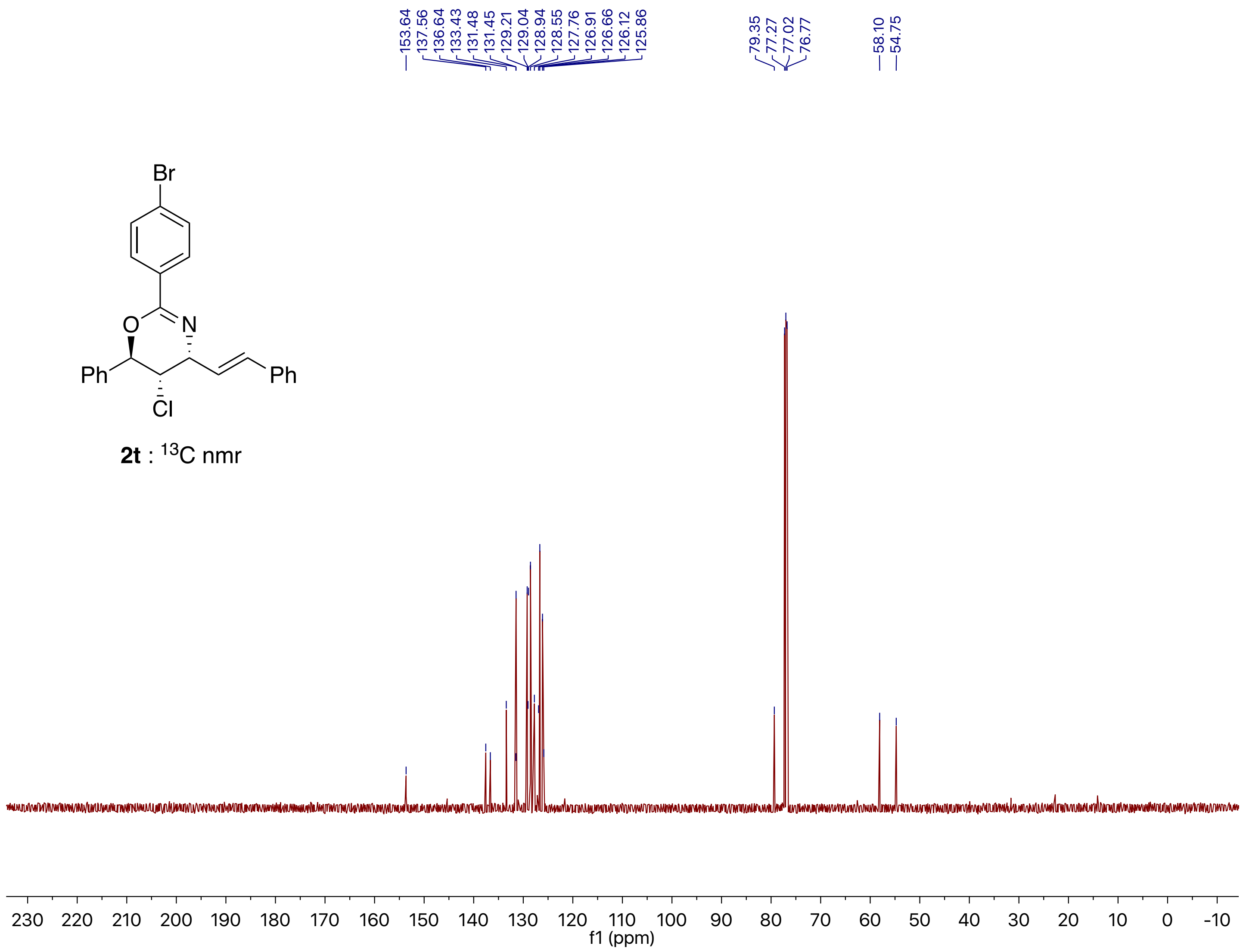
153.64
137.56
136.64
133.43
131.48
131.45
129.21
129.04
128.94
128.55
127.76
126.91
126.66
126.12
125.86
79.35
77.27
77.02
76.77
58.10
54.75
Br
O
N
Ph
Ph
Cl
2t : ^{13}C nmr
230 220 210 200 190 180 170 160 150 140 130 120 110 100 90 80 70 60 50 40 30 20 10 0 -10
f1 (ppm)

Compound 4: (1*R*,2*R*)-1-((4*R*,5*S*,6*R*)-5-chloro-2,6-diphenyl-5,6-dihydro-4*H*-1,3-oxazin-4-yl)-2-phenylethane-1,2-diol- ^{1}H NMR (500 MHz, $CDCl_3$); $^{13}C\{^{1}H\}$ NMR (126 MHz, $CDCl_3$)

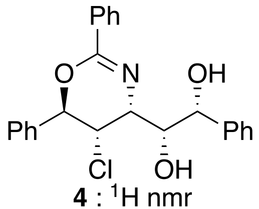

4 : ^{1}H nmr

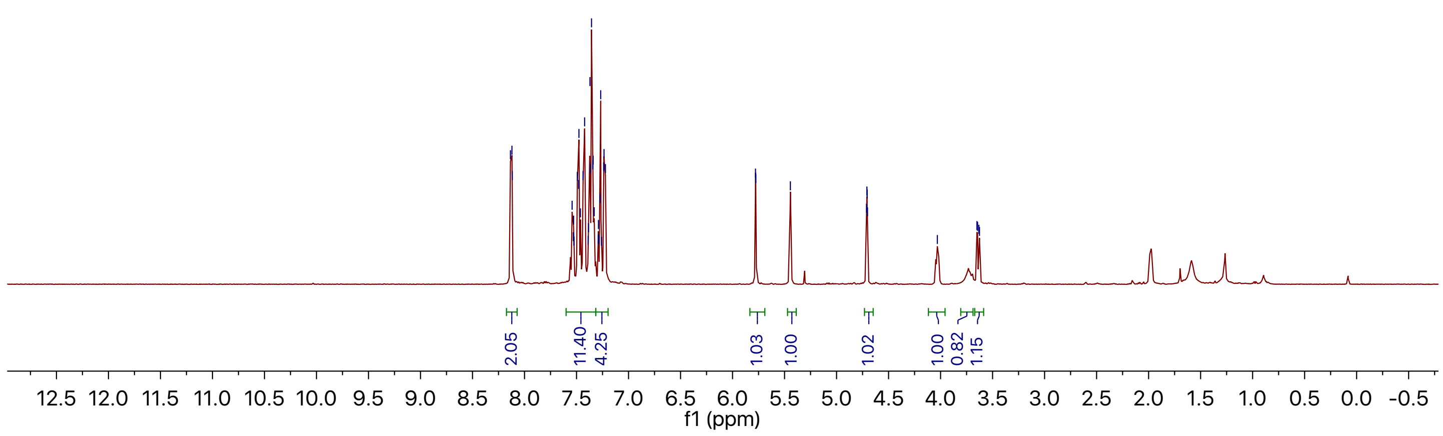

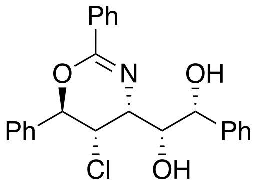

4 : ^{13}C nmr

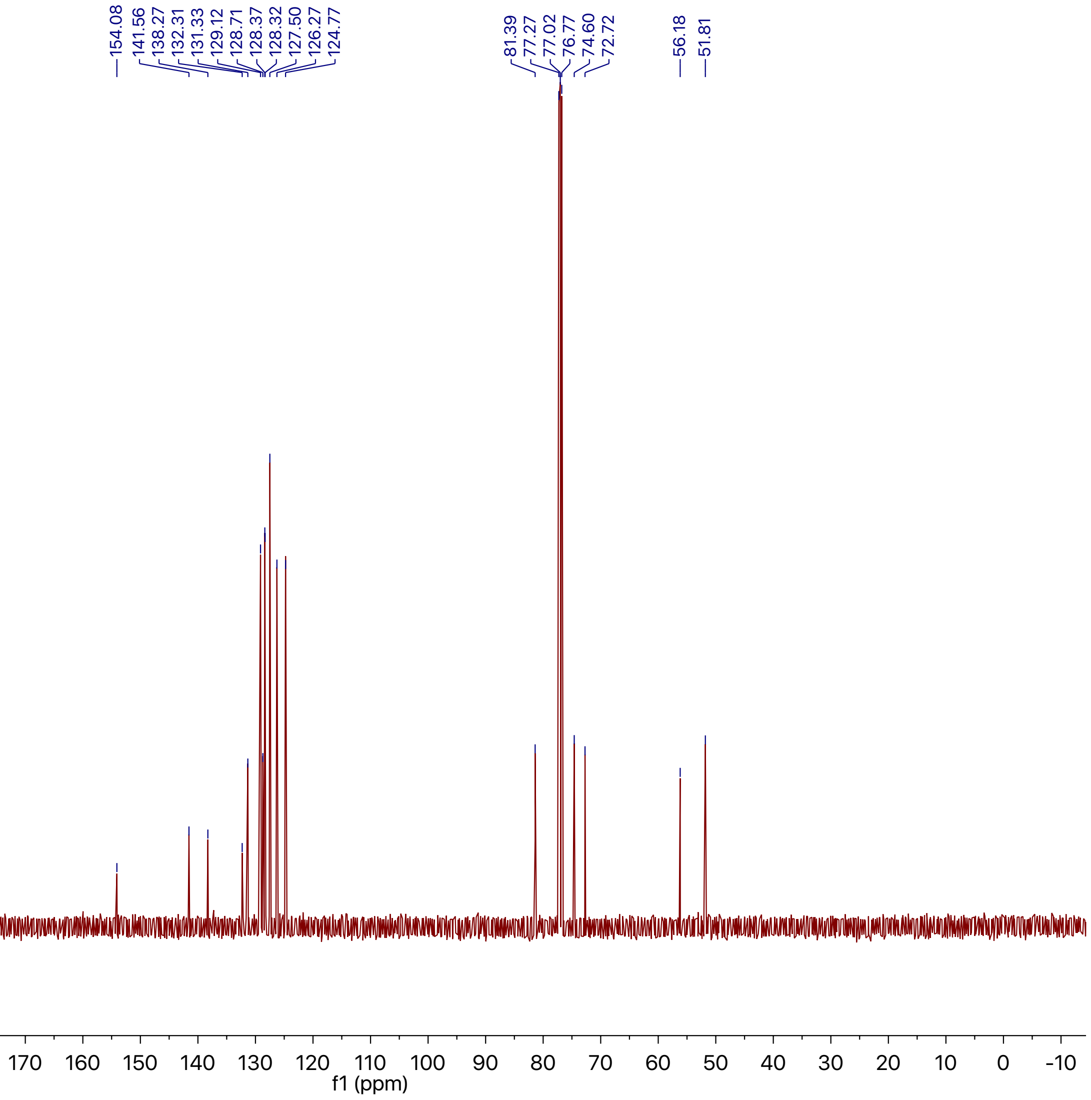
154.08
141.56
138.27
132.31
131.33
129.12
128.71
128.37
128.32
127.50
126.27
124.77
81.39
77.27
77.02
76.77
74.60
72.72
56.18
51.81
230 220 210 200 190 180 170 160 150 140 130 120 110 100 90 80 70 60 50 40 30 20 10 0 -10
f1 (ppm)

Compound 5: (4*R*,5*S*,6*R*)-5-chloro-2,6-diphenyl-4-((2*R*,3*R*)-3-phenyloxiran-2-yl)-5,6-dihydro-4*H*-1,3-oxazine- ^{1}H NMR (500 MHz, $CDCl_3$); $^{13}C\{^{1}H\}$ NMR (126 MHz, $CDCl_3$)

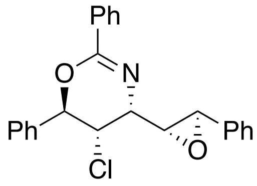

5 : ^{1}H nmr

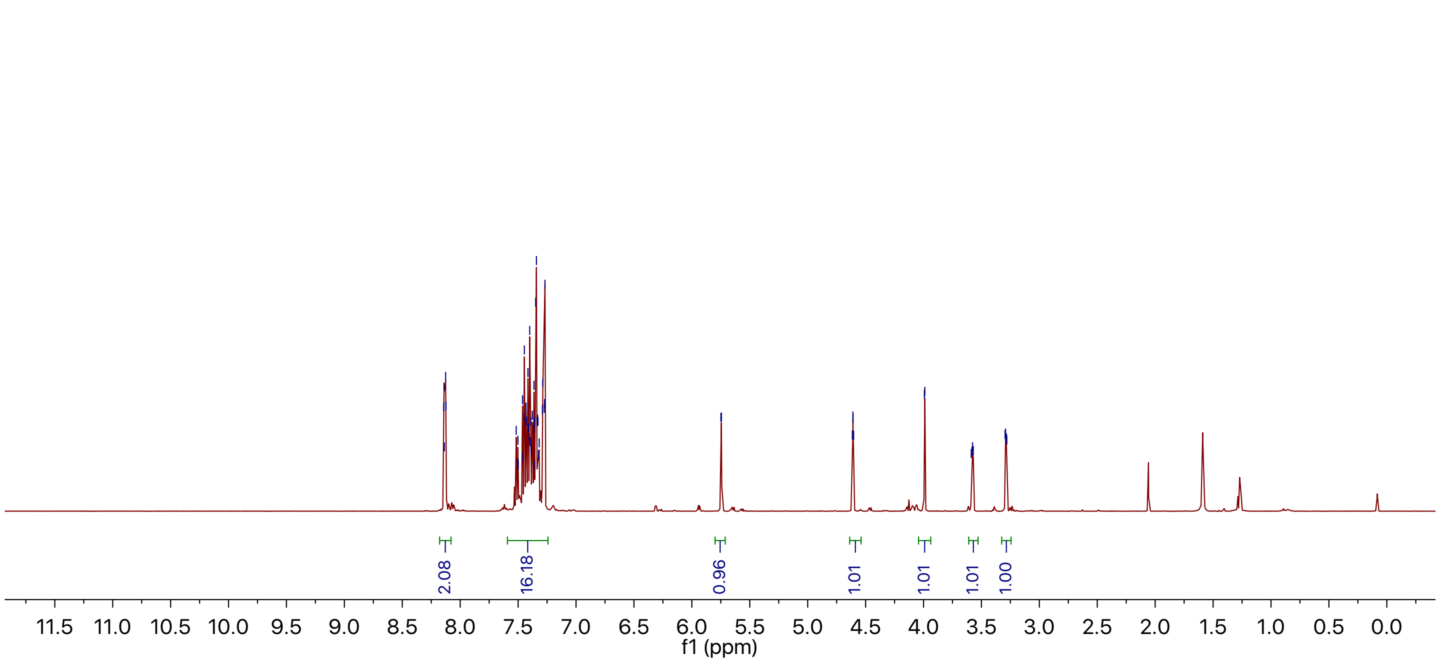

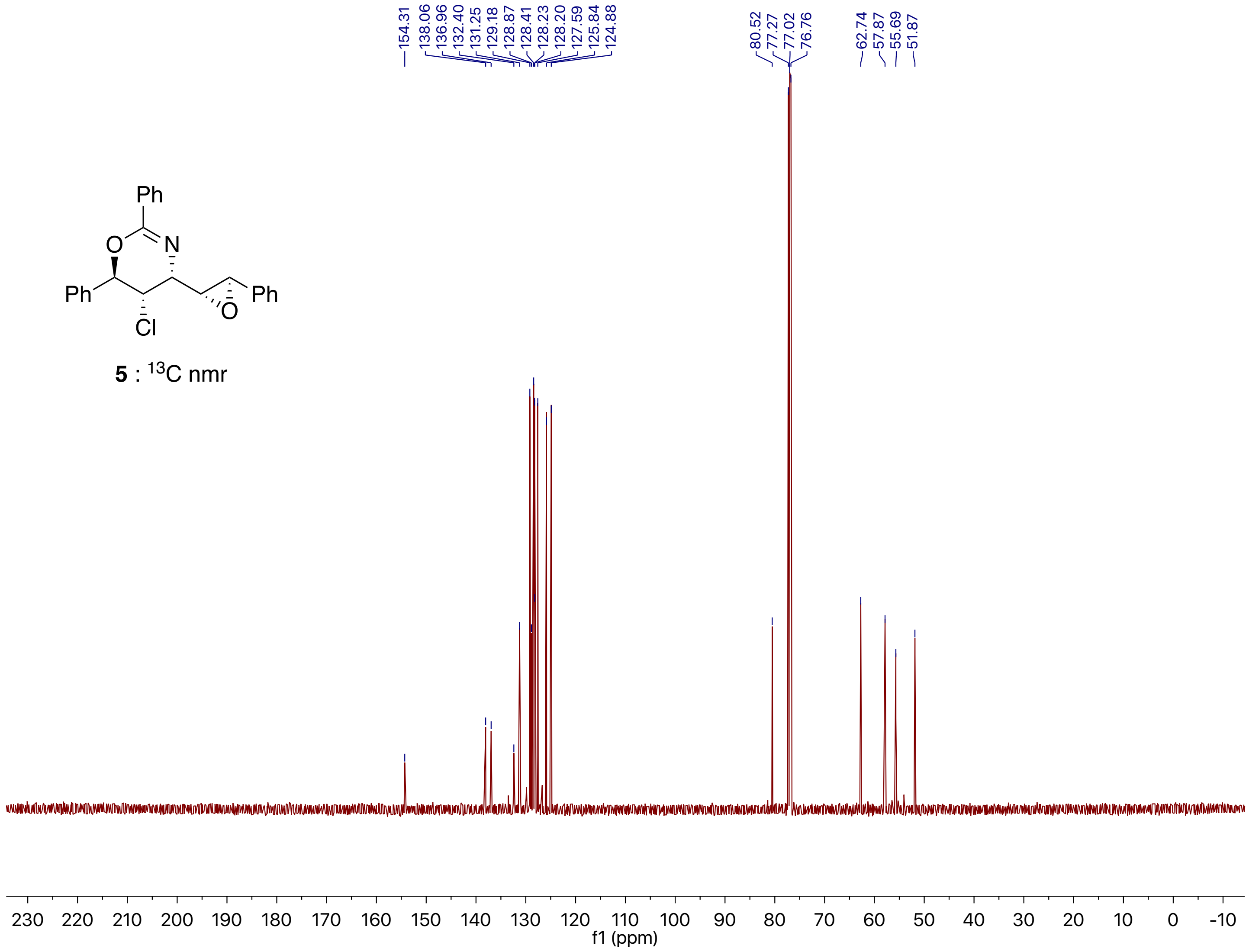
Ph
O
N
Ph
Ph
Cl
O
154.31
138.06
136.96
132.40
131.25
129.18
128.87
128.41
128.23
128.20
127.59
125.84
124.88
80.52
77.27
77.02
76.76
62.74
57.87
55.69
51.87
230 220 210 200 190 180 170 160 150 140 130 120 110 100 90 80 70 60 50 40 30 20 10 0 -10
f1 (ppm)

5 : ^{13}C nmr

Compound 6: 4-bromo-*N*-((3*R*,4*S*,5*R*,*E*)-4-chloro-5-hydroxy-1,5-diphenylpent-1-en-3-yl)benzamide-^{1}H NMR (500 MHz, $CDCl_3$); $^{13}C\{^{1}H\}$ NMR (126 MHz, $CDCl_3$)

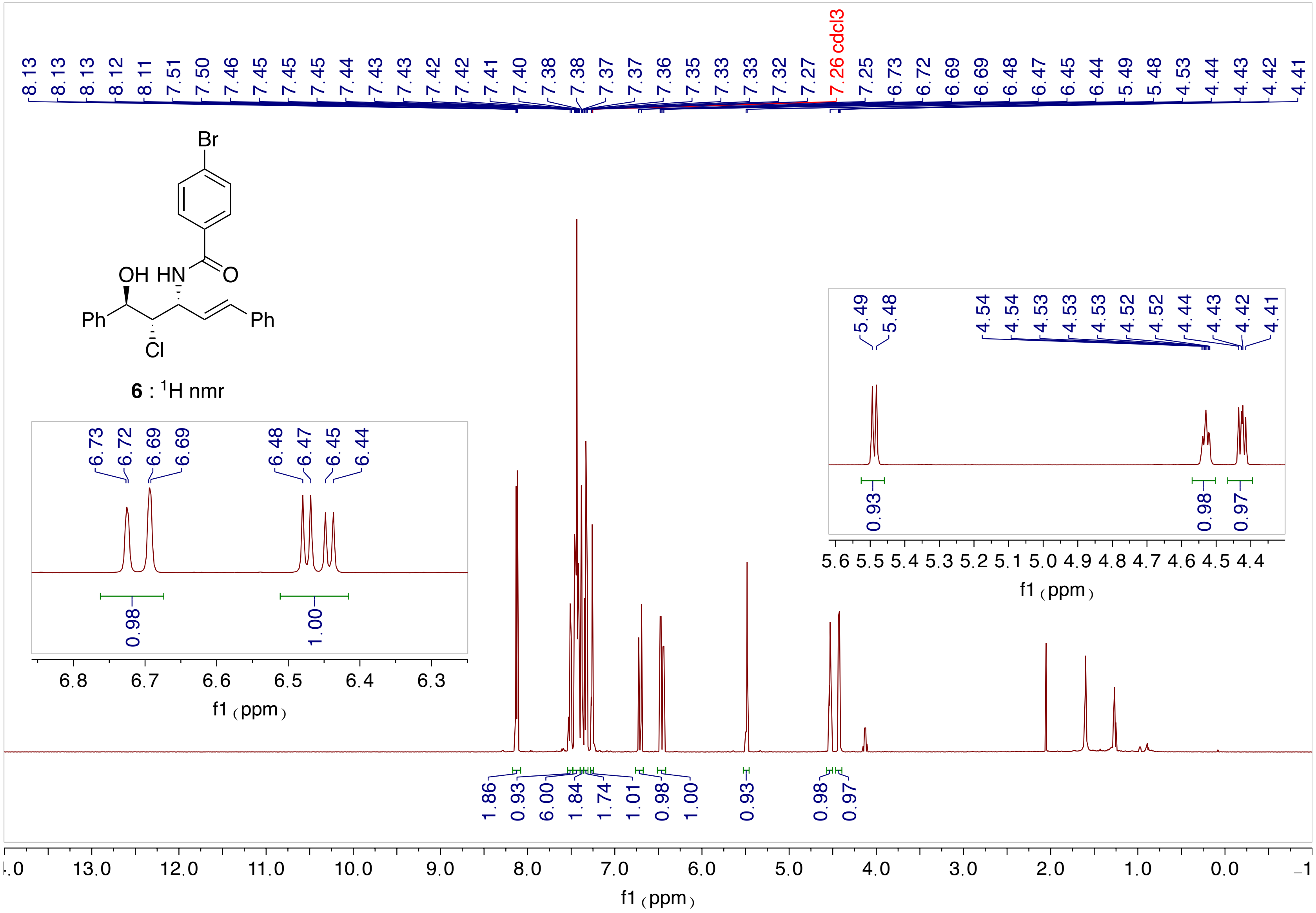
6 : 1H nmr
Br
OH HN
O
Ph
Ph
Cl
8.13 8.13 8.13 8.12 8.11 7.51 7.50 7.46 7.45 7.45 7.45 7.44 7.43 7.43 7.42 7.42 7.41 7.40 7.38 7.38 7.37 7.37 7.36 7.35 7.33 7.33 7.32 7.27 7.26 cdcl3 7.25 6.73 6.72 6.69 6.69 6.48 6.47 6.45 6.44 5.49 5.48 4.53 4.44 4.43 4.42 4.41
1.86 0.93 6.00 1.84 1.74 1.01 0.98 1.00 0.93 0.98 0.97
f1 (ppm)

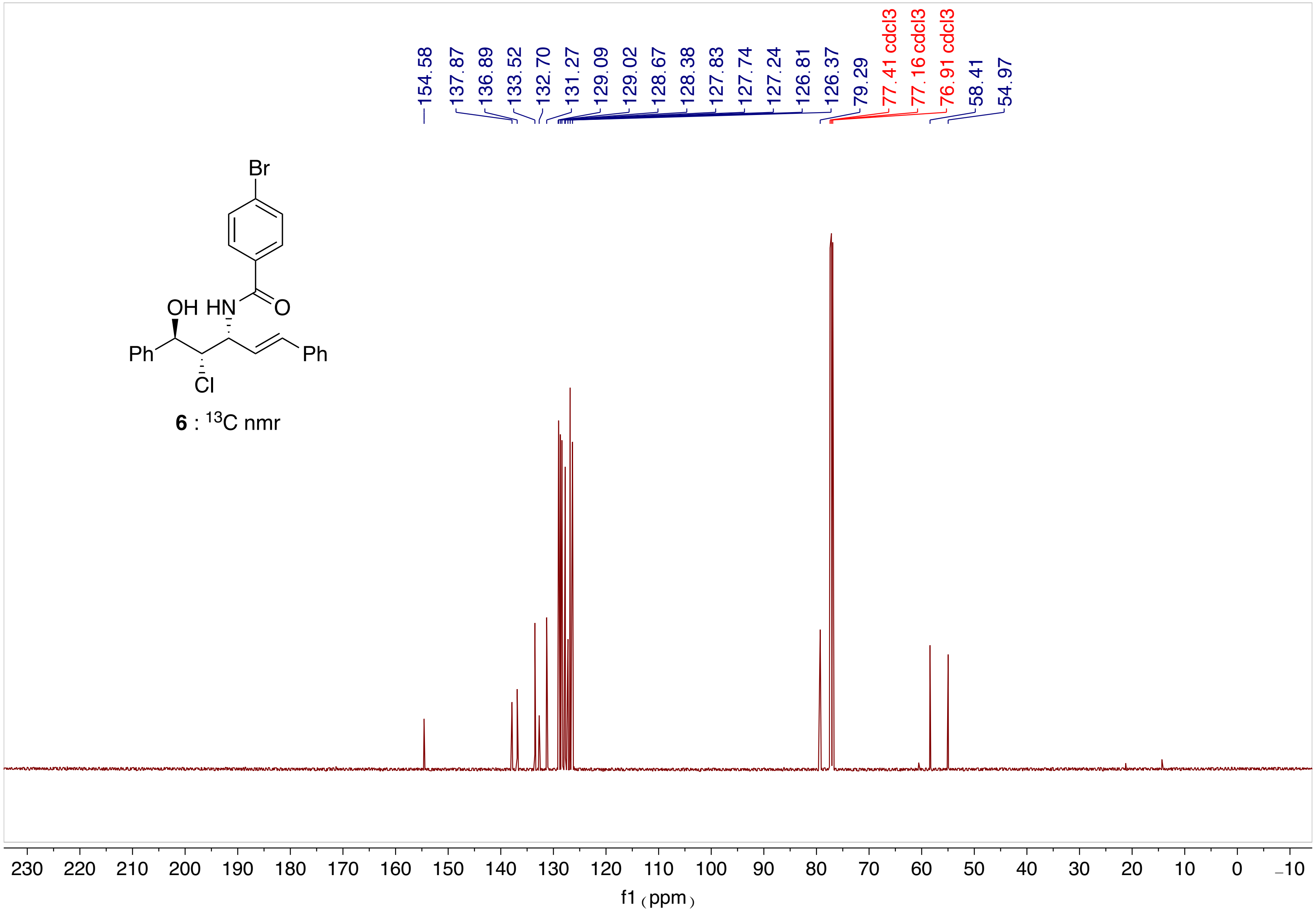
154.58
137.87
136.89
133.52
132.70
131.27
129.09
129.02
128.67
128.38
127.83
127.74
127.24
126.81
126.37
79.29
77.41 cdcl3
77.16 cdcl3
76.91 cdcl3
58.41
54.97
Br
OH HN
O
Ph
Ph
Cl
6 : 13C nmr
230 220 210 200 190 180 170 160 150 140 130 120 110 100 90 80 70 60 50 40 30 20 10 0 -10
f1 (ppm)
